# Comprehensive Evaluation of the Genetic Basis of Keratoconus: New Perspectives for Clinical Translation

**DOI:** 10.1167/iovs.65.12.32

**Published:** 2024-10-22

**Authors:** Miriam Cerván-Martín, Inmaculada Higueras-Serrano, Sara González-Muñoz, Andrea Guzmán-Jiménez, Blas Chaves-Urbano, Rogelio J. Palomino-Morales, Arancha Poo-López, Luis Fernández-Vega-Cueto, Jesús Merayo-Lloves, Ignacio Alcalde, Lara Bossini-Castillo, F. David Carmona

**Affiliations:** 1 Departamento de Genética e Instituto de Biotecnología, Centro de Investigación Biomédica, Universidad de Granada, Granada, Spain; 2 Instituto de Investigación Biosanitaria ibs.GRANADA, Granada, Spain; 3 Computational Oncology Group, Spanish National Cancer Research Centre, Madrid, Spain; 4 Departamento de Bioquímica y Biología Molecular I, Universidad de Granada, Granada, Spain; 5 Instituto Universitario Fernández-Vega, Universidad de Oviedo, Fundación de Investigación Oftalmológica, Oviedo, Spain; 6 Instituto de Investigación Sanitaria del Principado de Asturias, Oviedo, Spain

**Keywords:** keratoconus, genetic associations, MINK1, polygenic risk score, acetylcysteine

## Abstract

**Purpose:**

Keratoconus (KC) is a corneal disorder with complex etiology, apparently involving both genetic and environmental factors, characterized by progressive thinning and protrusion of the cornea. We aimed to identify novel genetic regions associated with KC susceptibility, elucidate relevant genes for disease development, and explore the translational implications for therapeutic intervention and risk assessment.

**Methods:**

We conducted a genome-wide association study (GWAS) that integrated previously published data with newly generated genotyping data from an independent European cohort. To evaluate the clinical translation of our results, we performed functional annotation, gene prioritization, polygenic risk score (PRS), and drug repositioning analyses.

**Results:**

We identified two novel genetic loci associated with KC, with rs2806689 and rs807037 emerging as lead variants (*P* = 1.71E-08, odds ratio [OR] = 0.88; *P* = 1.93E-08, OR = 1.16, respectively). Most importantly, we identified 315 candidate genes influenced by confirmed KC-associated variants. Among these, *MINK1* was found to play a pivotal role in KC pathogenesis through the WNT signaling pathway. Moreover, we developed a PRS model that successfully differentiated KC patients from controls (*P* = 7.61E-16; area under the curve = 0.713). This model has the potential to identify individuals at high risk for developing KC, which could be instrumental in early diagnosis and management. Additionally, our drug repositioning analysis identified acetylcysteine as a potential treatment option for KC, opening up new avenues for therapeutic intervention.

**Conclusions:**

Our study provides valuable insights into the genetic and molecular basis of KC, offering new targets for therapy and highlighting the clinical utility of PRS models in predicting disease risk.

Keratoconus (KC) is an ocular condition characterized by progressive thinning and bulging of the cornea, leading to irregular astigmatism and a reduction in both visual quality and quantity.^1^^,^^2^ Although previously considered non-inflammatory, several proinflammatory cytokines and proteinases have been associated with the disease.^3^ Both a family history of the disease and the habit of rubbing the eyes have frequently been linked to KC.^4^ Nocturnal ocular compression has been also identified as a potential risk factor.^5^ This asymmetric and bilateral eye disorder typically first appears during adolescence and progresses into the 30s or 40s.^1^ Despite extensive research, knowledge about the etiology of KC remains limited due to challenges in studying and acquiring corneal samples, as well as in establishing suitable animal models for its investigation.^2^ In this regard, KC is considered a complex disease in which both environmental and genetic factors interact for disease development.^6^

Common variation in the human genome—specifically, single-nucleotide polymorphisms (SNPs)—plays a pivotal role in the susceptibility to complex traits. Genome-wide association studies (GWASs) are of great value for analyzing the effects of SNPs on the onset and progression of this type of disorder.^7^ This strategy allows the simultaneous exploration of millions of genetic variants across the genome without the constraints of predefined hypotheses, leading to remarkable advancements in biomedical discovery over the past decade.^8^ Furthermore, insights gained from GWAS findings can be used to calculate polygenic risk scores (PRSs) for a given phenotype, which combine the effect sizes of SNPs associated with such a phenotype into a single score for predictive or clinical purposes, showcasing the translational potential of GWAS results.^9^ However, despite the multiple advantages of GWASs, this approach has some important limitations, including a high number of type 2 (false-negative) errors located in the so-called “gray zone,” where signals are close to the significance threshold (*P* < 5E-08).^10^ These errors can occur due to a lack of statistical power, which is indeed one of the major drawbacks of this analytical approach.

Different GWASs have been conducted to unravel the genetic component of KC, focusing on both KC and closely related phenotypes, such as central corneal thickness (CCT), corneal hysteresis (CH), or corneal resistance factor (CRF).^11^^–^^15^ To date, the most extensive GWAS for KC was performed by Hardcastle and colleagues in 2021.^16^ In this study, a cohort comprised of 4669 KC cases and 116,547 unaffected controls was analyzed, identifying 36 loci associated with the disease. The majority of these loci were implicated in processes such as the dysregulation of corneal collagen matrix integrity or cell differentiation pathways.^16^ Also noteworthy is the study on CCT by Choquet et al.^15^ In this analysis of 44,039 individuals, 98 associations with CCT were identified, 20 of which were also found to be significantly associated with KC.

The main objective of this study was to increase the current knowledge on the genetic and molecular basis of KC by conducting a GWAS in an independent Spanish cohort of KC patients and controls. This allowed us not only to perform a replication study of the GWAS by Hardcastle et al.^16^ but also to identify potential novel associations with the disease within the gray zone of their findings. Most importantly, we assessed the predictive value of a PRS method for KC susceptibility in our Spanish case–control cohort using previously published data.^15^^,^^16^ Additionally, we gained valuable functional and translational insights derived from genetic data through an extensive functional analysis of all identified associations from the meta-analysis, alongside a comprehensive drug repositioning study.

## Methods

### Study Population

The case–control analysis of the newly genotyped cohort included 143 patients diagnosed with KC and 987 unaffected controls, all from Spain and with European ancestry. All participants provided informed written consent before enrolling in the study, and all DNA samples were irreversibly anonymized. Subsequent procedures adhered to the tenets of the Declaration of Helsinki and received approval from the ethics committee, CEIM/CEI Provincial de Granada (Andalusia, Spain; approval no. 10/18).

All KC participants of the Spanish cohort were recruited by the Fernandez-Vega Ophthalmological Institute (Oviedo, Spain). The Spanish controls were provided by the National DNA Bank (University of Salamanca, Spain). A detailed description of the cohort selection criteria is available in Supplementary Methods. The case–control cohort used for the meta-analysis was described in Hardcastle et al.^16^ and was comprised of a total of 4669 KC cases and 116,547 unaffected subjects.

### Generation of New Large-Scale Genotyping Data

Genomic DNA from blood cells was genotyped using the Infinium GSA-24 v3.0 platform (Illumina, San Diego, CA, USA), with quality control applied using PLINK 1.9^17^ and R (R Foundation for Statistical Computing, Vienna, Austria), as described in Supplementary Methods. Whole-genome genotype imputation for chromosomes 1 to 22 was performed using the TOPMed haplotype data as a reference panel in the TOPMed Imputation Server (National Institutes of Health).^18^^,^^19^ After all of the quality-control procedures (Supplementary Methods) were completed, the Spanish case–control dataset was comprised of 133 KC patients and 983 controls with genotype information for 6,486,492 SNPs.

### Statistical Analysis

PLINK and R were used for the case–control analyses in the Spanish cohort controlling for possible population stratification (Supplementary Methods, Supplementary Fig. S1). A meta-analysis between the Spanish results and those reported by Hardcastle et al.^16^ (available in the NHGRI-EBI Catalog of GWASs) was conducted using the inverse-variance method under a fixed-effects model, as described in Supplementary Methods.

### Evaluation of Functional Relevance and Clinical Translation of Association Signals

A PRS analysis was conducted with PRSice-2^20^ to predict the genetic predisposition to KC, using data from studies by Hardcastle et al.^16^ and Choquet et al.^15^ The Spanish cohort dataset was used for model evaluation. Functional relevance of associated variants was assessed using FUMA,^21^ and genes were categorized in tiers by coding and non-coding effects. Enrichment analyses of protein–protein interactions were conducted for genes in tiers 1 and 2 with STRING^22^ to provide an illustrative picture of the putative functional role of prioritized genes. Finally, drug repositioning analysis screened the DrugBank database^23^ for potential KC treatments, focusing on genes in tiers 1 and 2 for approved drugs with relevant mechanisms in KC. A detailed description of these methods is available in Supplementary Methods.

## Results

### Genetic Association Analysis

**Figure 1.**
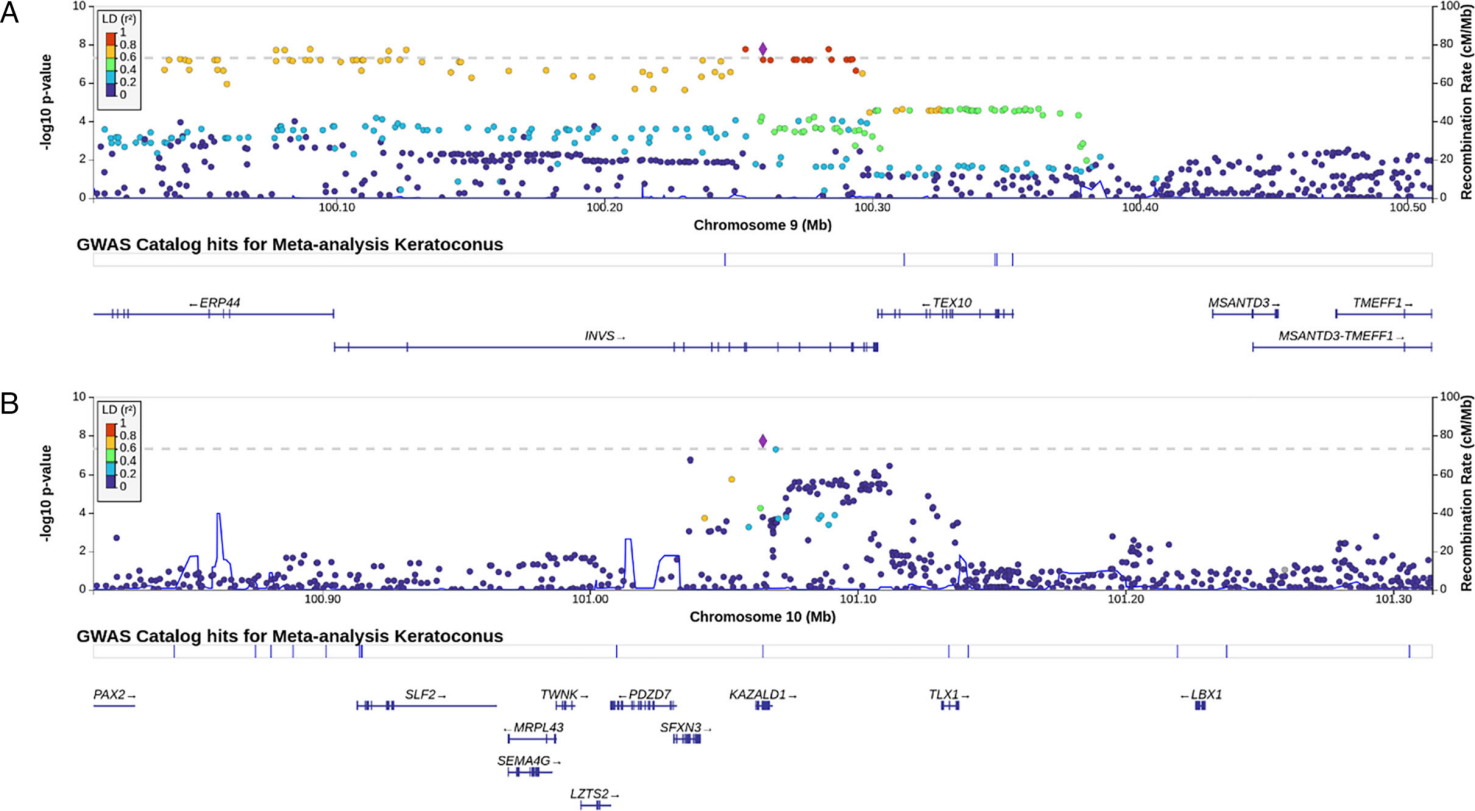
(**A**, **B**) Regional plots for the novel keratoconus association signals identified in this study: rs2806689 (**A**) and rs807037 (**B**). The plots display −log_10_ *P* values for SNPs within a region flanking 500 kb on both sides of the top SNPs. Lead variants are highlighted in *purple*, and the *r*^2^ values of other variants are represented by different colors. Genes in the region and their transcription direction (*arrows*) are indicated. The *y*-axis shows the recombination rates, aligned with chromosomal positions on the *x*-axis.

We performed a case–control comparison of minor allele frequencies at the genome-wide level in the Spanish cohort and meta-analyzed the obtained results with the summary statistics generated by Hardcastle et al.^16^ Two novel KC-associated genetic loci were observed, with rs2806689 and rs807037 representing the top SNPs (*P* = 1.71E-08, odds ratio [OR] = 0.88; *P* = 1.93E-08, OR = 1.16, respectively) (Table, Fig. 1). Both variants were located in the gray zone of the Hardcastle GWAS^16^ (*P*_rs2806689_ = 2.69E-07, *P*_rs807037_ = 5.84E-08) and showed a nominal association (*P* < 0.05) in the Spanish GWAS (*P*_rs2806689_ = 2.47E-02, *P*_rs807037_ = 2.54E-02). No significant heterogeneity between the ORs was evident either for rs2806689 (OR_HARDCASTLE_ = 0.88, OR_SPANISH_ = 0.74, *Q* = 0.20, *I*^2^ = 38.18) or for rs807037 (OR_HARDCASTLE_ = 1.15, OR_SPANISH_ = 1.36, *Q* = 0.23, *I*^2^ = 30.65) (Table).

**Table.** Summary of Genetic Variants Associated With KC in the GWAS Meta-Analysis, Including Newly Generated Data From a Spanish Cohort and From Hardcastle et al.^13^

				Spanish Study (*N* = 1116)		Hardcastle et al. Study (*N* = 121,216)		Meta-Analysis			
CHR	GRCh38 bp	Variant ID	Ref	*P*	OR (95% CI)	*P*	OR (95% CI)	*P*	OR	*Q*	*I* ^2^
1	169088731	rs1200108	A	2.88E-01	0.87 (0.67–1.13)	4.52E-10	0.85 (0.81–0.89)	3.14E-10	0.85	0.89	0.00
1	175024486	rs6669560	T	7.00E-01	1.06 (0.79–1.41)	2.92E-09	1.17 (1.11–1.23)	2.42E-09	1.17	0.50	0.00
1	207808074	rs761276	A	2.47E-02	0.74 (0.57–0.96)	8.02E-09	0.87 (0.83–0.91)	7.55E-10	0.87	0.23	29.29
2	141120826	rs116792882	T	9.70E-02	5.47 (0.74–40.72)	4.82E-10	0.5 (0.41–0.62)	1.23E-09	0.51	0.02	81.47
3	29352153	rs11129361	A	6.27E-02	1.3 (0.99–1.71)	2.19E-07	1.15 (1.09–1.21)	3.26E-08	1.15	0.39	0.00
3	172279709	rs4894414	T	5.61E-01	1.11 (0.79–1.56)	1.21E-26	1.36 (1.28–1.43)	6.46E-27	1.35	0.25	25.80
5	53312130	rs252035	T	1.85E-01	0.83 (0.62–1.1)	7.79E-18	0.79 (0.75–0.84)	4.58E-19	0.79	0.76	0.00
5	122087230	rs2731657	A	6.41E-01	0.94 (0.72–1.23)	1.98E-20	0.79 (0.75–0.83)	2.34E-20	0.79	0.21	35.32
6	39586825	rs10947821	T	7.03E-02	1.33 (0.98–1.82)	1.30E-07	1.18 (1.11–1.26)	3.34E-08	1.19	0.45	0.00
6	50953889	rs6904450	A	7.83E-01	0.95 (0.68–1.33)	6.44E-11	0.83 (0.78–0.88)	1.76E-10	0.83	0.42	0.00
6	51742973	rs9382005	C	9.55E-01	1.01 (0.77–1.31)	2.31E-09	0.86 (0.82–0.9)	4.94E-09	0.86	0.25	24.65
6	75061719	rs118043261	A	5.65E-04	2.46 (1.48–4.11)	1.35E-14	1.72 (1.5–1.98)	8.46E-17	1.76	0.19	43.01
6	88852991	rs1321085	A	1.55E-01	1.29 (0.91–1.83)	1.00E-07	1.17 (1.1–1.24)	4.61E-08	1.17	0.59	0.00
8	23524256	rs73228208	A	5.32E-02	0.69 (0.48–1.01)	7.01E-08	0.85 (0.8–0.9)	1.43E-08	0.85	0.29	11.59
8	94907003	rs7820818	A	4.39E-01	0.9 (0.68–1.18)	4.06E-10	0.85 (0.81–0.9)	1.73E-10	0.85	0.70	0.00
9	13555514	rs1556575	A	2.41E-01	0.84 (0.62–1.13)	3.06E-19	0.78 (0.74–0.83)	2.66E-20	0.78	0.65	0.00
**9**	**100259252**	**rs2806689**	**T**	**2.47E-02**	**0.74 (0.57** **–** **0.96)**	**2.69E-07**	**0.88 (0.84** **–** **0.93)**	**1.71E-08**	**0.88**	**0.20**	**38.18**
9	108684737	rs7859737	A	9.34E-01	1.01 (0.78–1.32)	3.59E-11	0.85 (0.82–0.9)	1.60E-11	0.85	0.20	38.54
9	134548237	rs3118518	A	1.61E-02	1.38 (1.06–1.8)	1.83E-28	1.31 (1.25–1.37)	1.06E-29	1.31	0.69	0.00
9	136967018	rs7019538	T	4.68E-01	0.91 (0.71–1.17)	2.69E-11	0.84 (0.8–0.89)	6.15E-12	0.84	0.55	0.00
10	53436353	rs117905623	T	8.65E-01	0.94 (0.46–1.92)	1.89E-08	0.68 (0.6–0.78)	1.87E-08	0.69	0.38	0.00
**10**	**101064592**	**rs807037**	**C**	**2.54E-02**	**1.36 (1.04** **–** **1.79)**	**5.84E-08**	**1.15 (1.1** **–** **1.22)**	**1.93E-08**	**1.16**	**0.23**	**30.65**
10	119068783	rs10886377	C	3.49E-01	0.88 (0.66–1.16)	6.29E-12	0.84 (0.8–0.88)	3.69E-12	0.84	0.77	0.00
11	791462	rs4963153	A	1.49E-02	1.39 (1.07–1.8)	3.62E-26	1.3 (1.24–1.37)	5.12E-27	1.30	0.64	0.00
11	47641380	rs7120548	T	2.77E-01	0.87 (0.67–1.12)	1.91E-07	0.87 (0.83–0.92)	3.26E-08	0.87	0.98	0.00
11	95575690	rs11021221	A	6.92E-01	0.92 (0.61–1.38)	1.49E-09	1.23 (1.15–1.31)	2.21E-09	1.22	0.17	47.12
12	14137583	rs17340879	T	4.16E-01	1.48 (0.57–3.82)	1.77E-09	0.61 (0.52–0.72)	6.08E-09	0.63	0.07	69.51
12	51362485	rs3782473	T	2.38E-01	1.21 (0.88–1.68)	6.60E-10	1.19 (1.12–1.25)	1.94E-10	1.19	0.90	0.00
13	40536133	rs2755238	T	6.02E-05	0.54 (0.4–0.73)	1.49E-34	0.63 (0.59–0.68)	6.72E-39	0.62	0.31	1.23
13	41324269	rs9566743	A	3.54E-05	0.47 (0.33–0.67)	1.86E-09	0.78 (0.72–0.85)	7.67E-12	0.76	0.01	86.37
13	73070755	rs17285550	A	6.05E-01	0.93 (0.7–1.23)	2.84E-12	0.83 (0.79–0.88)	7.40E-13	0.83	0.44	0.00
15	51072532	rs11634895	A	3.16E-01	0.87 (0.66–1.14)	7.88E-10	0.86 (0.82–0.91)	1.41E-10	0.86	0.93	0.00
15	67174959	rs12912045	T	2.77E-01	1.19 (0.87–1.61)	4.57E-26	0.73 (0.69–0.78)	2.61E-25	0.74	0.00	89.23
16	88265987	rs35542380	C	9.94E-02	1.27 (0.96–1.68)	4.32E-19	1.26 (1.2–1.32)	8.35E-20	1.26	0.97	0.00
17	5027076	rs12603055	C	6.64E-02	0.7 (0.49–1.02)	2.23E-14	0.79 (0.74–0.84)	3.48E-15	0.79	0.55	0.00
17	19748589	rs4646785	T	8.88E-01	1.02 (0.74–1.41)	9.01E-12	0.82 (0.78–0.87)	1.38E-11	0.83	0.18	44.40
17	31558198	rs56161228	A	4.48E-02	0.64 (0.41–0.99)	2.70E-10	0.81 (0.76–0.86)	5.58E-11	0.81	0.29	9.63
17	48518906	rs7225995	C	2.26E-02	1.44 (1.05–1.96)	6.63E-09	1.19 (1.12–1.26)	8.34E-10	1.20	0.24	26.24
17	50167170	rs12939159	C	8.87E-01	0.97 (0.64–1.47)	2.11E-08	0.82 (0.77–0.88)	1.08E-08	0.82	0.43	0.00
20	2095648	rs6106210	T	5.01E-01	1.1 (0.83–1.46)	2.85E-11	1.19 (1.13–1.25)	1.42E-11	1.19	0.60	0.00
21	28247396	rs118737	T	5.56E-01	1.1 (0.81–1.49)	1.11E-10	1.22 (1.15–1.3)	1.70E-10	1.22	0.51	0.00
22	20969069	rs756878	T	4.76E-02	1.31 (1–1.72)	4.01E-09	1.17 (1.11–1.24)	1.15E-09	1.18	0.41	0.00

Firm novel genetic associations meeting established criteria are highlighted in bold. bp, base pair; CHR, chromosome; GRCh38, genome reference consortium human build 38; *I*^2^, measure of heterogeneity; OR, per-allele odds ratio for the reference allele; *Q*, Cochran’s *Q* test value; Ref, reference allele.

Five additional signals surpassed the genome-wide significance threshold in the meta-analysis (*P* < 5E-08) but their P values in the Spanish cohort were >0.05 (Table). Additionally, 25 out of the 36 KC-associated loci described in the original study by Hardcastle et al.^16^ had improved P values in our meta-analysis, as 14 of them were associated at the nominal level in the Spanish cohort (Supplementary Fig. S2, Supplementary Table S1).

### Polygenic Risk Score Analysis

**Figure 2.**
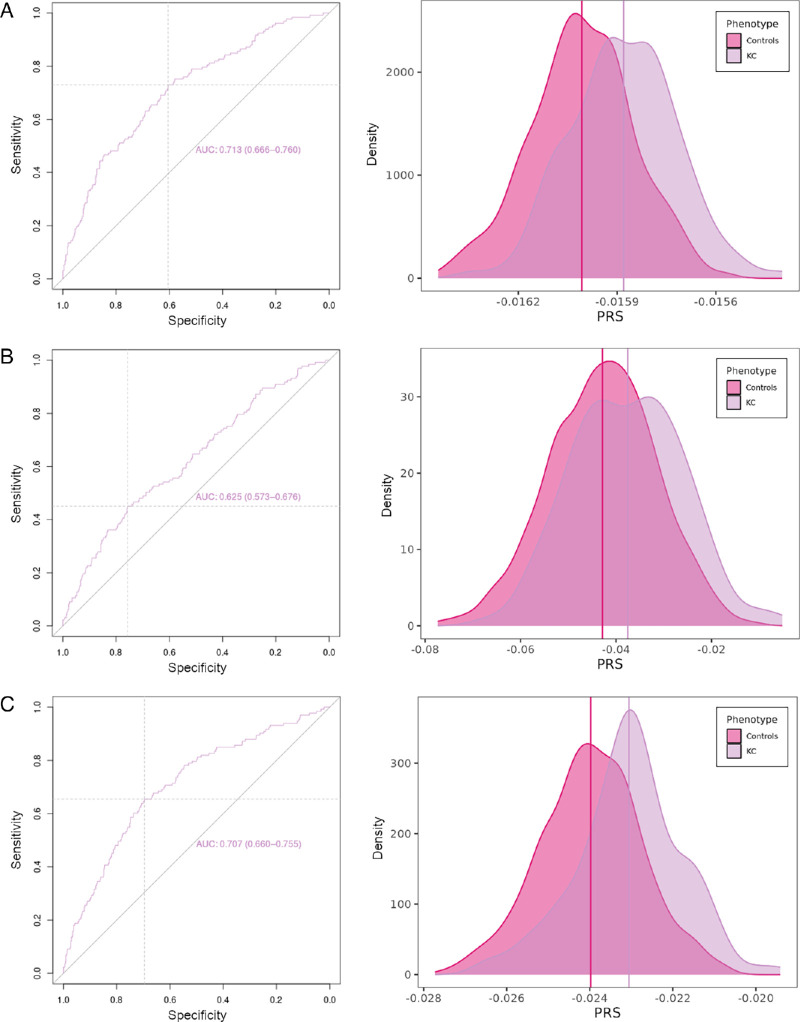
PRS models assessed in this study. (**A**–**C**) Receiver operating characteristic (ROC) curves (*left*) and distribution of PRS values for KC cases and controls in the Spanish testing cohort (*right*) are shown for the models considering KC data from Hardcastle et al.^16^ (**A**), CCT data from Choquet et al.^15^ (**B**), and KC and CCT combined data from both studies (**C**).

To evaluate the predictive ability of the GWAS results reported by Hardcastle et al.^16^ in identifying individuals at increased risk of developing KC, we calculated PRSs for each individual in our Spanish cohort by combining the weighted allelic effects described in their summary statistics. The best-fitting PRS model (PRS-*R*^2^ = 0.126; *P* = 7.61E-16; permutation *P* = 9.99E-04) was based on 34,832 SNPs with *P* < 3.98E-02 (Supplementary Fig. S3A). This PRS model effectively distinguished KC patients from controls (area under the curve [AUC] = 0.713, 95% confidence interval [CI], 0.666–0.760) (Fig. 2), with the distribution of PRS values in KC cases and controls being significantly different (control mean = −1.60E-02 vs. KC mean = −1.59E-02; *P* = 4.61E-15, *t*-test). The predictive capability of the PRSs derived solely from genome-wide significant signals (i.e., SNPs with *P* < 5E-08) was less efficient compared to that obtained when using the SNPs of the best-fitting model (PRS-*R*^2^ = 0.070) (Supplementary Fig. S3A).

Alternatively, to evaluate whether CCT data were equally valid in distinguishing between KC and controls in the Spanish texting set, we calculated additional PRSs based on the genomic information described in the most recent CCT GWAS.^15^ In this case, the best-fitting model (PRS-*R*^2^ = 0.045; *P* = 6.82E-07; permutation *P* = 9.99E-04) included a total of 1521 SNPs with *P* < 8.80E-04 (Supplementary Fig. S3B). Interestingly, this PRS model was also efficient in separating the distributions of PRS values between KC cases and controls (control mean = −4.28E-02 vs. KC mean = −3.76E-02; *P* = 1.19E-06, *t*-test; AUC = 0.625; 95% CI, 0.573–0.676) (Fig. 2B). Again, the PRSs calculated using signals reaching genome-wide statistical significance showed a lower predictive capability (PRS-*R*^2^ = 0.023) compared to the best-fitting PRS model (Supplementary Fig. S3B).

Finally, we sought to assess whether combining KC and CCT data would enhance the ability to discriminate between KC cases and controls compared with considering each trait independently. This time, the PRSs were calculated utilizing the results of the meta-analysis of the genetic variants associated with both KC and CCT.^15^^,^^16^ The best-fitting model (PRS-*R*^2^ = 0.108; *P* = 9.75E-14; permutation *P* = 9.99E-04) included a total of 1005 SNPs with *P* < 2.50E-04 (Supplementary Fig. S3C). As in the previous tests, this model allowed an adequate discrimination between KC cases and controls (AUC = 0.707; 95% CI, 0.660–0.755), with an evident distinction in the distribution of PRS values between the two groups (control mean = −2.40E-02 vs. KC mean = −2.31E-02; *P* = 3.95E-13, *t*-test) (Fig. 2C).

### Functional Annotation and Gene Prioritization of Associated Signals

In an attempt to identify potential genes involved in the development of the KC condition, we performed a SNP2GENE analysis using FUMA.^21^ This approach led to the identification of 119 independent significant SNPs (is-SNPs), defined as SNPs with a *P* < 5E-08 and demonstrating mutual independence (*r*^2^ < 0.6). Subsequently, 4001 candidate SNPs were identified as those with *r*^2^ ≥ 0.6 with is-SNPs, collectively tagging a total of 315 genes. Additionally, 51 independent lead SNPs (il-SNPs) were determined, corresponding to those is-SNPs with *r*^2^ < 0.1 among themselves. When considering the merging of loci within a 250-kB range, a total of 41 genomic risk loci were defined (Supplementary Fig. S4).

Then, we conducted a MAGMA tissue expression analysis to gain a thorough understanding of the correlation between tissue-specific expression profiles and all KC-associated variants. Based on the information extracted from the GTEx v8 project,^24^ we found no significant impact of KC-associated variants on gene expression profiles in 30 general organs and tissues, although a clear trend of association was observed in the skin. Conversely, when 53 specific structure types were considered, we observed that KC-associated variants were significantly linked to genes expressed in cultured fibroblasts (Supplementary Fig. S5).

Furthermore, we observed that the above-mentioned 315 genes selected by FUMA were enriched not only in KC-associated genes but also in genes relevant for other eye phenotypes, such as intraocular pressure (IOP), CCT, corneal structure, refractive error, CH, CRF, and Fuch's corneal dystrophy (Supplementary Fig. S6).

**Figure 3.**
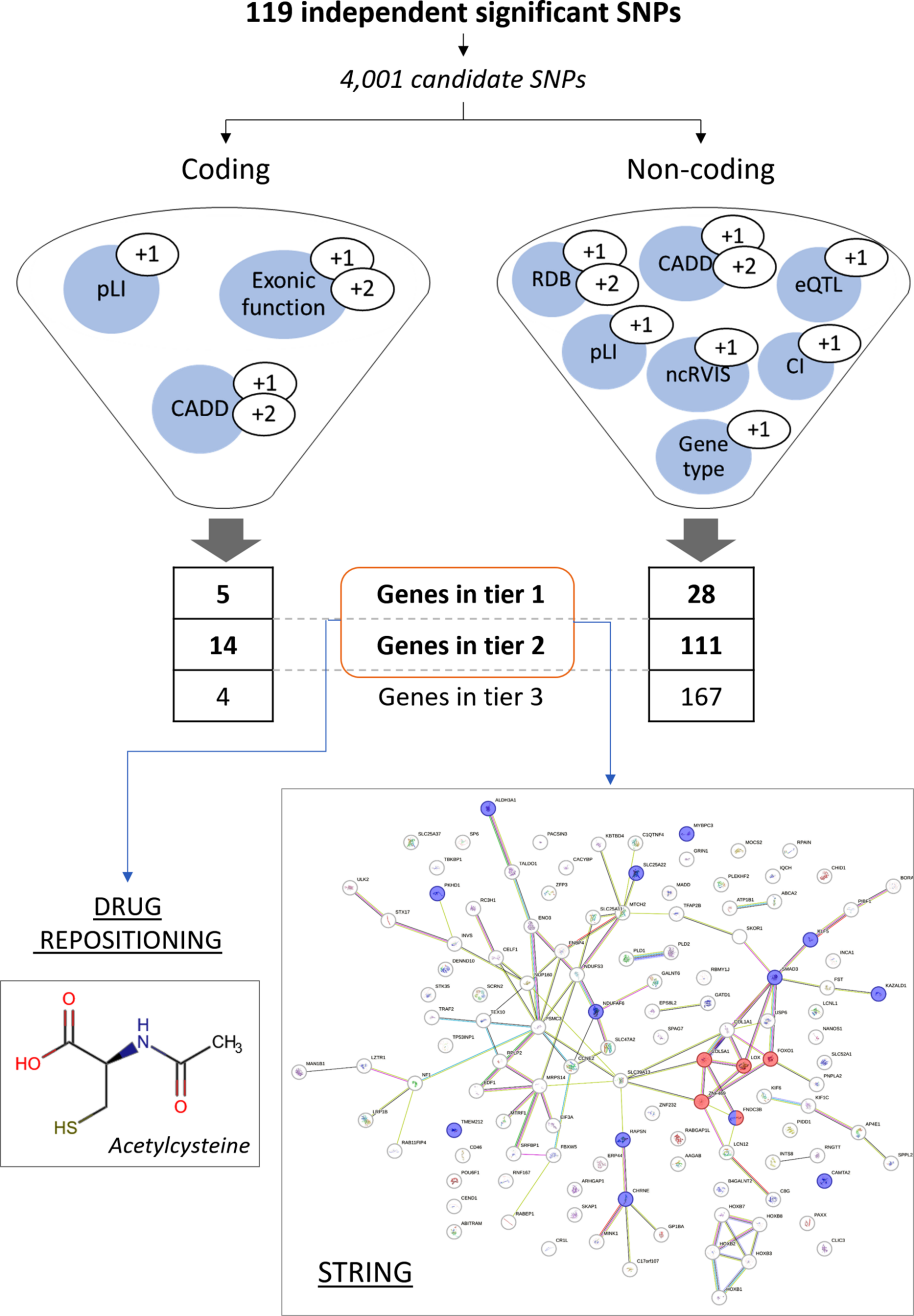
Schematic representation of the computational process for functionally annotating and prioritizing KC-associated signals. The meta-analysis revealed 119 independent SNP associations with KC (*P* < 5E-08, *r*^2^ < 0.6). A total of 4001 candidate SNPs were considered due to their linkage with the initial 119 variants (*r*^2^ ≥ 0.6). The candidate SNPs were further categorized based on their impact on genes, distinguishing between coding and non-coding effects. Each gene affected by these SNPs received a score according to the following criteria: (1) For genes influenced by SNPs with coding effects, points were assigned as follows—1 point for a probability of loss-of-function intolerance (pLI) ≥ 0.9, 1 point for any associated SNP with a deleteriousness score (Combined Annotation Dependent Depletion [CADD]) ≥ 10, and 2 points for CADD ≥ 20; also, depending on the type of SNP effect, 1 point for synonymous or non-reading frame-changing mutations and 2 points for non-synonymous or reading frame-changing mutations. (2) For genes influenced by SNPs with non-coding effects, points were allocated based on CADD and pLI values as before—1 point for a non-coding residual variation intolerance score (ncRVIS) < 0, 1 point if the gene was protein coding, 1 point if there was an expression quantitative trait locus (eQTL) effect, 1 point if the associated SNP had a RegulomeDB (RDB) score of 3/4 and 2 points for scores of 1/2, and 1 point if the gene was mapped by chromatin interaction (CI) mapping. According to the scores, the genes were classified into three tiers: tier 1, score 6 to 9 for non-coding effects and 4 or 5 for coding effects; tier 2, score 3 to 5 for non-coding effects and 2 or 3 for coding effects; and tier 3, score 0 to 2 for non-coding effects and 0 or 1 for coding effects. Subsequently, genes in tiers 1 and 2 underwent PPI and biological pathway enrichment analyses using STRING and were subjected to drug repositioning analysis. In the STRING-derived image, confirmed genes associated with KC are depicted in *red*, and those associated with intraocular pressure measurement are shown in *blue*.

To gain a better understanding of the primary molecular pathways contributing to KC development and progression, we decided to conduct a parallel prioritization analysis by assessing variants affecting coding regions separately from those located in non-coding regions. Consequently, different parameters were considered for gene prioritization depending on the distinct effects of the variants by which each gene is influenced, as summarized in Figure 3. The results of both approaches are detailed in Supplementary Tables S2 and S3. A scoring system reflective of the type and magnitude of the impact of each feature was applied, leading to the classification of the two groups of genes into three distinct tiers (Fig. 3, Supplementary Table S4). Among all of the prioritized genes, *MINK1* stood out as the only gene classified in tier 1 within both groups. Notably, most of the tier 1 genes showed potential involvement in KC pathogenesis (Supplementary Table S5).

Finally, we selected all genes included in tier 1 and tier 2 of both groups and performed a protein–protein interaction (PPI) and biological pathway enrichment analysis of their encoded proteins (Fig. 3). The protein molecular network exhibited significantly more interactions than expected (number of nodes = 120; number of edges = 98; average node degree = 1.63; average local clustering coefficient = 0.37; expected number of edges = 47; PPI enrichment = 8.42E-11) (Fig. 3). The functional enrichment of the network showed associations with various human phenotypes, with IOP measurement showing the most significant *P* (*P* = 1.28E-05). Notably, KC (*P* = 0.001), corneal disease (*P* = 0.039), and Ehlers–Danlos syndrome (*P* = 0.039) were the associated diseases correlated with this network. Indeed, some genes associated with KC disease (highlighted in red in Fig. 3) and with IOP measurement (highlighted in blue in Fig. 3) represented relevant nodes in the PPI network.

### Drug Repositioning

Drug repositioning assessment was undertaken to explore novel therapeutic avenues for KC disease. Specifically, genes classified in tiers 1 and 2 were analyzed as drug targets (Fig. 3). Through a comprehensive screening of pharmaceutical compounds within the DrugBank database, our investigation revealed that a collective total of 60 drugs documented in this repository target genes from both tier 1 and tier 2, with 38 of them being approved (Supplementary Table S6). One particularly notable medication in this category is acetylcysteine, which is prescribed for treating keratopathies, corneal diseases, and corneal ulceration.^23^

## Discussion

In the midst of an ongoing debate about the role of genetic factors in KC versus its classification as a purely environmental disease,^4^^,^^25^ we present evidence for seven new putative genetic associations with KC. Additionally, we identified two more KC-associated signals by demonstrating that two loci identified by Hardcastle et al.^16^ actually represent four independent associations. This discrepancy occurred because Hardcastle et al.^16^ determined independence based on physical distance rather than linkage disequilibrium patterns, which are commonly used.^26^

To our knowledge, this study includes the first comprehensive in silico exploration of the molecular mechanisms underlying KC, marking a significant advancement in our understanding of this disease. A total of 315 genes influenced by 4001 candidate SNPs for KC predisposition were revealed. This geneset exhibited enrichment in associations with various parameters concerning corneal structure and biomechanical properties, including CH, CRF, and CCT, which serve as critical indicators of corneal viscosity and its ability to withstand mechanical stress.^27^ KC patients often exhibit reduced values for them,^28^^,^^29^ indicative of a higher vulnerability of their corneas to damage from various mechanical forces. Additionally, we found connections with Fuchs’ corneal dystrophy, a condition hypothesized to share common etiological factors with KC, such as elevated levels of oxidative stress in the cornea.^30^

Our prioritization analysis, employing tier categorization, pinpointed *MINK1* as a pivotal gene in KC pathogenesis. *MINK1* encodes a serine/threonine kinase belonging to the germinal center kinase family, which plays a role in crucial molecular pathways, including JNK, p38, and WNT signaling.^31^^,^^32^ Regarding the latter, MINK1 phosphorylates PRICKLE1, a relevant component in planar cell polarity (PCP) signaling during mouse eyelid development.^32^^,^^33^ Strikingly, *Prickle1* mutant mice exhibited corneal malformations post-eyelid opening, characterized by thickened corneal epithelium, the presence of corneal lesions covered by tissue debris, alterations in corneal cell fate, and an expression profile resembling that of various dermatological diseases.^33^^,^^34^ These findings suggest that the association of *MINK1* with KC may rely on alterations within the WNT pathway, potentially influencing PCP establishment and maintenance. Indeed, experimental knockdown of *VANGL2* (a key PCP signaling component) within the corneal epithelium of adult mice resulted in attenuated cell migration during wound healing, aberrations in tissue stratification, and partial depletion of the epithelial basement membrane.^35^^,^^36^ The involvement of WNT signaling in KC pathogenesis, along with alterations in its components or their association with the disease, has been previously reported in multiple studies.^37^^,^^38^

We observed a higher than expected number of interactions within the PPI network formed among the encoded proteins of tier 1 and tier 2 genes, which showed enrichment in associated phenotypes such as IOP, corneal disease, and Ehlers–Danlos syndrome, a frequent comorbidity in KC patients.^39^ Specifically, genes associated with both KC and IOP represented significant nodes in this PPI network. These findings are consistent with the omnigenic model proposed for complex diseases, wherein a set of genes, denoted as core genes, holds particular functional relevance in the pathogenesis of the disease.^40^^,^^41^ Hence, our results suggest that tier 1 and 2 genesets, characterized by the strongest evidence of functional impact, likely include core genes contributing to KC development. Indeed, previous studies demonstrated that the key genes identified in our analysis align with established molecular and cellular mechanisms underlying KC pathology (Supplementary Table S5).

In light of the findings from our study, we decided to explore their potential translational applicability in clinical settings by developing a PRS model and evaluating alternative therapeutic interventions. In this sense, acetylcysteine emerged as a potential novel treatment for KC following an analysis of drugs targeting all genes classified in tiers 1 and 2. It is worth noting that this molecule has previously exhibited efficacy in managing various ocular pathologies, including keratoconjunctivitis sicca, dry eye disease, refractory filamentary keratitis, corneal mucous plaques, and alkali-burned cornea.^42^^–^^46^ Furthermore, extensive research supports its effectiveness in mitigating corneal damage. For example, acetylcysteine has been found to reduce the healing time of corneal wounds in dogs and rabbits,^47^^,^^48^ enhance the cellular survival of corneal endothelium in a murine models of Fuchs endothelial corneal dystrophy,^49^ and improve the integrity of corneal epithelium in diabetic mouse models.^50^ Further studies are warranted to thoroughly evaluate the therapeutic potential of acetylcysteine in the management of KC.

Regarding the PRS approach, our results revealed that the data derived from the KC GWAS by Hardcastle et al.^16^ and those from the CCT GWAS by Choquet et al.^15^ had a good predictive capability for identifying individuals at high risk for KC. Similar observations regarding PRS efficacy have been reported across various ocular disorders.^51^^–^^53^ The utility of PRSs is of particular interest in the early stages of diseases, where they can facilitate diagnosis and open avenues for exploring potential treatment options.^9^ This is especially important in KC, where delayed diagnosis may lead to corneal transplantation, an option often economically prohibitive for many affected individuals.^54^ Early KC detection allows for collagen cross-linking treatment and intrastromal ring segments implantation,^55^^–^^57^ capable of stabilizing or even reversing disease progression. Such measures could prevent post-laser in situ keratomileusis (LASIK) ectasia, given the prevalent occurrence of undiagnosed early KC among individuals subsequently manifesting this condition.^58^

Moreover, integrating our proposed PRS model with data from recently identified molecular biomarkers for KC, such as circular RNAs and microRNAs,^59^^,^^60^ could significantly enhance its diagnostic and prognostic capabilities. However, employing 1005 SNPs for calculating PRSs may present challenges for clinical implementation due to the costs and complexities of large-scale genotyping. That said, integrating PRSs into risk screening has already demonstrated success in improving the detection of high-risk individuals in other complex diseases, leading to lower long-term healthcare costs and better outcomes.^61^^,^^62^ Furthermore, recent advancements in biochip technologies and genotyping assays^63^ have significantly reduced costs, making PRS strategies more feasible, cost effective, and clinically viable for KC in the near future.

In summary, our investigation provides valuable insights into the genetic basis of KC by establishing two previously unknown genetic associations (and suggesting five additional markers), replicating 33 reported signals, and evaluating the utility of different PRS models as predictive tools. Our extensive computational analysis not only has elucidated major molecular mechanisms underlying KC but has also pinpointed potential core genes pivotal in its pathogenesis. Moreover, our study underscores the clinical potential of acetylcysteine as an emerging therapeutic option for KC. Nevertheless, further research is needed to validate our findings and ascertain the efficacy of the suggested clinical intervention.

## Supplementary Material

Supplement 1

Supplement 2

Supplement 3
