# Supplementary material for: Comprehensive Evaluation of the Genetic Basis of Keratoconus: New Perspectives for Clinical Translation
Source: Invest Ophthalmol Vis Sci. 2024 Oct 22;65(12):32. doi: 10.1167/iovs.65.12.32 (PMC11500050; doi:10.1167/iovs.65.12.32)

SUPPLEMENTARY FIGURES

Comprehensive evaluation of the genetic basis of keratoconus: new perspectives for clinical translation

Miriam Cerván-Martín, Inmaculada Higueras-Serrano, Sara González-Muñoz, Andrea Guzmán-Jiménez, Blas Chaves-Urbano, Rogelio J. Palomino-Morales, Arancha Poo-López, Luis Fernández-Vega Cueto, Jesús Merayo-Lloves, Ignacio Alcalde, Lara Bossini-Castillo, F. David Carmona

Figure S1. Plot of the first and second principal components of the case-control Spanish cohort analysed in this study. Cases are represented by circles and controls are represented by squares.

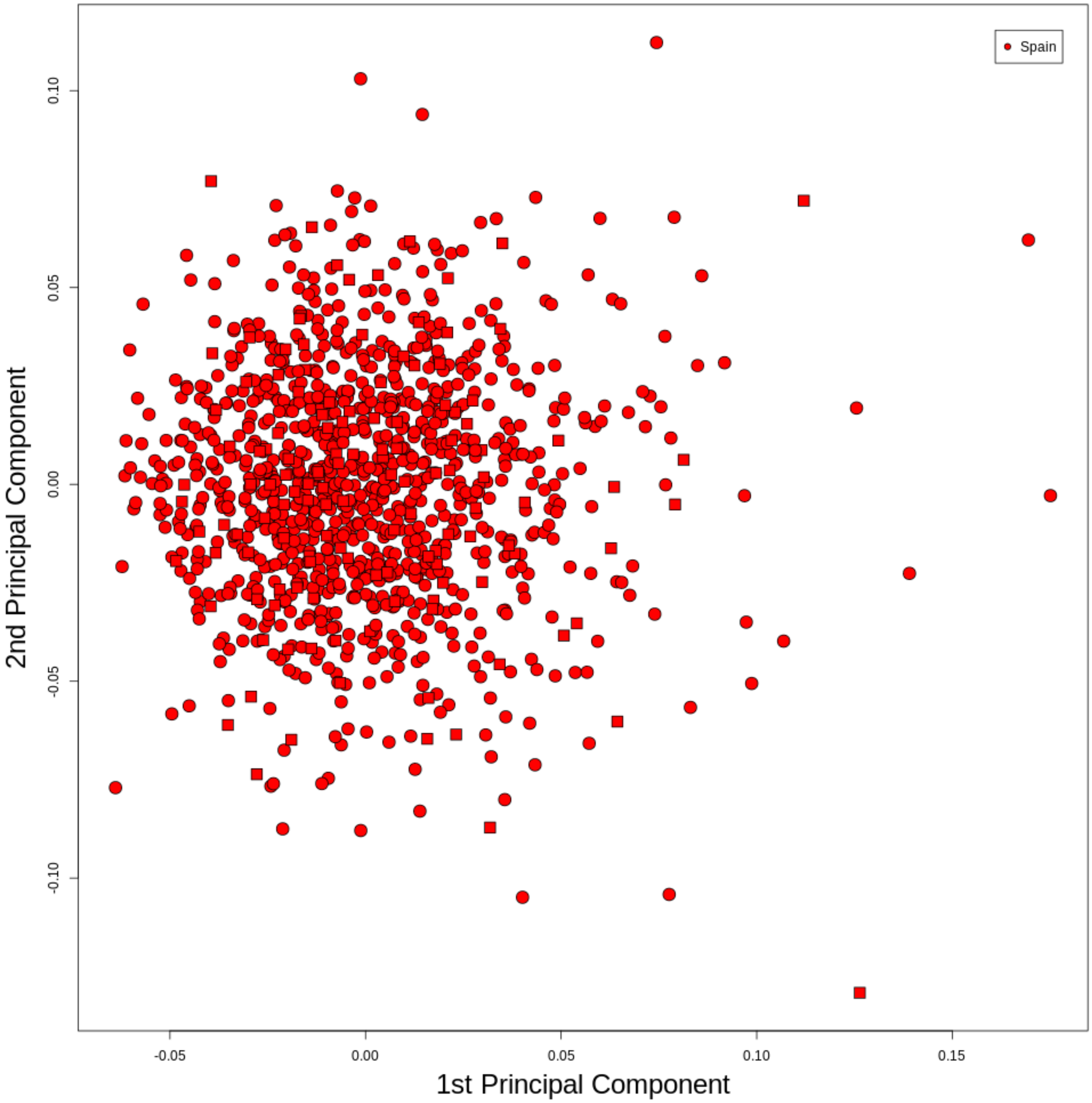

Figure S2. Manhattan plot of the meta-analysis results. The −log10 of the SNPs P-values are plotted against its physical chromosomal position. The red line represents the genome-wide significance threshold (P < 5E-08).

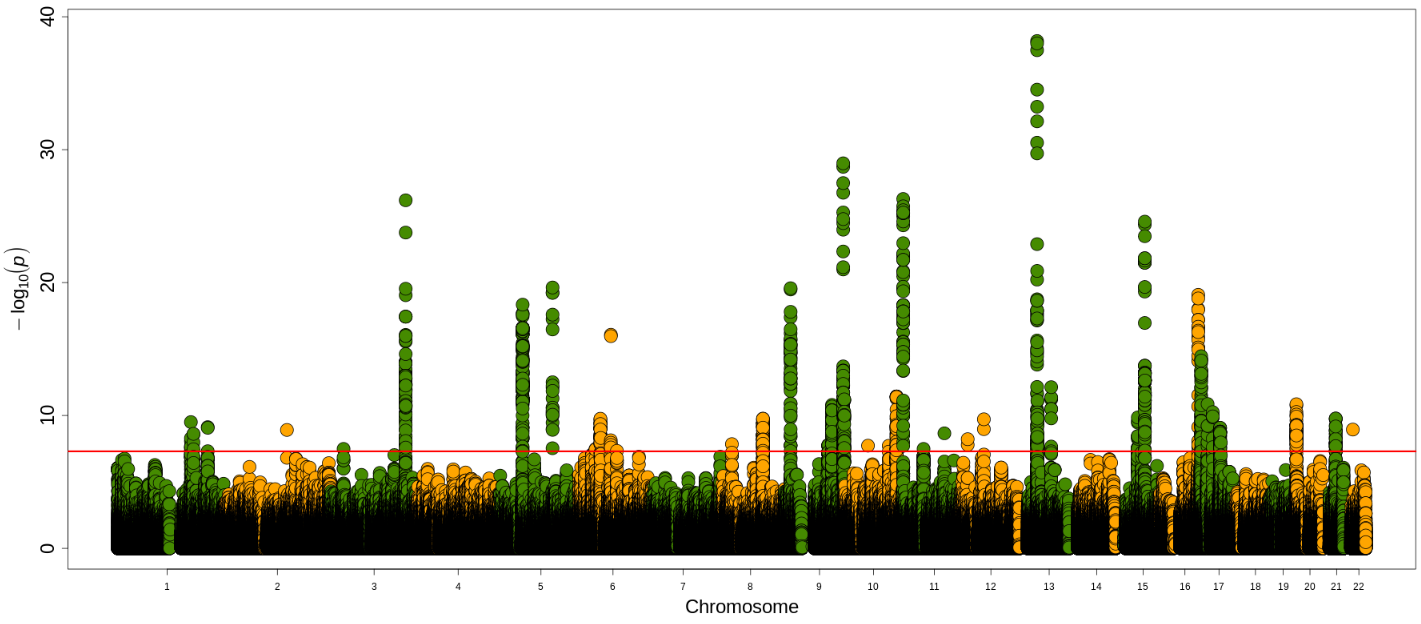

Figure S3. Identification of the best-fitting polygenic risk score for keratoconus (KC). The weighted allelic effects for calculating the scores were based on the results from **a)** the KC GWAS by Hardcastle *et al*. [14] alone, **b)** the central corneal thickness (CCT) GWAS by Choquet *et al*. [13] alone, and **c)** both studies combined. Graphs on the left depict, on the left Y-axis, the number of single-nucleotide polymorphisms (SNPs) corresponding to the p-value thresholds applied to the data from each study. On the right Y-axis, the model fit (R^2) is presented, and on the X-axis, all tested p-value thresholds. A comparison of the best-fitting model with the model calculated using only signals reaching genome-wide significance levels is also shown.

a)

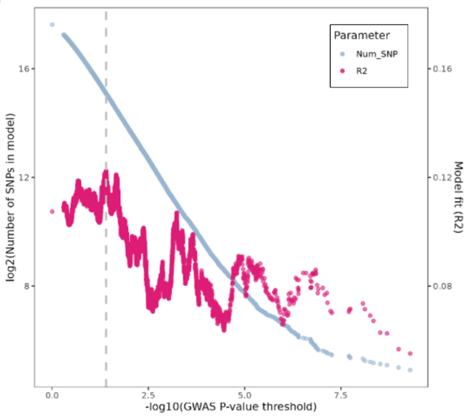

	Best fit model	GWAS signals model
**Phenotype**	KC	KC
**Threshold**	3.98E-02	1.95E-08
**PRS (R2)**	0.126	0.070
**Full (R2)**	0.148	NA
**Null (R2)**	0.026	NA
**Coefficient**	5272.040	58.725
**Standard Error**	654.073	9.337
**P-value**	7.61E-16	3.19E-10
**Nº SNPs**	34,832	43
**Empirical P-value**	9.99E-04	NA

b)

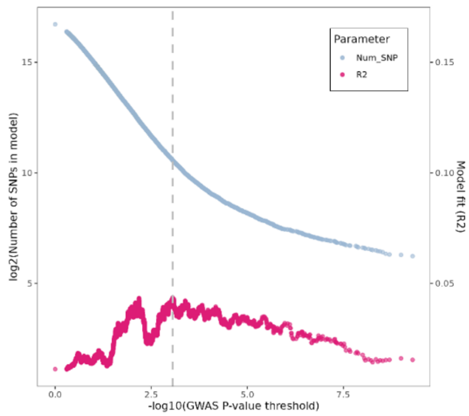

	Best fit model	GWAS signals model
**Phenotype**	CCT	CCT
**Threshold**	8.80E-04	5.00E-08
**PRS (R2)**	0.045	0.023
**Full (R2)**	0.069	NA
**Null (R2)**	0.026	NA
**Coefficient**	43.806	4.851
**Standard Error**	8.820	1.318
**P-value**	6.82E-07	2.32E-04
**Nº SNPs**	1,521	117
**Empirical P-value**	9.99E-04	NA

c)

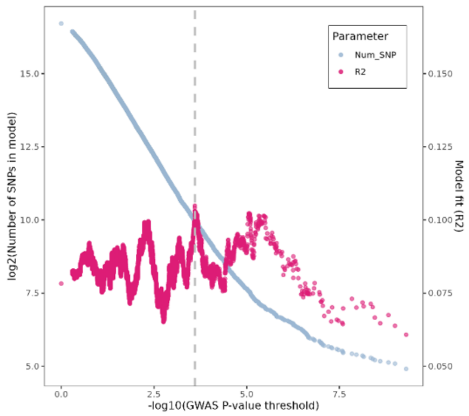

	Best fit model	GWAS signals model
**Phenotype**	KC + CCT	KC + CCT
**Threshold**	2.50E-04	4.95E-08
**PRS (R2)**	0.108	0.070
**Full (R2)**	0.131	NA
**Null (R2)**	0.026	NA
**Coefficient**	631.913	61.849
**Standard Error**	84.886	9.938
**P-value**	9.75E-14	4.86E-10
**Nº SNPs**	1,005	49
**Empirical P-value**	9.99E-04	NA

Figure S4. Results of the SNP2GENE analysis using FUMA. **a)** Summary results per genomic risk *locus*. The bars illustrate the size of each genomic risk *locus* along with the count of candidate SNPs, mapped genes, and physically located genes within each *locus*. **b)** Functional impacts of keratoconus-associated candidate SNPs on affected genes. The bars represent the percentage of candidate SNPs with each functional annotation. Log2 (enrichment) relative to all SNPs in the selected reference panel is represented by a red-blue colour gradient. Enrichment is computed as the ratio of the proportion of candidate SNPs with a specific annotation to the proportion of candidate SNPs with that annotation relative to all available SNPs in the reference panel. Fisher's exact test (two-sided) was conducted for each annotation. *p < 0.05; **p < 0.05/11.

a)

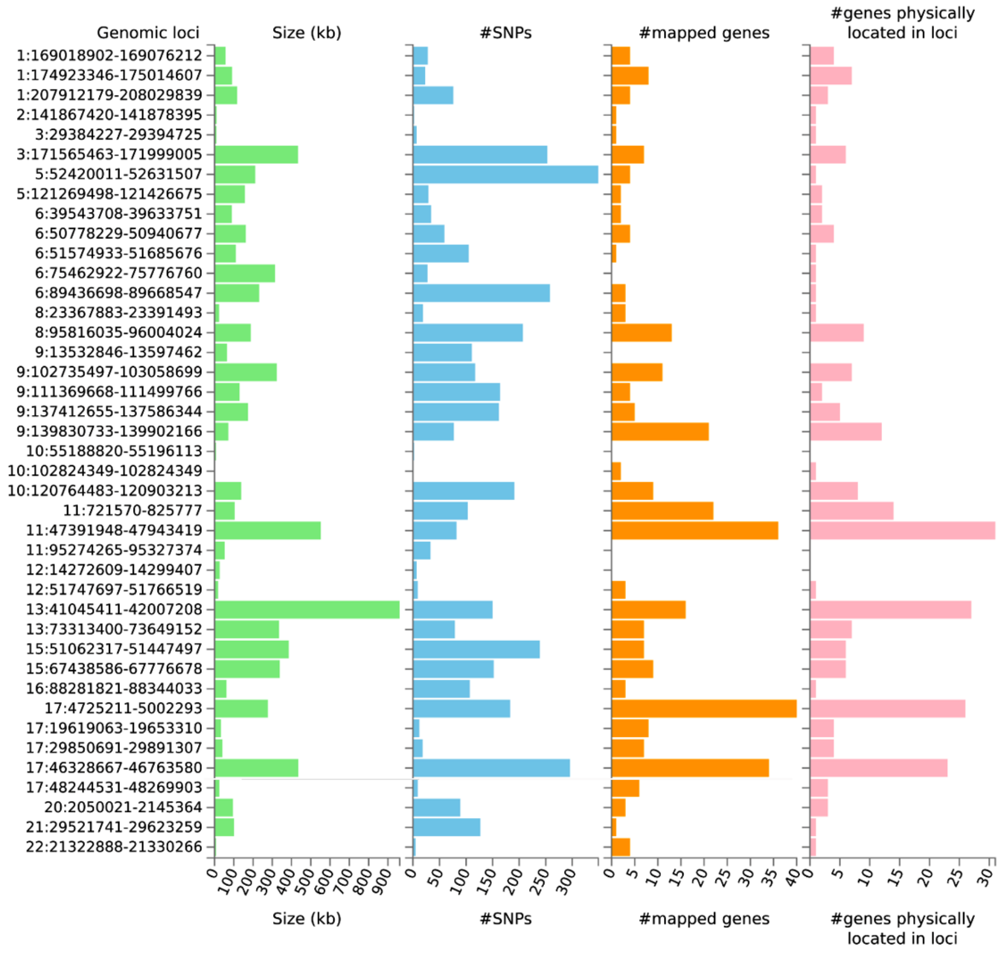

b)

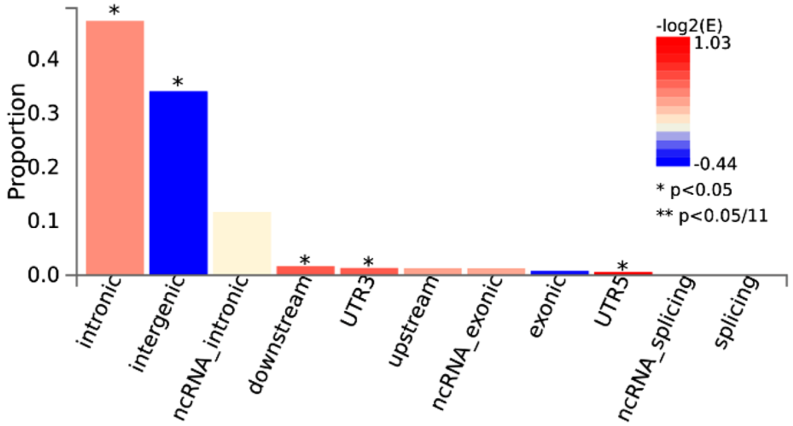

Figure S5. Bar charts illustrating the MAGMA tissue expression analysis results using GTEx v8 data. **a)** Analysis considering 30 general organs and tissues. **b)** Analysis considering 53 specific structure types. The horizontal line denotes the threshold for statistical significance. The colour of the bars indicates whether statistical significance was achieved (red) or not (blue).

a)

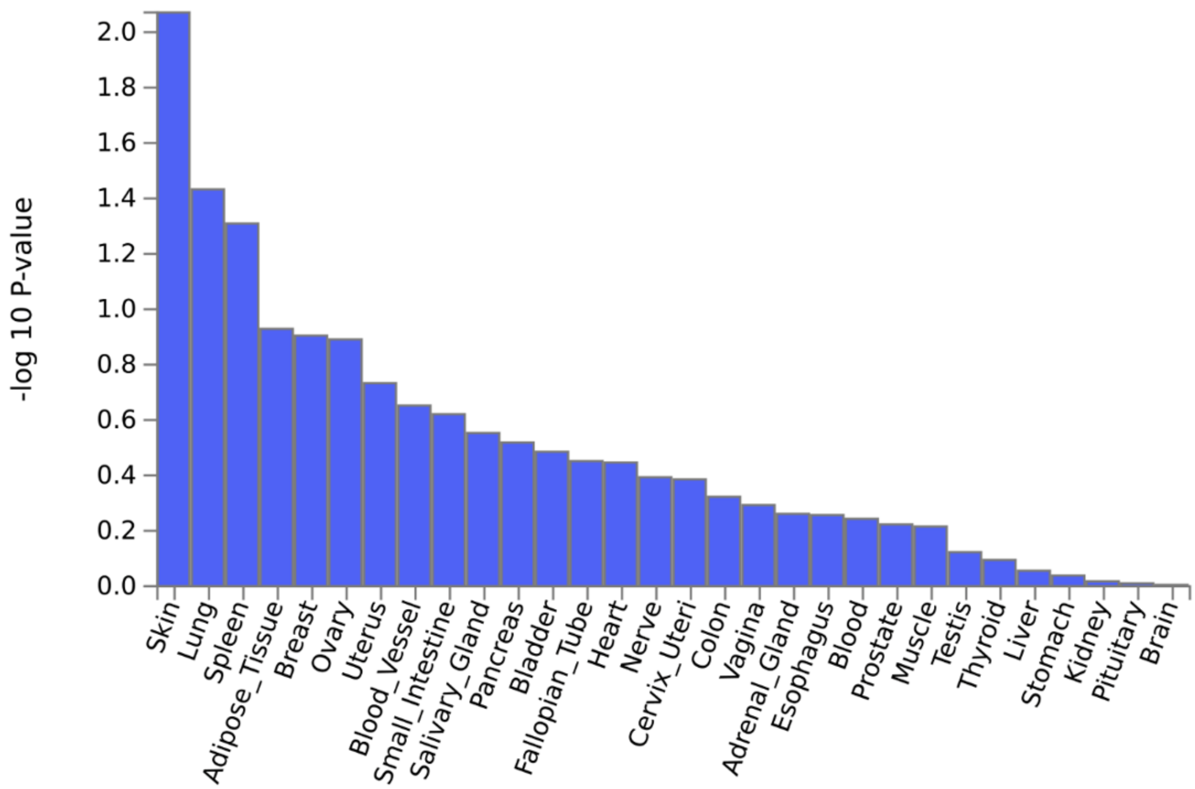

b)

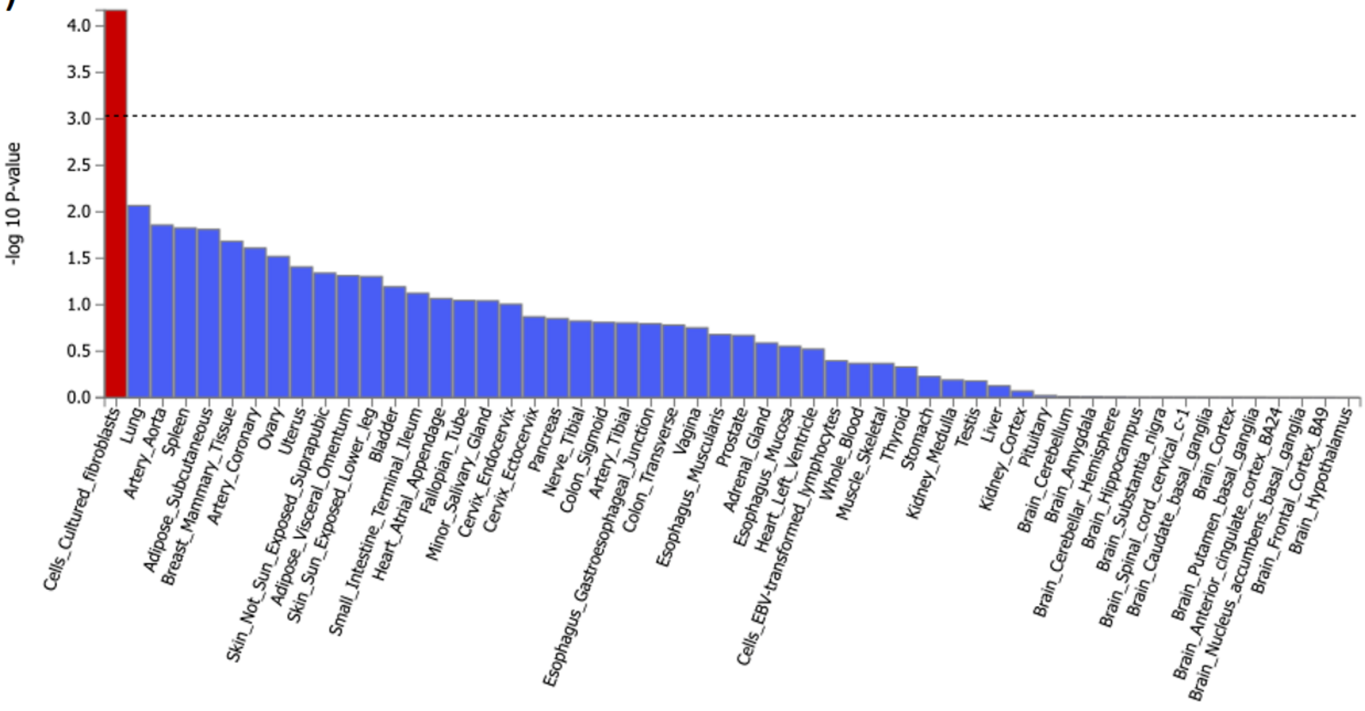

Figure S6. Enrichment analysis of genes associated with phenotypes included in the GWAS catalog considering the 315 prioritised keratoconus-linked genes according to FUMA.

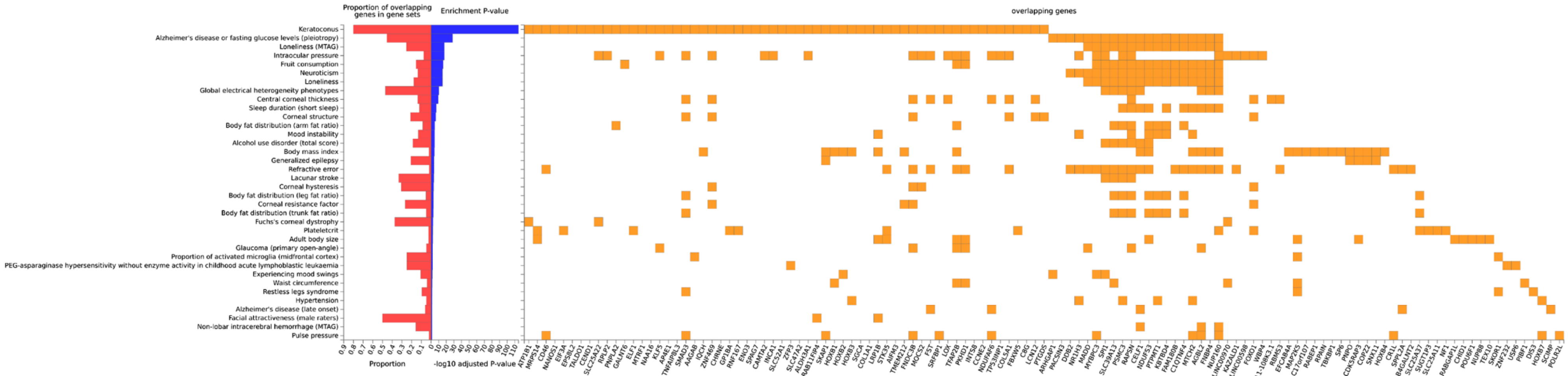

Supplement: Supplement 1 [file iovs-65-12-32_s001.pdf]
