# Supplementary material for: Comprehensive Evaluation of the Genetic Basis of Keratoconus: New Perspectives for Clinical Translation
Source: Invest Ophthalmol Vis Sci. 2024 Oct 22;65(12):32. doi: 10.1167/iovs.65.12.32 (PMC11500050; doi:10.1167/iovs.65.12.32)
Supplement: Supplement 2 [file iovs-65-12-32_s002.pdf]

SUPPLEMENTARY TABLES

Comprehensive evaluation of the genetic basis of keratoconus: new perspectives for clinical translation

Miriam Cerván-Martín, Inmaculada Higueras-Serrano, Sara González-Muñoz, Andrea Guzmán-Jiménez, Blas Chaves-Urbano, Rogelio J. Palomino-Morales, Arancha Poo-López, Luis Fernández-Vega Cueto, Jesús Merayo-Lloves, Ignacio Alcalde, Lara Bossini-Castillo, F. David Carmona

Table S1. Genetic variants associated with keratoconus susceptibility at the genome-wide significance level (P<5x10-8) in the meta-analysis. The results of the different independent studies are also shown. CHR, chromosome; BP, base pair; Ref: Reference Allele; OR, per-allele odds ratio for the minor allele; L95: lower limit of the 95% confidence interval; U95, upper limit of the 95% confidence interval; Q, Cochran's Q test; I: I^2.

				Spanish Cohort (N = 1,116)				Hardcastle Cohort (N = 121,216)				META-ANALYSIS					
CHR	BP (GRCh38)	Variant ID	Ref	P-value	OR	L95	U95	P-value	OR	L95	U95	P-value	OR	P-value (R)	OR (R)	Q	I
1	169049664	rs3007364	A	0.7966	0.9654	0.7388	1.262	3.30E-08	0.85	0.8	0.9	4.81E-08	0.8548	4.81E-08	0.8548	0.3619	0
1	169056567	rs3007369	A	0.6317	1.068	0.8172	1.395	1.05E-08	1.18	1.12	1.25	2.08E-08	1.1748	2.08E-08	1.1748	0.4748	0
1	169059478	rs2982459	A	0.7351	1.047	0.8009	1.37	3.14E-08	1.18	1.11	1.25	2.77E-08	1.1738	2.77E-08	1.1738	0.3931	0
1	169060293	rs2982461	T	0.7351	0.9547	0.73	1.249	3.30E-08	0.85	0.8	0.9	4.85E-08	0.8544	4.85E-08	0.8544	0.4068	0
1	169063587	rs3007370	T	0.7351	1.047	0.8009	1.37	3.27E-08	1.18	1.11	1.25	2.77E-08	1.1738	2.77E-08	1.1738	0.3931	0
1	169083679	rs1200103	T	0.7942	0.965	0.7385	1.261	7.62E-09	0.85	0.81	0.9	8.45E-09	0.8543	8.45E-09	0.8543	0.3624	0
1	169088731	rs1200108	A	0.2882	0.8666	0.6654	1.129	4.52E-10	0.85	0.81	0.89	3.14E-10	0.8506	3.14E-10	0.8506	0.888	0
1	169099332	rs1200122	T	0.6282	1.069	0.8157	1.401	6.48E-09	1.17	1.11	1.23	5.12E-09	1.1662	5.12E-09	1.1662	0.5207	0
1	169103698	rs932393	C	0.5646	1.083	0.8265	1.418	5.21E-08	1.17	1.1	1.23	2.27E-08	1.1664	2.27E-08	1.1664	0.5825	0
1	175021769	rs7514888	T	0.7605	1.046	0.7853	1.392	4.70E-09	1.17	1.11	1.23	2.64E-09	1.1659	2.64E-09	1.1659	0.4501	0
1	175022798	rs2105949	A	0.7605	0.9565	0.7185	1.273	3.18E-09	0.86	0.81	0.9	1.07E-08	0.8629	1.07E-08	0.8629	0.4734	0
1	175024486	rs6669560	T	0.7003	1.058	0.7942	1.409	2.92E-09	1.17	1.11	1.23	2.42E-09	1.1663	2.42E-09	1.1663	0.4984	0
1	175040874	rs997985	A	0.6835	1.056	0.814	1.369	4.75E-08	0.87	0.83	0.92	3.49E-08	0.8756	0.3183	0.9174	0.1508	51.56
1	207801473	rs2724377	A	0.02576	0.7424	0.5714	0.9646	3.10E-07	0.88	0.84	0.92	4.89E-08	0.875	0.01752	0.8503	0.2109	36.12
1	207808074	rs761276	A	0.02466	0.74	0.5691	0.9623	8.02E-09	0.87	0.83	0.91	7.55E-10	0.8657	0.005946	0.8467	0.2344	29.29
1	207848946	rs1204707	T	0.0232	0.7383	0.5681	0.9594	1.49E-08	0.87	0.83	0.92	8.50E-10	0.8656	0.007351	0.8448	0.2268	31.53
2	141120826	rs116792882	T	0.09699	5.471	0.7352	40.72	4.82E-10	0.5	0.41	0.62	1.23E-09	0.5139	0.8076	1.3318	0.0202	81.47
3	29352153	rs11129361	A	0.06268	1.3	0.9862	1.714	2.19E-07	1.15	1.09	1.21	3.26E-08	1.1548	3.26E-08	1.1548	0.3931	0
3	171855649	rs35398158	T	0.07122	1.369	0.9732	1.925	2.12E-13	1.25	1.18	1.33	5.56E-14	1.2534	5.56E-14	1.2534	0.6067	0
3	171857360	rs57403442	A	0.07478	0.7331	0.521	1.032	3.94E-13	0.8	0.75	0.85	3.15E-13	0.7978	3.15E-13	0.7978	0.622	0
3	171858646	rs35240776	T	0.07122	0.7305	0.5194	1.028	6.49E-13	0.79	0.75	0.85	3.70E-14	0.788	3.70E-14	0.788	0.6581	0
3	171862275	rs35615803	A	0.08523	0.7434	0.5303	1.042	1.81E-13	0.8	0.76	0.85	9.47E-15	0.7983	9.47E-15	0.7983	0.6747	0
3	171862283	rs35159555	A	0.08524	1.345	0.9597	1.886	1.71E-13	1.24	1.17	1.32	7.57E-14	1.2429	7.57E-14	1.2429	0.6419	0
3	171862347	rs17480104	A	0.08523	0.7434	0.5303	1.042	1.83E-13	0.8	0.76	0.85	9.47E-15	0.7983	9.47E-15	0.7983	0.6747	0
3	171862406	rs35718709	T	0.08524	1.345	0.9597	1.886	1.86E-13	1.24	1.17	1.32	7.57E-14	1.2429	7.57E-14	1.2429	0.6419	0
3	171862418	rs17420793	A	0.08524	1.345	0.9597	1.886	1.86E-13	1.24	1.17	1.32	7.57E-14	1.2429	7.57E-14	1.2429	0.6419	0
3	171862472	rs34139626	T	0.08524	1.345	0.9597	1.886	1.86E-13	1.24	1.17	1.32	7.57E-14	1.2429	7.57E-14	1.2429	0.6419	0
3	171862522	rs12493217	C	0.08299	0.7422	0.5298	1.04	1.32E-13	0.81	0.76	0.86	6.26E-14	0.8081	6.26E-14	0.8081	0.6162	0
3	171862583	rs12493242	T	0.08299	0.7422	0.5298	1.04	1.01E-13	0.81	0.76	0.85	6.26E-14	0.8081	6.26E-14	0.8081	0.6162	0

3	171862956	rs17480220	T	0.08524	1.345	0.9597	1.886	2.50E-13	1.24	1.17	1.32	7.57E-14	1.2429	7.57E-14	1.2429	0.6419	0
3	171863783	rs1828671	T	0.08523	0.7434	0.5303	1.042	2.01E-13	0.8	0.76	0.85	1.15E-14	0.7983	1.15E-14	0.7983	0.6747	0
3	171864222	rs17421040	A	0.09434	0.7489	0.5337	1.051	1.77E-13	0.8	0.76	0.85	3.19E-14	0.7984	3.19E-14	0.7984	0.7067	0
3	171864423	rs17421075	C	0.08523	0.7434	0.5303	1.042	1.32E-13	0.8	0.76	0.85	1.15E-14	0.7983	1.15E-14	0.7983	0.6747	0
3	171864641	rs34131881	T	0.08523	0.7434	0.5303	1.042	1.51E-13	0.8	0.76	0.85	1.15E-14	0.7983	1.15E-14	0.7983	0.6747	0
3	171864678	rs13080733	A	0.08524	1.345	0.9597	1.886	1.58E-13	1.24	1.17	1.32	9.08E-14	1.2429	9.08E-14	1.2429	0.642	0
3	171864810	rs11914718	T	0.08524	1.345	0.9597	1.886	2.08E-13	1.24	1.17	1.32	9.08E-14	1.2429	9.08E-14	1.2429	0.642	0
3	171864943	rs11926168	C	0.08523	0.7434	0.5303	1.042	1.14E-13	0.8	0.76	0.85	1.15E-14	0.7983	1.15E-14	0.7983	0.6747	0
3	171864944	rs11926169	A	0.08523	0.7434	0.5303	1.042	1.12E-13	0.8	0.76	0.85	1.15E-14	0.7983	1.15E-14	0.7983	0.6747	0
3	171864960	rs11918437	A	0.08524	1.345	0.9597	1.886	1.05E-13	1.25	1.18	1.32	1.15E-14	1.2526	1.15E-14	1.2526	0.6752	0
3	171865321	rs11927027	A	0.0952	0.7494	0.534	1.052	1.16E-13	0.8	0.76	0.85	1.24E-14	0.7985	1.24E-14	0.7985	0.7095	0
3	171865530	rs12495924	C	0.08032	1.356	0.9639	1.908	2.75E-13	1.24	1.17	1.32	1.06E-13	1.2431	1.06E-13	1.2431	0.6128	0
3	171865583	rs12486488	T	0.08032	1.356	0.9639	1.908	2.68E-13	1.24	1.17	1.32	1.06E-13	1.2431	1.06E-13	1.2431	0.6128	0
3	171866206	rs16856736	T	0.08369	1.344	0.9614	1.879	2.12E-12	1.24	1.17	1.32	5.02E-13	1.2431	5.02E-13	1.2431	0.6427	0
3	171867878	rs7648132	A	0.2223	0.8107	0.5787	1.136	5.09E-11	0.82	0.78	0.87	1.50E-11	0.8197	1.50E-11	0.8197	0.9479	0
3	171871063	rs62281899	A	0.2257	0.813	0.5817	1.136	6.39E-11	0.82	0.78	0.87	1.12E-11	0.8198	1.12E-11	0.8198	0.9606	0
3	171871424	rs12496525	T	0.2257	0.813	0.5817	1.136	9.79E-11	0.83	0.78	0.87	1.67E-10	0.8295	1.67E-10	0.8295	0.905	0
3	171871683	rs12496652	A	0.2257	0.813	0.5817	1.136	7.24E-11	0.82	0.78	0.87	1.12E-11	0.8198	1.12E-11	0.8198	0.9606	0
3	171872135	rs17421152	T	0.2257	1.23	0.88	1.719	7.31E-11	1.21	1.14	1.29	6.54E-11	1.2106	6.54E-11	1.2106	0.9247	0
3	171873407	rs3850192	T	0.2257	0.813	0.5817	1.136	4.51E-11	0.82	0.78	0.87	1.12E-11	0.8198	1.12E-11	0.8198	0.9606	0
3	171876667	rs56312515	T	0.2257	0.813	0.5817	1.136	5.28E-11	0.82	0.78	0.87	9.56E-12	0.8198	9.56E-12	0.8198	0.9606	0
3	171877361	rs10755129	C	0.2257	0.813	0.5817	1.136	3.92E-11	0.82	0.78	0.87	1.12E-11	0.8198	1.12E-11	0.8198	0.9606	0
3	171878819	rs11914354	A	0.2548	0.8235	0.5895	1.15	3.59E-11	0.82	0.78	0.87	1.04E-11	0.8201	1.04E-11	0.8201	0.9804	0
3	171901726	rs3850195	A	0.251	0.8484	0.6407	1.123	1.26E-10	0.85	0.81	0.89	6.78E-11	0.85	6.78E-11	0.85	0.9897	0
3	171902440	rs7619019	A	0.3104	0.8643	0.6521	1.146	1.23E-11	0.84	0.8	0.88	4.03E-12	0.8407	4.03E-12	0.8407	0.8452	0
3	171902444	rs73178251	T	0.3104	0.8643	0.6521	1.146	1.22E-11	0.84	0.8	0.88	4.03E-12	0.8407	4.03E-12	0.8407	0.8452	0
3	171903031	rs9854807	C	0.3019	1.16	0.8752	1.537	1.64E-11	1.19	1.13	1.25	4.40E-12	1.1891	4.40E-12	1.1891	0.8611	0
3	171905622	rs9870071	A	0.1829	1.21	0.9142	1.6	1.15E-11	1.19	1.13	1.25	3.03E-12	1.1906	3.03E-12	1.1906	0.9085	0
3	171905850	rs2422405	A	0.08501	1.268	0.9678	1.66	8.16E-09	1.16	1.1	1.22	3.46E-09	1.1636	3.46E-09	1.1636	0.5253	0
3	171906568	rs4594649	C	0.2108	0.8368	0.6331	1.106	3.01E-09	0.86	0.81	0.9	4.45E-09	0.8592	4.45E-09	0.8592	0.8502	0
3	171906631	rs2901739	A	0.1829	1.21	0.9142	1.6	1.95E-10	1.18	1.12	1.25	1.48E-10	1.181	1.48E-10	1.181	0.8627	0
3	171907177	rs4894515	T	0.223	1.189	0.9001	1.57	2.97E-09	1.17	1.11	1.23	1.12E-09	1.1706	1.12E-09	1.1706	0.9111	0
3	171907421	rs55971088	T	0.1881	1.206	0.9125	1.594	2.36E-10	1.18	1.12	1.24	1.51E-10	1.1809	1.51E-10	1.1809	0.8803	0
3	171907678	rs12486288	A	0.1881	0.8292	0.6274	1.096	2.15E-10	0.85	0.8	0.89	3.12E-10	0.8493	3.12E-10	0.8493	0.8641	0
3	171907895	rs4894782	C	0.1881	1.206	0.9125	1.594	1.92E-10	1.18	1.12	1.25	1.51E-10	1.1809	1.51E-10	1.1809	0.8803	0
3	171910859	rs9868683	T	0.2761	1.166	0.8844	1.538	8.25E-12	1.19	1.13	1.25	3.41E-12	1.1892	3.41E-12	1.1892	0.887	0

3	171914980	rs4894517	A	0.2761	1.166	0.8844	1.538	1.65E-11	1.19	1.13	1.25	3.41E-12	1.1892	3.41E-12	1.1892	0.887	0
3	171915658	rs6801882	A	0.2761	1.166	0.8844	1.538	1.42E-11	1.19	1.13	1.25	3.41E-12	1.1892	3.41E-12	1.1892	0.887	0
3	171916607	rs56054326	C	0.2761	0.8576	0.6504	1.131	1.75E-11	0.84	0.8	0.89	3.05E-12	0.8405	3.05E-12	0.8405	0.885	0
3	171920272	rs9855075	A	0.5539	0.9164	0.6863	1.224	1.85E-08	0.85	0.81	0.9	6.14E-09	0.8522	6.14E-09	0.8522	0.6164	0
3	171928252	rs9832169	A	0.5415	1.094	0.8195	1.461	2.56E-08	1.17	1.11	1.23	1.68E-08	1.1673	1.68E-08	1.1673	0.6546	0
3	171937884	rs6792542	A	0.2743	0.8571	0.65	1.13	3.89E-11	0.85	0.81	0.89	7.23E-11	0.8502	7.23E-11	0.8502	0.9537	0
3	171941366	rs6787669	A	0.5512	1.092	0.8177	1.458	9.32E-09	1.18	1.11	1.25	6.83E-09	1.1767	6.83E-09	1.1767	0.606	0
3	172202779	rs6445044	A	0.5381	0.87	0.5584	1.355	6.44E-08	0.81	0.75	0.88	4.48E-08	0.8116	4.48E-08	0.8116	0.7556	0
3	172208583	rs4535251	T	0.8711	1.022	0.7856	1.33	3.11E-09	1.16	1.1	1.22	3.86E-09	1.1551	3.86E-09	1.1551	0.3538	0
3	172215120	rs73025487	A	0.8428	0.9622	0.6574	1.408	4.27E-10	0.81	0.76	0.87	5.18E-10	0.8141	5.18E-10	0.8141	0.3828	0
3	172215279	rs12629746	A	0.4711	0.9077	0.6975	1.181	6.10E-08	0.87	0.83	0.92	1.80E-08	0.8712	1.80E-08	0.8712	0.7563	0
3	172224239	rs6794186	A	0.2195	1.198	0.8977	1.6	2.09E-17	1.24	1.18	1.3	3.68E-18	1.2388	3.68E-18	1.2388	0.8177	0
3	172224436	rs4894527	T	0.2195	1.198	0.8977	1.6	2.31E-17	1.24	1.18	1.3	3.68E-18	1.2388	3.68E-18	1.2388	0.8177	0
3	172225227	rs1317940	A	0.1105	0.7762	0.5686	1.06	2.36E-12	0.83	0.79	0.88	4.16E-13	0.8285	4.16E-13	0.8285	0.6772	0
3	172225683	rs7428410	T	0.1028	1.297	0.9491	1.771	3.96E-11	1.19	1.13	1.26	1.83E-11	1.1928	1.83E-11	1.1928	0.5937	0
3	172225721	rs4423796	T	0.1161	1.283	0.9402	1.752	2.49E-12	1.2	1.14	1.27	1.29E-12	1.2021	1.29E-12	1.2021	0.6778	0
3	172226166	rs4894821	A	0.1105	1.288	0.9438	1.759	3.33E-12	1.2	1.14	1.26	1.25E-12	1.2023	1.25E-12	1.2023	0.6602	0
3	172227351	rs7638177	A	0.08181	0.7035	0.4734	1.045	2.26E-10	0.8	0.74	0.86	8.47E-11	0.7969	8.47E-11	0.7969	0.531	0
3	172227389	rs2132164	C	0.08112	1.317	0.9665	1.795	2.29E-12	1.2	1.14	1.27	1.05E-12	1.203	1.05E-12	1.203	0.5614	0
3	172227559	rs13073573	T	0.08112	1.317	0.9665	1.795	2.21E-12	1.2	1.14	1.27	1.05E-12	1.203	1.05E-12	1.203	0.5614	0
3	172228381	rs4525896	A	0.118	1.283	0.9387	1.753	6.89E-11	1.19	1.13	1.25	1.98E-11	1.1924	1.98E-11	1.1924	0.6415	0
3	172229645	rs4428215	A	0.04688	0.7373	0.5459	0.9958	4.52E-16	0.81	0.77	0.85	8.60E-17	0.8079	8.60E-17	0.8079	0.5456	0
3	172230221	rs34124854	T	0.1307	1.272	0.9311	1.737	1.36E-12	1.21	1.14	1.27	1.70E-13	1.2116	1.70E-13	1.2116	0.7567	0
3	172230809	rs7643618	A	0.04688	1.356	1.004	1.832	5.55E-16	1.23	1.17	1.3	2.80E-16	1.2334	2.80E-16	1.2334	0.5308	0
3	172231317	rs7644098	C	0.03315	1.384	1.026	1.868	6.51E-16	1.23	1.17	1.3	2.28E-16	1.2341	2.28E-16	1.2341	0.4463	0
3	172231822	rs35327886	A	0.09688	0.769	0.5639	1.049	2.00E-12	0.83	0.79	0.87	4.66E-13	0.8283	4.66E-13	0.8283	0.6341	0
3	172232181	rs13060354	A	0.08809	0.763	0.5592	1.041	1.82E-12	0.83	0.79	0.87	4.42E-13	0.8281	4.42E-13	0.8281	0.6006	0
3	172232359	rs4243423	A	0.09688	0.769	0.5639	1.049	1.73E-12	0.83	0.79	0.87	4.66E-13	0.8283	4.66E-13	0.8283	0.6341	0
3	172232432	rs4243424	T	0.1307	1.272	0.9311	1.737	1.34E-12	1.21	1.14	1.27	1.70E-13	1.2116	1.70E-13	1.2116	0.7567	0
3	172232874	rs4622921	A	0.1307	0.7863	0.5757	1.074	1.53E-12	0.83	0.79	0.87	5.59E-13	0.8288	5.59E-13	0.8288	0.7373	0
3	172233143	rs4280664	A	0.1243	1.277	0.935	1.744	9.85E-13	1.21	1.15	1.27	1.34E-13	1.2117	1.34E-13	1.2117	0.7381	0
3	172233370	rs4364195	A	0.1307	0.7863	0.5757	1.074	1.45E-12	0.83	0.79	0.87	5.59E-13	0.8288	5.59E-13	0.8288	0.7373	0
3	172233569	rs4287929	A	0.1307	1.272	0.9311	1.737	2.67E-12	1.2	1.14	1.27	1.66E-12	1.2019	1.66E-12	1.2019	0.7179	0
3	172233576	rs4605578	A	0.1307	0.7863	0.5757	1.074	1.71E-12	0.83	0.79	0.87	5.59E-13	0.8288	5.59E-13	0.8288	0.7373	0
3	172233930	rs7653242	A	0.2197	1.198	0.8979	1.598	1.95E-18	1.24	1.19	1.31	3.67E-18	1.2388	3.67E-18	1.2388	0.8172	0
3	172235435	rs4894412	T	0.1367	1.355	0.9082	2.021	9.27E-11	1.26	1.17	1.35	2.58E-11	1.2627	2.58E-11	1.2627	0.7257	0

3	172240073	rs7611338	A	0.1367	1.355	0.9082	2.021	7.87E-11	1.26	1.17	1.35	2.28E-11	1.2627	2.28E-11	1.2627	0.7257	0
3	172243669	rs35417004	T	0.1035	1.419	0.9312	2.162	5.59E-08	1.26	1.16	1.37	1.20E-08	1.2656	1.20E-08	1.2656	0.5872	0
3	172246177	rs4481179	C	0.04164	0.7316	0.5417	0.9882	2.51E-16	0.81	0.77	0.85	1.03E-16	0.8077	1.03E-16	0.8077	0.513	0
3	172246433	rs12635823	A	0.04164	0.7316	0.5417	0.9882	2.87E-16	0.81	0.77	0.85	1.03E-16	0.8077	1.03E-16	0.8077	0.513	0
3	172249260	rs13099331	T	0.1188	1.375	0.9216	2.051	4.87E-11	1.27	1.18	1.36	8.64E-12	1.273	8.64E-12	1.273	0.7015	0
3	172252907	rs4894531	C	0.1188	0.7273	0.4875	1.085	2.74E-11	0.79	0.73	0.84	1.62E-11	0.788	1.62E-11	0.788	0.6899	0
3	172253225	rs4243401	A	0.1339	0.7364	0.4936	1.099	2.82E-11	0.79	0.74	0.85	8.17E-12	0.7884	8.17E-12	0.7884	0.7345	0
3	172254873	rs12637941	A	0.08351	0.7047	0.4741	1.047	5.44E-11	0.79	0.74	0.85	1.48E-11	0.7872	1.48E-11	0.7872	0.578	0
3	172257048	rs13080090	T	0.1261	0.7317	0.4904	1.092	8.81E-11	0.79	0.74	0.85	1.89E-11	0.7882	1.89E-11	0.7882	0.7116	0
3	172265161	rs28641087	A	0.6481	1.15	0.6313	2.094	4.06E-10	0.73	0.66	0.8	1.35E-09	0.7389	0.3577	0.8289	0.1427	53.46
3	172267514	rs16845236	T	0.1028	1.387	0.9363	2.055	3.63E-11	1.26	1.18	1.35	1.91E-11	1.2637	1.91E-11	1.2637	0.6372	0
3	172269608	rs7647839	A	0.7376	0.906	0.5084	1.614	4.72E-09	1.33	1.21	1.46	8.41E-09	1.3167	0.1958	1.2255	0.1988	39.44
3	172269958	rs59540498	T	0.7376	1.104	0.6194	1.967	1.04E-08	0.76	0.69	0.83	2.62E-08	0.7674	0.1756	0.8179	0.2113	35.99
3	172274219	rs6445054	T	0.6153	0.9155	0.6489	1.292	2.40E-19	0.77	0.73	0.82	8.98E-20	0.7735	8.98E-20	0.7735	0.3306	0
3	172274597	rs6445055	A	0.6232	1.09	0.7734	1.536	2.30E-15	1.25	1.18	1.32	2.40E-15	1.2457	2.40E-15	1.2457	0.4397	0
3	172277815	rs4894535	T	0.8879	1.027	0.7129	1.478	2.97E-20	1.31	1.23	1.38	2.91E-20	1.3025	0.02936	1.2433	0.1963	40.11
3	172278729	rs6800129	C	0.498	0.8898	0.6348	1.247	2.52E-25	0.75	0.71	0.79	1.73E-24	0.7533	1.73E-24	0.7533	0.3275	0
3	172279709	rs4894414	T	0.5606	1.107	0.7857	1.56	1.21E-26	1.36	1.28	1.43	6.46E-27	1.3528	0.0001518	1.3192	0.2457	25.8
5	53126169	rs10069631	A	0.1635	0.7916	0.5698	1.1	1.12E-07	0.83	0.78	0.89	2.33E-08	0.8284	2.33E-08	0.8284	0.7821	0
5	53128688	rs10471373	T	0.2321	0.8217	0.5955	1.134	2.57E-08	0.83	0.78	0.89	5.25E-09	0.8297	5.25E-09	0.8297	0.9522	0
5	53130776	rs6867917	T	0.4186	1.111	0.8604	1.435	6.19E-08	1.14	1.09	1.19	3.06E-08	1.139	3.06E-08	1.139	0.8461	0
5	53131090	rs10054234	A	0.1884	0.805	0.5828	1.112	1.97E-08	0.83	0.78	0.89	4.57E-09	0.829	4.57E-09	0.829	0.8556	0
5	53131816	rs1374058	T	0.1446	0.8274	0.6415	1.067	9.93E-08	0.88	0.84	0.92	3.74E-08	0.8782	3.74E-08	0.8782	0.6408	0
5	53138236	rs10053775	C	0.06971	0.7458	0.5432	1.024	2.25E-08	0.84	0.79	0.89	5.52E-09	0.8364	5.52E-09	0.8364	0.4701	0
5	53138302	rs10076879	A	0.06971	1.341	0.9766	1.841	2.25E-08	1.19	1.12	1.27	5.95E-09	1.1951	5.95E-09	1.1951	0.4682	0
5	53138391	rs10053848	A	0.06971	0.7458	0.5432	1.024	1.65E-08	0.84	0.79	0.89	5.52E-09	0.8364	5.52E-09	0.8364	0.4701	0
5	53139030	rs62357364	A	0.06971	0.7458	0.5432	1.024	1.89E-08	0.84	0.79	0.89	5.52E-09	0.8364	5.52E-09	0.8364	0.4701	0
5	53139497	rs10065796	C	0.06823	1.343	0.9782	1.843	8.39E-09	1.19	1.12	1.27	3.86E-09	1.1951	3.86E-09	1.1951	0.4619	0
5	53139556	rs10073544	A	0.06823	1.343	0.9782	1.843	6.48E-09	1.2	1.13	1.27	7.42E-10	1.2048	7.42E-10	1.2048	0.4935	0
5	53141069	rs11955696	T	0.06823	0.7449	0.5427	1.022	3.02E-08	0.84	0.79	0.9	3.99E-09	0.8364	3.99E-09	0.8364	0.465	0
5	53141947	rs7732297	A	0.0494	0.7747	0.6006	0.9993	9.75E-08	0.88	0.84	0.92	2.21E-08	0.8763	2.21E-08	0.8763	0.3347	0
5	53145528	rs10039786	C	0.06823	0.7449	0.5427	1.022	2.09E-09	0.83	0.78	0.88	3.72E-10	0.8268	3.72E-10	0.8268	0.5106	0
5	53148000	rs9292010	A	0.07542	1.333	0.971	1.831	6.11E-09	1.2	1.13	1.28	1.56E-09	1.2046	1.56E-09	1.2046	0.5236	0
5	53148883	rs17233845	T	0.07542	0.75	0.5462	1.03	1.79E-09	0.83	0.78	0.88	7.95E-10	0.8269	7.95E-10	0.8269	0.5386	0
5	53149124	rs10064585	T	0.06977	1.341	0.9766	1.841	2.82E-09	1.2	1.13	1.28	8.46E-10	1.2047	8.46E-10	1.2047	0.4998	0
5	53149418	rs11954163	T	0.06977	1.341	0.9766	1.841	2.55E-09	1.2	1.13	1.28	8.46E-10	1.2047	8.46E-10	1.2047	0.4998	0

5	53149743	rs13361332	T	0.06977	0.7458	0.5432	1.024	2.10E-09	0.83	0.78	0.88	3.77E-10	0.8269	3.77E-10	0.8269	0.5158	0
5	53149866	rs12515104	T	0.06977	1.341	0.9766	1.841	2.09E-09	1.2	1.13	1.28	8.46E-10	1.2047	8.46E-10	1.2047	0.4998	0
5	53150185	rs254614	T	0.2591	1.169	0.8911	1.535	2.18E-07	1.14	1.08	1.19	4.70E-08	1.1409	4.70E-08	1.1409	0.8584	0
5	53150444	rs254617	A	0.2591	1.169	0.8911	1.535	1.96E-07	1.14	1.08	1.19	4.70E-08	1.1409	4.70E-08	1.1409	0.8584	0
5	53151524	rs10059321	T	0.1042	1.297	0.9477	1.776	2.78E-09	1.2	1.13	1.28	1.33E-09	1.2034	1.33E-09	1.2034	0.6338	0
5	53156676	rs6886825	T	0.09765	1.24	0.9614	1.598	1.56E-10	1.17	1.11	1.22	1.37E-11	1.1722	1.37E-11	1.1722	0.6595	0
5	53159825	rs6899102	A	0.09765	1.24	0.9614	1.598	7.75E-11	1.17	1.11	1.22	1.65E-11	1.1723	1.65E-11	1.1723	0.6595	0
5	53161723	rs34949026	A	0.1005	0.8083	0.627	1.042	3.71E-11	0.85	0.81	0.89	3.47E-12	0.8486	3.47E-12	0.8486	0.7027	0
5	53174246	rs1025027	A	0.112	1.228	0.9531	1.583	4.34E-10	1.17	1.11	1.23	9.98E-11	1.172	9.98E-11	1.172	0.7135	0
5	53182885	rs2112876	T	0.1927	1.226	0.9022	1.667	5.12E-13	1.25	1.18	1.33	3.63E-13	1.2491	3.63E-13	1.2491	0.9033	0
5	53187399	rs254510	C	0.1287	0.8223	0.6388	1.058	1.34E-13	0.84	0.8	0.88	1.46E-13	0.8394	1.46E-13	0.8394	0.8709	0
5	53188736	rs251584	T	0.4519	0.9038	0.6943	1.176	2.67E-13	0.84	0.8	0.88	2.59E-13	0.8419	2.59E-13	0.8419	0.592	0
5	53189611	rs194215	A	0.5482	1.084	0.833	1.411	1.54E-13	1.19	1.14	1.25	5.57E-13	1.1865	5.57E-13	1.1865	0.4944	0
5	53190163	rs153665	T	0.5104	1.093	0.8388	1.424	5.94E-13	1.19	1.14	1.25	6.40E-13	1.1869	6.40E-13	1.1869	0.5353	0
5	53191765	rs251580	A	0.5395	0.9203	0.706	1.2	3.54E-13	0.84	0.8	0.88	6.01E-13	0.8424	6.01E-13	0.8424	0.5065	0
5	53194539	rs254504	T	0.5482	1.084	0.833	1.411	2.64E-13	1.19	1.14	1.25	5.57E-13	1.1865	5.57E-13	1.1865	0.4944	0
5	53195963	rs16774	A	0.2895	1.164	0.8788	1.542	3.03E-12	1.2	1.14	1.26	1.35E-12	1.1988	1.35E-12	1.1988	0.8344	0
5	53196847	rs254503	A	0.1237	1.22	0.9472	1.57	8.28E-14	1.19	1.14	1.25	5.15E-14	1.191	5.15E-14	1.191	0.8494	0
5	53197103	rs17241927	T	0.2176	1.213	0.8922	1.65	1.71E-13	1.26	1.18	1.34	5.12E-14	1.2582	5.12E-14	1.2582	0.812	0
5	53198133	rs17242004	A	0.1541	1.202	0.9333	1.548	1.69E-13	1.19	1.14	1.25	7.62E-14	1.1904	7.62E-14	1.1904	0.939	0
5	53198143	rs61125444	A	0.2993	0.8619	0.6509	1.141	2.56E-12	0.83	0.79	0.88	4.60E-13	0.831	4.60E-13	0.831	0.7955	0
5	53198906	rs774234	A	0.4688	1.103	0.8466	1.436	6.39E-13	1.19	1.13	1.24	3.02E-13	1.1872	3.02E-13	1.1872	0.5792	0
5	53199127	rs62357405	A	0.2176	1.213	0.8922	1.65	2.08E-13	1.26	1.18	1.34	5.12E-14	1.2582	5.12E-14	1.2582	0.812	0
5	53199215	rs1392541	T	0.1541	0.832	0.6461	1.071	3.46E-13	0.84	0.8	0.88	6.72E-14	0.8397	6.72E-14	0.8397	0.9418	0
5	53199219	rs62357406	A	0.2993	0.8619	0.6509	1.141	3.43E-12	0.83	0.79	0.88	4.60E-13	0.831	4.60E-13	0.831	0.7955	0
5	53200622	rs62357407	A	0.2043	1.221	0.8972	1.66	3.80E-13	1.25	1.18	1.33	2.71E-13	1.2489	2.71E-13	1.2489	0.8834	0
5	53202440	rs192262	T	0.1821	0.8423	0.6546	1.084	8.08E-14	0.84	0.8	0.88	6.03E-14	0.8401	6.03E-14	0.8401	0.9833	0
5	53202943	rs166129	A	0.4418	1.109	0.8519	1.444	7.89E-13	1.19	1.13	1.24	2.86E-13	1.1874	2.86E-13	1.1874	0.6063	0
5	53204165	rs56197881	A	0.2779	0.856	0.6464	1.134	2.75E-12	0.83	0.79	0.88	4.32E-13	0.8308	4.32E-13	0.8308	0.8323	0
5	53204209	rs251512	T	0.5311	1.088	0.8359	1.416	4.89E-13	1.19	1.14	1.25	5.38E-13	1.1867	5.38E-13	1.1867	0.5116	0
5	53204982	rs254198	T	0.1452	1.207	0.9371	1.554	1.01E-12	1.18	1.13	1.24	9.89E-13	1.1809	9.89E-13	1.1809	0.8632	0
5	53206696	rs2548321	T	0.1616	1.198	0.9303	1.542	2.25E-13	1.19	1.14	1.25	7.88E-14	1.1903	7.88E-14	1.1903	0.9592	0
5	53208231	rs1838736	T	0.3075	1.157	0.8743	1.531	4.74E-12	1.2	1.14	1.26	1.42E-12	1.1986	1.42E-12	1.1986	0.8016	0
5	53209183	rs7711335	A	0.2961	0.8611	0.6506	1.14	2.46E-11	0.84	0.8	0.89	6.79E-12	0.8407	6.79E-12	0.8407	0.8645	0
5	53209434	rs254199	C	0.1616	0.8349	0.6485	1.075	6.10E-13	0.84	0.8	0.88	6.96E-14	0.8398	6.96E-14	0.8398	0.9629	0
5	53209626	rs62357423	T	0.2961	1.161	0.8773	1.537	2.91E-11	1.19	1.13	1.25	6.31E-12	1.1891	6.31E-12	1.1891	0.8652	0

5	53209780	rs152977	A	0.5639	1.08	0.8309	1.405	7.69E-13	1.19	1.13	1.25	5.78E-13	1.1864	5.78E-13	1.1864	0.4759	0
5	53209861	rs62357424	T	0.2961	0.8611	0.6506	1.14	2.92E-11	0.84	0.8	0.89	5.65E-12	0.8406	5.65E-12	0.8406	0.8645	0
5	53210280	rs2897474	A	0.3158	1.153	0.8731	1.522	1.85E-11	1.19	1.13	1.25	5.51E-12	1.1888	5.51E-12	1.1888	0.8265	0
5	53211714	rs34277022	T	0.3481	1.143	0.8648	1.51	1.39E-11	1.19	1.13	1.25	5.96E-12	1.1885	5.96E-12	1.1885	0.7803	0
5	53211721	rs56686182	T	0.2961	0.8611	0.6506	1.14	1.66E-11	0.84	0.8	0.88	6.79E-12	0.8407	6.79E-12	0.8407	0.8645	0
5	53211812	rs2897475	A	0.3481	1.143	0.8648	1.51	1.36E-09	1.17	1.12	1.24	2.31E-09	1.1691	2.31E-09	1.1691	0.8718	0
5	53212451	rs1445992	A	0.2961	0.8611	0.6506	1.14	1.78E-11	0.84	0.8	0.89	6.79E-12	0.8407	6.79E-12	0.8407	0.8645	0
5	53213121	rs251517	A	0.5459	1.084	0.834	1.41	5.38E-13	1.19	1.13	1.25	5.57E-13	1.1865	5.57E-13	1.1865	0.4929	0
5	53213746	rs16881069	T	0.2837	1.166	0.8808	1.543	1.63E-11	1.19	1.13	1.25	7.29E-12	1.1892	7.29E-12	1.1892	0.8885	0
5	53214329	rs6414820	T	0.3342	1.147	0.8683	1.515	8.81E-12	1.19	1.13	1.25	5.78E-12	1.1886	5.78E-12	1.1886	0.7988	0
5	53214615	rs6895908	A	0.2837	1.166	0.8808	1.543	1.44E-11	1.19	1.13	1.25	7.29E-12	1.1892	7.29E-12	1.1892	0.8885	0
5	53214634	rs6873915	C	0.2947	0.861	0.6507	1.139	3.89E-12	0.83	0.79	0.88	4.55E-13	0.831	4.55E-13	0.831	0.8007	0
5	53214703	rs39860	A	0.1898	1.184	0.9199	1.523	1.22E-13	1.19	1.14	1.25	8.91E-14	1.1898	8.91E-14	1.1898	0.9692	0
5	53214819	rs6860071	T	0.3311	1.148	0.869	1.517	9.08E-12	1.19	1.13	1.25	5.74E-12	1.1887	5.74E-12	1.1887	0.8034	0
5	53215195	rs6896916	A	0.3311	1.148	0.869	1.517	7.98E-12	1.19	1.13	1.25	5.74E-12	1.1887	5.74E-12	1.1887	0.8034	0
5	53215294	rs6897066	A	0.3311	1.148	0.869	1.517	6.49E-12	1.19	1.13	1.25	5.74E-12	1.1887	5.74E-12	1.1887	0.8034	0
5	53215392	rs6875198	T	0.3311	0.871	0.6592	1.151	7.71E-12	0.84	0.8	0.88	5.14E-12	0.841	5.14E-12	0.841	0.8018	0
5	53216736	rs7725098	A	0.2808	0.8571	0.6475	1.134	9.98E-12	0.84	0.8	0.88	6.51E-12	0.8405	6.51E-12	0.8405	0.8897	0
5	53216779	rs7726216	T	0.2808	0.8571	0.6475	1.134	9.98E-12	0.84	0.8	0.88	6.51E-12	0.8405	6.51E-12	0.8405	0.8897	0
5	53216806	rs7707949	A	0.2808	1.167	0.8815	1.544	9.97E-12	1.19	1.13	1.25	7.24E-12	1.1893	7.24E-12	1.1893	0.8931	0
5	53216931	rs7725380	A	0.2808	0.8571	0.6475	1.134	8.79E-12	0.84	0.8	0.88	6.51E-12	0.8405	6.51E-12	0.8405	0.8897	0
5	53217156	rs7726834	T	0.3311	0.871	0.6592	1.151	7.83E-12	0.84	0.8	0.88	5.14E-12	0.841	5.14E-12	0.841	0.8018	0
5	53217378	rs10940294	T	0.3018	1.159	0.8758	1.534	1.20E-11	1.19	1.13	1.25	7.68E-12	1.189	7.68E-12	1.189	0.8558	0
5	53217705	rs2406615	T	0.1662	0.8115	0.6038	1.091	1.95E-13	0.81	0.76	0.85	2.88E-13	0.8101	2.88E-13	0.8101	0.9904	0
5	53218404	rs10043264	T	0.3311	1.148	0.869	1.517	7.08E-12	1.19	1.13	1.25	5.74E-12	1.1887	5.74E-12	1.1887	0.8034	0
5	53218777	rs12521034	A	0.2918	1.163	0.8786	1.538	5.31E-12	1.2	1.14	1.26	1.36E-12	1.1988	1.36E-12	1.1988	0.8293	0
5	53218847	rs6897187	A	0.2918	0.8602	0.6501	1.138	4.43E-12	0.84	0.79	0.88	1.15E-11	0.8406	1.15E-11	0.8406	0.87	0
5	53219317	rs6897912	A	0.2918	0.8602	0.6501	1.138	3.98E-12	0.83	0.79	0.88	4.51E-13	0.831	4.51E-13	0.831	0.8056	0
5	53220186	rs9292012	T	0.3311	1.148	0.869	1.517	7.44E-12	1.19	1.13	1.25	5.74E-12	1.1887	5.74E-12	1.1887	0.8034	0
5	53220380	rs9292013	C	0.3311	1.148	0.869	1.517	6.24E-12	1.19	1.13	1.25	5.74E-12	1.1887	5.74E-12	1.1887	0.8034	0
5	53220479	rs9918202	T	0.2808	1.167	0.8815	1.544	1.74E-11	1.19	1.13	1.25	7.24E-12	1.1893	7.24E-12	1.1893	0.8931	0
5	53220893	rs62357427	A	0.3311	1.148	0.869	1.517	6.07E-12	1.19	1.13	1.25	5.74E-12	1.1887	5.74E-12	1.1887	0.8034	0
5	53220934	rs11955319	T	0.3311	1.148	0.869	1.517	4.64E-12	1.19	1.13	1.25	5.74E-12	1.1887	5.74E-12	1.1887	0.8034	0
5	53221059	rs10940296	A	0.2808	1.167	0.8815	1.544	1.63E-11	1.19	1.13	1.25	7.24E-12	1.1893	7.24E-12	1.1893	0.8931	0
5	53221335	rs7705986	T	0.3311	1.148	0.869	1.517	4.86E-12	1.19	1.13	1.25	5.74E-12	1.1887	5.74E-12	1.1887	0.8034	0
5	53221668	rs7724504	T	0.2918	0.8602	0.6501	1.138	1.96E-12	0.83	0.79	0.88	4.51E-13	0.831	4.51E-13	0.831	0.8056	0

5	53221761	rs7710423	T	0.3311	1.148	0.869	1.517	2.45E-12	1.2	1.14	1.26	5.64E-13	1.1983	5.64E-13	1.1983	0.759	0
5	53221818	rs7724705	T	0.2808	0.8571	0.6475	1.134	9.44E-12	0.84	0.8	0.88	6.51E-12	0.8405	6.51E-12	0.8405	0.8897	0
5	53222029	rs7725139	A	0.3311	0.871	0.6592	1.151	2.46E-12	0.84	0.8	0.88	5.14E-12	0.841	5.14E-12	0.841	0.8018	0
5	53222305	rs7725467	T	0.3311	0.871	0.6592	1.151	2.20E-12	0.84	0.8	0.88	5.14E-12	0.841	5.14E-12	0.841	0.8018	0
5	53222409	rs4106003	A	0.2808	0.8571	0.6475	1.134	1.03E-11	0.84	0.8	0.88	6.51E-12	0.8405	6.51E-12	0.8405	0.8897	0
5	53222774	rs10071518	T	0.3311	1.148	0.869	1.517	2.37E-12	1.2	1.14	1.26	5.64E-13	1.1983	5.64E-13	1.1983	0.759	0
5	53222846	rs11743062	T	0.2918	0.8602	0.6501	1.138	1.39E-12	0.83	0.79	0.88	4.51E-13	0.831	4.51E-13	0.831	0.8056	0
5	53222994	rs10070123	A	0.3311	1.148	0.869	1.517	1.73E-12	1.2	1.14	1.26	5.64E-13	1.1983	5.64E-13	1.1983	0.759	0
5	53225602	rs56909170	A	0.1662	0.8115	0.6038	1.091	5.42E-14	0.8	0.76	0.85	1.21E-14	0.8004	1.21E-14	0.8004	0.926	0
5	53227498	rs6863326	T	0.2808	1.167	0.8815	1.544	7.66E-12	1.19	1.13	1.25	7.24E-12	1.1893	7.24E-12	1.1893	0.8931	0
5	53229068	rs153621	T	0.1934	1.182	0.9186	1.522	8.44E-15	1.2	1.15	1.26	7.91E-15	1.1994	7.91E-15	1.1994	0.9081	0
5	53229298	rs6870259	T	0.2808	1.167	0.8815	1.544	8.28E-12	1.19	1.13	1.25	7.24E-12	1.1893	7.24E-12	1.1893	0.8931	0
5	53229609	rs254146	A	0.1542	1.203	0.9331	1.55	1.30E-13	1.19	1.14	1.25	7.60E-14	1.1904	7.60E-14	1.1904	0.9342	0
5	53229633	rs6450113	T	0.3223	0.8687	0.6575	1.148	2.09E-12	0.84	0.79	0.88	6.06E-12	0.8409	6.06E-12	0.8409	0.8161	0
5	53229751	rs4361475	A	0.3223	1.151	0.8712	1.521	2.14E-12	1.2	1.14	1.26	5.51E-13	1.1984	5.51E-13	1.1984	0.7729	0
5	53229906	rs7720876	A	0.3223	1.151	0.8712	1.521	2.47E-12	1.2	1.14	1.26	5.51E-13	1.1984	5.51E-13	1.1984	0.7729	0
5	53230056	rs7721172	A	0.2729	1.17	0.8838	1.549	7.08E-12	1.19	1.13	1.25	7.08E-12	1.1894	7.08E-12	1.1894	0.9072	0
5	53230873	rs6450115	C	0.2982	1.16	0.8768	1.536	7.22E-12	1.19	1.13	1.25	7.62E-12	1.189	7.62E-12	1.189	0.8605	0
5	53231039	rs6450116	A	0.2871	0.8591	0.6495	1.136	8.51E-12	0.84	0.8	0.88	6.63E-12	0.8406	6.63E-12	0.8406	0.8768	0
5	53231042	rs62357442	A	0.1781	0.8154	0.6059	1.097	2.21E-13	0.81	0.76	0.85	3.63E-13	0.8102	3.63E-13	0.8102	0.9657	0
5	53231141	rs6450117	T	0.2982	1.16	0.8768	1.536	6.95E-12	1.19	1.13	1.25	7.62E-12	1.189	7.62E-12	1.189	0.8605	0
5	53231175	rs153121	T	0.5517	1.083	0.8331	1.408	5.32E-14	1.2	1.14	1.26	4.32E-14	1.1961	4.32E-14	1.1961	0.4505	0
5	53231957	rs153120	T	0.4683	0.9072	0.6974	1.18	1.02E-13	0.84	0.8	0.88	2.68E-13	0.842	2.68E-13	0.842	0.5723	0
5	53232376	rs6872591	A	0.2982	0.8618	0.6512	1.141	6.24E-12	0.84	0.8	0.88	6.83E-12	0.8407	6.83E-12	0.8407	0.86	0
5	53232385	rs153119	T	0.6187	1.069	0.8213	1.392	6.76E-14	1.2	1.14	1.26	4.92E-14	1.1957	4.92E-14	1.1957	0.3979	0
5	53232509	rs6876789	A	0.3503	0.8757	0.6629	1.157	2.71E-12	0.84	0.8	0.88	5.39E-12	0.8411	5.39E-12	0.8411	0.7731	0
5	53232713	rs6897965	A	0.3503	1.142	0.8644	1.508	2.66E-12	1.2	1.14	1.26	5.91E-13	1.1981	5.91E-13	1.1981	0.7315	0
5	53232886	rs6898261	A	0.2982	1.16	0.8768	1.536	7.01E-12	1.19	1.13	1.25	7.62E-12	1.189	7.62E-12	1.189	0.8605	0
5	53233116	rs61388559	A	0.2982	0.8618	0.6512	1.141	9.51E-12	0.84	0.8	0.88	6.83E-12	0.8407	6.83E-12	0.8407	0.86	0
5	53233256	rs6898901	A	0.3503	1.142	0.8644	1.508	2.26E-12	1.2	1.14	1.26	5.91E-13	1.1981	5.91E-13	1.1981	0.7315	0
5	53234200	rs6882524	A	0.2982	0.8618	0.6512	1.141	6.11E-12	0.84	0.8	0.88	6.83E-12	0.8407	6.83E-12	0.8407	0.86	0
5	53236620	rs6450120	A	0.2982	1.16	0.8768	1.536	3.94E-12	1.19	1.14	1.26	7.62E-12	1.189	7.62E-12	1.189	0.8605	0
5	53237456	rs7704622	A	0.3503	0.8757	0.6629	1.157	2.22E-12	0.84	0.8	0.88	5.39E-12	0.8411	5.39E-12	0.8411	0.7731	0
5	53237885	rs10067414	T	0.3503	0.8757	0.6629	1.157	2.03E-12	0.84	0.79	0.88	5.39E-12	0.8411	5.39E-12	0.8411	0.7731	0
5	53237922	rs10067418	A	0.3503	0.8757	0.6629	1.157	1.57E-12	0.83	0.79	0.88	1.88E-13	0.8314	1.88E-13	0.8314	0.7104	0
5	53238151	rs7705771	A	0.3503	0.8757	0.6629	1.157	2.28E-12	0.84	0.8	0.88	5.39E-12	0.8411	5.39E-12	0.8411	0.7731	0

5	53238232	rs7707051	A	0.3096	0.8649	0.6537	1.144	1.65E-12	0.83	0.79	0.88	4.74E-13	0.8311	4.74E-13	0.8311	0.7766	0
5	53239028	rs6896855	A	0.3503	0.8757	0.6629	1.157	2.21E-12	0.84	0.8	0.88	5.39E-12	0.8411	5.39E-12	0.8411	0.7731	0
5	53239160	rs6897000	T	0.3503	1.142	0.8644	1.508	2.03E-12	1.2	1.14	1.26	5.91E-13	1.1981	5.91E-13	1.1981	0.7315	0
5	53240216	rs1816904	A	0.3503	1.142	0.8644	1.508	1.66E-12	1.2	1.14	1.26	7.24E-13	1.1981	7.24E-13	1.1981	0.7315	0
5	53240557	rs17242584	A	0.1781	0.8154	0.6059	1.097	4.70E-14	0.8	0.76	0.85	1.28E-14	0.8006	1.28E-14	0.8006	0.9017	0
5	53240936	rs27316	T	0.4683	0.9072	0.6974	1.18	4.23E-14	0.83	0.8	0.87	6.06E-15	0.8323	6.06E-15	0.8323	0.5141	0
5	53242007	rs6450121	T	0.3503	1.142	0.8644	1.508	1.31E-12	1.2	1.14	1.26	7.24E-13	1.1981	7.24E-13	1.1981	0.7315	0
5	53242034	rs7734784	T	0.3503	1.142	0.8644	1.508	1.29E-12	1.2	1.14	1.26	7.24E-13	1.1981	7.24E-13	1.1981	0.7315	0
5	53243656	rs731383	T	0.3161	0.8666	0.6551	1.146	2.07E-12	0.83	0.79	0.88	4.82E-13	0.8312	4.82E-13	0.8312	0.7662	0
5	53243997	rs1549247	A	0.3465	1.143	0.8656	1.509	6.87E-13	1.2	1.14	1.26	7.17E-13	1.1982	7.17E-13	1.1982	0.7354	0
5	53244105	rs733420	T	0.3045	0.8635	0.6526	1.143	7.92E-12	0.84	0.8	0.88	6.94E-12	0.8407	6.94E-12	0.8407	0.8493	0
5	53244425	rs733421	T	0.2158	1.195	0.9012	1.585	1.14E-11	1.19	1.13	1.25	5.96E-12	1.1902	5.96E-12	1.1902	0.9771	0
5	53245564	rs6892815	T	0.225	0.8397	0.6333	1.113	3.02E-12	0.83	0.79	0.88	3.65E-13	0.8303	3.65E-13	0.8303	0.9367	0
5	53245835	rs6450122	A	0.2583	0.8506	0.6425	1.126	2.02E-12	0.84	0.79	0.88	5.08E-12	0.8403	5.08E-12	0.8403	0.9313	0
5	53246363	rs1374052	T	0.244	1.181	0.8926	1.563	1.00E-12	1.2	1.14	1.26	4.39E-13	1.1994	4.39E-13	1.1994	0.9125	0
5	53246573	rs1374053	A	0.225	0.8397	0.6333	1.113	3.01E-12	0.83	0.79	0.88	3.65E-13	0.8303	3.65E-13	0.8303	0.9367	0
5	53246821	rs1374054	A	0.225	1.191	0.8981	1.579	3.01E-12	1.2	1.14	1.26	1.11E-12	1.1997	1.11E-12	1.1997	0.959	0
5	53247761	rs454858	C	0.352	0.8809	0.6745	1.15	5.89E-13	0.84	0.8	0.88	3.97E-13	0.8412	3.97E-13	0.8412	0.7311	0
5	53248259	rs251505	A	0.3458	1.137	0.8708	1.484	5.41E-13	1.19	1.14	1.25	3.55E-13	1.1884	3.55E-13	1.1884	0.7417	0
5	53248401	rs1445985	A	0.2158	0.8366	0.6308	1.11	1.03E-11	0.84	0.8	0.88	5.34E-12	0.8399	5.34E-12	0.8399	0.9779	0
5	53249579	rs58049370	T	0.1832	0.8246	0.6208	1.095	6.21E-12	0.84	0.8	0.88	5.72E-12	0.8395	5.72E-12	0.8395	0.9	0
5	53250011	rs6885965	T	0.1913	0.8277	0.6233	1.099	1.98E-12	0.83	0.79	0.88	4.80E-13	0.8299	4.80E-13	0.8299	0.9849	0
5	53250022	rs6866517	A	0.1871	1.21	0.9114	1.608	1.66E-12	1.2	1.14	1.27	1.42E-12	1.2003	1.42E-12	1.2003	0.955	0
5	53250445	rs1318732	A	0.1731	0.8204	0.617	1.091	5.69E-12	0.84	0.79	0.88	9.37E-12	0.8394	9.37E-12	0.8394	0.8729	0
5	53250575	rs2406617	T	0.1731	0.8204	0.617	1.091	5.91E-12	0.84	0.79	0.88	9.37E-12	0.8394	9.37E-12	0.8394	0.8729	0
5	53250862	rs2406618	A	0.1687	0.8186	0.6156	1.089	5.91E-12	0.84	0.79	0.88	9.21E-12	0.8393	9.21E-12	0.8393	0.8613	0
5	53250970	rs1960785	T	0.2047	1.201	0.9049	1.594	9.14E-13	1.2	1.14	1.27	1.03E-12	1.2	1.03E-12	1.2	0.9955	0
5	53250994	rs1960786	A	0.1687	0.8186	0.6156	1.089	5.34E-12	0.84	0.79	0.88	9.21E-12	0.8393	9.21E-12	0.8393	0.8613	0
5	53251069	rs1813600	T	0.1687	1.222	0.9187	1.624	4.89E-12	1.2	1.14	1.26	1.09E-12	1.2007	1.09E-12	1.2007	0.9021	0
5	53252893	rs16881173	A	0.1431	0.8075	0.6065	1.075	8.40E-13	0.83	0.79	0.87	6.98E-13	0.8293	6.98E-13	0.8293	0.8531	0
5	53252959	rs153619	T	0.137	0.8247	0.6397	1.063	1.24E-14	0.83	0.8	0.87	1.23E-15	0.8298	1.23E-15	0.8298	0.9612	0
5	53253057	rs72762377	A	0.1251	0.791	0.5862	1.067	2.10E-13	0.81	0.76	0.85	2.79E-13	0.8093	2.79E-13	0.8093	0.8788	0
5	53254320	rs12517334	A	0.1329	1.246	0.9353	1.659	2.48E-12	1.2	1.14	1.26	9.26E-13	1.2014	9.26E-13	1.2014	0.8	0
5	53254409	rs153618	A	0.1726	0.8385	0.6511	1.08	4.42E-15	0.83	0.79	0.87	1.47E-15	0.8303	1.47E-15	0.8303	0.9381	0
5	53254750	rs27324	T	0.2057	0.8412	0.6435	1.1	2.42E-14	0.83	0.79	0.87	3.68E-15	0.8303	3.68E-15	0.8303	0.9231	0
5	53255681	rs56296560	T	0.1375	0.8049	0.6045	1.072	2.90E-13	0.82	0.78	0.87	1.84E-14	0.8195	1.84E-14	0.8195	0.9004	0

5	53256409	rs27323	A	0.2753	1.16	0.8882	1.516	1.10E-14	1.2	1.15	1.26	2.19E-14	1.1988	2.19E-14	1.1988	0.8066	0
5	53256752	rs16881189	A	0.1276	0.8002	0.6007	1.066	1.25E-12	0.83	0.79	0.88	1.99E-13	0.8291	1.99E-13	0.8291	0.8056	0
5	53257875	rs12520159	A	0.1342	1.257	0.9319	1.695	2.89E-14	1.25	1.18	1.32	8.37E-15	1.2502	8.37E-15	1.2502	0.9713	0
5	53260952	rs4865543	T	0.2311	0.8573	0.6663	1.103	4.41E-16	0.82	0.79	0.86	2.87E-17	0.8212	2.87E-17	0.8212	0.7337	0
5	53261380	rs977576	A	0.2356	1.164	0.9054	1.498	9.18E-17	1.22	1.16	1.28	2.55E-17	1.2181	2.55E-17	1.2181	0.7189	0
5	53261716	rs977575	C	0.2399	1.163	0.9042	1.495	1.23E-16	1.22	1.16	1.27	2.57E-17	1.2181	2.57E-17	1.2181	0.7138	0
5	53262931	rs38058	A	0.3014	1.161	0.8745	1.542	6.06E-16	1.24	1.18	1.31	1.05E-15	1.2373	1.05E-15	1.2373	0.6547	0
5	53264155	rs38056	T	0.3333	0.8695	0.655	1.154	6.79E-17	0.8	0.76	0.84	7.93E-17	0.8022	7.93E-17	0.8022	0.5711	0
5	53264814	rs38055	A	0.3333	0.8695	0.655	1.154	1.41E-16	0.8	0.76	0.84	7.93E-17	0.8022	7.93E-17	0.8022	0.5711	0
5	53265155	rs40232	A	0.3333	1.15	0.8663	1.527	6.45E-17	1.25	1.19	1.32	7.94E-17	1.2465	7.94E-17	1.2465	0.5708	0
5	53267293	rs153129	A	0.3333	0.8695	0.655	1.154	8.74E-17	0.8	0.76	0.84	7.93E-17	0.8022	7.93E-17	0.8022	0.5711	0
5	53267694	rs251528	A	0.3333	1.15	0.8663	1.527	5.11E-17	1.25	1.19	1.32	7.94E-17	1.2465	7.94E-17	1.2465	0.5708	0
5	53268141	rs57244200	T	0.2915	1.145	0.8902	1.473	3.02E-14	1.21	1.15	1.27	9.43E-15	1.2076	9.43E-15	1.2076	0.6731	0
5	53268442	rs7722242	T	0.2937	1.145	0.8896	1.472	7.09E-16	1.21	1.16	1.27	7.19E-16	1.2078	7.19E-16	1.2078	0.6727	0
5	53268642	rs13175197	A	0.2915	1.145	0.8902	1.473	1.14E-14	1.21	1.15	1.27	7.39E-15	1.2076	7.39E-15	1.2076	0.673	0
5	53274450	rs10940307	T	0.3371	1.131	0.8795	1.455	9.96E-15	1.21	1.15	1.27	8.52E-15	1.2071	8.52E-15	1.2071	0.6056	0
5	53275193	rs454737	T	0.3333	1.15	0.8663	1.527	6.20E-17	1.25	1.19	1.32	7.94E-17	1.2465	7.94E-17	1.2465	0.5708	0
5	53277462	rs37809	A	0.3721	1.134	0.8606	1.494	3.26E-13	1.21	1.15	1.27	1.68E-13	1.2074	1.68E-13	1.2074	0.6501	0
5	53277913	rs37807	A	0.2428	1.184	0.892	1.571	9.28E-17	1.25	1.19	1.32	4.47E-17	1.2477	4.47E-17	1.2477	0.7119	0
5	53282178	rs454780	T	0.2803	0.8591	0.6521	1.132	1.17E-15	0.82	0.78	0.86	9.28E-16	0.8212	9.28E-16	0.8212	0.7444	0
5	53283161	rs435548	A	0.2428	1.184	0.892	1.571	4.32E-17	1.25	1.19	1.32	4.47E-17	1.2477	4.47E-17	1.2477	0.7119	0
5	53283382	rs565253	T	0.2428	0.8448	0.6366	1.121	3.81E-17	0.8	0.76	0.84	4.48E-17	0.8015	4.48E-17	0.8015	0.7106	0
5	53284613	rs10940308	T	0.1431	0.7978	0.5896	1.079	9.65E-16	0.79	0.75	0.84	3.64E-16	0.7903	3.64E-16	0.7903	0.9501	0
5	53285299	rs251519	T	0.2428	0.8448	0.6366	1.121	2.62E-17	0.8	0.76	0.84	4.48E-17	0.8015	4.48E-17	0.8015	0.7106	0
5	53285349	rs12515400	T	0.2657	1.159	0.8939	1.502	3.16E-18	1.24	1.18	1.3	3.43E-18	1.2371	3.43E-18	1.2371	0.6161	0
5	53285842	rs251520	T	0.2428	0.8448	0.6366	1.121	7.96E-15	0.8	0.76	0.85	1.08E-15	0.8016	1.08E-15	0.8016	0.7111	0
5	53285980	rs13171110	A	0.2118	1.174	0.9125	1.512	4.19E-15	1.21	1.16	1.27	5.55E-15	1.2087	5.55E-15	1.2087	0.8179	0
5	53287101	rs4865762	A	0.2118	1.174	0.9125	1.512	4.15E-15	1.21	1.16	1.27	5.55E-15	1.2087	5.55E-15	1.2087	0.8179	0
5	53287330	rs251522	A	0.2428	1.184	0.892	1.571	3.48E-17	1.25	1.19	1.32	4.47E-17	1.2477	4.47E-17	1.2477	0.7119	0
5	53289681	rs251525	A	0.197	1.205	0.9077	1.6	1.36E-17	1.26	1.19	1.32	2.21E-18	1.2581	2.21E-18	1.2581	0.7613	0
5	53295506	rs3910971	A	0.2351	1.164	0.9057	1.497	1.15E-14	1.21	1.15	1.27	6.08E-15	1.2083	6.08E-15	1.2083	0.7664	0
5	53296267	rs40230	T	0.1854	0.8263	0.623	1.096	2.26E-15	0.8	0.76	0.85	5.33E-16	0.8009	5.33E-16	0.8009	0.8256	0
5	53300560	rs4865763	A	0.2396	0.86	0.6689	1.106	5.90E-15	0.82	0.79	0.87	4.98E-16	0.8214	4.98E-16	0.8214	0.7153	0
5	53301705	rs27967	T	0.1854	1.21	0.9125	1.605	7.48E-18	1.26	1.19	1.33	2.10E-18	1.2583	2.10E-18	1.2583	0.7823	0
5	53307111	rs7737693	T	0.2316	1.166	0.9067	1.499	1.39E-17	1.22	1.17	1.28	2.49E-17	1.2182	2.49E-17	1.2182	0.7284	0
5	53307130	rs7737805	C	0.2316	0.8578	0.6673	1.103	1.69E-17	0.82	0.78	0.86	2.88E-17	0.8212	2.88E-17	0.8212	0.7296	0

5	53308163	rs67239762	T	0.2314	1.166	0.9067	1.5	3.88E-14	1.21	1.15	1.27	5.98E-15	1.2084	5.98E-15	1.2084	0.777	0
5	53308971	rs252042	T	0.227	1.189	0.898	1.574	8.52E-18	1.26	1.19	1.33	2.53E-18	1.2575	2.53E-18	1.2575	0.6905	0
5	53309142	rs252041	A	0.1801	0.8243	0.6214	1.093	7.09E-17	0.8	0.76	0.84	2.63E-17	0.8008	2.63E-17	0.8008	0.8382	0
5	53309358	rs252040	A	0.227	1.189	0.898	1.574	5.93E-17	1.25	1.19	1.32	3.24E-17	1.2479	3.24E-17	1.2479	0.7313	0
5	53309832	rs252038	A	0.1854	0.8263	0.623	1.096	8.49E-18	0.8	0.75	0.84	2.70E-17	0.8009	2.70E-17	0.8009	0.8253	0
5	53312130	rs252035	T	0.1854	0.8263	0.623	1.096	7.79E-18	0.79	0.75	0.84	4.58E-19	0.7912	4.58E-19	0.7912	0.7592	0
5	53313889	rs372044	T	0.3991	0.8897	0.6782	1.167	1.08E-13	0.83	0.78	0.87	4.95E-13	0.832	4.95E-13	0.832	0.622	0
5	53314938	rs4865764	A	0.2354	1.164	0.9056	1.497	1.32E-16	1.22	1.16	1.27	2.55E-17	1.2181	2.55E-17	1.2181	0.7187	0
5	53317694	rs384866	C	0.227	0.8411	0.6353	1.114	1.09E-17	0.8	0.76	0.84	3.25E-17	0.8013	3.25E-17	0.8013	0.7309	0
5	53317725	rs408393	A	0.227	0.8411	0.6353	1.114	3.40E-17	0.8	0.76	0.84	3.25E-17	0.8013	3.25E-17	0.8013	0.7309	0
5	53318961	rs252034	A	0.1854	0.8263	0.623	1.096	1.18E-15	0.8	0.76	0.84	5.33E-16	0.8009	5.33E-16	0.8009	0.8256	0
5	53324826	rs370753	A	0.2038	0.8322	0.6269	1.105	5.82E-15	0.8	0.76	0.85	7.28E-16	0.8011	7.28E-16	0.8011	0.7886	0
5	53325450	rs1363920	A	0.1997	0.8474	0.6579	1.091	1.15E-16	0.82	0.78	0.86	2.51E-17	0.8209	2.51E-17	0.8209	0.8023	0
5	53327934	rs626726	A	0.1939	0.8287	0.6241	1.1	1.58E-15	0.8	0.76	0.85	5.53E-16	0.801	5.53E-16	0.801	0.811	0
5	53330023	rs1363919	T	0.2211	0.8541	0.6635	1.1	1.81E-17	0.82	0.78	0.86	2.75E-17	0.8211	2.75E-17	0.8211	0.7559	0
5	53330398	rs584593	A	0.2431	1.183	0.8923	1.567	8.18E-16	1.25	1.19	1.32	6.79E-16	1.2475	6.79E-16	1.2475	0.7067	0
5	53330469	rs574790	A	0.2486	0.8473	0.6394	1.123	4.10E-15	0.8	0.76	0.85	8.77E-16	0.8017	8.77E-16	0.8017	0.6946	0
5	53332300	rs37776	T	0.2249	1.191	0.8981	1.58	1.07E-14	1.24	1.18	1.31	7.67E-15	1.2382	7.67E-15	1.2382	0.7836	0
5	53332520	rs37777	T	0.2289	0.8418	0.6358	1.114	7.66E-16	0.8	0.76	0.84	6.46E-16	0.8015	6.46E-16	0.8015	0.727	0
5	53333400	rs37778	A	0.2431	1.183	0.8923	1.567	1.23E-15	1.25	1.18	1.32	6.79E-16	1.2475	6.79E-16	1.2475	0.7067	0
5	53335237	rs629725	T	0.2216	0.8375	0.6302	1.113	1.71E-15	0.8	0.76	0.85	6.22E-16	0.8013	6.22E-16	0.8013	0.7565	0
5	53335677	rs1423422	A	0.3252	0.8815	0.6856	1.133	3.53E-11	0.85	0.81	0.89	2.99E-11	0.8511	2.99E-11	0.8511	0.7805	0
5	121933803	rs2914598	A	0.6734	0.9454	0.7282	1.227	1.61E-11	0.84	0.79	0.88	5.37E-11	0.8438	5.37E-11	0.8438	0.384	0
5	121943558	rs12153375	A	0.7415	1.045	0.8047	1.357	7.60E-11	1.18	1.12	1.24	7.27E-11	1.1751	7.27E-11	1.1751	0.3705	0
5	121990502	rs2972344	T	0.7523	0.9587	0.7376	1.246	1.95E-10	0.85	0.8	0.89	1.06E-09	0.8538	1.06E-09	0.8538	0.3772	0
5	122028215	rs17352041	T	0.8773	1.029	0.7149	1.481	1.91E-08	1.22	1.14	1.31	2.91E-08	1.2128	2.91E-08	1.2128	0.3683	0
5	122071524	rs10519694	T	0.9583	1.009	0.7269	1.4	3.71E-10	0.83	0.79	0.88	3.46E-10	0.8348	0.02139	0.8538	0.2499	24.46
5	122078979	rs7711879	T	0.867	1.028	0.7455	1.417	1.70E-11	1.21	1.14	1.28	2.63E-11	1.2043	2.63E-11	1.2043	0.3268	0
5	122079902	rs41383946	C	0.6572	1.075	0.7803	1.482	3.14E-12	1.22	1.15	1.29	5.17E-12	1.2154	5.17E-12	1.2154	0.4462	0
5	122081099	rs41490044	A	0.7445	0.9482	0.6887	1.306	1.01E-12	0.81	0.77	0.86	3.15E-13	0.8138	3.15E-13	0.8138	0.3418	0
5	122081164	rs41424048	T	0.7445	1.055	0.766	1.452	6.71E-13	1.23	1.16	1.3	8.02E-13	1.2244	8.02E-13	1.2244	0.3543	0
5	122081768	rs840464	T	0.6124	1.072	0.8202	1.4	1.72E-20	1.26	1.2	1.33	5.81E-20	1.2533	0.0004408	1.2287	0.244	26.33
5	122081992	rs3853401	A	0.7609	0.9516	0.6911	1.31	4.60E-13	0.81	0.77	0.86	5.71E-13	0.814	5.71E-13	0.814	0.3308	0
5	122082156	rs62382962	A	0.7609	1.051	0.7633	1.447	4.48E-13	1.23	1.17	1.31	1.43E-12	1.2241	1.43E-12	1.2241	0.3424	0
5	122082732	rs17431081	A	0.8572	1.03	0.7444	1.426	4.14E-11	1.21	1.15	1.28	1.05E-10	1.2042	1.05E-10	1.2042	0.3387	0
5	122082850	rs17352686	A	0.8566	1.03	0.7445	1.426	1.66E-11	1.22	1.15	1.29	2.60E-11	1.2137	7.88E-10	1.2127	0.3148	1.05

5	122082908	rs72786962	A	0.8746	0.9742	0.7039	1.348	3.62E-11	0.82	0.78	0.87	3.98E-11	0.8244	7.19E-07	0.8274	0.3063	4.45
5	122082951	rs840462	A	0.5667	0.925	0.7083	1.208	2.08E-17	0.8	0.76	0.85	5.71E-18	0.804	3.73E-08	0.8089	0.2945	8.99
5	122083564	rs840461	T	0.6274	1.069	0.8176	1.397	4.24E-20	1.26	1.2	1.32	6.00E-20	1.2533	0.0008749	1.226	0.2366	28.62
5	122084731	rs2731647	A	0.5338	0.9206	0.7093	1.195	1.51E-16	0.81	0.78	0.86	3.38E-17	0.8135	3.38E-17	0.8135	0.3442	0
5	122085224	rs2731646	A	0.4899	0.9102	0.6969	1.189	6.56E-19	0.8	0.76	0.84	2.58E-18	0.8035	2.58E-18	0.8035	0.3517	0
5	122085251	rs72786964	T	0.8396	1.034	0.7471	1.432	7.47E-10	1.2	1.13	1.27	1.22E-09	1.1945	1.22E-09	1.1945	0.377	0
5	122086865	rs995687	A	0.7338	1.057	0.7675	1.456	1.09E-10	1.21	1.14	1.28	8.82E-11	1.2049	8.82E-11	1.2049	0.4151	0
5	122087230	rs2731657	A	0.6411	0.9385	0.7186	1.226	1.98E-20	0.79	0.75	0.83	2.34E-20	0.7946	0.003326	0.8174	0.2137	35.32
6	39586825	rs10947821	T	0.07027	1.332	0.9766	1.817	1.30E-07	1.18	1.11	1.26	3.34E-08	1.1854	3.34E-08	1.1854	0.453	0
6	39588364	rs6931536	T	0.07027	1.332	0.9766	1.817	2.48E-07	1.18	1.11	1.25	3.66E-08	1.1855	3.66E-08	1.1855	0.4531	0
6	50821065	rs3798519	A	0.8119	0.9611	0.6929	1.333	3.99E-10	0.83	0.79	0.88	1.88E-10	0.8336	1.88E-10	0.8336	0.3866	0
6	50823927	rs2076308	C	0.8972	1.022	0.7371	1.416	4.46E-09	1.18	1.12	1.25	9.12E-09	1.1752	9.12E-09	1.1752	0.395	0
6	50829192	rs62405422	T	0.7877	0.9561	0.6895	1.326	8.83E-10	0.84	0.79	0.89	2.70E-09	0.8432	2.70E-09	0.8432	0.4445	0
6	50830813	rs2206277	T	0.8072	1.042	0.7511	1.444	1.33E-09	1.19	1.13	1.26	2.65E-09	1.1854	2.65E-09	1.1854	0.4328	0
6	50835337	rs987237	A	0.7572	0.9498	0.6851	1.317	3.73E-09	0.84	0.79	0.89	3.77E-09	0.8431	3.77E-09	0.8431	0.468	0
6	50841565	rs4715207	T	0.7877	1.046	0.7543	1.45	7.56E-10	1.2	1.13	1.27	5.76E-10	1.1951	5.76E-10	1.1951	0.4173	0
6	50842074	rs3798526	A	0.7877	0.9561	0.6895	1.326	6.67E-10	0.83	0.79	0.88	2.42E-10	0.8335	2.42E-10	0.8335	0.4036	0
6	50846016	rs62405437	T	0.7616	0.9506	0.6853	1.319	1.41E-09	0.84	0.79	0.89	3.35E-09	0.8431	3.35E-09	0.8431	0.4657	0
6	50847601	rs62405438	A	0.7616	1.052	0.7584	1.459	1.20E-09	1.19	1.13	1.27	3.63E-09	1.1856	3.63E-09	1.1856	0.4672	0
6	50849174	rs72892910	T	0.635	1.084	0.7761	1.515	2.67E-09	1.19	1.13	1.26	3.84E-09	1.1868	3.84E-09	1.1868	0.5902	0
6	50865371	rs4711961	A	0.6305	1.087	0.7743	1.525	3.86E-09	1.19	1.12	1.26	5.40E-09	1.1869	5.40E-09	1.1869	0.6059	0
6	50868566	rs734597	A	0.6305	1.087	0.7743	1.525	2.15E-09	1.2	1.13	1.27	9.89E-10	1.1966	9.89E-10	1.1966	0.573	0
6	50869063	rs1075847	A	0.6305	0.9202	0.6556	1.291	2.15E-09	0.84	0.79	0.89	4.99E-09	0.8422	4.99E-09	0.8422	0.6032	0
6	50872229	rs72889996	T	0.1465	1.578	0.8526	2.92	3.20E-08	1.39	1.24	1.56	1.27E-08	1.3962	1.27E-08	1.3962	0.6915	0
6	50877777	rs2207139	A	0.641	0.9225	0.6572	1.295	2.34E-09	0.84	0.79	0.89	5.07E-09	0.8423	5.07E-09	0.8423	0.5936	0
6	50879773	rs62405439	A	0.641	1.084	0.7723	1.521	1.89E-09	1.2	1.13	1.27	1.01E-09	1.1965	1.01E-09	1.1965	0.5625	0
6	50890522	rs72891717	A	0.65	1.082	0.7706	1.518	1.34E-09	1.2	1.13	1.27	8.94E-10	1.1964	8.94E-10	1.1964	0.5554	0
6	50896622	rs62405443	A	0.641	0.9225	0.6572	1.295	1.56E-09	0.84	0.79	0.89	5.07E-09	0.8423	5.07E-09	0.8423	0.5936	0
6	50898107	rs943005	T	0.641	1.084	0.7723	1.521	1.57E-09	1.2	1.13	1.27	1.01E-09	1.1965	1.01E-09	1.1965	0.5625	0
6	50903450	rs72891726	A	0.1465	0.6338	0.3425	1.173	5.67E-08	0.72	0.64	0.81	1.23E-08	0.7168	1.23E-08	0.7168	0.6899	0
6	50905488	rs62407770	A	0.641	0.9225	0.6572	1.295	1.90E-09	0.84	0.79	0.89	5.07E-09	0.8423	5.07E-09	0.8423	0.5936	0
6	50911829	rs72887104	A	0.1465	0.6338	0.3425	1.173	4.22E-08	0.72	0.64	0.81	1.37E-08	0.7168	1.37E-08	0.7168	0.6899	0
6	50912891	rs62407774	T	0.641	0.9225	0.6572	1.295	1.46E-09	0.84	0.79	0.89	4.50E-09	0.8423	4.50E-09	0.8423	0.5935	0
6	50922371	rs62407779	T	0.641	1.084	0.7723	1.521	1.36E-09	1.2	1.13	1.27	8.84E-10	1.1965	8.84E-10	1.1965	0.5625	0
6	50925730	rs12524834	T	0.641	1.084	0.7723	1.521	8.56E-10	1.2	1.13	1.27	8.84E-10	1.1965	8.84E-10	1.1965	0.5625	0
6	50925939	rs77891659	A	0.641	1.084	0.7723	1.521	7.71E-10	1.2	1.13	1.27	8.84E-10	1.1965	8.84E-10	1.1965	0.5625	0

6	50928917	rs12529728	A	0.641	0.9225	0.6572	1.295	8.67E-10	0.83	0.79	0.88	3.80E-10	0.8325	3.80E-10	0.8325	0.5472	0
6	50929538	rs4715210	T	0.641	1.084	0.7723	1.521	8.39E-10	1.2	1.13	1.27	8.84E-10	1.1965	8.84E-10	1.1965	0.5625	0
6	50934988	rs72887130	A	0.641	0.9225	0.6572	1.295	1.01E-09	0.83	0.79	0.88	3.80E-10	0.8325	3.80E-10	0.8325	0.5472	0
6	50937168	rs16880854	A	0.641	1.084	0.7723	1.521	1.00E-09	1.2	1.13	1.27	8.84E-10	1.1965	8.84E-10	1.1965	0.5625	0
6	50937354	rs3857596	T	0.641	1.084	0.7723	1.521	1.00E-09	1.2	1.13	1.27	8.84E-10	1.1965	8.84E-10	1.1965	0.5625	0
6	50937937	rs6925404	C	0.641	0.9225	0.6572	1.295	1.00E-09	0.83	0.79	0.88	3.80E-10	0.8325	3.80E-10	0.8325	0.5472	0
6	50941279	rs16880869	T	0.641	0.9225	0.6572	1.295	1.07E-09	0.83	0.79	0.88	3.80E-10	0.8325	3.80E-10	0.8325	0.5472	0
6	50942569	rs62407801	T	0.641	0.9225	0.6572	1.295	1.58E-09	0.84	0.79	0.89	4.50E-09	0.8423	4.50E-09	0.8423	0.5935	0
6	50943378	rs4715213	T	0.641	1.084	0.7723	1.521	1.45E-09	1.2	1.13	1.27	1.01E-09	1.1965	1.01E-09	1.1965	0.5625	0
6	50946635	rs72887147	T	0.641	1.084	0.7723	1.521	1.39E-09	1.2	1.13	1.27	1.01E-09	1.1965	1.01E-09	1.1965	0.5625	0
6	50948976	rs72899088	T	0.1465	1.578	0.8526	2.92	2.91E-08	1.37	1.23	1.54	1.39E-08	1.3762	1.39E-08	1.3762	0.6579	0
6	50953889	rs6904450	A	0.7829	0.954	0.6825	1.334	6.44E-11	0.83	0.78	0.88	1.76E-10	0.8332	1.76E-10	0.8332	0.4218	0
6	50963135	rs79541760	A	0.5658	0.9054	0.6448	1.271	1.06E-09	0.83	0.79	0.88	4.42E-10	0.8321	4.42E-10	0.8321	0.6206	0
6	50967800	rs1928185	T	0.641	0.9225	0.6572	1.295	2.62E-09	0.84	0.79	0.89	6.40E-09	0.8423	6.40E-09	0.8423	0.5936	0
6	50969699	rs4711965	A	0.641	1.084	0.7723	1.521	2.22E-09	1.2	1.13	1.27	1.14E-09	1.1965	1.14E-09	1.1965	0.5625	0
6	50970534	rs3857599	A	0.6637	1.079	0.766	1.52	8.92E-09	1.19	1.12	1.26	1.12E-08	1.1866	1.12E-08	1.1866	0.581	0
6	51742973	rs9382005	C	0.955	1.008	0.7726	1.314	2.31E-09	0.86	0.82	0.9	4.94E-09	0.8646	0.02429	0.8805	0.2493	24.65
6	74784722	rs6924936	T	0.01092	0.615	0.423	0.8942	1.50E-07	0.8	0.74	0.87	7.35E-09	0.7905	0.01114	0.7493	0.1785	44.75
6	74789370	rs148628698	C	0.01092	0.615	0.423	0.8942	2.23E-07	0.81	0.74	0.87	4.53E-08	0.7999	0.01975	0.7517	0.159	49.6
6	74790178	rs111977209	A	0.01646	0.6581	0.4676	0.9263	2.54E-07	0.84	0.79	0.9	1.45E-08	0.833	0.02609	0.7896	0.1691	47.13
6	74807378	rs75587050	A	0.01712	0.6309	0.4321	0.9213	9.32E-08	0.8	0.74	0.87	1.13E-08	0.7915	0.003757	0.7657	0.2297	30.69
6	74835688	rs139385675	T	0.001075	2.639	1.475	4.722	9.06E-07	1.58	1.32	1.9	1.66E-08	1.655	0.00924	1.89	0.0993	63.19
6	74895729	rs117624271	T	0.002687	2.331	1.341	4.051	1.18E-06	1.5	1.27	1.76	3.42E-08	1.554	0.00785	1.7221	0.1338	55.51
6	74932159	rs118040907	T	0.005187	2.233	1.271	3.921	5.52E-07	1.52	1.29	1.8	2.56E-08	1.5667	0.001929	1.6701	0.1988	39.44
6	75061719	rs118043261	A	0.0005652	2.462	1.475	4.11	1.35E-14	1.72	1.5	1.98	8.46E-17	1.7623	4.95E-05	1.8839	0.1853	43.01
6	75067044	rs117693620	T	0.0003411	2.514	1.518	4.162	1.86E-14	1.71	1.49	1.97	1.08E-16	1.7565	0.0002146	1.915	0.1485	52.09
6	88852991	rs1321085	A	0.1547	1.288	0.9088	1.826	1.00E-07	1.17	1.1	1.24	4.61E-08	1.173	4.61E-08	1.173	0.5944	0
8	23524256	rs73228208	A	0.0532	0.6929	0.4776	1.005	7.01E-08	0.85	0.8	0.9	1.43E-08	0.8458	0.001214	0.8363	0.2875	11.59
8	94820220	rs9650133	T	0.426	0.9002	0.6948	1.166	2.48E-08	0.87	0.83	0.91	1.67E-08	0.871	1.67E-08	0.871	0.7996	0
8	94820983	rs10096090	T	0.4411	0.9034	0.6975	1.17	1.87E-08	0.87	0.83	0.91	1.71E-08	0.8711	1.71E-08	0.8711	0.7791	0
8	94824584	rs4500055	A	0.4136	1.114	0.8601	1.443	1.40E-08	1.15	1.1	1.21	1.45E-08	1.1487	1.45E-08	1.1487	0.8127	0
8	94841480	rs11996455	A	0.3612	0.8864	0.6844	1.148	3.14E-08	0.88	0.84	0.92	4.49E-08	0.8802	4.49E-08	0.8802	0.9569	0
8	94841679	rs13271375	A	0.4156	1.113	0.8598	1.442	3.54E-08	1.14	1.09	1.2	3.04E-08	1.1391	3.04E-08	1.1391	0.8581	0
8	94850469	rs3779789	A	0.4006	1.117	0.8627	1.447	2.55E-08	1.14	1.09	1.2	2.96E-08	1.1393	2.96E-08	1.1393	0.8791	0
8	94863448	rs6991067	A	0.3451	1.132	0.8747	1.466	3.67E-08	1.14	1.09	1.19	2.34E-08	1.1397	2.34E-08	1.1397	0.958	0
8	94870137	rs4734285	T	0.1324	1.23	0.9393	1.61	3.71E-08	1.16	1.1	1.22	5.02E-09	1.1624	5.02E-09	1.1624	0.6755	0

8	94895839	rs34208552	C	0.302	1.151	0.8811	1.504	5.27E-09	1.15	1.1	1.21	5.89E-09	1.15	5.89E-09	1.15	0.995	0
8	94895850	rs34293080	T	0.302	0.8686	0.6648	1.135	2.91E-09	0.87	0.82	0.91	6.63E-09	0.87	6.63E-09	0.87	0.9907	0
8	94896311	rs4735329	T	0.3267	0.8748	0.6695	1.143	5.81E-09	0.87	0.83	0.91	7.00E-09	0.8701	7.00E-09	0.8701	0.9683	0
8	94899249	rs12546365	T	0.5638	1.08	0.8313	1.403	2.63E-09	1.15	1.1	1.21	3.48E-09	1.1478	3.48E-09	1.1478	0.6435	0
8	94901875	rs13254283	T	0.5752	0.928	0.7145	1.205	8.61E-09	0.87	0.83	0.91	4.02E-09	0.8717	4.02E-09	0.8717	0.6338	0
8	94904583	rs2340584	A	0.5391	1.085	0.8357	1.409	7.53E-09	1.15	1.09	1.2	3.36E-09	1.148	3.36E-09	1.148	0.6674	0
8	94904860	rs4570121	T	0.3392	0.8776	0.6715	1.147	1.65E-09	0.86	0.82	0.91	4.03E-10	0.8605	4.03E-10	0.8605	0.8839	0
8	94905048	rs9643350	A	0.3392	0.8776	0.6715	1.147	2.01E-09	0.86	0.82	0.91	4.03E-10	0.8605	4.03E-10	0.8605	0.8839	0
8	94905062	rs9643351	T	0.3392	0.8776	0.6715	1.147	2.46E-09	0.86	0.82	0.91	4.03E-10	0.8605	4.03E-10	0.8605	0.8839	0
8	94905542	rs4735330	A	0.3392	1.139	0.8719	1.489	1.52E-09	1.16	1.1	1.22	7.48E-10	1.1593	7.48E-10	1.1593	0.8953	0
8	94905730	rs4735331	T	0.3392	0.8776	0.6715	1.147	7.22E-10	0.86	0.82	0.9	4.03E-10	0.8605	4.03E-10	0.8605	0.8839	0
8	94906060	rs9643352	T	0.3392	0.8776	0.6715	1.147	1.52E-09	0.86	0.82	0.91	4.03E-10	0.8605	4.03E-10	0.8605	0.8839	0
8	94907003	rs7820818	A	0.4389	0.8982	0.6845	1.179	4.06E-10	0.85	0.81	0.9	1.73E-10	0.8515	1.73E-10	0.8515	0.6955	0
8	94907558	rs10956930	C	0.3392	1.139	0.8719	1.489	1.36E-09	1.16	1.11	1.22	7.48E-10	1.1593	7.48E-10	1.1593	0.8953	0
8	94907965	rs7015841	A	0.2873	1.156	0.8851	1.51	6.60E-10	1.16	1.11	1.22	6.61E-10	1.1599	6.61E-10	1.1599	0.9801	0
8	94908862	rs7001535	C	0.2873	1.156	0.8851	1.51	1.39E-09	1.16	1.11	1.22	6.61E-10	1.1599	6.61E-10	1.1599	0.9801	0
8	94909067	rs6987400	T	0.2873	1.156	0.8851	1.51	1.39E-09	1.16	1.11	1.22	6.61E-10	1.1599	6.61E-10	1.1599	0.9801	0
8	94910263	rs6471501	A	0.3767	1.13	0.8618	1.481	3.87E-10	1.17	1.12	1.23	5.96E-10	1.1687	5.96E-10	1.1687	0.8045	0
8	94910387	rs7016078	A	0.2873	0.8651	0.6624	1.13	6.12E-10	0.86	0.82	0.9	3.56E-10	0.8602	3.56E-10	0.8602	0.9659	0
8	94910524	rs7017487	T	0.2873	0.8651	0.6624	1.13	7.34E-10	0.86	0.82	0.9	3.56E-10	0.8602	3.56E-10	0.8602	0.9659	0
8	94911432	rs4735332	T	0.2873	0.8651	0.6624	1.13	7.47E-10	0.86	0.82	0.9	3.56E-10	0.8602	3.56E-10	0.8602	0.9659	0
8	94914662	rs13262110	T	0.4778	0.9097	0.7004	1.181	7.13E-09	0.87	0.83	0.91	3.43E-09	0.8712	3.43E-09	0.8712	0.7419	0
8	94916974	rs12548367	T	0.2716	0.8609	0.6591	1.124	1.18E-08	0.87	0.83	0.91	6.18E-09	0.8697	6.18E-09	0.8697	0.9395	0
8	94917615	rs12548999	T	0.2716	0.8609	0.6591	1.124	9.73E-09	0.87	0.83	0.91	6.18E-09	0.8697	6.18E-09	0.8697	0.9395	0
8	94918073	rs10956931	T	0.3576	1.136	0.8661	1.489	1.94E-09	1.17	1.11	1.23	5.71E-10	1.1689	5.71E-10	1.1689	0.8339	0
8	94919554	rs2123647	A	0.2716	0.8609	0.6591	1.124	6.35E-09	0.87	0.83	0.91	6.18E-09	0.8697	6.18E-09	0.8697	0.9395	0
8	94922936	rs12548874	A	0.5006	0.915	0.7065	1.185	1.18E-08	0.87	0.83	0.91	2.46E-08	0.8715	2.46E-08	0.8715	0.7072	0
8	94925274	rs7845219	T	0.4703	1.101	0.8474	1.432	2.65E-09	1.15	1.1	1.21	2.99E-09	1.1485	2.99E-09	1.1485	0.7486	0
8	94927366	rs2945554	A	0.2886	0.8657	0.6632	1.13	1.62E-09	0.86	0.82	0.91	3.58E-10	0.8602	3.58E-10	0.8602	0.9618	0
8	94933775	rs2340583	A	0.3486	0.8784	0.6699	1.152	1.56E-09	0.86	0.81	0.9	2.83E-09	0.8606	2.83E-09	0.8606	0.8804	0
8	94936785	rs3019175	A	0.2442	0.8533	0.6533	1.114	9.25E-08	0.87	0.83	0.92	2.10E-08	0.8694	2.10E-08	0.8694	0.8888	0
8	94940249	rs2956210	A	0.6971	1.06	0.7919	1.418	5.46E-08	1.15	1.09	1.2	2.43E-08	1.1474	2.43E-08	1.1474	0.5884	0
8	94942846	rs4735333	A	0.3818	0.8888	0.6825	1.158	6.84E-08	0.87	0.83	0.92	1.53E-08	0.8706	1.53E-08	0.8706	0.8761	0
8	94943517	rs11784717	T	0.6971	1.06	0.7919	1.418	4.53E-08	1.15	1.09	1.2	2.43E-08	1.1474	2.43E-08	1.1474	0.5884	0
8	94957029	rs4582532	A	0.5238	0.9181	0.706	1.194	5.97E-09	0.87	0.83	0.91	3.18E-09	0.8714	3.18E-09	0.8714	0.6925	0
8	94957033	rs2340586	A	0.5238	0.9181	0.706	1.194	6.47E-09	0.87	0.83	0.91	3.18E-09	0.8714	3.18E-09	0.8714	0.6925	0

8	94957094	rs10429294	T	0.557	0.9242	0.7106	1.202	2.49E-09	0.87	0.83	0.91	2.88E-09	0.8716	2.88E-09	0.8716	0.6571	0
8	94957217	rs9297949	A	0.557	1.082	0.8318	1.407	3.21E-09	1.15	1.1	1.2	2.53E-09	1.1479	2.53E-09	1.1479	0.6544	0
8	94957719	rs1453377	T	0.557	0.9242	0.7106	1.202	3.16E-09	0.87	0.83	0.91	2.88E-09	0.8716	2.88E-09	0.8716	0.6571	0
8	94958011	rs1453379	T	0.4852	1.103	0.8375	1.453	3.32E-10	1.17	1.11	1.23	2.08E-10	1.1679	2.08E-10	1.1679	0.6794	0
8	94958936	rs10429443	A	0.5727	1.078	0.8295	1.402	2.95E-09	1.15	1.1	1.2	2.61E-09	1.1478	2.61E-09	1.1478	0.6344	0
8	94959692	rs2011567	A	0.5727	0.9272	0.7132	1.206	3.07E-09	0.87	0.83	0.91	2.95E-09	0.8717	2.95E-09	0.8717	0.6395	0
8	94960225	rs896846	A	0.5727	0.9272	0.7132	1.206	3.23E-09	0.87	0.83	0.91	2.95E-09	0.8717	2.95E-09	0.8717	0.6395	0
8	94960323	rs896847	A	0.5727	0.9272	0.7132	1.206	2.81E-09	0.87	0.83	0.91	2.95E-09	0.8717	2.95E-09	0.8717	0.6395	0
8	94960510	rs13281625	T	0.5727	1.078	0.8295	1.402	3.23E-09	1.15	1.1	1.2	2.61E-09	1.1478	2.61E-09	1.1478	0.6344	0
8	94960591	rs13281709	A	0.5727	0.9272	0.7132	1.206	3.22E-09	0.87	0.83	0.91	2.95E-09	0.8717	2.95E-09	0.8717	0.6395	0
8	94960746	rs13278601	C	0.5661	0.926	0.7121	1.204	2.45E-09	0.87	0.83	0.91	2.92E-09	0.8716	2.92E-09	0.8716	0.6466	0
8	94961099	rs4735335	C	0.5727	0.9272	0.7132	1.206	3.16E-09	0.87	0.83	0.91	2.95E-09	0.8717	2.95E-09	0.8717	0.6395	0
8	94961237	rs4735337	T	0.5727	1.078	0.8295	1.402	3.17E-09	1.15	1.1	1.2	2.61E-09	1.1478	2.61E-09	1.1478	0.6344	0
8	94961376	rs16893776	T	0.5727	0.9272	0.7132	1.206	3.15E-09	0.87	0.83	0.91	2.95E-09	0.8717	2.95E-09	0.8717	0.6395	0
8	94961409	rs28591375	C	0.5727	1.078	0.8295	1.402	3.18E-09	1.15	1.1	1.2	2.61E-09	1.1478	2.61E-09	1.1478	0.6344	0
8	94961453	rs28560301	A	0.5727	0.9272	0.7132	1.206	2.95E-09	0.87	0.83	0.91	2.95E-09	0.8717	2.95E-09	0.8717	0.6395	0
8	94961658	rs4735338	T	0.5727	1.078	0.8295	1.402	3.18E-09	1.15	1.1	1.2	2.61E-09	1.1478	2.61E-09	1.1478	0.6344	0
8	94962145	rs4735339	T	0.5727	0.9272	0.7132	1.206	4.31E-09	0.87	0.83	0.91	1.25E-08	0.8718	1.25E-08	0.8718	0.6399	0
8	94962257	rs28716845	A	0.5727	1.078	0.8295	1.402	4.31E-09	1.15	1.1	1.21	1.11E-08	1.1476	1.11E-08	1.1476	0.6348	0
8	94962570	rs7815762	A	0.5727	1.078	0.8295	1.402	4.43E-09	1.15	1.1	1.21	1.11E-08	1.1476	1.11E-08	1.1476	0.6348	0
8	94962733	rs7823059	T	0.5727	1.078	0.8295	1.402	5.42E-09	1.15	1.1	1.21	1.11E-08	1.1476	1.11E-08	1.1476	0.6348	0
8	94962852	rs7835379	A	0.4274	0.8941	0.6782	1.179	1.22E-09	0.85	0.81	0.9	2.31E-10	0.8514	2.31E-10	0.8514	0.7242	0
8	94962931	rs7823366	T	0.4203	1.121	0.8496	1.478	1.75E-09	1.17	1.11	1.23	8.69E-10	1.1684	8.69E-10	1.1684	0.7657	0
8	94962940	rs7819800	A	0.5727	1.078	0.8295	1.402	3.95E-09	1.16	1.1	1.21	1.36E-09	1.1572	1.36E-09	1.1572	0.5902	0
8	94963074	rs13266313	T	0.5727	0.9272	0.7132	1.206	4.35E-09	0.87	0.83	0.91	1.25E-08	0.8718	1.25E-08	0.8718	0.6399	0
8	94963317	rs6993278	T	0.4338	1.117	0.8472	1.472	3.64E-09	1.16	1.11	1.22	6.63E-09	1.1586	6.63E-09	1.1586	0.792	0
8	94964023	rs4735340	A	0.5727	1.078	0.8295	1.402	3.88E-09	1.16	1.1	1.21	1.36E-09	1.1572	1.36E-09	1.1572	0.5902	0
8	94965166	rs1349763	A	0.4338	1.117	0.8472	1.472	5.16E-09	1.16	1.11	1.22	6.63E-09	1.1586	6.63E-09	1.1586	0.792	0
8	94967769	rs4734294	A	0.3894	1.129	0.8562	1.49	2.11E-09	1.17	1.11	1.23	8.22E-10	1.1687	8.22E-10	1.1687	0.8039	0
8	94968480	rs6982393	T	0.5567	1.082	0.8318	1.408	4.92E-09	1.15	1.1	1.21	1.08E-08	1.1477	1.08E-08	1.1477	0.655	0
8	94969050	rs12549003	A	0.4044	1.125	0.853	1.483	4.68E-10	1.17	1.11	1.22	1.77E-10	1.1686	1.77E-10	1.1686	0.7843	0
8	94973548	rs13265518	A	0.3587	0.8761	0.6605	1.162	1.49E-08	0.86	0.82	0.91	4.31E-09	0.8605	4.31E-09	0.8605	0.8992	0
8	94973811	rs10081596	A	0.3983	1.127	0.8542	1.486	3.80E-09	1.16	1.11	1.22	6.21E-09	1.1589	6.21E-09	1.1589	0.8408	0
8	94974112	rs7827478	T	0.3983	1.127	0.8542	1.486	4.11E-09	1.16	1.11	1.22	6.21E-09	1.1589	6.21E-09	1.1589	0.8408	0
8	94974398	rs13274945	T	0.5156	0.9164	0.7044	1.192	3.89E-08	0.87	0.83	0.92	1.70E-08	0.8715	1.70E-08	0.8715	0.7036	0
8	94974699	rs10086284	A	0.3983	1.127	0.8542	1.486	3.09E-09	1.17	1.11	1.23	8.33E-10	1.1686	8.33E-10	1.1686	0.7943	0

8	94975025	rs3802193	T	0.4074	1.127	0.8494	1.495	7.09E-09	1.16	1.11	1.22	9.28E-09	1.1589	9.28E-09	1.1589	0.8439	0
8	94975933	rs2084968	A	0.4035	1.125	0.8532	1.484	1.47E-08	1.16	1.1	1.22	6.29E-09	1.1589	6.29E-09	1.1589	0.831	0
8	94976130	rs7007935	T	0.4375	1.116	0.8461	1.472	1.33E-08	1.16	1.1	1.22	6.68E-09	1.1586	6.68E-09	1.1586	0.7876	0
8	94976153	rs6984240	T	0.6218	0.936	0.7198	1.217	5.07E-09	0.87	0.83	0.91	1.35E-08	0.8721	1.35E-08	0.8721	0.5914	0
8	94980102	rs2340534	A	0.3711	0.8866	0.6811	1.154	7.04E-08	0.88	0.84	0.92	4.57E-08	0.8802	4.57E-08	0.8802	0.9564	0
9	13533301	rs35859888	A	0.1581	1.268	0.912	1.762	3.24E-12	1.24	1.17	1.32	8.88E-13	1.2409	8.88E-13	1.2409	0.896	0
9	13534995	rs36052788	A	0.2333	0.8234	0.5981	1.133	2.15E-15	0.8	0.75	0.84	4.61E-15	0.8007	4.61E-15	0.8007	0.8618	0
9	13535324	rs1324170	A	0.1849	1.207	0.9139	1.594	5.68E-08	1.15	1.09	1.21	1.82E-08	1.1517	1.82E-08	1.1517	0.7374	0
9	13535496	rs1931230	T	0.1849	1.207	0.9139	1.594	4.02E-08	1.15	1.09	1.21	1.82E-08	1.1517	1.82E-08	1.1517	0.7374	0
9	13535632	rs2890970	A	0.1849	1.207	0.9139	1.594	4.72E-08	1.15	1.09	1.21	1.82E-08	1.1517	1.82E-08	1.1517	0.7374	0
9	13535703	rs2382399	T	0.1849	1.207	0.9139	1.594	5.06E-08	1.15	1.09	1.21	1.82E-08	1.1517	1.82E-08	1.1517	0.7374	0
9	13536084	rs12002959	A	0.3188	1.178	0.8537	1.625	1.03E-15	1.26	1.19	1.34	1.05E-15	1.2574	1.05E-15	1.2574	0.6867	0
9	13537769	rs12003602	A	0.1985	1.232	0.8963	1.694	2.02E-15	1.26	1.19	1.34	2.06E-15	1.2591	2.06E-15	1.2591	0.8917	0
9	13543647	rs13296965	T	0.3001	1.184	0.86	1.631	9.49E-17	1.27	1.2	1.34	6.67E-17	1.2673	6.67E-17	1.2673	0.6724	0
9	13544780	rs67878159	A	0.389	0.8683	0.6296	1.197	2.20E-16	0.79	0.75	0.84	2.21E-16	0.7922	2.21E-16	0.7922	0.5703	0
9	13546791	rs12686335	A	0.2696	1.197	0.8699	1.647	9.77E-16	1.27	1.19	1.34	1.97E-16	1.2676	1.97E-16	1.2676	0.7204	0
9	13548061	rs10491754	A	0.3306	1.172	0.8514	1.613	2.46E-14	1.24	1.18	1.31	2.90E-14	1.2379	2.90E-14	1.2379	0.7332	0
9	13549156	rs1324175	T	0.2191	0.8301	0.6168	1.117	2.59E-19	0.78	0.74	0.83	3.32E-20	0.7815	3.32E-20	0.7815	0.6861	0
9	13550070	rs2224860	A	0.273	1.194	0.8696	1.639	2.41E-13	1.23	1.16	1.3	1.72E-13	1.2289	1.72E-13	1.2289	0.8565	0
9	13550542	rs977580	A	0.3807	0.8715	0.6407	1.185	1.03E-15	0.81	0.76	0.85	4.50E-15	0.8117	4.50E-15	0.8117	0.646	0
9	13555075	rs13294011	A	0.4356	1.132	0.8287	1.547	1.06E-16	1.25	1.19	1.32	1.61E-16	1.2465	1.61E-16	1.2465	0.5392	0
9	13555514	rs1556575	A	0.2413	0.8364	0.6204	1.128	3.06E-19	0.78	0.74	0.83	2.66E-20	0.7817	2.66E-20	0.7817	0.652	0
9	13555913	rs11999938	T	0.401	1.142	0.8378	1.556	6.22E-18	1.27	1.2	1.34	4.12E-18	1.266	4.12E-18	1.266	0.5077	0
9	13557421	rs1324182	T	0.2115	0.8275	0.6148	1.114	1.18E-15	0.8	0.76	0.84	5.87E-16	0.8009	5.87E-16	0.8009	0.8265	0
9	13557492	rs1324183	A	0.2115	1.209	0.8979	1.627	9.69E-16	1.25	1.18	1.32	5.85E-16	1.2486	5.85E-16	1.2486	0.8287	0
9	13557505	rs7851523	T	0.3683	1.152	0.8466	1.567	6.83E-15	1.25	1.18	1.32	1.90E-15	1.2468	1.90E-15	1.2468	0.609	0
9	13557522	rs1324184	A	0.2169	1.206	0.8958	1.624	5.81E-16	1.25	1.19	1.32	7.56E-16	1.2485	7.56E-16	1.2485	0.8163	0
9	13557624	rs4481709	A	0.2115	0.8275	0.6148	1.114	1.27E-15	0.8	0.75	0.84	1.82E-15	0.8009	1.82E-15	0.8009	0.8266	0
9	13557666	rs1324185	A	0.2115	0.8275	0.6148	1.114	1.64E-15	0.8	0.76	0.85	4.65E-16	0.8009	4.65E-16	0.8009	0.8264	0
9	13557748	rs7851770	T	0.3359	1.163	0.8553	1.581	5.11E-15	1.25	1.18	1.32	1.39E-15	1.2472	1.39E-15	1.2472	0.6504	0
9	13557887	rs4579618	T	0.1809	0.8176	0.6087	1.098	2.04E-15	0.8	0.76	0.85	4.08E-16	0.8006	4.08E-16	0.8006	0.8869	0
9	13558318	rs1831902	T	0.2115	0.8275	0.6148	1.114	2.12E-15	0.8	0.76	0.85	4.65E-16	0.8009	4.65E-16	0.8009	0.8264	0
9	13558379	rs13291445	A	0.4208	1.136	0.8333	1.548	4.84E-15	1.25	1.18	1.32	2.16E-15	1.2463	2.16E-15	1.2463	0.551	0
9	13558456	rs12686184	A	0.3446	0.8623	0.6341	1.172	8.53E-15	0.8	0.76	0.85	1.43E-15	0.8019	1.43E-15	0.8019	0.6378	0
9	13558523	rs1831904	A	0.2115	0.8275	0.6148	1.114	2.19E-15	0.8	0.76	0.85	4.65E-16	0.8009	4.65E-16	0.8009	0.8264	0
9	13559718	rs66720556	A	0.3592	1.155	0.8489	1.571	6.07E-18	1.27	1.2	1.33	1.59E-18	1.2665	1.59E-18	1.2665	0.5514	0

9	13559822	rs10491756	T	0.3592	1.155	0.8489	1.571	4.86E-17	1.26	1.19	1.33	3.27E-17	1.2567	3.27E-17	1.2567	0.5851	0
9	13562147	rs12004620	A	0.3109	0.8541	0.6296	1.159	2.81E-13	0.81	0.77	0.86	1.60E-13	0.8114	1.60E-13	0.8114	0.7376	0
9	13563514	rs66592246	T	0.3834	1.146	0.8431	1.559	3.73E-14	1.25	1.18	1.32	1.12E-14	1.2464	1.12E-14	1.2464	0.5859	0
9	13563661	rs13290289	T	0.3834	1.146	0.8431	1.559	1.84E-14	1.25	1.18	1.32	7.39E-15	1.2465	7.39E-15	1.2465	0.5858	0
9	13565223	rs34944131	T	0.3876	1.145	0.8425	1.555	1.30E-13	1.24	1.17	1.31	7.62E-14	1.2367	7.62E-14	1.2367	0.616	0
9	13565429	rs147892492	A	0.3407	0.8626	0.6365	1.169	6.51E-13	0.81	0.77	0.86	1.44E-13	0.8117	1.44E-13	0.8117	0.69	0
9	13565697	rs60613246	C	0.3731	0.8707	0.642	1.181	2.87E-13	0.81	0.76	0.86	2.75E-13	0.812	2.75E-13	0.812	0.6478	0
9	13565966	rs34416482	T	0.364	1.151	0.8492	1.561	1.46E-13	1.24	1.17	1.31	7.25E-14	1.2369	7.25E-14	1.2369	0.6375	0
9	13566457	rs34074476	A	0.3471	1.157	0.8533	1.57	1.33E-12	1.23	1.16	1.3	4.54E-13	1.2275	4.54E-13	1.2275	0.699	0
9	13566853	rs61183216	T	0.3494	1.157	0.8526	1.57	3.31E-12	1.22	1.15	1.29	1.43E-12	1.2179	1.43E-12	1.2179	0.7376	0
9	13566988	rs12684945	A	0.3494	1.157	0.8526	1.57	1.83E-11	1.21	1.14	1.28	1.28E-11	1.2083	1.28E-11	1.2083	0.7772	0
9	13567070	rs12683079	A	0.3494	0.8644	0.6371	1.173	1.87E-10	0.83	0.79	0.88	3.03E-11	0.8311	3.03E-11	0.8311	0.7975	0
9	13567094	rs12685155	A	0.3232	1.167	0.8593	1.584	3.24E-13	1.23	1.17	1.31	4.22E-13	1.2279	4.22E-13	1.2279	0.7402	0
9	13567441	rs12685187	A	0.3526	1.156	0.8518	1.568	1.76E-11	1.21	1.14	1.28	1.29E-11	1.2082	1.29E-11	1.2082	0.773	0
9	13567823	rs34813744	T	0.26	1.193	0.8778	1.62	3.86E-13	1.23	1.17	1.31	4.20E-13	1.2288	4.20E-13	1.2288	0.8477	0
9	13567946	rs35638627	A	0.2662	0.8404	0.6185	1.142	3.65E-13	0.81	0.77	0.86	1.68E-13	0.811	1.68E-13	0.811	0.8168	0
9	13567985	rs35641278	A	0.2797	1.185	0.8713	1.61	2.23E-13	1.24	1.17	1.31	5.63E-14	1.2382	5.63E-14	1.2382	0.7759	0
9	13568185	rs35846464	A	0.26	0.8385	0.6172	1.139	4.19E-13	0.81	0.77	0.86	1.65E-13	0.8109	1.65E-13	0.8109	0.8279	0
9	13568682	rs2027072	C	0.06834	1.304	0.9802	1.736	6.86E-10	1.18	1.12	1.24	9.75E-11	1.1838	9.75E-11	1.1838	0.5001	0
9	13570463	rs34759288	C	0.3202	1.168	0.8596	1.588	9.26E-15	1.25	1.18	1.32	6.21E-15	1.2472	6.21E-15	1.2472	0.67	0
9	13570729	rs1556576	T	0.1084	1.263	0.9498	1.679	2.81E-10	1.18	1.12	1.25	1.46E-10	1.1826	1.46E-10	1.1826	0.6456	0
9	13592419	rs1029140	T	0.1463	1.271	0.9196	1.757	7.27E-08	1.17	1.11	1.24	4.77E-08	1.173	4.77E-08	1.173	0.6218	0
9	13592767	rs10123222	A	0.06216	1.356	0.9847	1.867	3.16E-08	1.18	1.11	1.25	3.87E-09	1.1851	3.87E-09	1.1851	0.4018	0
9	13592775	rs10123223	A	0.06216	1.356	0.9847	1.867	2.72E-08	1.18	1.11	1.25	3.87E-09	1.1851	3.87E-09	1.1851	0.4018	0
9	13595903	rs1931231	T	0.01093	0.7068	0.5411	0.9233	1.87E-09	0.86	0.82	0.91	5.48E-11	0.8548	0.0192	0.8162	0.1565	50.2
9	13596117	rs72710071	A	0.3613	0.8105	0.5163	1.272	9.57E-12	1.27	1.18	1.36	2.84E-11	1.2573	0.7399	1.0747	0.0536	73.15
9	13596317	rs10809994	A	0.02779	0.74	0.5659	0.9677	8.92E-09	0.87	0.83	0.91	2.50E-09	0.8656	0.004968	0.8485	0.2443	26.24
9	13596333	rs10809995	T	0.01606	0.7208	0.5522	0.941	1.32E-09	0.86	0.82	0.9	1.04E-10	0.8552	0.009151	0.8282	0.2014	38.73
9	13596675	rs34804190	A	0.006663	1.445	1.108	1.886	5.73E-09	1.15	1.1	1.21	1.09E-09	1.1583	0.04519	1.2397	0.098	63.48
9	100077417	rs7036518	T	0.03285	0.7507	0.5768	0.9769	1.63E-07	0.88	0.84	0.92	1.93E-08	0.8757	0.007902	0.8588	0.2444	26.19
9	100080659	rs7018981	T	0.03285	0.7507	0.5768	0.9769	1.95E-07	0.88	0.84	0.92	1.93E-08	0.8757	0.007902	0.8588	0.2444	26.19
9	100090162	rs10988969	A	0.02522	0.7404	0.5691	0.9633	2.22E-07	0.88	0.84	0.93	1.73E-08	0.8753	0.01945	0.849	0.2055	37.62
9	100119495	rs846755	C	0.03154	0.7492	0.5759	0.9748	1.64E-07	0.88	0.84	0.92	2.14E-08	0.8756	0.009535	0.8572	0.2383	28.1
9	100126039	rs846762	T	0.03062	0.7478	0.5747	0.9732	2.82E-07	0.88	0.84	0.93	1.87E-08	0.8756	0.01099	0.856	0.2331	29.66
9	100252736	rs1338121	T	0.02588	0.7421	0.5709	0.9648	2.32E-07	0.88	0.84	0.93	1.74E-08	0.8753	0.01767	0.8503	0.2102	36.3
9	100259252	rs2806689	T	0.02467	0.7401	0.5692	0.9623	2.69E-07	0.88	0.84	0.93	1.71E-08	0.8753	0.0199	0.8486	0.2034	38.18

9	100283799	rs2787379	T	0.02586	0.7417	0.5703	0.9647	3.02E-07	0.88	0.84	0.93	1.74E-08	0.8753	0.01804	0.8501	0.2095	36.51
9	108610312	rs7038482	T	0.4078	0.8853	0.6635	1.181	1.95E-08	0.86	0.81	0.9	4.14E-08	0.8609	4.14E-08	0.8609	0.8464	0
9	108627272	rs10816690	C	0.4465	1.123	0.8331	1.514	4.96E-09	1.18	1.12	1.25	4.39E-09	1.178	4.39E-09	1.178	0.7494	0
9	108662874	rs1994807	T	0.7074	0.9504	0.7287	1.24	2.08E-09	1.15	1.1	1.21	1.24E-08	1.1434	0.273	1.0954	0.1659	47.91
9	108662941	rs7044044	T	0.6956	1.054	0.8087	1.375	2.16E-09	0.87	0.83	0.91	1.42E-08	0.8751	0.2861	0.9143	0.1629	48.64
9	108665474	rs2417919	T	0.6956	0.9484	0.7274	1.237	2.94E-09	1.15	1.1	1.21	1.27E-08	1.1433	0.2905	1.0936	0.161	49.11
9	108665697	rs1319974	A	0.7467	1.044	0.802	1.36	3.56E-09	0.87	0.83	0.91	1.33E-08	0.8748	0.2127	0.9081	0.1829	43.62
9	108669195	rs1319971	T	0.6862	0.9467	0.726	1.235	2.89E-09	1.15	1.1	1.21	1.28E-08	1.1433	0.3042	1.0922	0.1574	49.97
9	108670660	rs2417922	T	0.6756	1.058	0.8118	1.38	2.68E-09	0.87	0.83	0.91	1.47E-08	0.8752	0.3169	0.9169	0.1545	50.68
9	108677675	rs7852537	A	0.6862	1.056	0.81	1.377	9.72E-10	0.86	0.82	0.91	8.01E-10	0.8653	0.3256	0.9126	0.1357	55.09
9	108679274	rs4978724	C	0.9877	1.002	0.771	1.302	9.94E-11	1.17	1.11	1.22	6.77E-11	1.1645	0.01158	1.145	0.254	23.15
9	108679642	rs6477666	T	0.947	0.9912	0.7629	1.288	5.66E-10	0.86	0.82	0.9	4.09E-10	0.8638	0.0001184	0.8688	0.2954	8.65
9	108680767	rs7851111	T	0.9583	1.007	0.775	1.308	5.13E-10	1.16	1.11	1.21	7.82E-10	1.155	9.79E-05	1.1489	0.2973	7.95
9	108681548	rs1319972	T	0.983	1.003	0.7722	1.302	5.43E-10	1.16	1.11	1.21	8.10E-10	1.1548	0.001431	1.1444	0.2828	13.3
9	108681684	rs937158	T	0.9637	0.9939	0.7647	1.292	2.01E-10	1.16	1.11	1.22	8.69E-10	1.1545	0.01666	1.1357	0.2555	22.67
9	108681851	rs937159	A	0.9637	0.9939	0.7647	1.292	1.98E-10	1.16	1.11	1.22	8.69E-10	1.1545	0.01666	1.1357	0.2555	22.67
9	108682289	rs2417925	T	0.9637	0.9939	0.7647	1.292	1.74E-10	1.16	1.11	1.22	8.69E-10	1.1545	0.01666	1.1357	0.2555	22.67
9	108684737	rs7859737	A	0.9344	1.011	0.7777	1.315	3.59E-11	0.85	0.82	0.9	1.60E-11	0.8545	0.07557	0.8817	0.2021	38.54
9	108686328	rs1873759	T	0.9637	0.9939	0.7647	1.292	5.37E-10	1.16	1.11	1.21	8.69E-10	1.1545	0.01666	1.1357	0.2555	22.67
9	108690139	rs10125599	A	0.9375	0.9896	0.761	1.287	3.01E-10	1.16	1.11	1.22	8.99E-10	1.1544	0.03167	1.1317	0.2431	26.62
9	108690671	rs1339878	C	0.9502	0.9917	0.7628	1.289	3.05E-10	1.16	1.11	1.22	8.84E-10	1.1544	0.02373	1.1336	0.2491	24.73
9	108691586	rs7024563	T	0.9502	0.9917	0.7628	1.289	9.90E-10	1.16	1.1	1.21	8.84E-10	1.1544	0.02373	1.1336	0.2491	24.73
9	108692624	rs1339879	T	0.8703	1.022	0.7857	1.33	1.88E-10	0.86	0.82	0.9	4.41E-10	0.8645	0.09987	0.8913	0.2054	37.65
9	108693021	rs12683387	A	0.8703	0.9783	0.752	1.273	1.08E-10	1.17	1.11	1.22	8.29E-11	1.1637	0.1211	1.1233	0.1892	42
9	108693228	rs12685634	T	0.8703	1.022	0.7857	1.33	1.07E-10	0.86	0.82	0.9	4.41E-10	0.8645	0.09987	0.8913	0.2054	37.65
9	108694631	rs10759297	T	0.8703	0.9783	0.752	1.273	1.17E-10	1.16	1.11	1.22	8.33E-10	1.154	0.09268	1.1213	0.2113	36.01
9	108694816	rs2900470	T	0.8703	0.9783	0.752	1.273	2.28E-10	1.16	1.11	1.22	8.33E-10	1.154	0.09268	1.1213	0.2113	36.01
9	108695754	rs7853769	A	0.8703	0.9783	0.752	1.273	2.35E-10	1.16	1.11	1.22	8.33E-10	1.154	0.09268	1.1213	0.2113	36.01
9	108702336	rs7860325	A	0.5026	1.094	0.8406	1.425	1.80E-09	1.15	1.1	1.21	3.15E-09	1.1483	3.15E-09	1.1483	0.7151	0
9	108710722	rs6477672	T	0.9966	0.9994	0.7659	1.304	1.04E-10	0.86	0.82	0.9	8.44E-10	0.864	0.003257	0.8736	0.2761	15.68
9	108711242	rs9886871	A	0.8696	1.023	0.7819	1.338	9.61E-10	1.16	1.11	1.22	2.07E-09	1.1555	2.07E-09	1.1555	0.3665	0
9	108713256	rs1934464	T	0.9966	0.9994	0.7659	1.304	8.81E-11	0.85	0.81	0.9	3.79E-11	0.8542	0.02111	0.8722	0.2405	27.43
9	108713344	rs7021659	T	0.9667	1.006	0.7681	1.317	1.63E-09	1.16	1.11	1.22	2.35E-09	1.1549	4.09E-06	1.152	0.3078	3.85
9	108713949	rs10979448	A	0.9969	0.9995	0.7643	1.307	8.58E-11	1.17	1.12	1.23	2.01E-10	1.1644	0.01137	1.1455	0.2573	22.07
9	108717309	rs7039135	T	0.9621	0.9935	0.7599	1.299	4.08E-11	1.17	1.12	1.23	2.11E-10	1.1642	0.03007	1.1395	0.2389	27.91
9	108718387	rs2417929	A	0.9689	0.9947	0.7625	1.298	1.31E-10	1.17	1.11	1.23	1.77E-10	1.1642	0.02895	1.1396	0.2386	27.99

9	108718728	rs7871006	T	0.9621	0.9935	0.7599	1.299	5.90E-11	1.17	1.12	1.23	2.11E-10	1.1642	0.03007	1.1395	0.2389	27.91
9	108718822	rs7855570	A	0.9621	0.9935	0.7599	1.299	4.93E-11	1.17	1.12	1.23	1.77E-10	1.1642	0.03001	1.1395	0.2388	27.93
9	108719036	rs7871396	T	0.9621	0.9935	0.7599	1.299	9.65E-11	1.17	1.12	1.23	2.11E-10	1.1642	0.03007	1.1395	0.2389	27.91
9	108719526	rs10979450	A	0.9621	0.9935	0.7599	1.299	5.25E-11	1.17	1.12	1.23	2.11E-10	1.1642	0.03007	1.1395	0.2389	27.91
9	108719576	rs10979452	A	0.9621	1.007	0.7699	1.316	4.90E-11	0.85	0.81	0.89	4.84E-11	0.8544	0.0463	0.8771	0.2222	32.9
9	108719620	rs10979453	A	0.9621	0.9935	0.7599	1.299	5.44E-11	1.17	1.12	1.23	1.77E-10	1.1642	0.03001	1.1395	0.2388	27.93
9	108719672	rs10979454	A	0.9621	0.9935	0.7599	1.299	4.25E-11	1.17	1.12	1.23	1.77E-10	1.1642	0.03001	1.1395	0.2388	27.93
9	108719882	rs1538655	T	0.9621	0.9935	0.7599	1.299	3.90E-11	1.17	1.12	1.23	1.77E-10	1.1642	0.03001	1.1395	0.2388	27.93
9	108719891	rs10816702	C	0.9689	1.005	0.7706	1.311	1.29E-10	0.86	0.82	0.9	8.85E-10	0.8641	0.01409	0.8781	0.258	21.85
9	108720106	rs1538656	A	0.9621	0.9935	0.7599	1.299	4.13E-11	1.17	1.12	1.23	1.77E-10	1.1642	0.03001	1.1395	0.2388	27.93
9	108720220	rs1538657	A	0.9621	1.007	0.7699	1.316	3.96E-11	0.85	0.81	0.89	4.00E-11	0.8544	0.04624	0.8771	0.2221	32.91
9	108720394	rs2417930	T	0.9621	0.9935	0.7599	1.299	2.62E-11	1.18	1.12	1.23	2.12E-11	1.1738	0.0523	1.1412	0.2153	34.87
9	108720443	rs2417932	A	0.9621	0.9935	0.7599	1.299	1.02E-10	1.17	1.12	1.23	1.77E-10	1.1642	0.03001	1.1395	0.2388	27.93
9	108720504	rs2417933	T	0.9621	1.007	0.7699	1.316	4.80E-11	0.85	0.81	0.89	4.00E-11	0.8544	0.04624	0.8771	0.2221	32.91
9	108720572	rs2417934	T	0.9621	0.9935	0.7599	1.299	4.72E-11	1.17	1.12	1.23	1.77E-10	1.1642	0.03001	1.1395	0.2388	27.93
9	108720581	rs2900472	T	0.9621	1.007	0.7699	1.316	4.62E-11	0.85	0.81	0.89	4.00E-11	0.8544	0.04624	0.8771	0.2221	32.91
9	108721949	rs10816703	T	0.9306	0.9882	0.7558	1.292	7.16E-11	1.17	1.12	1.23	2.21E-10	1.164	0.05284	1.1345	0.2242	32.31
9	108722106	rs2417935	C	0.9306	1.012	0.774	1.323	8.19E-11	0.85	0.81	0.9	4.17E-11	0.8545	0.06931	0.8805	0.2092	36.58
9	108722676	rs10739266	T	0.9376	0.9894	0.7584	1.291	2.97E-10	1.16	1.11	1.22	1.70E-09	1.1543	0.02689	1.1331	0.2485	24.92
9	134523448	rs3118579	T	0.05094	0.7597	0.5764	1.001	5.96E-11	0.84	0.8	0.89	5.22E-12	0.8372	5.22E-12	0.8372	0.4829	0
9	134527056	rs9409901	A	0.06337	1.299	0.9855	1.713	6.59E-12	1.2	1.14	1.26	5.74E-13	1.2032	5.74E-13	1.2032	0.5804	0
9	134527565	rs3118584	T	0.04561	1.326	1.006	1.75	2.40E-11	1.19	1.13	1.25	5.49E-12	1.1943	5.49E-12	1.1943	0.4514	0
9	134527972	rs2182640	T	0.06577	1.296	0.9833	1.707	3.99E-12	1.2	1.14	1.26	5.83E-13	1.2031	5.83E-13	1.2031	0.5907	0
9	134528616	rs3118585	T	0.04561	1.326	1.006	1.75	9.03E-12	1.2	1.14	1.26	5.75E-13	1.204	5.75E-13	1.204	0.4872	0
9	134529487	rs3118587	T	0.05506	0.7628	0.5785	1.006	3.63E-12	0.83	0.79	0.88	1.72E-13	0.8277	1.72E-13	0.8277	0.5563	0
9	134529820	rs3118588	A	0.05011	0.7588	0.5757	1	4.17E-12	0.83	0.79	0.88	1.63E-13	0.8275	1.63E-13	0.8275	0.5314	0
9	134530473	rs2148702	A	0.05011	1.318	0.9999	1.737	2.76E-12	1.2	1.14	1.26	4.99E-13	1.2037	4.99E-13	1.2037	0.5128	0
9	134531605	rs1555951	A	0.04561	1.326	1.006	1.75	4.30E-12	1.2	1.14	1.26	5.75E-13	1.204	5.75E-13	1.204	0.4872	0
9	134532196	rs4841895	A	0.3861	0.8877	0.678	1.162	2.55E-08	0.86	0.82	0.91	5.26E-09	0.8609	5.26E-09	0.8609	0.8208	0
9	134532208	rs3132322	T	0.2209	0.8446	0.6446	1.107	1.88E-10	0.85	0.81	0.89	8.60E-11	0.8498	8.60E-11	0.8498	0.9638	0
9	134532256	rs4841896	A	0.3861	0.8877	0.678	1.162	2.41E-08	0.86	0.82	0.91	5.26E-09	0.8609	5.26E-09	0.8609	0.8208	0
9	134532269	rs4842036	T	0.3283	0.8731	0.6651	1.146	9.81E-08	0.86	0.82	0.91	2.28E-08	0.8605	2.28E-08	0.8605	0.9149	0
9	134532319	rs4841897	A	0.3283	0.8731	0.6651	1.146	5.66E-08	0.86	0.82	0.91	2.85E-08	0.8605	2.85E-08	0.8605	0.9149	0
9	134532387	rs4841898	T	0.3861	0.8877	0.678	1.162	2.18E-08	0.86	0.82	0.91	5.26E-09	0.8609	5.26E-09	0.8609	0.8208	0
9	134532566	rs4841899	T	0.3861	1.127	0.8605	1.475	1.71E-08	1.16	1.1	1.22	8.98E-09	1.1588	8.98E-09	1.1588	0.8366	0
9	134532598	rs9409902	A	0.2209	1.184	0.9035	1.551	4.64E-11	1.18	1.12	1.23	6.61E-12	1.1801	6.61E-12	1.1801	0.9807	0

9	134532822	rs10745372	T	0.3861	0.8877	0.678	1.162	1.66E-08	0.86	0.82	0.91	5.26E-09	0.8609	5.26E-09	0.8609	0.8208	0
9	134532938	rs10745373	T	0.3861	1.127	0.8605	1.475	1.84E-08	1.16	1.1	1.22	8.98E-09	1.1588	8.98E-09	1.1588	0.8366	0
9	134533193	rs9409903	T	0.2921	1.158	0.8815	1.521	8.30E-11	1.18	1.12	1.24	5.78E-11	1.1793	5.78E-11	1.1793	0.8942	0
9	134533595	rs3132321	T	0.3936	1.127	0.8563	1.483	2.87E-11	1.19	1.13	1.25	8.04E-12	1.1879	8.04E-12	1.1879	0.7025	0
9	134533837	rs3132320	T	0.4065	1.122	0.8548	1.473	6.09E-11	1.18	1.12	1.24	7.59E-11	1.178	7.59E-11	1.178	0.7212	0
9	134534013	rs3132319	A	0.07539	0.7767	0.5878	1.026	1.13E-12	0.83	0.79	0.87	2.55E-13	0.8282	2.55E-13	0.8282	0.646	0
9	134534081	rs3132318	T	0.2831	1.161	0.884	1.525	7.01E-11	1.18	1.12	1.24	6.66E-11	1.1794	6.66E-11	1.1794	0.9086	0
9	134534307	rs3118592	A	0.2775	0.86	0.655	1.129	6.05E-10	0.85	0.81	0.9	1.40E-10	0.8503	1.40E-10	0.8503	0.934	0
9	134534488	rs3118593	A	0.2811	1.162	0.8845	1.526	5.41E-10	1.17	1.12	1.23	5.52E-10	1.1697	5.52E-10	1.1697	0.9613	0
9	134536579	rs1409832	T	0.08438	0.7769	0.5832	1.035	2.36E-14	0.82	0.77	0.86	2.03E-14	0.8186	2.03E-14	0.8186	0.7165	0
9	134536760	rs9409905	C	0.1714	1.206	0.9222	1.576	6.09E-12	1.19	1.13	1.26	5.01E-12	1.1905	5.01E-12	1.1905	0.9235	0
9	134537254	rs9409977	A	0.1994	0.8388	0.6413	1.097	1.36E-11	0.84	0.8	0.88	6.04E-12	0.84	6.04E-12	0.84	0.9918	0
9	134537261	rs9409978	T	0.207	0.8414	0.6434	1.1	1.34E-11	0.84	0.8	0.88	6.22E-12	0.84	6.22E-12	0.84	0.9905	0
9	134537281	rs9409979	A	0.1994	1.192	0.9115	1.559	1.19E-11	1.19	1.13	1.25	5.62E-12	1.1901	5.62E-12	1.1901	0.9904	0
9	134537343	rs10858174	T	0.1559	0.8234	0.6296	1.077	1.02E-10	0.84	0.8	0.89	2.37E-11	0.8394	2.37E-11	0.8394	0.8862	0
9	134537353	rs9409906	T	0.1994	0.8388	0.6413	1.097	1.65E-11	0.84	0.8	0.88	6.04E-12	0.84	6.04E-12	0.84	0.9918	0
9	134537506	rs10858176	A	0.1994	1.192	0.9115	1.559	8.63E-12	1.19	1.13	1.25	5.62E-12	1.1901	5.62E-12	1.1901	0.9904	0
9	134537550	rs4370607	A	0.1994	1.192	0.9115	1.559	1.23E-11	1.19	1.13	1.25	5.62E-12	1.1901	5.62E-12	1.1901	0.9904	0
9	134537561	rs4313224	A	0.1994	0.8388	0.6413	1.097	1.22E-11	0.84	0.8	0.88	5.04E-12	0.84	5.04E-12	0.84	0.9918	0
9	134537744	rs4391503	A	0.05049	1.305	0.9994	1.704	5.30E-13	1.2	1.14	1.26	4.20E-14	1.2033	4.20E-14	1.2033	0.5443	0
9	134537873	rs4240698	T	0.1994	1.192	0.9115	1.559	1.26E-11	1.19	1.13	1.25	5.62E-12	1.1901	5.62E-12	1.1901	0.9904	0
9	134538030	rs4240699	A	0.1994	0.8388	0.6413	1.097	1.21E-11	0.84	0.8	0.88	5.04E-12	0.84	5.04E-12	0.84	0.9918	0
9	134538137	rs4842039	A	0.2072	0.8415	0.6435	1.1	8.46E-12	0.84	0.8	0.88	5.18E-12	0.8401	5.18E-12	0.8401	0.9898	0
9	134538164	rs4842040	A	0.2072	0.8415	0.6435	1.1	8.37E-12	0.84	0.8	0.88	5.18E-12	0.8401	5.18E-12	0.8401	0.9898	0
9	134538435	rs4074178	A	0.05035	1.305	0.9996	1.705	6.31E-13	1.2	1.14	1.26	4.21E-14	1.2033	4.21E-14	1.2033	0.5446	0
9	134538828	rs9409980	A	0.05035	1.305	0.9996	1.705	2.28E-12	1.19	1.13	1.25	5.06E-13	1.1936	5.06E-13	1.1936	0.5052	0
9	134538872	rs9409907	A	0.05101	0.7666	0.5869	1.001	1.28E-12	0.84	0.8	0.88	5.52E-13	0.8375	5.52E-13	0.8375	0.5091	0
9	134538880	rs9409981	T	0.05101	1.305	0.9988	1.704	1.24E-12	1.19	1.14	1.25	6.17E-13	1.1936	6.17E-13	1.1936	0.5053	0
9	134539052	rs7038424	T	0.1559	0.8216	0.6262	1.078	6.79E-11	0.84	0.79	0.88	5.26E-11	0.8393	5.26E-11	0.8393	0.8753	0
9	134539093	rs7048416	T	0.05117	1.304	0.9987	1.704	2.72E-12	1.19	1.13	1.25	5.10E-13	1.1935	5.10E-13	1.1935	0.5088	0
9	134539578	rs1316404	A	0.03774	1.327	1.016	1.733	5.87E-13	1.2	1.14	1.26	4.41E-14	1.2039	4.41E-14	1.2039	0.4672	0
9	134539616	rs943425	A	0.03774	1.327	1.016	1.733	9.12E-13	1.2	1.14	1.26	4.41E-14	1.2039	4.41E-14	1.2039	0.4672	0
9	134539911	rs7858156	C	0.1187	0.8054	0.6137	1.057	7.44E-11	0.84	0.79	0.88	4.38E-11	0.8387	4.38E-11	0.8387	0.766	0
9	134540058	rs4842044	T	0.153	0.8218	0.6279	1.076	8.57E-12	0.84	0.8	0.88	5.00E-12	0.8394	5.00E-12	0.8394	0.8754	0
9	134540402	rs1536478	A	0.153	0.8218	0.6279	1.076	4.62E-12	0.84	0.8	0.88	5.00E-12	0.8394	5.00E-12	0.8394	0.8754	0
9	134540491	rs1536477	T	0.03774	1.327	1.016	1.733	9.42E-13	1.2	1.14	1.26	1.03E-13	1.2041	1.03E-13	1.2041	0.4675	0

9	134540802	rs9409908	T	0.153	0.8218	0.6279	1.076	3.76E-12	0.84	0.79	0.88	5.00E-12	0.8394	5.00E-12	0.8394	0.8754	0
9	134542491	rs9409982	A	0.1435	0.8178	0.6247	1.071	1.40E-11	0.84	0.8	0.88	4.79E-12	0.8392	4.79E-12	0.8392	0.8482	0
9	134542600	rs9409911	A	0.1152	1.244	0.948	1.634	8.08E-11	1.19	1.13	1.26	5.54E-11	1.192	5.54E-11	1.192	0.7537	0
9	134542701	rs9409912	T	0.03468	1.334	1.021	1.742	2.82E-12	1.19	1.13	1.25	5.00E-13	1.1944	5.00E-13	1.1944	0.4098	0
9	134544144	rs4842057	A	0.1417	0.8158	0.6218	1.07	2.76E-10	0.84	0.8	0.89	4.22E-11	0.8391	4.22E-11	0.8391	0.836	0
9	134544300	rs4842058	C	0.04592	0.7622	0.5838	0.9951	8.65E-12	0.84	0.8	0.89	4.25E-13	0.8374	4.25E-13	0.8374	0.4821	0
9	134544368	rs3132307	C	0.01068	1.43	1.087	1.882	9.34E-21	1.27	1.21	1.34	1.04E-21	1.275	1.04E-21	1.275	0.4049	0
9	134544468	rs3118515	A	0.009644	1.436	1.092	1.89	1.97E-20	1.27	1.21	1.33	6.94E-22	1.2751	6.94E-22	1.2751	0.3878	0
9	134547946	rs3118516	A	0.01852	1.375	1.055	1.793	1.08E-27	1.31	1.24	1.37	1.96E-29	1.312	1.96E-29	1.312	0.7247	0
9	134548059	rs3118517	T	0.008787	1.423	1.093	1.853	4.92E-26	1.29	1.23	1.35	1.75E-27	1.2939	1.75E-27	1.2939	0.4733	0
9	134548237	rs3118518	A	0.01614	1.384	1.062	1.802	1.83E-28	1.31	1.25	1.37	1.06E-29	1.3123	1.06E-29	1.3123	0.6885	0
9	134548366	rs3132306	T	0.01556	0.7223	0.555	0.9401	5.33E-28	0.77	0.73	0.8	3.26E-28	0.7684	3.26E-28	0.7684	0.6399	0
9	134548682	rs1536482	A	0.008698	1.421	1.093	1.847	1.64E-24	1.28	1.22	1.34	5.30E-26	1.2842	5.30E-26	1.2842	0.4424	0
9	134548838	rs1536483	T	0.004801	1.455	1.121	1.889	1.64E-23	1.27	1.21	1.33	1.06E-24	1.2755	1.42E-21	1.2764	0.3147	1.06
9	134548862	rs1536484	C	0.02593	1.346	1.036	1.748	1.62E-22	1.28	1.22	1.35	4.59E-23	1.2823	4.59E-23	1.2823	0.7111	0
9	134549319	rs3118519	A	0.04868	0.769	0.5922	0.9985	1.02E-23	0.78	0.75	0.82	3.35E-25	0.7796	3.35E-25	0.7796	0.9165	0
9	134549749	rs3118520	A	0.04868	0.769	0.5922	0.9985	1.02E-23	0.77	0.74	0.81	1.68E-25	0.77	1.68E-25	0.77	0.9924	0
9	134552050	rs3132304	A	0.06202	1.339	0.9854	1.821	7.22E-09	1.19	1.12	1.26	8.26E-10	1.1948	8.26E-10	1.1948	0.4591	0
9	134626453	rs10858263	T	0.0457	0.7582	0.5779	0.9947	7.28E-08	0.86	0.82	0.91	6.37E-09	0.856	6.37E-09	0.856	0.3721	0
9	134646897	rs7863874	A	0.1959	1.265	0.8859	1.806	2.11E-08	1.19	1.12	1.27	1.65E-08	1.1921	1.65E-08	1.1921	0.7403	0
9	134648061	rs12554217	A	0.1648	0.7806	0.5504	1.107	8.02E-09	0.83	0.78	0.89	1.31E-09	0.8285	1.31E-09	0.8285	0.7347	0
9	134648527	rs59699848	T	0.03226	0.6967	0.5005	0.9699	4.17E-09	0.84	0.79	0.89	1.52E-09	0.8351	0.0007428	0.8235	0.2754	15.93
9	134648821	rs61045038	A	0.03282	1.441	1.03	2.017	2.21E-08	1.2	1.12	1.27	1.86E-09	1.2074	5.76E-05	1.2171	0.2936	9.36
9	134650018	rs12555243	T	0.02418	0.6855	0.4937	0.9519	8.18E-11	0.83	0.78	0.88	3.31E-11	0.8253	0.0009815	0.8099	0.2607	20.95
9	134650284	rs56361301	A	0.03008	0.696	0.5016	0.9657	3.97E-11	0.82	0.78	0.87	1.84E-12	0.816	1.84E-12	0.816	0.3338	0
9	134650292	rs60063652	T	0.02418	1.459	1.051	2.026	1.19E-10	1.21	1.14	1.28	1.24E-11	1.2168	0.0003333	1.2356	0.2712	17.41
9	134650617	rs12005599	T	0.02334	0.684	0.4926	0.9497	5.41E-11	0.82	0.78	0.87	1.92E-12	0.8156	2.68E-05	0.8072	0.2863	12.06
9	134657730	rs75675845	A	0.1479	1.297	0.9119	1.845	1.15E-07	1.21	1.13	1.3	3.70E-08	1.2132	3.70E-08	1.2132	0.7049	0
9	134665688	rs7854299	A	0.01661	0.6856	0.5035	0.9337	1.72E-08	0.85	0.8	0.9	4.27E-09	0.8439	0.01971	0.8072	0.18	44.37
9	134666044	rs58681734	A	0.01661	1.458	1.071	1.986	8.23E-08	1.17	1.11	1.24	1.42E-08	1.1787	0.02619	1.2369	0.1699	46.91
9	134666334	rs58811417	T	0.01661	1.458	1.071	1.986	4.58E-08	1.17	1.11	1.24	1.42E-08	1.1787	0.02619	1.2369	0.1699	46.91
9	134667465	rs6537937	A	0.02055	0.6939	0.5093	0.9454	5.75E-09	0.84	0.79	0.89	6.46E-10	0.8345	0.004013	0.813	0.2341	29.36
9	134667477	rs6537938	A	0.01661	1.458	1.071	1.986	3.69E-08	1.18	1.11	1.25	2.60E-09	1.1885	0.01627	1.2394	0.187	42.56
9	134667520	rs6537939	T	0.01661	1.458	1.071	1.986	2.96E-08	1.18	1.11	1.25	2.60E-09	1.1885	0.01627	1.2394	0.187	42.56
9	134667539	rs6537940	T	0.01661	1.458	1.071	1.986	2.94E-08	1.18	1.11	1.25	2.60E-09	1.1885	0.01627	1.2394	0.187	42.56
9	134667571	rs6537941	A	0.01661	1.458	1.071	1.986	3.26E-08	1.18	1.11	1.25	2.60E-09	1.1885	0.01627	1.2394	0.187	42.56

9	134667710	rs6537942	A	0.01751	0.6876	0.5048	0.9366	4.40E-08	0.85	0.8	0.9	4.86E-09	0.8439	0.01807	0.809	0.1863	42.75
9	134667927	rs7032486	T	0.01661	1.458	1.071	1.986	3.13E-08	1.18	1.11	1.25	2.60E-09	1.1885	0.01627	1.2394	0.187	42.56
9	134667929	rs7032489	C	0.01661	0.6856	0.5035	0.9337	3.35E-08	0.85	0.8	0.9	4.75E-09	0.8438	0.01971	0.8072	0.1801	44.36
9	134667941	rs7042369	T	0.01661	0.6856	0.5035	0.9337	3.10E-08	0.85	0.8	0.9	4.75E-09	0.8438	0.01971	0.8072	0.1801	44.36
9	134668498	rs12000599	A	0.01661	1.458	1.071	1.986	1.67E-08	1.18	1.11	1.25	2.60E-09	1.1885	0.01627	1.2394	0.187	42.56
9	134668853	rs78786052	A	0.01661	1.458	1.071	1.986	1.93E-08	1.18	1.11	1.25	2.60E-09	1.1885	0.01627	1.2394	0.187	42.56
9	134672231	rs73665731	A	0.01584	0.6838	0.5022	0.9312	3.62E-08	0.85	0.8	0.9	5.17E-09	0.8437	0.02119	0.8055	0.1746	45.75
9	134672573	rs12003552	A	0.01584	0.6838	0.5022	0.9312	3.12E-08	0.85	0.8	0.9	5.17E-09	0.8437	0.02119	0.8055	0.1746	45.75
9	134673486	rs6537943	A	0.01584	0.6838	0.5022	0.9312	3.54E-08	0.85	0.8	0.9	5.17E-09	0.8437	0.02119	0.8055	0.1746	45.75
9	134675045	rs7855673	A	0.0222	1.438	1.053	1.963	2.43E-08	1.18	1.11	1.25	2.96E-09	1.1878	0.00875	1.2249	0.2209	33.28
9	134676205	rs7044529	T	0.03034	1.416	1.034	1.939	3.03E-07	1.17	1.1	1.24	2.71E-08	1.1775	0.007223	1.206	0.2425	26.81
9	136936281	rs7045803	C	0.857	0.9756	0.746	1.276	2.96E-09	0.86	0.82	0.91	2.08E-09	0.8635	2.08E-09	0.8635	0.3647	0
9	136936488	rs6560652	T	0.8338	1.029	0.787	1.346	3.78E-09	1.16	1.1	1.22	3.60E-09	1.1556	3.60E-09	1.1556	0.3891	0
9	136937244	rs10116414	T	0.857	0.9756	0.746	1.276	3.29E-09	0.86	0.82	0.91	2.08E-09	0.8635	2.08E-09	0.8635	0.3647	0
9	136937553	rs10118333	A	0.08192	1.295	0.9678	1.733	1.75E-08	1.17	1.11	1.24	6.13E-09	1.1741	6.13E-09	1.1741	0.5021	0
9	136938989	rs2090627	T	0.104	1.28	0.9505	1.724	4.86E-09	1.18	1.12	1.25	1.88E-09	1.1833	1.88E-09	1.1833	0.5987	0
9	136941910	rs7040705	A	0.8031	0.967	0.7426	1.259	5.39E-09	0.87	0.82	0.91	2.26E-08	0.873	2.26E-08	0.873	0.4402	0
9	136943538	rs2271872	C	0.4945	0.9129	0.7029	1.186	3.67E-09	0.86	0.82	0.91	1.40E-09	0.8617	1.40E-09	0.8617	0.6601	0
9	136944238	rs28578007	T	0.7214	1.049	0.8065	1.364	2.19E-09	1.16	1.11	1.22	3.11E-09	1.1561	3.11E-09	1.1561	0.4612	0
9	136945452	rs2071006	T	0.5635	1.081	0.8305	1.406	7.13E-09	1.15	1.1	1.21	1.43E-08	1.1477	1.43E-08	1.1477	0.6505	0
9	136946019	rs7862602	T	0.5671	0.9261	0.712	1.205	9.13E-09	0.87	0.83	0.91	1.85E-08	0.8718	1.85E-08	0.8718	0.6468	0
9	136951220	rs925936	T	0.1734	1.223	0.9152	1.635	2.24E-08	1.17	1.11	1.24	1.04E-08	1.1718	1.04E-08	1.1718	0.7689	0
9	136953821	rs2271869	A	0.7104	1.05	0.8115	1.359	4.69E-08	1.15	1.09	1.2	3.04E-08	1.1463	3.04E-08	1.1463	0.4968	0
9	136953826	rs2271868	T	0.6912	1.054	0.814	1.364	1.19E-08	1.15	1.1	1.21	2.95E-08	1.1465	2.95E-08	1.1465	0.5156	0
9	136955667	rs868102	A	0.6327	0.939	0.7256	1.215	9.74E-08	0.87	0.83	0.92	3.03E-08	0.8723	3.03E-08	0.8723	0.5689	0
9	136956223	rs908833	T	0.7176	0.9535	0.7368	1.234	9.85E-08	0.87	0.83	0.92	3.43E-08	0.8728	3.43E-08	0.8728	0.4939	0
9	136961154	rs11145945	A	0.3897	0.8789	0.6549	1.179	5.56E-09	0.85	0.8	0.9	5.79E-09	0.851	5.79E-09	0.851	0.8267	0
9	136961876	rs908837	A	0.4126	0.8842	0.6586	1.187	4.59E-09	0.85	0.8	0.9	5.36E-09	0.8511	5.36E-09	0.8511	0.7964	0
9	136962187	rs908839	C	0.5074	0.9174	0.7109	1.184	1.28E-11	0.84	0.8	0.89	6.69E-12	0.8427	6.69E-12	0.8427	0.5061	0
9	136962358	rs885070	A	0.796	1.034	0.8005	1.337	3.34E-08	1.15	1.09	1.21	3.46E-08	1.1457	3.46E-08	1.1457	0.4247	0
9	136962688	rs11145946	T	0.6433	1.073	0.7953	1.449	2.45E-08	1.16	1.1	1.23	3.94E-08	1.1573	3.94E-08	1.1573	0.6158	0
9	136963077	rs11145947	T	0.6542	0.9337	0.6918	1.26	1.65E-08	0.85	0.81	0.9	7.95E-09	0.8526	7.95E-09	0.8526	0.546	0
9	136963460	rs11145948	A	0.5158	1.088	0.8434	1.404	9.89E-12	1.19	1.13	1.25	7.60E-12	1.1861	7.60E-12	1.1861	0.4987	0
9	136963506	rs11145949	A	0.5158	0.919	0.7123	1.186	1.48E-11	0.84	0.8	0.89	6.81E-12	0.8428	6.81E-12	0.8428	0.4974	0
9	136964382	rs4542025	A	0.5839	0.9313	0.7218	1.202	9.17E-11	0.85	0.81	0.89	1.72E-10	0.8529	1.72E-10	0.8529	0.4904	0
9	136964414	rs13292961	A	0.6638	0.9451	0.7328	1.219	8.51E-11	0.85	0.81	0.89	1.98E-10	0.8533	1.98E-10	0.8533	0.4226	0

9	136964428	rs13289286	T	0.5839	1.074	0.8322	1.386	1.14E-10	1.18	1.12	1.24	8.00E-11	1.1759	8.00E-11	1.1759	0.4773	0
9	136964561	rs7040970	T	0.5605	0.9271	0.7184	1.196	4.98E-11	0.85	0.81	0.89	1.39E-10	0.8527	1.39E-10	0.8527	0.5124	0
9	136965648	rs7868669	A	0.4716	0.9107	0.706	1.175	9.71E-11	0.85	0.81	0.89	1.17E-10	0.8521	1.17E-10	0.8521	0.6022	0
9	136965812	rs11145951	T	0.4867	0.9136	0.7083	1.178	7.72E-11	0.85	0.81	0.89	1.21E-10	0.8522	1.21E-10	0.8522	0.5856	0
9	136965908	rs72761096	T	0.4937	1.109	0.8249	1.49	2.20E-08	1.17	1.11	1.24	1.93E-08	1.1679	1.93E-08	1.1679	0.7272	0
9	136966038	rs72761097	A	0.4947	1.109	0.8244	1.491	2.34E-08	1.17	1.11	1.24	2.16E-08	1.1679	2.16E-08	1.1679	0.7277	0
9	136966741	rs112406207	A	0.5822	1.087	0.8075	1.463	1.69E-08	1.17	1.11	1.24	2.49E-08	1.1671	2.49E-08	1.1671	0.6334	0
9	136966756	rs111383616	T	0.5822	1.087	0.8075	1.463	1.49E-08	1.17	1.11	1.24	2.49E-08	1.1671	2.49E-08	1.1671	0.6334	0
9	136967018	rs7019538	T	0.4681	0.91	0.7054	1.174	2.69E-11	0.84	0.8	0.89	6.15E-12	0.8425	6.15E-12	0.8425	0.5457	0
9	136967872	rs2386138	T	0.5755	0.9185	0.682	1.237	1.20E-08	0.85	0.81	0.9	9.03E-09	0.8522	9.03E-09	0.8522	0.6157	0
9	136968446	rs11145954	T	0.4541	1.102	0.8543	1.422	3.68E-10	1.17	1.12	1.23	4.54E-10	1.1674	4.54E-10	1.1674	0.6512	0
9	136969280	rs908829	C	0.4541	1.102	0.8543	1.422	1.78E-10	1.18	1.12	1.24	5.23E-11	1.1771	5.23E-11	1.1771	0.6056	0
9	136969581	rs2386137	A	0.5949	1.084	0.805	1.46	2.55E-08	1.17	1.11	1.24	2.84E-08	1.167	2.84E-08	1.167	0.6212	0
9	136969889	rs2386136	A	0.3565	0.8868	0.687	1.145	4.32E-10	0.85	0.81	0.9	9.07E-11	0.8513	9.07E-11	0.8513	0.7495	0
9	136971795	rs11790360	T	0.4056	0.8962	0.6922	1.16	2.51E-10	0.85	0.81	0.9	1.01E-10	0.8516	1.01E-10	0.8516	0.6933	0
10	53436353	rs117905623	T	0.865	0.94	0.4605	1.919	1.89E-08	0.68	0.6	0.78	1.87E-08	0.6874	1.87E-08	0.6874	0.382	0
10	101064592	rs807037	C	0.02538	1.362	1.039	1.787	5.84E-08	1.15	1.1	1.22	1.93E-08	1.1569	0.008636	1.1851	0.2298	30.65
10	119005659	rs11198725	T	0.1331	1.25	0.9341	1.674	4.15E-08	1.16	1.1	1.23	3.32E-08	1.1629	3.32E-08	1.1629	0.6216	0
10	119020249	rs10787897	A	0.2859	1.16	0.8829	1.525	1.58E-08	1.15	1.1	1.21	8.55E-09	1.1503	8.55E-09	1.1503	0.9513	0
10	119020703	rs10886366	A	0.312	0.8657	0.6546	1.145	1.64E-08	0.86	0.81	0.91	1.37E-08	0.8602	1.37E-08	0.8602	0.9637	0
10	119020852	rs10886367	C	0.3144	1.154	0.8727	1.527	1.85E-08	1.16	1.1	1.23	2.05E-08	1.1598	2.05E-08	1.1598	0.9715	0
10	119023094	rs2420492	T	0.3628	1.135	0.8644	1.489	1.73E-08	1.16	1.1	1.22	1.41E-08	1.1591	1.41E-08	1.1591	0.8775	0
10	119024129	rs10886369	A	0.3271	1.15	0.8698	1.519	3.83E-08	1.17	1.1	1.23	1.41E-08	1.1692	1.41E-08	1.1692	0.9054	0
10	119025465	rs494469	A	0.5979	1.075	0.822	1.405	8.99E-08	1.15	1.09	1.2	3.66E-08	1.1474	3.66E-08	1.1474	0.6279	0
10	119025813	rs609163	A	0.3145	1.154	0.873	1.525	6.02E-09	1.18	1.11	1.24	2.32E-09	1.179	2.32E-09	1.179	0.8779	0
10	119025951	rs608660	T	0.3145	0.8666	0.6557	1.145	5.76E-09	0.85	0.8	0.9	4.39E-09	0.8506	4.39E-09	0.8506	0.8939	0
10	119029218	rs11198741	A	0.3382	0.8735	0.6624	1.152	1.04E-08	0.85	0.81	0.9	2.18E-09	0.8508	2.18E-09	0.8508	0.8496	0
10	119039143	rs10787899	A	0.43	1.114	0.8523	1.456	3.32E-09	1.16	1.11	1.22	5.09E-09	1.1584	5.09E-09	1.1584	0.7708	0
10	119043923	rs565922	T	0.3531	0.8772	0.6652	1.157	4.59E-12	0.84	0.8	0.88	3.73E-12	0.8411	3.73E-12	0.8411	0.7624	0
10	119044234	rs3740553	T	0.4249	0.8968	0.6864	1.172	2.08E-10	0.86	0.82	0.9	6.60E-10	0.8611	6.60E-10	0.8611	0.7626	0
10	119044276	rs658352	T	0.3531	1.14	0.8645	1.503	3.71E-12	1.19	1.13	1.25	4.16E-12	1.1884	4.16E-12	1.1884	0.7646	0
10	119044922	rs67487722	A	0.3577	0.8782	0.6658	1.158	3.13E-11	0.84	0.8	0.89	5.50E-12	0.8412	5.50E-12	0.8412	0.7568	0
10	119046651	rs608790	T	0.3531	1.14	0.8645	1.503	4.31E-12	1.19	1.13	1.25	4.16E-12	1.1884	4.16E-12	1.1884	0.7646	0
10	119048962	rs530409	T	0.3577	0.8782	0.6658	1.158	3.74E-12	0.84	0.8	0.88	3.77E-12	0.8412	3.77E-12	0.8412	0.7567	0
10	119051111	rs669964	A	0.3506	1.141	0.865	1.505	1.81E-10	1.18	1.12	1.24	1.75E-10	1.1787	1.75E-10	1.1787	0.8151	0
10	119051561	rs671736	T	0.3506	1.141	0.865	1.505	2.09E-10	1.18	1.12	1.24	1.75E-10	1.1787	1.75E-10	1.1787	0.8151	0

10	119054937	rs10886373	C	0.4378	0.8994	0.688	1.176	3.84E-10	0.86	0.82	0.9	5.78E-10	0.8612	5.78E-10	0.8612	0.747	0
10	119055045	rs10886374	T	0.4378	0.8994	0.688	1.176	3.61E-10	0.86	0.82	0.9	5.78E-10	0.8612	5.78E-10	0.8612	0.747	0
10	119062521	rs2152240	T	0.4335	1.117	0.8471	1.472	5.00E-11	1.19	1.13	1.25	3.57E-11	1.1875	3.57E-11	1.1875	0.659	0
10	119064457	rs10787903	T	0.5338	0.9185	0.7027	1.201	2.54E-09	0.86	0.82	0.9	3.06E-09	0.8619	3.06E-09	0.8619	0.636	0
10	119068783	rs10886377	C	0.3488	0.8762	0.6646	1.155	6.29E-12	0.84	0.8	0.88	3.69E-12	0.8411	3.69E-12	0.8411	0.7684	0
10	119072669	rs11198754	A	0.3488	0.8762	0.6646	1.155	8.87E-12	0.84	0.8	0.88	3.69E-12	0.8411	3.69E-12	0.8411	0.7684	0
10	119073292	rs1397617	T	0.4278	1.114	0.8528	1.456	4.01E-10	1.17	1.11	1.22	1.14E-10	1.1682	1.14E-10	1.1682	0.7236	0
10	119074436	rs10400136	A	0.4278	1.114	0.8528	1.456	2.93E-10	1.17	1.11	1.22	1.14E-10	1.1682	1.14E-10	1.1682	0.7236	0
10	119076264	rs12264824	T	0.3488	0.8762	0.6646	1.155	1.19E-11	0.84	0.8	0.88	4.46E-12	0.8411	4.46E-12	0.8411	0.7684	0
10	119081149	rs3824830	A	0.3488	1.141	0.8656	1.505	1.39E-11	1.19	1.13	1.25	4.13E-12	1.1884	4.13E-12	1.1884	0.7691	0
10	119081666	rs11198760	A	0.4836	1.101	0.8415	1.44	1.70E-08	1.15	1.1	1.21	3.45E-08	1.1483	3.45E-08	1.1483	0.7547	0
10	119082698	rs11198762	C	0.362	1.137	0.8627	1.499	2.61E-10	1.17	1.12	1.24	4.94E-10	1.1689	4.94E-10	1.1689	0.8416	0
10	119085108	rs10749286	T	0.4344	1.113	0.8515	1.453	1.10E-09	1.16	1.11	1.22	1.05E-09	1.1585	1.05E-09	1.1585	0.7654	0
10	119086994	rs11198765	T	0.4344	0.8989	0.688	1.174	3.36E-08	0.87	0.83	0.91	3.55E-08	0.871	3.55E-08	0.871	0.8138	0
10	119087544	rs10886379	T	0.4437	1.11	0.8498	1.45	3.35E-08	1.15	1.1	1.21	3.24E-08	1.1486	3.24E-08	1.1486	0.7985	0
10	119088281	rs11198766	T	0.4344	1.113	0.8515	1.453	2.93E-08	1.15	1.1	1.21	3.17E-08	1.1487	3.17E-08	1.1487	0.8137	0
10	119088762	rs12249648	T	0.4344	1.113	0.8515	1.453	3.05E-08	1.15	1.1	1.21	3.17E-08	1.1487	3.17E-08	1.1487	0.8137	0
10	119088838	rs35588588	A	0.4344	0.8989	0.688	1.174	2.84E-08	0.87	0.83	0.91	3.55E-08	0.871	3.55E-08	0.871	0.8138	0
10	119091119	rs10886380	A	0.3335	1.148	0.8676	1.52	5.05E-09	1.16	1.1	1.22	2.77E-09	1.1596	2.77E-09	1.1596	0.943	0
10	119092299	rs2420491	T	0.4429	0.8966	0.6785	1.185	2.18E-08	0.86	0.82	0.91	6.62E-09	0.8612	6.62E-09	0.8612	0.7732	0
10	119093142	rs10886381	T	0.327	0.8691	0.6565	1.151	4.60E-09	0.86	0.82	0.91	1.33E-09	0.8603	1.33E-09	0.8603	0.9423	0
10	119097510	rs11198768	T	0.3348	0.8709	0.6576	1.153	3.23E-09	0.86	0.82	0.91	1.35E-09	0.8603	1.35E-09	0.8603	0.931	0
10	119098296	rs9943375	T	0.2445	1.182	0.8919	1.567	8.90E-10	1.16	1.11	1.22	1.09E-09	1.1606	1.09E-09	1.1606	0.8975	0
10	119098833	rs7097835	T	0.3687	0.8798	0.6655	1.163	1.14E-08	0.86	0.82	0.91	3.86E-09	0.8606	3.86E-09	0.8606	0.875	0
10	119102716	rs7908170	T	0.2776	0.8555	0.6455	1.134	1.75E-09	0.86	0.82	0.9	6.51E-10	0.8599	6.51E-10	0.8599	0.9713	0
10	119103029	rs7911636	A	0.2776	0.8555	0.6455	1.134	2.00E-09	0.86	0.82	0.9	7.57E-10	0.8599	7.57E-10	0.8599	0.9713	0
10	119105089	rs2271359	A	0.2982	0.8613	0.6502	1.141	1.97E-09	0.86	0.82	0.9	7.95E-10	0.86	7.95E-10	0.86	0.9917	0
10	119105675	rs11198775	A	0.2982	0.8613	0.6502	1.141	1.99E-09	0.86	0.82	0.9	7.95E-10	0.86	7.95E-10	0.86	0.9917	0
10	119108381	rs11198776	A	0.2982	0.8613	0.6502	1.141	1.70E-09	0.86	0.82	0.9	7.95E-10	0.86	7.95E-10	0.86	0.9917	0
10	119109449	rs10886384	T	0.3482	0.8739	0.6594	1.158	6.19E-08	0.87	0.83	0.92	2.66E-08	0.8701	2.66E-08	0.8701	0.9755	0
10	119110295	rs10886386	T	0.392	1.129	0.8551	1.491	7.98E-08	1.15	1.09	1.21	3.30E-08	1.1493	3.30E-08	1.1493	0.8982	0
10	119110322	rs6585534	T	0.2686	0.8532	0.6441	1.13	8.98E-10	0.86	0.82	0.9	6.36E-10	0.8598	6.36E-10	0.8598	0.9565	0
10	119113006	rs11198779	T	0.2982	0.8613	0.6502	1.141	1.71E-09	0.86	0.82	0.9	9.24E-10	0.86	9.24E-10	0.86	0.9917	0
10	119114555	rs7906373	A	0.3115	0.865	0.6532	1.145	6.09E-09	0.87	0.83	0.91	1.02E-08	0.8699	1.02E-08	0.8699	0.9684	0
10	119114649	rs67965895	A	0.2982	0.8613	0.6502	1.141	3.89E-09	0.86	0.82	0.91	7.95E-10	0.86	7.95E-10	0.86	0.9917	0
10	119114839	rs34795123	A	0.3754	0.8806	0.6648	1.166	7.85E-09	0.86	0.82	0.91	3.91E-09	0.8606	3.91E-09	0.8606	0.871	0

10	119115290	rs17098456	T	0.2804	1.168	0.881	1.548	3.21E-09	1.16	1.1	1.21	1.03E-09	1.1602	1.03E-09	1.1602	0.9625	0
10	119117332	rs12774592	A	0.2788	0.8566	0.6473	1.134	4.87E-09	0.87	0.82	0.91	9.45E-09	0.8696	9.45E-09	0.8696	0.9148	0
10	119117860	rs11198785	A	0.2166	0.8376	0.6323	1.109	2.63E-09	0.86	0.82	0.91	6.44E-10	0.8593	6.44E-10	0.8593	0.8561	0
10	119128477	rs34887065	A	0.058	0.7611	0.574	1.009	2.63E-07	0.87	0.83	0.92	2.05E-08	0.8663	2.05E-08	0.8663	0.3608	0
10	119137551	rs4751697	T	0.08487	1.28	0.9666	1.695	1.21E-07	1.15	1.09	1.2	1.54E-08	1.1538	1.54E-08	1.1538	0.462	0
10	119137967	rs34179455	A	0.08259	0.7797	0.5887	1.033	1.49E-07	0.87	0.83	0.92	1.70E-08	0.867	1.70E-08	0.867	0.4519	0
10	119138226	rs10886402	A	0.058	0.7618	0.575	1.009	1.77E-07	0.87	0.83	0.92	1.62E-08	0.8664	1.62E-08	0.8664	0.3624	0
10	119143701	rs11198803	T	0.04649	1.335	1.005	1.774	1.62E-07	1.14	1.09	1.2	4.17E-08	1.1452	0.001367	1.1565	0.2833	13.15
11	723429	rs4078520	T	0.02439	1.342	1.039	1.735	7.65E-15	1.22	1.16	1.28	4.90E-16	1.2242	4.90E-16	1.2242	0.4744	0
11	743813	rs10902218	T	0.02918	0.7533	0.5841	0.9717	4.06E-16	0.81	0.77	0.85	5.52E-17	0.8077	5.52E-17	0.8077	0.5838	0
11	745884	rs7936322	T	0.01257	1.391	1.073	1.802	4.13E-18	1.24	1.18	1.31	6.34E-19	1.245	6.34E-19	1.245	0.3928	0
11	749452	rs3901233	A	0.01927	1.363	1.052	1.767	1.00E-20	1.27	1.21	1.34	1.42E-21	1.2733	1.42E-21	1.2733	0.6001	0
11	750849	rs7945912	A	0.01071	0.7132	0.5502	0.9246	1.21E-17	0.81	0.77	0.85	2.69E-18	0.8064	2.69E-18	0.8064	0.3449	0
11	751726	rs3932434	T	0.07891	1.284	0.9716	1.696	1.91E-10	1.19	1.13	1.26	9.25E-11	1.1933	9.25E-11	1.1933	0.5995	0
11	752059	rs7478765	A	0.08266	0.7815	0.5916	1.032	2.82E-10	0.84	0.79	0.89	8.67E-11	0.8378	8.67E-11	0.8378	0.6179	0
11	754157	rs11246302	A	0.07954	0.7794	0.5899	1.03	2.52E-10	0.84	0.79	0.89	8.48E-11	0.8377	8.48E-11	0.8377	0.6051	0
11	758446	rs10902219	A	0.07206	1.291	0.9773	1.706	1.10E-10	1.2	1.13	1.26	1.02E-11	1.2032	1.02E-11	1.2032	0.6136	0
11	761525	rs11246306	T	0.07659	1.286	0.9735	1.7	4.83E-10	1.19	1.13	1.26	9.15E-11	1.1934	9.15E-11	1.1934	0.5923	0
11	762441	rs4963163	A	0.01108	0.713	0.5492	0.9256	3.55E-20	0.79	0.75	0.83	3.30E-21	0.7871	3.30E-21	0.7871	0.4497	0
11	762791	rs4963124	C	0.01958	1.423	1.058	1.913	3.93E-08	1.16	1.1	1.23	1.01E-08	1.1676	0.02344	1.2173	0.1831	43.59
11	766069	rs11602009	T	0.06841	0.7715	0.5837	1.02	2.48E-10	0.84	0.79	0.89	9.00E-11	0.8374	9.00E-11	0.8374	0.5577	0
11	767750	rs11606152	T	0.08169	1.281	0.9694	1.692	3.87E-10	1.19	1.13	1.26	1.09E-10	1.1933	1.09E-10	1.1933	0.6109	0
11	770007	rs7940065	T	0.007979	1.423	1.096	1.846	6.54E-18	1.24	1.18	1.3	4.93E-19	1.2459	1.81E-13	1.2487	0.3088	3.47
11	771034	rs7930569	A	0.007979	0.7029	0.5417	0.912	4.44E-18	0.8	0.77	0.85	2.76E-20	0.7964	2.76E-20	0.7964	0.3387	0
11	771716	rs3934992	A	0.01511	0.7255	0.56	0.9398	9.33E-22	0.79	0.75	0.83	1.24E-22	0.7877	1.24E-22	0.7877	0.5263	0
11	771986	rs28638097	A	0.08784	0.7849	0.5944	1.037	2.66E-10	0.84	0.79	0.89	1.04E-10	0.8379	1.04E-10	0.8379	0.639	0
11	772177	rs12800942	T	0.08715	1.275	0.9653	1.683	5.41E-10	1.19	1.13	1.26	1.13E-10	1.1931	1.13E-10	1.1931	0.6333	0
11	772490	rs12224894	A	0.01163	1.398	1.078	1.814	4.92E-21	1.28	1.21	1.34	7.76E-23	1.2841	7.76E-23	1.2841	0.5149	0
11	772701	rs12223324	A	0.01172	0.7156	0.5516	0.9283	1.64E-18	0.8	0.76	0.84	2.52E-20	0.797	2.52E-20	0.797	0.4093	0
11	774568	rs7951926	T	0.01459	1.383	1.066	1.794	1.07E-20	1.27	1.21	1.34	1.64E-21	1.274	1.64E-21	1.274	0.5284	0
11	774608	rs7952569	A	0.00857	1.418	1.093	1.84	7.12E-18	1.24	1.18	1.3	5.13E-19	1.2458	5.13E-19	1.2458	0.321	0
11	774982	rs7952095	A	0.02224	1.355	1.044	1.757	1.09E-16	1.23	1.17	1.29	1.12E-17	1.2341	1.12E-17	1.2341	0.4738	0
11	775275	rs7942564	A	0.008569	0.7052	0.5436	0.915	2.93E-18	0.8	0.77	0.84	2.08E-20	0.7966	2.08E-20	0.7966	0.3506	0
11	775423	rs7948070	T	0.00857	1.418	1.093	1.84	6.74E-18	1.24	1.18	1.3	5.13E-19	1.2458	5.13E-19	1.2458	0.321	0
11	775651	rs7948539	A	0.00857	1.418	1.093	1.84	4.22E-18	1.24	1.18	1.31	5.13E-19	1.2458	5.13E-19	1.2458	0.321	0
11	776279	rs11246309	A	0.05027	0.7593	0.5763	1	4.03E-11	0.84	0.79	0.88	2.27E-11	0.837	2.27E-11	0.837	0.4809	0

11	776496	rs10902220	T	0.09721	1.264	0.9584	1.666	5.41E-10	1.19	1.12	1.25	6.74E-11	1.1926	6.74E-11	1.1926	0.6747	0
11	779671	rs7111003	T	0.906	0.9827	0.7356	1.313	7.29E-10	0.84	0.79	0.89	3.17E-09	0.8449	6.91E-05	0.8497	0.2975	7.85
11	780395	rs56364586	T	0.7233	0.9479	0.7046	1.275	1.05E-08	0.84	0.79	0.89	1.94E-08	0.844	1.94E-08	0.844	0.4338	0
11	780410	rs6597984	T	0.121	1.233	0.9462	1.606	4.77E-15	1.22	1.16	1.28	1.14E-15	1.2204	1.14E-15	1.2204	0.9385	0
11	780827	rs4963156	T	0.04317	1.315	1.008	1.715	1.01E-21	1.29	1.22	1.36	6.57E-23	1.2909	6.57E-23	1.2909	0.8893	0
11	781076	rs12226463	T	0.08362	1.269	0.9689	1.662	1.98E-10	1.19	1.12	1.25	2.06E-11	1.1928	2.06E-11	1.1928	0.6466	0
11	782563	rs11246310	C	0.09252	1.259	0.9627	1.647	4.25E-11	1.19	1.13	1.26	2.57E-11	1.1925	2.57E-11	1.1925	0.6864	0
11	784340	rs7104929	C	0.004636	0.684	0.5259	0.8897	1.14E-23	0.77	0.73	0.81	4.96E-25	0.7667	4.96E-25	0.7667	0.3861	0
11	784775	rs7107271	T	0.02025	1.366	1.05	1.778	3.33E-15	1.22	1.16	1.28	2.76E-16	1.2247	2.76E-16	1.2247	0.4084	0
11	785219	rs28634261	T	0.02492	1.364	1.04	1.79	1.38E-10	1.19	1.13	1.25	1.24E-11	1.1959	1.24E-11	1.1959	0.3334	0
11	786577	rs9943674	A	0.03414	0.7454	0.5679	0.9783	1.15E-10	0.84	0.8	0.89	1.55E-11	0.8363	1.55E-11	0.8363	0.3978	0
11	786953	rs9943678	C	0.0339	0.7454	0.5681	0.9779	8.47E-11	0.84	0.8	0.89	1.54E-11	0.8363	1.54E-11	0.8363	0.3971	0
11	787679	rs7946354	T	0.02388	1.357	1.041	1.768	9.34E-16	1.22	1.17	1.29	3.05E-16	1.2244	3.05E-16	1.2244	0.4387	0
11	788007	rs6597982	A	0.03549	1.338	1.02	1.756	1.12E-10	1.19	1.13	1.25	1.49E-11	1.1951	1.49E-11	1.1951	0.4064	0
11	791462	rs4963153	A	0.01494	1.385	1.065	1.799	3.62E-26	1.3	1.24	1.37	5.12E-27	1.3028	5.12E-27	1.3028	0.6415	0
11	798865	rs12277141	C	0.2567	1.165	0.8949	1.516	2.70E-15	1.24	1.17	1.3	4.53E-16	1.2371	4.53E-16	1.2371	0.6489	0
11	799670	rs28649421	C	0.2626	1.162	0.8933	1.512	7.86E-16	1.24	1.18	1.31	4.66E-16	1.2369	4.66E-16	1.2369	0.6352	0
11	802115	rs7479101	A	0.2418	0.8542	0.6561	1.112	8.13E-14	0.82	0.78	0.86	4.49E-14	0.8213	4.49E-14	0.8213	0.7658	0
11	802379	rs10902221	T	0.1866	0.8387	0.646	1.089	3.90E-25	0.77	0.74	0.81	1.84E-26	0.7722	1.84E-26	0.7722	0.5281	0
11	802902	rs28360884	T	0.2567	0.8586	0.6597	1.117	1.25E-14	0.81	0.77	0.86	1.69E-15	0.8118	1.69E-15	0.8118	0.6707	0
11	803017	rs6597981	A	0.1812	0.8365	0.644	1.087	3.48E-26	0.77	0.73	0.81	1.06E-25	0.7722	1.06E-25	0.7722	0.542	0
11	804212	rs7104785	A	0.151	1.211	0.9325	1.573	6.53E-25	1.29	1.23	1.35	2.52E-25	1.2873	2.52E-25	1.2873	0.6413	0
11	805234	rs7484123	A	0.1563	1.208	0.9302	1.569	5.90E-25	1.3	1.23	1.36	5.99E-26	1.2967	5.99E-26	1.2967	0.5887	0
11	805302	rs28710291	A	0.2391	1.171	0.9003	1.523	2.09E-15	1.23	1.17	1.3	2.98E-15	1.2277	2.98E-15	1.2277	0.7193	0
11	805419	rs7484068	A	0.1469	1.213	0.9343	1.576	1.91E-25	1.3	1.24	1.36	3.62E-26	1.2969	3.62E-26	1.2969	0.6095	0
11	805435	rs28689676	A	0.2522	0.8573	0.6586	1.116	8.52E-15	0.81	0.77	0.86	1.67E-15	0.8117	1.67E-15	0.8117	0.679	0
11	805578	rs11246313	T	0.152	1.21	0.9321	1.572	6.87E-25	1.3	1.23	1.36	5.84E-26	1.2968	5.84E-26	1.2968	0.5969	0
11	805589	rs11246314	A	0.152	1.21	0.9321	1.572	7.27E-25	1.3	1.23	1.36	5.84E-26	1.2968	5.84E-26	1.2968	0.5969	0
11	805712	rs11246316	A	0.1469	0.8242	0.6347	1.07	1.31E-25	0.77	0.73	0.81	5.48E-26	0.7718	5.48E-26	0.7718	0.616	0
11	808423	rs28694634	A	0.2683	1.161	0.8915	1.511	3.48E-15	1.23	1.17	1.3	5.24E-15	1.2273	5.24E-15	1.2273	0.674	0
11	809666	rs72844798	A	0.2983	0.8532	0.6326	1.151	4.17E-09	0.83	0.78	0.88	1.93E-09	0.8309	1.93E-09	0.8309	0.8597	0
11	810882	rs10902222	T	0.2542	1.166	0.8954	1.519	2.60E-14	1.22	1.16	1.29	4.20E-14	1.2179	4.20E-14	1.2179	0.7418	0
11	812188	rs4131364	A	0.09641	1.249	0.9611	1.622	1.68E-22	1.27	1.21	1.34	2.00E-22	1.2693	2.00E-22	1.2693	0.9023	0
11	813264	rs28633403	A	0.1867	1.192	0.9185	1.546	1.08E-17	1.24	1.18	1.31	1.07E-17	1.2383	1.07E-17	1.2383	0.7703	0
11	813524	rs11246319	T	0.0935	1.251	0.963	1.624	3.70E-22	1.27	1.21	1.34	1.96E-22	1.2694	1.96E-22	1.2694	0.9116	0
11	815323	rs6597979	T	0.1167	1.233	0.9492	1.601	9.31E-23	1.28	1.22	1.34	1.08E-23	1.2784	1.08E-23	1.2784	0.7826	0

11	817286	rs6597978	C	0.2147	1.182	0.9074	1.541	8.36E-19	1.25	1.19	1.32	1.44E-18	1.2476	1.44E-18	1.2476	0.6842	0
11	817786	rs10902223	T	0.1684	1.206	0.9237	1.575	3.10E-17	1.25	1.18	1.31	4.90E-18	1.2484	4.90E-18	1.2484	0.7961	0
11	820268	rs7925131	A	0.2982	0.8643	0.6566	1.138	7.90E-12	0.83	0.79	0.87	7.43E-12	0.8312	7.43E-12	0.8312	0.7768	0
11	820754	rs61876744	T	0.1877	0.8361	0.6407	1.091	2.35E-15	0.82	0.78	0.86	3.79E-15	0.8205	3.79E-15	0.8205	0.8882	0
11	822622	rs7942159	A	0.1611	0.8266	0.6332	1.079	9.33E-20	0.8	0.76	0.84	4.50E-20	0.8008	4.50E-20	0.8008	0.8128	0
11	823809	rs1135628	C	0.4325	1.123	0.8402	1.502	5.60E-10	1.2	1.13	1.27	5.88E-10	1.197	5.88E-10	1.197	0.6608	0
11	825110	rs1138714	A	0.2031	0.8413	0.6447	1.098	1.32E-17	0.8	0.76	0.84	5.82E-18	0.8014	5.82E-18	0.8014	0.7159	0
11	825777	rs4963120	T	0.1169	1.236	0.9484	1.61	5.50E-18	1.25	1.19	1.31	1.50E-18	1.2495	1.50E-18	1.2495	0.9347	0
11	47641380	rs7120548	T	0.2768	0.8674	0.6713	1.121	1.91E-07	0.87	0.83	0.92	3.26E-08	0.8699	3.26E-08	0.8699	0.9821	0
11	95575690	rs11021221	A	0.6915	0.9208	0.6128	1.384	1.49E-09	1.23	1.15	1.31	2.21E-09	1.2209	0.2816	1.1444	0.1691	47.12
12	14135489	rs117522474	T	0.4336	0.6846	0.2651	1.768	4.09E-09	1.62	1.38	1.91	1.63E-08	1.5813	0.6539	1.2018	0.0794	67.51
12	14137583	rs17340879	T	0.4158	1.482	0.5746	3.821	1.77E-09	0.61	0.52	0.72	6.08E-09	0.6253	0.675	0.8367	0.0702	69.51
12	51360845	rs2241545	A	0.2668	1.2	0.8701	1.654	1.93E-09	1.18	1.12	1.25	1.07E-09	1.1805	1.07E-09	1.1805	0.9195	0
12	51362485	rs3782473	T	0.2384	1.214	0.8793	1.677	6.60E-10	1.19	1.12	1.25	1.94E-10	1.1907	1.94E-10	1.1907	0.9048	0
13	40475054	rs76513130	A	0.09227	0.7547	0.5439	1.047	7.89E-10	0.8	0.75	0.86	1.58E-10	0.7979	1.58E-10	0.7979	0.7331	0
13	40475570	rs11619643	T	0.1109	1.306	0.9406	1.813	3.05E-09	1.23	1.15	1.32	8.66E-10	1.2331	8.66E-10	1.2331	0.7259	0
13	40477248	rs2015989	A	0.07628	1.327	0.9706	1.813	3.05E-07	1.18	1.1	1.25	3.83E-08	1.1852	3.83E-08	1.1852	0.4702	0
13	40484109	rs7982453	A	0.0383	0.7281	0.5393	0.983	1.02E-07	0.86	0.81	0.91	1.75E-08	0.8553	0.0005431	0.847	0.2848	12.57
13	40484560	rs17068690	T	0.03175	0.719	0.5321	0.9716	5.93E-08	0.86	0.81	0.91	1.62E-08	0.855	0.00501	0.838	0.2515	23.94
13	40487691	rs9603762	T	0.05518	0.7304	0.5297	1.007	3.78E-10	0.8	0.75	0.86	3.65E-11	0.7968	3.65E-11	0.7968	0.5871	0
13	40489271	rs9603763	A	0.04709	1.387	1.004	1.917	4.59E-10	1.24	1.16	1.33	1.06E-10	1.2459	1.06E-10	1.2459	0.5062	0
13	40491476	rs9603764	A	0.0501	1.382	0.9999	1.909	1.77E-09	1.23	1.15	1.32	5.91E-10	1.2362	5.91E-10	1.2362	0.4897	0
13	40492476	rs9603765	T	0.07136	1.348	0.9743	1.866	1.19E-08	1.23	1.15	1.33	4.05E-09	1.2353	4.05E-09	1.2353	0.5896	0
13	40493853	rs61963660	T	0.04514	1.391	1.007	1.922	3.36E-10	1.25	1.16	1.34	3.66E-11	1.2558	3.66E-11	1.2558	0.5262	0
13	40496004	rs2721056	T	0.0001504	1.781	1.321	2.4	1.78E-13	1.28	1.2	1.37	2.17E-15	1.3001	0.01924	1.4603	0.0343	77.69
13	40496005	rs2721055	T	0.0002449	0.5731	0.4257	0.7717	4.58E-12	0.78	0.73	0.84	1.52E-14	0.7678	0.01436	0.6927	0.048	74.43
13	40496260	rs2755227	T	0.0006844	0.5971	0.4434	0.8041	1.01E-12	0.78	0.73	0.84	8.74E-15	0.7698	0.007311	0.7109	0.0861	66.05
13	40498364	rs2701852	T	0.0003027	1.722	1.282	2.314	1.01E-10	1.23	1.16	1.31	2.45E-12	1.2485	0.03772	1.4094	0.0288	79.07
13	40499289	rs2802497	A	0.0005772	0.5923	0.4396	0.7981	2.83E-13	0.77	0.72	0.83	1.57E-15	0.7597	0.004792	0.7041	0.0929	64.57
13	40500329	rs2802499	A	0.0005773	1.688	1.253	2.275	6.48E-14	1.3	1.22	1.4	9.16E-16	1.3174	0.004536	1.4205	0.0943	64.27
13	40500420	rs2721060	A	0.0005773	1.688	1.253	2.275	1.86E-13	1.3	1.22	1.4	3.66E-15	1.3182	0.004535	1.4207	0.0948	64.18
13	40502273	rs2802501	A	0.003908	0.6657	0.5049	0.8776	1.64E-10	0.82	0.78	0.87	2.07E-12	0.8125	0.006098	0.7732	0.1483	52.15
13	40504531	rs2721043	A	1.72E-05	2.049	1.477	2.842	1.22E-27	1.54	1.42	1.66	3.00E-31	1.5635	9.21E-05	1.6964	0.0959	63.93
13	40506187	rs2701886	T	0.0004239	1.714	1.27	2.312	7.06E-16	1.34	1.24	1.43	1.88E-18	1.3573	0.001213	1.4491	0.1168	59.35
13	40507208	rs2802503	A	0.000387	1.72	1.275	2.32	8.04E-16	1.34	1.25	1.43	2.62E-18	1.3577	0.001386	1.4526	0.1118	60.46
13	40507273	rs9549229	T	0.002428	0.6581	0.5021	0.8625	7.72E-08	0.87	0.82	0.91	1.50E-08	0.8612	0.06975	0.7819	0.0471	74.63

13	40508349	rs2701885	T	0.0003869	0.5815	0.4311	0.7845	2.44E-16	0.75	0.69	0.8	7.15E-18	0.7401	0.001895	0.6897	0.105	61.95
13	40508360	rs2701884	A	0.0004239	1.714	1.27	2.312	1.17E-16	1.34	1.25	1.44	1.29E-18	1.3571	0.001212	1.449	0.1167	59.38
13	40510104	rs1923254	A	0.002428	1.52	1.159	1.992	9.49E-08	1.15	1.1	1.22	1.35E-08	1.1618	0.06911	1.2794	0.0473	74.6
13	40511386	rs2802505	A	0.0004239	0.5836	0.4325	0.7873	1.19E-16	0.75	0.7	0.8	3.59E-18	0.7404	0.001675	0.6914	0.1098	60.9
13	40513375	rs1923255	A	0.0003651	0.5803	0.4302	0.7827	9.00E-17	0.74	0.69	0.8	1.81E-19	0.7307	0.000821	0.6857	0.1211	58.39
13	40514110	rs2701879	A	0.0006596	0.5936	0.4397	0.8014	2.03E-16	0.75	0.7	0.8	6.62E-18	0.741	0.0008328	0.6996	0.1368	54.81
13	40514445	rs2802506	T	0.000387	1.72	1.275	2.32	2.43E-16	1.34	1.25	1.44	1.23E-18	1.3573	0.001385	1.4525	0.1116	60.51
13	40514469	rs2802507	T	0.000387	1.72	1.275	2.32	2.48E-16	1.34	1.25	1.44	1.23E-18	1.3573	0.001385	1.4525	0.1116	60.51
13	40516471	rs9532556	A	0.002321	0.6573	0.5018	0.8611	2.51E-08	0.86	0.82	0.91	1.30E-09	0.8515	0.05331	0.7778	0.0554	72.75
13	40517172	rs2701878	T	0.0008552	0.6092	0.4553	0.8152	1.15E-11	0.81	0.76	0.86	7.09E-13	0.8	0.02153	0.7289	0.0608	71.55
13	40519287	rs7328015	T	0.03254	0.7537	0.5816	0.9768	4.28E-10	0.86	0.82	0.9	4.40E-11	0.8564	4.40E-11	0.8564	0.3264	0
13	40523376	rs2701881	C	0.0004331	0.5865	0.4358	0.7895	1.49E-16	0.74	0.69	0.8	2.37E-19	0.731	0.0004979	0.6903	0.1356	55.1
13	40525562	rs2755229	A	0.07326	0.7901	0.6105	1.022	1.24E-07	0.88	0.84	0.92	1.85E-08	0.877	1.85E-08	0.877	0.4203	0
13	40525659	rs2701877	A	0.0004098	0.5842	0.4336	0.7871	2.77E-16	0.75	0.7	0.8	5.03E-18	0.7403	0.001619	0.6915	0.1098	60.89
13	40527037	rs2701876	T	0.05564	0.7781	0.6017	1.006	1.46E-07	0.88	0.84	0.92	2.99E-08	0.8764	2.99E-08	0.8764	0.3559	0
13	40527619	rs2701874	A	0.05564	0.7781	0.6017	1.006	1.32E-07	0.88	0.84	0.92	2.99E-08	0.8764	2.99E-08	0.8764	0.3559	0
13	40527751	rs2755230	T	0.0004098	0.5842	0.4336	0.7871	3.85E-17	0.74	0.69	0.79	2.33E-19	0.7309	0.000605	0.6886	0.1303	56.31
13	40529478	rs2755231	A	0.0003093	1.707	1.276	2.282	9.99E-14	1.27	1.19	1.36	1.26E-15	1.2871	0.01435	1.4212	0.0512	73.69
13	40530473	rs2701873	T	0.06767	1.271	0.9827	1.644	7.03E-08	1.14	1.09	1.19	1.55E-08	1.1441	1.55E-08	1.1441	0.4152	0
13	40530535	rs2701872	T	0.0004098	0.5842	0.4336	0.7871	5.46E-18	0.73	0.68	0.79	6.02E-21	0.7216	0.0001633	0.6859	0.1538	50.83
13	40531175	rs2755233	T	0.0007641	1.647	1.232	2.202	1.03E-14	1.28	1.2	1.37	2.28E-16	1.2946	0.005362	1.3929	0.0965	63.81
13	40531643	rs2755234	A	0.08551	1.253	0.969	1.621	1.34E-07	1.14	1.08	1.19	1.53E-08	1.1435	1.53E-08	1.1435	0.4786	0
13	40532362	rs2755235	A	0.07182	1.267	0.9792	1.639	9.74E-08	1.14	1.08	1.19	1.40E-08	1.1439	1.40E-08	1.1439	0.4288	0
13	40533278	rs2755236	A	0.0004299	1.692	1.263	2.268	1.37E-14	1.28	1.21	1.37	3.07E-16	1.2962	0.009464	1.4166	0.068	69.98
13	40534903	rs2721052	T	0.01352	1.378	1.068	1.777	4.36E-09	1.15	1.1	1.21	4.92E-10	1.1568	0.01823	1.2034	0.1705	46.77
13	40535292	rs2755237	A	0.0006284	0.617	0.4678	0.8138	2.95E-21	0.74	0.7	0.79	1.28E-23	0.7336	5.04E-06	0.7118	0.2091	36.62
13	40536133	rs2755238	T	6.02E-05	0.5366	0.3959	0.7274	1.49E-34	0.63	0.59	0.68	6.72E-39	0.6246	2.23E-35	0.624	0.3143	1.23
13	40536747	rs2721051	T	5.21E-05	1.873	1.382	2.539	5.71E-35	1.57	1.46	1.69	3.28E-38	1.5847	3.55E-15	1.6079	0.2681	18.45
13	40536785	rs74948688	T	2.28E-06	2.519	1.717	3.695	5.50E-31	1.79	1.62	1.98	3.06E-35	1.8284	1.64E-05	2.0154	0.0906	65.09
13	40540435	rs79728429	T	2.44E-07	3.044	1.995	4.645	5.06E-26	1.75	1.58	1.94	1.91E-30	1.8063	0.003625	2.2188	0.0127	83.91
13	40541449	rs2701857	A	5.81E-05	0.5359	0.3954	0.7263	2.16E-34	0.63	0.59	0.68	1.01E-38	0.6245	7.04E-32	0.6233	0.3108	2.67
13	40543978	rs1059246	C	0.1709	0.8354	0.6457	1.081	2.20E-08	0.87	0.83	0.92	4.00E-09	0.8688	4.00E-09	0.8688	0.7614	0
13	40545329	rs11616662	A	6.19E-05	1.862	1.374	2.524	8.79E-31	1.51	1.41	1.62	5.89E-34	1.526	2.39E-07	1.588	0.1882	42.24
13	40545810	rs7331857	C	0.1709	0.8354	0.6457	1.081	2.57E-08	0.87	0.83	0.92	4.00E-09	0.8688	4.00E-09	0.8688	0.7614	0
13	40550515	rs2701891	T	0.04492	1.303	1.006	1.687	4.42E-08	1.14	1.09	1.19	7.73E-09	1.1448	7.73E-09	1.1448	0.3187	0
13	40550820	rs2701892	T	0.04492	1.303	1.006	1.687	4.68E-08	1.14	1.09	1.19	7.73E-09	1.1448	7.73E-09	1.1448	0.3187	0

13	40551499	rs2701893	T	0.04492	1.303	1.006	1.687	3.61E-08	1.14	1.09	1.19	7.73E-09	1.1448	7.73E-09	1.1448	0.3187	0
13	40552895	rs2701895	A	0.04291	1.307	1.009	1.693	3.13E-08	1.14	1.09	1.2	7.58E-09	1.1449	2.65E-06	1.1475	0.3085	3.59
13	40553755	rs2701896	C	0.1612	1.203	0.929	1.557	1.79E-08	1.14	1.09	1.2	1.27E-08	1.1419	1.27E-08	1.1419	0.6879	0
13	40563647	rs80070740	A	1.22E-05	1.964	1.451	2.657	1.66E-29	1.52	1.42	1.64	7.41E-33	1.5417	2.95E-05	1.6539	0.1064	61.63
13	40563667	rs2755209	A	0.05779	0.7797	0.6029	1.008	5.37E-08	0.88	0.84	0.92	1.65E-08	0.8766	1.65E-08	0.8766	0.3641	0
13	40699382	rs113204272	T	3.15E-06	2.206	1.582	3.077	7.55E-18	1.46	1.34	1.59	1.32E-21	1.4979	0.006733	1.7371	0.0186	81.95
13	41285528	rs9566738	T	3.27E-05	2.135	1.493	3.053	2.66E-07	1.25	1.15	1.36	2.48E-09	1.2863	0.08285	1.5863	0.0043	87.71
13	41286756	rs9532773	A	0.0001206	0.5006	0.3518	0.7123	1.02E-06	0.83	0.76	0.89	4.98E-08	0.8111	0.1035	0.6646	0.0061	86.72
13	41289525	rs9566739	T	3.27E-05	2.135	1.493	3.053	4.42E-07	1.24	1.14	1.36	7.35E-09	1.2766	0.0901	1.5807	0.0038	88.07
13	41291189	rs9566740	T	3.27E-05	0.4684	0.3275	0.67	1.36E-08	0.79	0.73	0.86	8.29E-11	0.7702	0.07214	0.627	0.0052	87.18
13	41304902	rs34943826	A	3.54E-05	0.4698	0.3284	0.672	2.35E-08	0.8	0.73	0.86	5.11E-10	0.7798	0.08231	0.6316	0.0045	87.63
13	41309992	rs34250367	A	3.54E-05	2.129	1.488	3.045	1.07E-08	1.26	1.17	1.37	1.38E-10	1.2919	0.07544	1.5891	0.0051	87.26
13	41314475	rs9566741	C	3.54E-05	0.4698	0.3284	0.672	6.72E-09	0.79	0.73	0.85	6.69E-11	0.7706	0.07152	0.6281	0.0055	87.02
13	41324011	rs9562287	T	4.42E-05	0.4764	0.3338	0.68	1.98E-09	0.78	0.72	0.85	8.06E-12	0.7617	0.0581	0.6292	0.008	85.76
13	41324269	rs9566743	A	3.54E-05	0.4698	0.3284	0.672	1.86E-09	0.78	0.72	0.85	7.67E-12	0.7614	0.06134	0.6246	0.0068	86.37
13	41333690	rs11620416	A	4.42E-05	0.4764	0.3338	0.68	3.26E-09	0.79	0.72	0.85	7.01E-11	0.7709	0.06822	0.6327	0.0066	86.47
13	41338054	rs9562289	T	0.0001417	0.504	0.3542	0.7173	2.24E-08	0.81	0.76	0.87	2.23E-10	0.7946	0.07718	0.6602	0.0098	85
13	41340837	rs9566744	T	0.0001294	1.992	1.4	2.834	2.48E-08	1.23	1.14	1.32	4.44E-10	1.2544	0.08171	1.5157	0.0087	85.47
13	41344344	rs9566750	A	9.09E-05	2.04	1.428	2.916	8.40E-09	1.27	1.17	1.37	5.89E-11	1.2993	0.05916	1.557	0.0112	84.47
13	41345739	rs9562290	A	7.35E-05	2.068	1.444	2.962	1.17E-08	1.26	1.17	1.37	2.10E-10	1.2902	0.06901	1.5631	0.0084	85.63
13	41349325	rs9566751	A	7.35E-05	0.4835	0.3376	0.6925	2.73E-07	0.8	0.74	0.87	2.90E-09	0.7791	0.07589	0.6418	0.0075	86.01
13	41354457	rs9562292	A	7.35E-05	2.068	1.444	2.962	4.09E-07	1.24	1.14	1.36	9.13E-09	1.274	0.08315	1.5524	0.0066	86.44
13	41359328	rs9566753	T	3.30E-05	0.4687	0.3278	0.6703	4.14E-07	0.8	0.74	0.87	2.33E-09	0.7775	0.08269	0.6306	0.0044	87.69
13	41359501	rs9566754	A	4.45E-05	0.4767	0.334	0.6803	1.22E-06	0.82	0.75	0.89	3.32E-08	0.7977	0.1021	0.6434	0.0036	88.2
13	41361114	rs3829396	A	4.45E-05	2.098	1.47	2.994	3.97E-08	1.25	1.16	1.36	5.76E-10	1.282	0.07887	1.5713	0.0054	87.09
13	41363654	rs9562293	T	0.0001089	0.4986	0.3505	0.7093	7.83E-07	0.83	0.77	0.89	1.67E-08	0.8125	0.1044	0.6631	0.0055	87.01
13	41364148	rs9562294	T	3.22E-05	0.4681	0.3273	0.6695	1.51E-08	0.79	0.73	0.86	8.93E-11	0.7701	0.07228	0.6268	0.0052	87.2
13	41366461	rs9566755	A	3.12E-05	0.4698	0.3293	0.6704	1.16E-06	0.82	0.75	0.89	2.98E-08	0.7971	0.1052	0.6383	0.0028	88.83
13	41368916	rs9562295	A	3.12E-05	0.4698	0.3293	0.6704	6.42E-08	0.8	0.74	0.87	2.61E-10	0.7803	0.0821	0.6313	0.0042	87.82
13	41373989	rs9566757	A	2.64E-05	0.4644	0.3247	0.6641	5.53E-07	0.8	0.74	0.88	2.31E-09	0.777	0.08469	0.6274	0.0037	88.1
13	41376403	rs9315835	T	2.64E-05	2.153	1.506	3.08	2.40E-08	1.26	1.16	1.37	1.71E-10	1.2933	0.07784	1.5992	0.0042	87.8
13	41386494	rs11620020	T	2.24E-05	0.4615	0.3228	0.6598	1.01E-06	0.8	0.73	0.87	1.28E-08	0.7735	0.08587	0.625	0.0035	88.29
13	41387039	rs9562297	A	3.07E-05	2.139	1.496	3.058	8.40E-07	1.23	1.13	1.34	9.55E-09	1.2648	0.0981	1.5765	0.0031	88.56
13	41388861	rs3923988	A	7.18E-05	0.4896	0.3441	0.6965	2.23E-06	0.82	0.76	0.89	2.01E-08	0.7995	0.09584	0.6528	0.0052	87.2
13	41393893	rs9566761	A	0.001305	1.821	1.264	2.625	9.55E-08	1.23	1.14	1.33	4.81E-09	1.2504	0.05961	1.4345	0.0395	76.42
13	41411018	rs11616683	A	0.0009869	0.5423	0.3768	0.7804	1.69E-07	0.81	0.75	0.88	4.13E-09	0.7959	0.05934	0.6905	0.0347	77.58

13	73036446	rs13378884	A	0.2798	0.8501	0.6333	1.141	5.72E-08	0.86	0.82	0.91	2.85E-08	0.8597	2.85E-08	0.8597	0.9396	0
13	73039791	rs55761542	T	0.2613	0.8444	0.6287	1.134	9.39E-08	0.86	0.82	0.91	2.18E-08	0.8595	2.18E-08	0.8595	0.9048	0
13	73050852	rs7325460	A	0.2591	0.8571	0.6556	1.12	1.23E-07	0.87	0.83	0.92	4.96E-08	0.8695	4.96E-08	0.8695	0.9145	0
13	73059913	rs73220641	T	0.5525	1.089	0.8224	1.441	4.86E-12	1.2	1.14	1.26	6.26E-12	1.1961	6.26E-12	1.1961	0.5048	0
13	73063319	rs9592879	C	0.5903	1.08	0.8155	1.431	3.05E-12	1.2	1.14	1.26	4.66E-12	1.1959	4.66E-12	1.1959	0.4702	0
13	73065233	rs9530143	A	0.5802	1.083	0.8173	1.434	3.14E-12	1.2	1.14	1.26	4.55E-12	1.196	4.55E-12	1.196	0.4816	0
13	73070755	rs17285550	A	0.6048	0.9284	0.7007	1.23	2.84E-12	0.83	0.79	0.88	7.40E-13	0.8329	7.40E-13	0.8329	0.4426	0
13	73071858	rs4885062	T	0.4261	0.8925	0.6746	1.181	1.47E-11	0.84	0.79	0.88	3.16E-11	0.8417	3.16E-11	0.8417	0.6763	0
13	73072534	rs73220655	A	0.3636	0.8788	0.665	1.161	1.34E-11	0.84	0.79	0.88	3.26E-11	0.8413	3.26E-11	0.8413	0.7549	0
13	73075014	rs45597035	A	0.3475	0.8761	0.6649	1.154	2.68E-10	0.85	0.81	0.89	1.66E-10	0.8508	1.66E-10	0.8508	0.8326	0
15	50857052	rs35439349	A	0.5644	0.9116	0.6653	1.249	5.22E-08	0.85	0.8	0.9	2.70E-08	0.8519	2.70E-08	0.8519	0.6684	0
15	50867638	rs12916334	C	0.5876	0.9167	0.6694	1.255	3.20E-08	0.85	0.8	0.9	3.11E-08	0.8521	3.11E-08	0.8521	0.6432	0
15	50868599	rs12902196	C	0.5876	0.9167	0.6694	1.255	3.60E-08	0.85	0.8	0.9	2.79E-08	0.8521	2.79E-08	0.8521	0.6431	0
15	50869182	rs7166600	T	0.5876	0.9167	0.6694	1.255	2.69E-08	0.85	0.8	0.9	2.79E-08	0.8521	2.79E-08	0.8521	0.6431	0
15	51003737	rs3825799	A	0.2852	1.159	0.8841	1.52	1.16E-07	1.14	1.08	1.19	3.44E-08	1.1406	3.44E-08	1.1406	0.9063	0
15	51011792	rs11856609	T	0.3158	0.8709	0.6647	1.141	3.80E-09	0.87	0.83	0.91	3.89E-09	0.87	3.89E-09	0.87	0.9941	0
15	51031780	rs7170570	T	0.2936	1.156	0.8819	1.516	3.17E-08	1.14	1.09	1.2	3.96E-08	1.1405	3.96E-08	1.1405	0.9208	0
15	51038501	rs34590712	A	0.3028	1.153	0.8797	1.51	3.53E-08	1.14	1.09	1.2	3.14E-08	1.1404	3.14E-08	1.1404	0.9354	0
15	51043166	rs12900916	A	0.2936	0.865	0.6598	1.134	2.80E-08	0.87	0.83	0.92	5.67E-09	0.8698	5.67E-09	0.8698	0.9672	0
15	51063914	rs12899586	T	0.3585	1.135	0.8662	1.487	1.49E-09	1.16	1.1	1.21	3.48E-10	1.1593	3.48E-10	1.1593	0.8763	0
15	51065831	rs16953046	T	0.3585	0.8811	0.6725	1.154	1.57E-09	0.87	0.83	0.91	3.68E-09	0.8703	3.68E-09	0.8703	0.9278	0
15	51071674	rs9652426	T	0.3163	1.148	0.8762	1.505	8.19E-10	1.16	1.1	1.21	2.69E-10	1.1597	2.69E-10	1.1597	0.9408	0
15	51072532	rs11634895	A	0.3163	0.8709	0.6646	1.141	7.88E-10	0.86	0.82	0.91	1.41E-10	0.8603	1.41E-10	0.8603	0.9283	0
15	51076250	rs8026140	T	0.3484	0.8785	0.6702	1.152	2.15E-09	0.87	0.83	0.91	4.17E-09	0.8702	4.17E-09	0.8702	0.9447	0
15	51077741	rs7172283	T	0.2903	0.8638	0.6585	1.133	9.06E-09	0.87	0.83	0.91	4.25E-09	0.8698	4.25E-09	0.8698	0.9594	0
15	51082814	rs11636974	T	0.3484	0.8785	0.6702	1.152	6.18E-09	0.87	0.83	0.91	3.60E-09	0.8702	3.60E-09	0.8702	0.9447	0
15	67146248	rs750766	T	0.04844	1.308	1.002	1.709	6.68E-12	0.85	0.81	0.89	1.69E-10	0.8609	0.8809	1.0326	0.0018	89.71
15	67146869	rs2289263	T	0.07785	0.7875	0.6038	1.027	6.30E-14	1.2	1.14	1.26	9.36E-13	1.1846	0.9727	0.9928	0.0022	89.32
15	67147291	rs62006055	A	0.0543	0.7471	0.5551	1.005	1.01E-12	1.24	1.17	1.31	2.74E-11	1.2165	0.948	0.9837	0.001	90.71
15	67148918	rs7167532	T	0.3365	1.165	0.8534	1.59	3.21E-21	0.75	0.71	0.8	2.38E-20	0.7615	0.6635	0.9093	0.0064	86.55
15	67149528	rs2033785	C	0.3638	0.8664	0.6359	1.181	1.91E-21	1.33	1.25	1.41	4.76E-20	1.3103	0.6415	1.104	0.0077	85.94
15	67150217	rs11637816	A	0.3732	1.151	0.8446	1.569	5.67E-21	0.75	0.71	0.8	2.14E-20	0.7613	0.6322	0.9034	0.0077	85.9
15	67152409	rs10152544	T	0.8642	1.022	0.7937	1.317	1.16E-10	0.86	0.82	0.9	3.94E-10	0.8648	0.1255	0.8948	0.1885	42.17
15	67154447	rs744910	A	0.9456	1.009	0.7828	1.3	1.01E-10	0.86	0.82	0.9	3.50E-10	0.8644	0.04867	0.8854	0.2248	32.13
15	67155069	rs35533195	C	0.3765	1.151	0.8432	1.57	9.06E-23	0.75	0.71	0.8	2.85E-22	0.7603	0.6311	0.9031	0.0079	85.84
15	67155114	rs11634793	T	0.9137	1.014	0.7872	1.306	7.81E-11	0.86	0.82	0.9	3.67E-10	0.8646	0.07615	0.8892	0.2098	36.43

15	67155557	rs12441344	A	0.5421	0.9062	0.6602	1.244	4.84E-19	1.32	1.24	1.41	1.10E-17	1.3021	0.5064	1.1309	0.0223	80.84
15	67156801	rs11637537	C	0.4527	1.127	0.8251	1.539	5.17E-23	0.75	0.71	0.8	3.17E-22	0.7598	0.5711	0.8922	0.0117	84.26
15	67159616	rs4601989	T	0.4527	1.127	0.8251	1.539	6.39E-23	0.75	0.71	0.8	3.17E-22	0.7598	0.5711	0.8922	0.0117	84.26
15	67164997	rs1065080	A	0.5622	0.882	0.5769	1.348	8.47E-09	1.2	1.13	1.28	3.48E-08	1.192	0.445	1.1084	0.1597	49.41
15	67166592	rs7179840	T	0.3085	1.152	0.8775	1.512	3.22E-09	0.86	0.82	0.91	8.58E-09	0.8679	0.7978	0.9641	0.0382	76.72
15	67167671	rs11638064	A	0.3536	1.156	0.8509	1.571	1.40E-23	0.75	0.71	0.79	3.27E-22	0.7606	0.6456	0.906	0.0065	86.49
15	67168419	rs11639295	T	0.7949	1.038	0.7819	1.379	4.70E-14	0.82	0.78	0.87	6.53E-14	0.826	0.2603	0.8838	0.1088	61.12
15	67168973	rs7183244	T	0.3263	0.8725	0.6644	1.146	3.67E-10	1.17	1.12	1.23	3.04E-09	1.159	0.7685	1.043	0.0378	76.81
15	67169660	rs7165382	C	0.6579	0.9328	0.6854	1.269	8.66E-24	1.33	1.26	1.41	2.22E-22	1.315	0.4165	1.152	0.0264	79.71
15	67172207	rs34708051	T	0.4015	1.138	0.8414	1.539	1.88E-24	0.75	0.71	0.79	1.47E-22	0.7604	0.6057	0.8988	0.0078	85.89
15	67172982	rs1866320	T	0.4015	1.138	0.8414	1.539	5.97E-24	0.75	0.71	0.79	1.47E-22	0.7604	0.6057	0.8988	0.0078	85.89
15	67174805	rs12912010	T	0.244	1.2	0.8828	1.632	1.99E-26	0.73	0.69	0.78	4.43E-25	0.7423	0.7158	0.9139	0.0018	89.7
15	67174959	rs12912045	T	0.2767	1.185	0.8726	1.61	4.57E-26	0.73	0.69	0.78	2.61E-25	0.742	0.6879	0.9077	0.0023	89.23
15	67175169	rs12913547	T	0.2767	0.8437	0.6211	1.146	2.61E-26	1.36	1.29	1.44	3.26E-24	1.3383	0.6939	1.098	0.0027	88.91
15	67175203	rs12708492	T	0.5395	0.9245	0.7196	1.188	1.23E-09	1.16	1.11	1.22	5.40E-09	1.1507	0.5189	1.0722	0.0814	67.07
15	67176187	rs35251008	A	0.698	0.9429	0.7006	1.269	1.86E-14	1.23	1.17	1.3	2.18E-13	1.2196	0.3585	1.1228	0.0843	66.44
15	67177796	rs55994097	T	0.3902	1.118	0.8668	1.442	5.11E-13	0.82	0.78	0.87	1.79E-12	0.8305	0.6492	0.9329	0.0194	81.69
15	67183980	rs6494636	C	0.6647	1.059	0.8184	1.369	1.89E-11	1.18	1.12	1.24	2.18E-11	1.1757	2.18E-11	1.1757	0.418	0
15	67185112	rs55894213	T	0.6471	1.062	0.8206	1.375	1.34E-11	1.18	1.13	1.24	2.12E-11	1.1758	2.12E-11	1.1758	0.4313	0
15	67186713	rs7173811	T	0.6812	0.9475	0.7326	1.226	7.16E-12	0.84	0.8	0.88	1.62E-11	0.8437	1.62E-11	0.8437	0.3681	0
15	67190030	rs11629568	T	0.6746	1.056	0.8197	1.36	3.37E-10	0.85	0.8	0.89	2.74E-09	0.8574	0.3736	0.9132	0.0996	63.13
15	67192763	rs11638476	A	0.7966	0.9672	0.7506	1.246	2.30E-10	1.18	1.12	1.24	9.60E-10	1.1707	0.2415	1.1123	0.1321	55.91
15	67194045	rs11556090	A	0.6499	0.9418	0.727	1.22	1.68E-10	0.85	0.81	0.9	4.77E-11	0.8529	4.77E-11	0.8529	0.4453	0
15	67195726	rs746978	T	0.6094	1.07	0.8263	1.385	4.97E-12	1.19	1.13	1.24	2.27E-12	1.1857	2.27E-12	1.1857	0.4276	0
15	67200559	rs7172645	T	0.581	0.9298	0.718	1.204	1.37E-11	0.84	0.8	0.88	1.60E-11	0.8431	1.60E-11	0.8431	0.4498	0
15	67202156	rs1050285	T	0.581	0.9298	0.718	1.204	1.08E-11	0.84	0.8	0.88	1.60E-11	0.8431	1.60E-11	0.8431	0.4498	0
15	67207073	rs7165503	T	0.581	1.076	0.8305	1.393	1.38E-10	1.17	1.12	1.23	2.15E-10	1.1667	2.15E-10	1.1667	0.5325	0
15	67207711	rs7171475	C	0.581	1.076	0.8305	1.393	1.92E-10	1.17	1.11	1.23	2.15E-10	1.1667	2.15E-10	1.1667	0.5325	0
15	67210255	rs62014612	A	0.6874	1.054	0.8146	1.365	6.78E-13	1.19	1.13	1.24	3.00E-13	1.1855	3.00E-13	1.1855	0.3641	0
15	67210431	rs11858541	A	0.6874	1.054	0.8146	1.365	1.10E-12	1.18	1.13	1.24	3.81E-12	1.1758	3.81E-12	1.1758	0.3984	0
15	67211299	rs10163156	A	0.8749	0.9798	0.7599	1.263	4.70E-13	1.19	1.14	1.25	4.58E-12	1.182	0.177	1.1258	0.1409	53.87
15	67211914	rs8024648	C	0.6874	0.9484	0.7327	1.228	7.98E-13	0.84	0.81	0.88	2.64E-13	0.8432	2.64E-13	0.8432	0.364	0
15	67214441	rs11855544	T	0.6874	1.054	0.8146	1.365	3.90E-13	1.19	1.13	1.24	3.00E-13	1.1855	3.00E-13	1.1855	0.3641	0
15	67216248	rs8027890	T	0.681	1.056	0.8158	1.366	2.89E-13	1.19	1.14	1.25	2.95E-13	1.1855	2.95E-13	1.1855	0.3709	0
15	67227149	rs8182047	T	0.5893	0.9313	0.7194	1.206	8.51E-14	0.84	0.8	0.88	2.77E-13	0.8427	2.77E-13	0.8427	0.4411	0
15	67228564	rs8032898	A	0.5893	1.074	0.8294	1.39	1.02E-13	1.19	1.14	1.25	2.48E-13	1.1862	2.48E-13	1.1862	0.4437	0

15	67232612	rs1822829	T	0.5988	0.9331	0.7209	1.208	3.12E-13	0.84	0.8	0.88	2.24E-13	0.8428	2.24E-13	0.8428	0.4322	0
15	67235107	rs55841220	A	0.5893	0.9313	0.7194	1.206	1.43E-13	0.84	0.8	0.88	2.20E-13	0.8427	2.20E-13	0.8427	0.441	0
15	67235630	rs12592388	A	0.5456	0.9235	0.7135	1.195	5.79E-14	0.84	0.8	0.88	2.02E-13	0.8425	2.02E-13	0.8425	0.4788	0
15	67237697	rs6494638	A	0.5988	1.072	0.828	1.387	1.25E-13	1.19	1.14	1.25	2.53E-13	1.1861	2.53E-13	1.1861	0.4352	0
15	67241206	rs1992216	T	0.6085	0.9348	0.7224	1.21	1.30E-13	0.84	0.8	0.88	2.29E-13	0.8428	2.29E-13	0.8428	0.4236	0
15	67242853	rs13313480	A	0.6085	0.9348	0.7224	1.21	1.39E-13	0.84	0.8	0.88	2.29E-13	0.8428	2.29E-13	0.8428	0.4236	0
15	67249211	rs7167097	T	0.489	0.9128	0.7049	1.182	1.91E-13	0.84	0.8	0.88	1.80E-13	0.8422	1.80E-13	0.8422	0.5351	0
15	67254106	rs2044330	A	0.5395	0.9223	0.7122	1.194	1.53E-13	0.83	0.79	0.87	6.52E-14	0.833	6.52E-14	0.833	0.4321	0
15	67258094	rs11631135	C	0.569	1.078	0.8332	1.394	1.11E-13	1.2	1.14	1.26	2.30E-13	1.1956	2.30E-13	1.1956	0.422	0
15	67264928	rs1444934	A	0.5413	0.9226	0.7125	1.195	1.33E-13	0.83	0.79	0.87	6.54E-14	0.833	6.54E-14	0.833	0.4306	0
15	67265335	rs8032868	T	0.5831	0.9301	0.7181	1.205	6.32E-14	0.83	0.79	0.87	7.13E-14	0.8332	7.13E-14	0.8332	0.3966	0
15	67268363	rs11071945	A	0.6243	1.066	0.8244	1.38	1.29E-13	1.2	1.14	1.26	2.59E-13	1.1951	2.59E-13	1.1951	0.3759	0
15	67272811	rs11855736	T	0.5988	1.072	0.8283	1.386	2.41E-13	1.2	1.14	1.26	2.44E-13	1.1954	2.44E-13	1.1954	0.3989	0
15	67279947	rs956197	T	0.5191	1.089	0.8405	1.411	2.52E-13	1.2	1.14	1.26	2.07E-13	1.196	2.07E-13	1.196	0.4702	0
15	67281237	rs55885982	C	0.489	0.9128	0.7049	1.182	8.91E-14	0.83	0.79	0.87	5.84E-14	0.8327	5.84E-14	0.8327	0.4786	0
15	67282350	rs13379644	C	0.489	0.9128	0.7049	1.182	5.50E-14	0.83	0.79	0.87	5.84E-14	0.8327	5.84E-14	0.8327	0.4786	0
15	67287743	rs12591763	C	0.4995	0.9149	0.7066	1.184	3.40E-14	0.83	0.79	0.87	5.98E-14	0.8328	5.98E-14	0.8328	0.4677	0
15	67288169	rs8038739	T	0.4995	0.9149	0.7066	1.184	3.29E-14	0.83	0.79	0.87	5.98E-14	0.8328	5.98E-14	0.8328	0.4677	0
15	67288566	rs8032525	A	0.5661	1.078	0.8334	1.395	5.08E-14	1.21	1.15	1.27	1.87E-14	1.2052	1.87E-14	1.2052	0.3877	0
15	67293123	rs1037039	T	0.5403	1.084	0.8374	1.403	4.36E-14	1.21	1.15	1.27	1.76E-14	1.2055	1.76E-14	1.2055	0.4119	0
15	67293349	rs8040514	T	0.5549	1.081	0.8351	1.399	5.15E-14	1.21	1.15	1.27	1.82E-14	1.2053	1.82E-14	1.2053	0.3999	0
15	67300880	rs11631885	A	0.489	0.9128	0.7049	1.182	1.20E-13	0.83	0.79	0.87	5.84E-14	0.8327	5.84E-14	0.8327	0.4786	0
15	67303877	rs8027755	A	0.489	1.096	0.846	1.419	7.11E-14	1.2	1.15	1.26	1.93E-13	1.1963	1.93E-13	1.1963	0.4994	0
15	67305681	rs8038883	A	0.489	1.096	0.846	1.419	5.84E-14	1.2	1.15	1.26	1.93E-13	1.1963	1.93E-13	1.1963	0.4994	0
15	67314982	rs4641686	T	0.5672	1.078	0.833	1.396	7.88E-14	1.2	1.15	1.26	2.30E-13	1.1956	2.30E-13	1.1956	0.4237	0
15	67319394	rs7170004	T	0.5661	0.9274	0.7167	1.2	8.60E-14	0.83	0.79	0.87	6.92E-14	0.8332	6.92E-14	0.8332	0.4067	0
15	67321840	rs11852260	A	0.5549	0.9253	0.715	1.197	1.15E-13	0.83	0.79	0.87	6.75E-14	0.8331	6.75E-14	0.8331	0.417	0
15	67328909	rs55832357	A	0.5549	1.081	0.8351	1.399	7.71E-13	1.19	1.13	1.24	2.33E-13	1.1864	2.33E-13	1.1864	0.4725	0
15	67331954	rs58036323	A	0.6105	1.069	0.8263	1.384	1.58E-12	1.18	1.13	1.24	4.10E-12	1.1763	4.10E-12	1.1763	0.4598	0
15	67335883	rs72745452	T	0.9592	1.007	0.781	1.297	4.47E-11	0.85	0.81	0.89	3.65E-10	0.8554	0.07393	0.8822	0.199	39.38
15	67336837	rs956129	A	0.9504	0.992	0.7697	1.278	7.58E-13	1.19	1.14	1.25	3.29E-12	1.1825	0.1087	1.1359	0.1673	47.55
15	67340589	rs6494650	A	0.5549	1.081	0.8351	1.399	4.83E-13	1.19	1.13	1.24	2.33E-13	1.1864	2.33E-13	1.1864	0.4725	0
15	67340904	rs4506839	A	0.599	0.9331	0.7209	1.208	8.57E-13	0.84	0.81	0.88	2.83E-13	0.8428	2.83E-13	0.8428	0.4319	0
15	67344417	rs11636980	A	0.5711	1.077	0.8324	1.394	1.36E-12	1.18	1.13	1.24	3.10E-12	1.1766	3.10E-12	1.1766	0.4946	0
15	67345146	rs2166185	T	0.8574	0.9767	0.7555	1.263	5.95E-11	1.18	1.13	1.24	3.28E-10	1.1718	0.1718	1.1214	0.1566	50.16
15	67346111	rs8037981	T	0.5711	1.077	0.8324	1.394	1.20E-12	1.18	1.13	1.24	3.10E-12	1.1766	3.10E-12	1.1766	0.4946	0

15	67346550	rs2120836	T	0.5549	1.081	0.8351	1.399	5.22E-13	1.19	1.13	1.24	2.33E-13	1.1864	2.33E-13	1.1864	0.4725	0
15	67346989	rs8043518	T	0.5549	1.081	0.8351	1.399	5.89E-13	1.19	1.13	1.24	2.33E-13	1.1864	2.33E-13	1.1864	0.4725	0
15	67347155	rs55962710	A	0.5549	0.9253	0.715	1.197	6.54E-13	0.84	0.8	0.88	2.06E-13	0.8426	2.06E-13	0.8426	0.4695	0
15	67349007	rs11071949	A	0.5711	0.9282	0.7171	1.201	3.06E-12	0.85	0.81	0.89	7.43E-12	0.8524	7.43E-12	0.8524	0.5104	0
15	67351685	rs4301969	T	0.5711	1.077	0.8324	1.394	3.93E-12	1.18	1.13	1.23	3.10E-12	1.1766	3.10E-12	1.1766	0.4946	0
15	67352975	rs11853367	A	0.5549	1.081	0.8351	1.399	2.66E-12	1.18	1.13	1.24	2.99E-12	1.1768	2.99E-12	1.1768	0.5123	0
15	67357640	rs869304	A	0.5711	0.9282	0.7171	1.201	6.37E-12	0.85	0.81	0.89	7.43E-12	0.8524	7.43E-12	0.8524	0.5104	0
15	67357757	rs869305	C	0.5549	0.9253	0.715	1.197	2.24E-12	0.85	0.81	0.89	7.22E-12	0.8523	7.22E-12	0.8523	0.5256	0
15	67361790	rs11071950	T	0.567	0.9274	0.7166	1.2	9.07E-12	0.85	0.81	0.89	7.37E-12	0.8523	7.37E-12	0.8523	0.5146	0
15	67362031	rs4496076	T	0.5811	1.075	0.8311	1.391	1.52E-12	1.19	1.13	1.25	2.66E-12	1.1858	2.66E-12	1.1858	0.4472	0
15	67362869	rs62016013	T	0.519	0.9185	0.7094	1.189	5.23E-12	0.84	0.8	0.88	2.09E-12	0.8426	2.09E-12	0.8426	0.5053	0
15	67367449	rs11637059	A	0.599	1.072	0.828	1.387	1.93E-13	1.2	1.14	1.26	2.44E-13	1.1954	2.44E-13	1.1954	0.3996	0
15	67367796	rs4625676	A	0.5747	0.9289	0.7179	1.202	3.76E-13	0.84	0.8	0.88	1.91E-12	0.8429	1.91E-12	0.8429	0.4521	0
15	67369287	rs7178870	A	0.5549	1.081	0.8351	1.399	1.41E-13	1.2	1.14	1.26	2.23E-13	1.1957	2.23E-13	1.1957	0.4355	0
15	67370762	rs41357746	A	0.599	0.9331	0.7209	1.208	1.75E-13	0.83	0.79	0.87	7.39E-14	0.8333	7.39E-14	0.8333	0.3819	0
15	67370868	rs41370046	T	0.567	0.9274	0.7166	1.2	3.64E-13	0.84	0.8	0.88	1.88E-12	0.8428	1.88E-12	0.8428	0.4598	0
15	67370912	rs72745473	A	0.9865	1.002	0.7775	1.292	5.98E-11	0.85	0.81	0.89	4.10E-10	0.8552	0.05046	0.8788	0.2126	35.64
15	67375719	rs11633767	A	0.5549	1.081	0.8351	1.399	2.01E-13	1.2	1.14	1.26	2.23E-13	1.1957	2.23E-13	1.1957	0.4355	0
15	67375867	rs11633857	A	0.5549	1.081	0.8351	1.399	9.72E-14	1.2	1.15	1.26	2.23E-13	1.1957	2.23E-13	1.1957	0.4355	0
15	67377074	rs4580100	A	0.5549	1.081	0.8351	1.399	6.46E-14	1.2	1.15	1.26	2.23E-13	1.1957	2.23E-13	1.1957	0.4355	0
15	67378002	rs55691227	C	0.5554	1.081	0.8349	1.399	1.02E-13	1.2	1.15	1.26	2.23E-13	1.1957	2.23E-13	1.1957	0.4358	0
15	67380164	rs11857142	A	0.6046	0.9341	0.7216	1.209	1.32E-13	0.83	0.79	0.87	7.47E-14	0.8334	7.47E-14	0.8334	0.378	0
15	67381230	rs12164949	A	0.5783	1.076	0.8316	1.391	9.28E-14	1.2	1.15	1.26	2.35E-13	1.1955	2.35E-13	1.1955	0.4143	0
15	67382373	rs66926990	C	0.9649	0.9943	0.7716	1.281	3.05E-12	0.84	0.8	0.88	3.87E-12	0.845	0.04707	0.8712	0.2005	38.98
15	67383209	rs4776924	A	0.4058	1.116	0.8611	1.447	6.34E-10	1.16	1.11	1.21	3.29E-10	1.1586	3.29E-10	1.1586	0.7739	0
15	67389569	rs6494654	T	0.06726	0.7818	0.6006	1.018	2.12E-09	0.86	0.82	0.9	4.30E-10	0.8572	4.30E-10	0.8572	0.4859	0
15	67402428	rs11071951	T	0.06611	1.28	0.9837	1.666	7.96E-09	1.15	1.1	1.2	1.62E-09	1.1538	1.62E-09	1.1538	0.4332	0
15	67402442	rs11071952	A	0.06611	0.7811	0.6001	1.017	7.05E-09	0.87	0.83	0.91	1.83E-09	0.8671	1.83E-09	0.8671	0.4302	0
15	67410569	rs7359257	A	0.1533	0.8252	0.634	1.074	2.43E-07	0.88	0.84	0.93	4.42E-08	0.8782	4.42E-08	0.8782	0.638	0
16	88249678	rs56124600	T	0.1195	0.8095	0.6205	1.056	2.92E-10	0.85	0.81	0.9	2.07E-11	0.8486	2.07E-11	0.8486	0.7234	0
16	88249687	rs28704855	C	0.1054	1.245	0.9549	1.624	5.57E-09	1.16	1.1	1.21	7.53E-10	1.1627	7.53E-10	1.1627	0.608	0
16	88251393	rs2370303	A	0.1945	0.8421	0.6496	1.092	8.84E-08	0.87	0.83	0.92	2.31E-08	0.869	2.31E-08	0.869	0.809	0
16	88252962	rs7203236	A	0.1965	0.842	0.6486	1.093	8.22E-08	0.87	0.83	0.92	1.11E-08	0.869	1.11E-08	0.869	0.8091	0
16	88258555	rs11860207	A	0.1864	0.8379	0.6446	1.089	3.94E-08	0.87	0.83	0.92	8.30E-09	0.8689	8.30E-09	0.8689	0.7823	0
16	88258747	rs7499083	C	0.0875	0.7961	0.6129	1.034	1.78E-07	0.87	0.83	0.92	1.66E-08	0.8672	1.66E-08	0.8672	0.5135	0
16	88264518	rs12447690	T	0.09765	1.264	0.9579	1.669	2.48E-16	1.23	1.17	1.3	1.20E-16	1.2311	1.20E-16	1.2311	0.8497	0

16	88265099	rs7201034	A	0.09938	0.7902	0.5972	1.046	1.06E-17	0.8	0.76	0.84	9.82E-19	0.7997	9.82E-19	0.7997	0.9323	0
16	88265885	rs7500824	A	0.1175	0.8006	0.606	1.058	1.89E-17	0.8	0.76	0.85	1.12E-18	0.8	1.12E-18	0.8	0.9959	0
16	88265987	rs35542380	C	0.09938	1.266	0.9564	1.675	4.32E-19	1.26	1.2	1.32	8.35E-20	1.2602	8.35E-20	1.2602	0.9739	0
16	88268370	rs6540214	A	0.09983	1.265	0.9561	1.674	1.58E-18	1.26	1.19	1.32	1.57E-19	1.2602	1.57E-19	1.2602	0.9782	0
16	88289717	rs58577366	T	0.03666	1.362	1.019	1.821	2.86E-15	1.25	1.18	1.32	2.06E-16	1.2537	2.06E-16	1.2537	0.5689	0
16	88289993	rs28425635	A	0.02599	1.386	1.04	1.848	2.28E-17	1.24	1.18	1.31	6.22E-18	1.2441	6.22E-18	1.2441	0.4548	0
16	88290223	rs58657775	A	0.05821	1.315	0.9905	1.747	1.94E-17	1.25	1.19	1.32	6.50E-18	1.2521	6.50E-18	1.2521	0.7306	0
16	88290639	rs28493272	T	0.03015	0.7273	0.5454	0.9699	3.28E-17	0.81	0.77	0.85	2.22E-17	0.8074	2.22E-17	0.8074	0.4699	0
16	88291954	rs28411862	C	0.02741	0.7237	0.543	0.9646	3.18E-16	0.81	0.77	0.85	2.10E-17	0.8073	2.10E-17	0.8073	0.449	0
16	88292560	rs28481824	T	0.06066	0.758	0.5675	1.012	5.65E-14	0.81	0.77	0.86	7.87E-15	0.8082	7.87E-15	0.8082	0.6589	0
16	88293176	rs34715091	A	0.0272	1.38	1.037	1.836	1.13E-16	1.23	1.17	1.3	4.16E-17	1.2342	4.16E-17	1.2342	0.4369	0
16	88293816	rs28445880	T	0.05902	0.7586	0.5694	1.011	2.76E-14	0.81	0.77	0.86	3.29E-15	0.8082	3.29E-15	0.8082	0.6599	0
16	88293963	rs28563118	C	0.05672	1.319	0.9921	1.753	2.07E-15	1.23	1.17	1.3	7.88E-16	1.2328	7.88E-16	1.2328	0.636	0
16	88297909	rs9934580	A	0.05386	0.7569	0.5702	1.005	3.42E-15	0.82	0.78	0.86	7.71E-16	0.818	7.71E-16	0.818	0.5852	0
16	88298034	rs9938149	A	0.1136	1.255	0.9472	1.664	1.51E-15	1.22	1.16	1.29	1.10E-15	1.221	1.10E-15	1.221	0.8463	0
16	88299690	rs11259972	T	0.1167	0.7985	0.6028	1.058	9.19E-16	0.81	0.77	0.86	5.34E-17	0.8096	5.34E-17	0.8096	0.9218	0
16	88309991	rs9938961	A	0.1257	0.7694	0.55	1.076	2.00E-11	0.82	0.78	0.87	2.96E-12	0.8185	2.96E-12	0.8185	0.7138	0
17	4856736	rs4609912	C	0.3022	1.277	0.8027	2.031	5.42E-08	1.24	1.15	1.35	4.72E-08	1.241	4.72E-08	1.241	0.9026	0
17	4921330	rs12602901	C	0.2923	0.8326	0.5921	1.171	5.46E-11	0.82	0.77	0.87	2.84E-11	0.8204	2.84E-11	0.8204	0.9312	0
17	4921795	rs35414445	T	0.3059	1.195	0.8499	1.68	7.35E-11	1.22	1.15	1.3	9.51E-11	1.2192	9.51E-11	1.2192	0.9066	0
17	4921914	rs35659308	A	0.2695	0.8251	0.5865	1.161	6.59E-11	0.82	0.78	0.87	1.99E-11	0.8201	1.99E-11	0.8201	0.972	0
17	4922066	rs2586527	A	0.1381	1.281	0.9235	1.776	8.22E-11	1.21	1.14	1.28	3.56E-11	1.2121	3.56E-11	1.2121	0.7364	0
17	4922092	rs28846454	A	0.3006	0.8611	0.6487	1.143	1.32E-08	0.86	0.82	0.91	2.91E-09	0.86	2.91E-09	0.86	0.9931	0
17	4926265	rs34629349	A	0.2631	1.176	0.8854	1.561	9.50E-09	1.16	1.1	1.22	4.63E-09	1.1605	4.63E-09	1.1605	0.9257	0
17	4928495	rs9914087	A	0.2803	0.8555	0.6445	1.136	1.39E-08	0.86	0.82	0.91	2.77E-09	0.8599	2.77E-09	0.8599	0.9715	0
17	4928908	rs11078540	T	0.4124	1.151	0.8222	1.611	1.68E-10	1.22	1.15	1.3	1.39E-10	1.2177	1.39E-10	1.2177	0.7385	0
17	4929896	rs12952420	T	0.4124	0.8688	0.6206	1.216	1.17E-10	0.82	0.77	0.87	1.51E-10	0.8215	1.51E-10	0.8215	0.7403	0
17	4930183	rs9903826	A	0.2785	1.17	0.881	1.553	1.24E-08	1.16	1.1	1.22	4.80E-09	1.1603	4.80E-09	1.1603	0.9534	0
17	4930914	rs2243086	T	0.4293	0.8733	0.624	1.222	5.94E-11	0.82	0.77	0.87	1.56E-10	0.8217	1.56E-10	0.8217	0.7179	0
17	4931124	rs34377693	C	0.4703	0.8866	0.6395	1.229	3.12E-10	0.82	0.78	0.87	1.15E-10	0.8221	1.15E-10	0.8221	0.6451	0
17	4931922	rs2243090	T	0.1973	0.8294	0.6241	1.102	9.16E-09	0.86	0.82	0.91	1.93E-09	0.8591	1.93E-09	0.8591	0.8058	0
17	4935103	rs2243096	A	0.3699	0.8567	0.6109	1.201	1.23E-10	0.82	0.77	0.87	9.18E-11	0.8211	9.18E-11	0.8211	0.8027	0
17	4936635	rs400688	A	0.3467	0.85	0.6059	1.192	4.46E-11	0.82	0.77	0.87	7.60E-11	0.8209	7.60E-11	0.8209	0.8377	0
17	4941586	rs238248	T	0.3197	0.8413	0.5987	1.182	7.30E-11	0.82	0.77	0.87	7.12E-11	0.8206	7.12E-11	0.8206	0.8844	0
17	4944148	rs238245	T	0.3133	0.8398	0.5981	1.179	2.64E-11	0.82	0.77	0.87	1.42E-11	0.8206	1.42E-11	0.8206	0.892	0
17	4945155	rs6559	A	0.2187	0.8056	0.5708	1.137	2.64E-11	0.81	0.77	0.87	3.10E-12	0.8099	3.10E-12	0.8099	0.9757	0

17	4946340	rs238243	A	0.1937	0.7947	0.5619	1.124	1.12E-11	0.81	0.76	0.86	5.26E-12	0.8095	5.26E-12	0.8095	0.9154	0
17	4949168	rs4790714	T	0.1161	1.253	0.9458	1.659	3.84E-09	1.16	1.1	1.22	9.42E-10	1.1626	9.42E-10	1.1626	0.596	0
17	4953081	rs238238	A	0.07498	0.7582	0.5591	1.028	6.66E-09	0.86	0.81	0.9	3.02E-09	0.857	3.02E-09	0.857	0.4241	0
17	4958477	rs2871188	T	0.1423	0.7726	0.5474	1.09	8.19E-13	0.81	0.77	0.86	1.06E-13	0.809	1.06E-13	0.809	0.7907	0
17	4958909	rs2005724	T	0.1588	0.7797	0.5515	1.102	1.31E-12	0.81	0.76	0.86	8.02E-13	0.8091	8.02E-13	0.8091	0.8315	0
17	4960115	rs238224	A	0.4086	0.8552	0.5901	1.239	2.18E-12	0.8	0.75	0.85	7.58E-13	0.8014	7.58E-13	0.8014	0.728	0
17	4963004	rs35688913	T	0.1626	0.7716	0.5363	1.11	1.99E-12	0.8	0.75	0.85	1.17E-12	0.7992	1.17E-12	0.7992	0.8478	0
17	4966068	rs34235023	C	0.168	0.7743	0.5382	1.114	5.83E-13	0.79	0.74	0.84	2.16E-13	0.7895	2.16E-13	0.7895	0.9152	0
17	4970920	rs452825	T	0.1561	0.7687	0.5345	1.106	5.02E-13	0.8	0.75	0.85	7.09E-13	0.7991	7.09E-13	0.7991	0.832	0
17	4973825	rs238229	A	0.1618	1.289	0.9032	1.84	6.58E-14	1.25	1.18	1.33	4.62E-14	1.251	4.62E-14	1.251	0.8674	0
17	4975665	rs238228	A	0.1618	1.289	0.9032	1.84	4.55E-14	1.26	1.18	1.33	6.02E-15	1.2608	6.02E-15	1.2608	0.9016	0
17	4979657	rs1859434	A	0.3218	1.191	0.843	1.682	1.19E-13	1.25	1.18	1.33	6.43E-14	1.2483	6.43E-14	1.2483	0.7869	0
17	4980523	rs238234	C	0.1618	0.7758	0.5436	1.107	1.02E-13	0.8	0.75	0.85	4.62E-14	0.7993	4.62E-14	0.7993	0.8674	0
17	4982742	rs238232	A	0.1618	0.7758	0.5436	1.107	1.16E-13	0.8	0.75	0.85	3.83E-14	0.7993	3.83E-14	0.7993	0.8674	0
17	4985135	rs4790721	A	0.1531	0.7715	0.5405	1.101	9.66E-14	0.8	0.75	0.85	3.69E-14	0.7992	3.69E-14	0.7992	0.8437	0
17	4987548	rs8071428	T	0.1613	0.7962	0.5788	1.095	1.62E-09	0.85	0.8	0.89	1.76E-09	0.8484	1.76E-09	0.8484	0.692	0
17	4990062	rs427703	C	0.2093	1.211	0.8982	1.633	1.16E-08	1.17	1.11	1.23	2.14E-09	1.1712	2.14E-09	1.1712	0.824	0
17	4990830	rs113674559	T	0.1695	0.7993	0.5807	1.1	1.77E-09	0.85	0.8	0.89	2.05E-09	0.8485	2.05E-09	0.8485	0.7099	0
17	4993544	rs238227	T	0.1695	0.7993	0.5807	1.1	1.51E-09	0.85	0.8	0.89	2.05E-09	0.8485	2.05E-09	0.8485	0.7099	0
17	4995238	rs471064	A	0.2309	1.237	0.8737	1.75	4.27E-14	1.26	1.18	1.33	1.16E-14	1.2593	1.16E-14	1.2593	0.9184	0
17	4997334	rs1004379	A	0.06225	0.7056	0.4891	1.018	9.92E-13	0.8	0.76	0.85	9.22E-14	0.7974	9.22E-14	0.7974	0.5076	0
17	4997482	rs886582	T	0.07305	0.7156	0.4964	1.032	1.08E-12	0.8	0.75	0.85	3.26E-13	0.7975	3.26E-13	0.7975	0.556	0
17	4999663	rs238237	A	0.05708	1.397	0.99	1.973	5.43E-13	1.25	1.18	1.33	1.40E-13	1.2542	1.40E-13	1.2542	0.5337	0
17	5021038	rs238235	A	0.06062	0.699	0.4809	1.016	7.07E-14	0.79	0.75	0.84	5.88E-15	0.7875	5.88E-15	0.7875	0.5267	0
17	5022831	rs35748479	T	0.07962	0.7182	0.496	1.04	1.24E-13	0.79	0.75	0.84	6.83E-15	0.788	6.83E-15	0.788	0.6185	0
17	5027076	rs12603055	C	0.06636	0.7047	0.485	1.024	2.23E-14	0.79	0.74	0.84	3.48E-15	0.7877	3.48E-15	0.7877	0.554	0
17	5032312	rs34556430	A	0.09226	0.7273	0.502	1.054	1.06E-13	0.79	0.75	0.84	7.47E-15	0.7883	7.47E-15	0.7883	0.6661	0
17	5033603	rs2304445	T	0.1007	0.7331	0.506	1.062	2.71E-13	0.8	0.75	0.85	1.03E-13	0.7982	1.03E-13	0.7982	0.6485	0
17	5033677	rs346821	A	0.1411	1.237	0.9319	1.642	1.95E-09	1.17	1.11	1.23	8.42E-10	1.1721	8.42E-10	1.1721	0.7046	0
17	5034687	rs346824	T	0.1939	0.8284	0.6236	1.1	3.37E-10	0.85	0.8	0.89	4.92E-10	0.8493	4.92E-10	0.8493	0.8613	0
17	5035789	rs9905696	A	0.1939	0.8284	0.6236	1.1	3.55E-10	0.85	0.8	0.89	4.92E-10	0.8493	4.92E-10	0.8493	0.8613	0
17	5037805	rs7208035	A	0.04013	0.7213	0.528	0.9854	1.44E-10	0.84	0.8	0.89	8.65E-12	0.8366	8.65E-12	0.8366	0.3449	0
17	5038404	rs4239053	A	0.1612	1.226	0.9219	1.63	6.56E-10	1.18	1.12	1.24	2.88E-10	1.1815	2.88E-10	1.1815	0.7959	0
17	5040070	rs9909219	T	0.1667	1.223	0.9196	1.625	5.72E-10	1.18	1.12	1.25	2.93E-10	1.1814	2.93E-10	1.1814	0.8086	0
17	5040957	rs380229	A	0.1366	0.8049	0.6048	1.071	6.17E-10	0.85	0.8	0.89	6.03E-10	0.8485	6.03E-10	0.8485	0.7131	0
17	5042515	rs9898431	A	0.08666	1.284	0.9647	1.708	5.32E-09	1.17	1.11	1.23	6.43E-10	1.1734	6.43E-10	1.1734	0.53	0

17	5043030	rs398635	A	0.1344	1.244	0.9347	1.656	9.85E-10	1.18	1.12	1.24	2.57E-10	1.1821	2.57E-10	1.1821	0.7217	0
17	5045293	rs7207533	T	0.1223	1.253	0.9412	1.669	1.53E-09	1.18	1.12	1.24	2.44E-10	1.1823	2.44E-10	1.1823	0.6862	0
17	5045420	rs7208332	A	0.1374	0.8053	0.6052	1.072	1.42E-09	0.85	0.81	0.9	5.27E-10	0.8485	5.27E-10	0.8485	0.7156	0
17	5046379	rs55784950	T	0.08255	1.288	0.9679	1.714	5.43E-09	1.17	1.11	1.23	7.22E-10	1.1736	7.22E-10	1.1736	0.5167	0
17	5047007	rs62072530	A	0.1286	0.8011	0.6017	1.066	1.83E-09	0.85	0.81	0.9	5.07E-10	0.8483	5.07E-10	0.8483	0.6898	0
17	5047299	rs8070702	T	0.1286	0.8011	0.6017	1.066	1.30E-09	0.85	0.81	0.9	5.07E-10	0.8483	5.07E-10	0.8483	0.6898	0
17	5047338	rs4790730	A	0.1286	0.8011	0.6017	1.066	1.09E-09	0.85	0.8	0.89	5.07E-10	0.8483	5.07E-10	0.8483	0.6898	0
17	5047803	rs4790731	A	0.1286	1.248	0.9377	1.662	9.29E-10	1.18	1.12	1.24	2.90E-10	1.1822	2.90E-10	1.1822	0.7059	0
17	5048368	rs34548580	T	0.07824	1.406	0.9623	2.053	2.23E-13	1.26	1.19	1.34	7.46E-14	1.2636	7.46E-14	1.2636	0.5759	0
17	5048889	rs7218028	A	0.1719	1.217	0.918	1.614	8.50E-10	1.18	1.12	1.24	3.46E-10	1.1812	3.46E-10	1.1812	0.8331	0
17	5051537	rs8067699	A	0.6872	1.077	0.7501	1.547	5.63E-12	1.25	1.18	1.34	1.73E-11	1.2443	1.73E-11	1.2443	0.4272	0
17	5052550	rs12939323	A	0.8827	0.9734	0.6803	1.393	9.34E-11	0.81	0.76	0.86	2.46E-10	0.8147	2.46E-10	0.8147	0.3225	0
17	5054612	rs12945017	A	0.8278	1.04	0.7316	1.478	1.35E-09	1.21	1.14	1.29	2.53E-09	1.2045	2.53E-09	1.2045	0.4059	0
17	5055219	rs12943738	A	0.9736	0.9941	0.7017	1.409	9.40E-10	0.82	0.77	0.87	2.90E-09	0.8252	0.0008883	0.834	0.287	11.8
17	5055348	rs12951036	A	0.9885	1.003	0.7052	1.425	7.01E-10	0.82	0.77	0.87	8.15E-10	0.825	0.006505	0.8393	0.2691	18.11
17	5056295	rs71368530	A	0.9403	0.9866	0.6942	1.402	7.31E-10	0.82	0.77	0.88	7.37E-10	0.8246	5.92E-07	0.8268	0.31	2.97
17	5058048	rs12948476	A	0.9642	1.008	0.7048	1.442	2.04E-10	0.81	0.76	0.87	2.69E-10	0.8155	0.02976	0.8393	0.2387	27.96
17	5070267	rs12150058	A	0.9549	1.011	0.7008	1.457	5.10E-09	0.82	0.77	0.88	4.23E-09	0.8253	0.008922	0.8399	0.2697	17.9
17	5070701	rs61583868	T	0.9662	0.9923	0.6934	1.42	5.08E-08	1.19	1.12	1.26	4.96E-08	1.1838	4.96E-08	1.1838	0.3273	0
17	5077086	rs12601694	A	0.919	1.019	0.7069	1.469	3.33E-08	0.83	0.78	0.89	3.29E-08	0.8352	0.006709	0.847	0.2793	14.58
17	5077384	rs35012137	A	0.9654	1.008	0.6989	1.454	4.67E-08	0.83	0.78	0.89	2.81E-08	0.8349	4.23E-05	0.8384	0.306	4.59
17	5085167	rs56916247	A	0.947	1.013	0.6948	1.477	1.67E-08	0.83	0.78	0.89	3.41E-08	0.8348	3.41E-05	0.838	0.3072	4.07
17	5088878	rs77455068	A	0.9774	0.9946	0.6831	1.448	3.46E-08	1.2	1.12	1.28	3.27E-08	1.1937	3.27E-08	1.1937	0.3343	0
17	5089341	rs36046323	T	0.9402	0.9857	0.6761	1.437	4.99E-09	1.21	1.14	1.3	1.65E-08	1.2028	0.001011	1.192	0.2934	9.4
17	5090941	rs34424607	T	0.947	0.9873	0.6772	1.439	1.23E-09	1.22	1.15	1.31	4.29E-09	1.2125	0.00502	1.1946	0.2782	14.96
17	5094138	rs12600437	A	0.9842	1.004	0.6894	1.462	1.60E-08	0.83	0.78	0.89	1.18E-08	0.8343	1.18E-08	0.8343	0.3275	0
17	19716685	rs12943590	A	0.6062	0.926	0.6912	1.24	3.09E-08	0.86	0.82	0.91	1.10E-08	0.8619	1.10E-08	0.8619	0.6255	0
17	19717311	rs758426	A	0.6062	1.08	0.8061	1.447	2.90E-08	1.16	1.1	1.22	2.09E-08	1.1575	2.09E-08	1.1575	0.6372	0
17	19736402	rs1006556	C	0.8008	0.9588	0.6918	1.329	2.11E-08	0.85	0.81	0.9	1.04E-08	0.8529	1.04E-08	0.8529	0.476	0
17	19746728	rs12939864	T	0.9728	1.006	0.7253	1.394	2.11E-09	0.84	0.79	0.89	2.88E-09	0.8444	0.001633	0.8531	0.2865	11.98
17	19748169	rs2072327	T	0.8996	1.02	0.7476	1.392	4.05E-10	1.19	1.13	1.26	6.21E-10	1.1846	6.21E-10	1.1846	0.3387	0
17	19748589	rs4646785	T	0.8878	1.023	0.7443	1.407	9.01E-12	0.82	0.78	0.87	1.38E-11	0.8255	0.1227	0.8645	0.1799	44.4
17	19749997	rs12949049	A	0.8815	1.024	0.7508	1.396	8.06E-10	1.19	1.12	1.25	6.06E-10	1.1847	6.06E-10	1.1847	0.3499	0
17	31554201	rs8080041	T	0.1206	1.347	0.9248	1.962	2.69E-07	1.18	1.11	1.25	4.96E-08	1.1841	4.96E-08	1.1841	0.4961	0
17	31556171	rs8072121	T	0.07879	1.448	0.9583	2.188	2.14E-08	1.21	1.13	1.29	5.51E-09	1.2155	5.51E-09	1.2155	0.3999	0
17	31556453	rs8072466	T	0.05999	1.504	0.983	2.301	5.87E-10	1.23	1.15	1.31	1.00E-10	1.2356	1.00E-10	1.2356	0.3593	0

17	31558198	rs56161228	A	0.04478	0.6387	0.4123	0.9896	2.70E-10	0.81	0.76	0.86	5.58E-11	0.8058	0.0001462	0.7971	0.2928	9.63
17	31561073	rs57308089	T	0.03923	1.588	1.023	2.466	4.98E-10	1.23	1.15	1.31	1.24E-10	1.2368	0.004461	1.2692	0.2602	21.13
17	31561534	rs56057954	A	0.03736	1.596	1.028	2.478	4.34E-10	1.23	1.15	1.32	1.37E-10	1.237	0.006721	1.2746	0.2511	24.07
17	31562044	rs56908712	T	0.03736	1.596	1.028	2.478	3.30E-10	1.23	1.16	1.32	1.37E-10	1.237	0.006721	1.2746	0.2511	24.07
17	31562933	rs7216082	A	0.04721	1.552	1.005	2.395	5.32E-10	1.23	1.15	1.31	1.17E-10	1.2363	4.38E-05	1.2464	0.299	7.29
17	31563851	rs68052392	C	0.05908	1.5	0.9846	2.284	7.87E-08	1.2	1.12	1.28	2.45E-08	1.2066	5.85E-05	1.2131	0.3047	5.09
17	48251305	rs8081754	A	0.1151	1.29	0.9399	1.769	3.36E-08	1.18	1.11	1.25	1.00E-08	1.1835	1.00E-08	1.1835	0.5871	0
17	48253072	rs7222593	T	0.1267	0.7812	0.5691	1.073	3.99E-08	0.86	0.81	0.91	2.08E-08	0.8576	2.08E-08	0.8576	0.5581	0
17	48258937	rs7211607	T	0.1268	1.28	0.9324	1.757	6.90E-08	1.16	1.1	1.23	3.38E-08	1.1633	3.38E-08	1.1633	0.5485	0
17	48259402	rs12940931	T	0.1156	0.7757	0.5653	1.064	8.03E-09	0.85	0.8	0.9	3.45E-09	0.8477	3.45E-09	0.8477	0.5767	0
17	48259674	rs12941754	T	0.1267	0.7812	0.5691	1.073	6.41E-08	0.86	0.81	0.91	2.08E-08	0.8576	2.08E-08	0.8576	0.5581	0
17	48260211	rs8065115	T	0.1616	1.256	0.9129	1.727	6.23E-08	1.16	1.1	1.23	3.83E-08	1.1626	3.83E-08	1.1626	0.63	0
17	48263229	rs6504145	T	0.148	1.265	0.92	1.739	1.99E-08	1.17	1.11	1.24	1.10E-08	1.1727	1.10E-08	1.1727	0.6358	0
17	48263679	rs17694192	T	0.1372	0.7856	0.5715	1.08	1.03E-08	0.85	0.8	0.9	4.27E-09	0.848	4.27E-09	0.848	0.6328	0
17	48264913	rs17621199	A	0.148	1.265	0.92	1.739	1.79E-08	1.17	1.11	1.24	1.10E-08	1.1727	1.10E-08	1.1727	0.6358	0
17	48268623	rs12943082	T	0.1156	0.7757	0.5653	1.064	9.18E-09	0.85	0.8	0.9	3.45E-09	0.8477	3.45E-09	0.8477	0.5767	0
17	48268694	rs12950325	A	0.1156	1.289	0.9395	1.769	1.43E-08	1.18	1.11	1.24	2.07E-09	1.1832	2.07E-09	1.1832	0.5898	0
17	48269125	rs17621297	A	0.1156	0.7757	0.5653	1.064	1.73E-08	0.85	0.81	0.9	3.06E-09	0.8477	3.06E-09	0.8477	0.5767	0
17	48270566	rs12938032	T	0.1558	1.259	0.9159	1.732	1.71E-08	1.17	1.11	1.24	1.14E-08	1.1725	1.14E-08	1.1725	0.6567	0
17	48270633	rs62067799	A	0.1446	1.267	0.9218	1.743	7.79E-09	1.18	1.11	1.25	2.65E-09	1.1825	2.65E-09	1.1825	0.6664	0
17	48277311	rs12948128	A	0.1204	0.7781	0.567	1.068	1.28E-08	0.85	0.81	0.9	3.13E-09	0.8478	3.13E-09	0.8478	0.5899	0
17	48281916	rs17621707	T	0.1204	0.7781	0.567	1.068	1.47E-08	0.85	0.8	0.9	3.97E-09	0.8477	3.97E-09	0.8477	0.5899	0
17	48284393	rs17694895	C	0.1204	1.285	0.9364	1.764	1.42E-08	1.17	1.11	1.24	9.89E-09	1.1733	9.89E-09	1.1733	0.5674	0
17	48286425	rs8076655	T	0.1204	0.7781	0.567	1.068	1.45E-08	0.85	0.81	0.9	3.13E-09	0.8478	3.13E-09	0.8478	0.5899	0
17	48289363	rs8069900	A	0.1204	0.7781	0.567	1.068	1.24E-08	0.85	0.81	0.9	3.13E-09	0.8478	3.13E-09	0.8478	0.5899	0
17	48297849	rs4128439	A	0.1204	0.7781	0.567	1.068	1.25E-08	0.85	0.81	0.9	3.13E-09	0.8478	3.13E-09	0.8478	0.5899	0
17	48302682	rs7215143	T	0.1204	0.7781	0.567	1.068	1.22E-08	0.85	0.81	0.9	3.13E-09	0.8478	3.13E-09	0.8478	0.5899	0
17	48319469	rs7206951	A	0.1885	0.8085	0.5889	1.11	1.49E-08	0.85	0.81	0.9	3.16E-09	0.8488	3.16E-09	0.8488	0.7604	0
17	48338660	rs17623154	A	0.1885	1.237	0.901	1.698	8.11E-08	1.17	1.1	1.24	2.17E-08	1.172	2.17E-08	1.172	0.7346	0
17	48339953	rs12940162	T	0.1885	1.237	0.901	1.698	2.00E-08	1.17	1.11	1.24	1.00E-08	1.1719	1.00E-08	1.1719	0.7344	0
17	48340785	rs35509644	A	0.1885	1.237	0.901	1.698	1.40E-08	1.17	1.11	1.24	1.00E-08	1.1719	1.00E-08	1.1719	0.7344	0
17	48341227	rs7218848	A	0.1757	0.8034	0.5853	1.103	8.70E-09	0.85	0.8	0.9	4.34E-09	0.8486	4.34E-09	0.8486	0.7311	0
17	48348972	rs12941877	A	0.1885	1.237	0.901	1.698	1.42E-08	1.17	1.11	1.24	1.00E-08	1.1719	1.00E-08	1.1719	0.7344	0
17	48350623	rs8078999	C	0.1885	1.237	0.901	1.698	1.22E-08	1.17	1.11	1.24	1.00E-08	1.1719	1.00E-08	1.1719	0.7344	0
17	48351206	rs7224686	T	0.1885	1.237	0.901	1.698	1.12E-08	1.17	1.11	1.24	1.00E-08	1.1719	1.00E-08	1.1719	0.7344	0
17	48352260	rs8072521	T	0.1885	0.8085	0.5889	1.11	6.56E-09	0.85	0.8	0.9	3.56E-09	0.8487	3.56E-09	0.8487	0.7604	0

17	48354476	rs17696503	A	0.1885	0.8085	0.5889	1.11	1.15E-08	0.85	0.81	0.9	3.16E-09	0.8488	3.16E-09	0.8488	0.7604	0
17	48354811	rs17623482	T	0.1885	0.8085	0.5889	1.11	1.15E-08	0.85	0.81	0.9	3.16E-09	0.8488	3.16E-09	0.8488	0.7604	0
17	48355625	rs35759756	A	0.1885	1.237	0.901	1.698	1.15E-08	1.17	1.11	1.24	1.00E-08	1.1719	1.00E-08	1.1719	0.7344	0
17	48356052	rs34466582	T	0.1757	1.245	0.9067	1.708	8.05E-09	1.18	1.11	1.25	2.31E-09	1.1819	2.31E-09	1.1819	0.7438	0
17	48356245	rs35003027	A	0.1885	1.237	0.901	1.698	1.23E-08	1.17	1.11	1.24	1.00E-08	1.1719	1.00E-08	1.1719	0.7344	0
17	48365896	rs12150117	A	0.1885	1.237	0.901	1.698	1.22E-08	1.17	1.11	1.24	1.13E-08	1.1719	1.13E-08	1.1719	0.7344	0
17	48367058	rs17696640	A	0.1885	0.8085	0.5889	1.11	1.46E-08	0.85	0.81	0.9	3.56E-09	0.8487	3.56E-09	0.8487	0.7604	0
17	48381289	rs12939680	T	0.1885	0.8085	0.5889	1.11	1.42E-08	0.85	0.81	0.9	3.56E-09	0.8487	3.56E-09	0.8487	0.7604	0
17	48395087	rs56146024	A	0.1885	0.8085	0.5889	1.11	1.01E-08	0.85	0.81	0.9	3.56E-09	0.8487	3.56E-09	0.8487	0.7604	0
17	48400833	rs12952824	A	0.1757	1.245	0.9067	1.708	8.81E-09	1.18	1.11	1.24	2.31E-09	1.1819	2.31E-09	1.1819	0.7438	0
17	48404045	rs12948086	T	0.1885	1.237	0.901	1.698	5.33E-09	1.18	1.12	1.25	1.88E-09	1.1816	1.88E-09	1.1816	0.7738	0
17	48410238	rs35533148	A	0.1783	1.243	0.9056	1.706	8.54E-09	1.18	1.11	1.24	1.82E-09	1.1818	1.82E-09	1.1818	0.751	0
17	48418050	rs1396870	T	0.2127	0.8167	0.5939	1.123	4.59E-08	0.86	0.81	0.91	4.65E-08	0.8587	4.65E-08	0.8587	0.7541	0
17	48419579	rs12945780	A	0.2127	1.224	0.8904	1.684	4.23E-08	1.17	1.11	1.24	1.52E-08	1.1716	1.52E-08	1.1716	0.7845	0
17	48422738	rs7220387	A	0.2127	1.224	0.8904	1.684	4.85E-08	1.17	1.1	1.23	1.36E-08	1.1716	1.36E-08	1.1716	0.7844	0
17	48424348	rs35713035	T	0.2127	1.224	0.8904	1.684	4.46E-08	1.17	1.1	1.23	1.36E-08	1.1716	1.36E-08	1.1716	0.7844	0
17	48425526	rs7218217	T	0.2127	1.224	0.8904	1.684	5.68E-08	1.17	1.1	1.23	1.36E-08	1.1716	1.36E-08	1.1716	0.7844	0
17	48429317	rs8073466	T	0.1984	1.236	0.8949	1.708	3.32E-08	1.17	1.11	1.24	1.63E-08	1.1719	1.63E-08	1.1719	0.743	0
17	48435813	rs55682156	T	0.2127	1.224	0.8904	1.684	4.72E-08	1.17	1.1	1.23	1.52E-08	1.1716	1.52E-08	1.1716	0.7845	0
17	48441743	rs1533057	A	0.2127	1.224	0.8904	1.684	6.87E-08	1.17	1.1	1.23	1.36E-08	1.1716	1.36E-08	1.1716	0.7844	0
17	48448660	rs6504257	A	0.2237	0.8211	0.5978	1.128	8.04E-08	0.86	0.81	0.91	4.78E-08	0.8588	4.78E-08	0.8588	0.7784	0
17	48451013	rs35767434	T	0.2237	0.8211	0.5978	1.128	9.80E-08	0.86	0.81	0.91	4.78E-08	0.8588	4.78E-08	0.8588	0.7784	0
17	48451167	rs34771639	A	0.2237	0.8211	0.5978	1.128	8.22E-08	0.86	0.81	0.91	4.78E-08	0.8588	4.78E-08	0.8588	0.7784	0
17	48455544	rs4793846	T	0.1207	1.28	0.9371	1.75	6.73E-08	1.16	1.1	1.23	3.64E-08	1.1634	3.64E-08	1.1634	0.5427	0
17	48456524	rs8072770	T	0.2258	1.217	0.8858	1.671	6.86E-08	1.17	1.1	1.23	1.56E-08	1.1714	1.56E-08	1.1714	0.8107	0
17	48474867	rs12945803	T	0.2027	0.815	0.5949	1.117	5.30E-08	0.85	0.8	0.9	2.21E-08	0.8488	2.21E-08	0.8488	0.7969	0
17	48488124	rs7222242	A	0.03843	1.399	1.018	1.922	1.96E-08	1.18	1.12	1.25	5.90E-09	1.1866	1.82E-05	1.1925	0.3017	6.25
17	48489528	rs17703636	T	0.03843	0.715	0.5204	0.9823	1.56E-08	0.84	0.8	0.9	8.87E-10	0.8356	8.87E-10	0.8356	0.3283	0
17	48489772	rs17703660	T	0.03843	0.715	0.5204	0.9823	1.62E-08	0.84	0.8	0.9	8.87E-10	0.8356	8.87E-10	0.8356	0.3283	0
17	48490489	rs8064626	A	0.03843	1.399	1.018	1.922	1.70E-08	1.18	1.12	1.25	5.31E-09	1.1866	1.80E-05	1.1925	0.3016	6.27
17	48491854	rs8072250	A	0.02222	1.442	1.054	1.974	2.14E-07	1.17	1.1	1.23	1.62E-08	1.1781	0.01881	1.2242	0.1991	39.34
17	48506158	rs8066613	A	0.0217	1.442	1.055	1.972	1.59E-08	1.18	1.12	1.25	4.07E-09	1.188	0.009984	1.227	0.2165	34.52
17	48511903	rs34378400	A	0.0217	0.6933	0.5071	0.9479	1.49E-08	0.85	0.8	0.9	6.63E-09	0.8442	0.01296	0.8153	0.2091	36.61
17	48515496	rs62065846	A	0.02303	1.437	1.051	1.965	8.23E-09	1.18	1.12	1.25	2.43E-09	1.1876	0.008155	1.2233	0.2246	32.18
17	48517062	rs9989493	T	0.02402	1.432	1.048	1.957	7.87E-09	1.18	1.12	1.25	2.46E-09	1.1875	0.00658	1.2202	0.2318	30.06
17	48518906	rs7225995	C	0.02264	1.437	1.052	1.964	6.63E-09	1.19	1.12	1.26	8.34E-10	1.1977	0.002976	1.2256	0.2443	26.24

17	48528860	rs35621842	T	0.02795	1.423	1.039	1.949	6.45E-08	1.18	1.11	1.25	1.04E-08	1.1878	0.003538	1.2127	0.2518	23.86
17	48530596	rs12939811	A	0.02795	1.423	1.039	1.949	2.80E-08	1.19	1.12	1.26	2.03E-09	1.1975	0.000578	1.214	0.2738	16.51
17	48531730	rs62066689	T	0.02688	0.7011	0.5119	0.9602	1.71E-08	0.84	0.79	0.89	1.65E-09	0.8347	0.0008449	0.822	0.2686	18.31
17	48532666	rs8079617	T	0.02688	0.7011	0.5119	0.9602	3.56E-08	0.84	0.8	0.9	1.84E-09	0.8347	0.0008475	0.822	0.2686	18.29
17	48532698	rs8074125	T	0.02688	1.426	1.041	1.954	1.54E-08	1.19	1.12	1.26	1.79E-09	1.1975	0.0008944	1.2163	0.2681	18.46
17	48535216	rs56038617	T	0.02688	1.426	1.041	1.954	5.51E-08	1.18	1.11	1.25	1.02E-08	1.1879	0.004386	1.2149	0.2465	25.55
17	48535283	rs55859232	A	0.02688	0.7011	0.5119	0.9602	1.61E-08	0.84	0.79	0.89	1.65E-09	0.8347	0.0008449	0.822	0.2686	18.31
17	48536718	rs12946931	T	0.02688	0.7011	0.5119	0.9602	1.51E-08	0.84	0.79	0.89	1.65E-09	0.8347	0.0008449	0.822	0.2686	18.31
17	48537360	rs12938390	T	0.02688	0.7011	0.5119	0.9602	1.62E-08	0.84	0.79	0.89	1.65E-09	0.8347	0.0008449	0.822	0.2686	18.31
17	48537393	rs12947405	T	0.02688	1.426	1.041	1.954	4.84E-08	1.18	1.11	1.25	1.02E-08	1.1879	0.004386	1.2149	0.2465	25.55
17	48539801	rs12603976	A	0.02688	1.426	1.041	1.954	1.93E-08	1.19	1.12	1.26	1.79E-09	1.1975	0.0008944	1.2163	0.2681	18.46
17	48540097	rs12602539	A	0.02845	1.422	1.038	1.947	1.89E-08	1.19	1.12	1.26	1.83E-09	1.1974	0.0004776	1.2131	0.2759	15.76
17	48540168	rs62066693	C	0.02845	1.422	1.038	1.947	4.39E-08	1.18	1.11	1.26	1.04E-08	1.1877	0.003244	1.2119	0.2538	23.2
17	48541219	rs2326012	T	0.03209	1.411	1.03	1.934	1.31E-07	1.17	1.1	1.24	2.79E-08	1.1774	0.0052	1.2021	0.2519	23.83
17	48542733	rs1042822	T	0.03005	1.415	1.034	1.937	2.04E-08	1.19	1.12	1.26	1.87E-09	1.1972	0.0001164	1.2083	0.288	11.43
17	48542749	rs1042818	T	0.03005	0.7066	0.5163	0.967	1.99E-08	0.84	0.79	0.89	1.73E-09	0.8349	0.0001021	0.8275	0.2886	11.18
17	48543040	rs2229302	A	0.03005	1.415	1.034	1.937	1.37E-08	1.19	1.12	1.26	1.87E-09	1.1972	0.0001164	1.2083	0.288	11.43
17	48544466	rs56128659	A	0.02591	1.426	1.044	1.949	1.28E-08	1.19	1.12	1.26	1.74E-09	1.1976	0.001051	1.2175	0.2649	19.53
17	48547747	rs12951076	T	0.03022	0.7066	0.5161	0.9673	1.53E-08	0.84	0.79	0.89	1.74E-09	0.8349	9.48E-05	0.8276	0.2892	10.97
17	48559263	rs67412921	A	0.02685	1.426	1.041	1.951	7.61E-08	1.18	1.11	1.25	1.23E-08	1.188	0.004486	1.2152	0.2457	25.78
17	48560792	rs1062037	T	0.02954	0.7046	0.5141	0.9658	8.47E-08	0.85	0.8	0.9	2.25E-08	0.8444	0.004126	0.8271	0.2521	23.75
17	48568032	rs35825473	A	0.02029	1.451	1.06	1.986	3.28E-08	1.19	1.12	1.26	2.16E-09	1.1984	0.00627	1.2346	0.2242	32.32
17	48581113	rs35624684	T	0.01342	1.485	1.085	2.032	3.00E-07	1.18	1.11	1.25	2.74E-08	1.1905	0.02571	1.2549	0.1586	49.69
17	48582199	rs34527992	A	0.01043	1.508	1.101	2.065	6.28E-07	1.17	1.1	1.24	3.71E-08	1.1805	0.04474	1.265	0.1202	58.59
17	48587867	rs4793938	T	0.09571	0.7495	0.534	1.052	1.96E-07	0.84	0.79	0.9	2.63E-08	0.8367	2.63E-08	0.8367	0.5173	0
17	48587873	rs4793939	A	0.09571	1.334	0.9504	1.873	1.78E-07	1.19	1.11	1.26	2.82E-08	1.1947	2.82E-08	1.1947	0.5164	0
17	50167170	rs12939159	C	0.8874	0.9704	0.64	1.471	2.11E-08	0.82	0.77	0.88	1.08E-08	0.8235	1.08E-08	0.8235	0.4338	0
20	2095370	rs6136970	A	0.6844	0.9428	0.7098	1.252	3.77E-11	0.84	0.8	0.89	1.76E-11	0.843	1.76E-11	0.843	0.4325	0
20	2095648	rs6106210	T	0.5007	1.102	0.8308	1.462	2.85E-11	1.19	1.13	1.25	1.42E-11	1.1872	1.42E-11	1.1872	0.5997	0
20	2096964	rs2875717	A	0.664	1.065	0.8028	1.411	3.41E-09	1.17	1.11	1.23	3.06E-09	1.1664	3.06E-09	1.1664	0.5204	0
20	2097098	rs1418032	C	0.664	1.065	0.8028	1.411	1.81E-09	1.17	1.11	1.23	3.06E-09	1.1664	3.06E-09	1.1664	0.5204	0
20	2098177	rs6081944	C	0.621	1.077	0.8029	1.444	1.21E-09	1.18	1.12	1.24	5.20E-10	1.1767	5.20E-10	1.1767	0.5483	0
20	2119449	rs6137042	A	0.7994	1.041	0.7612	1.425	9.74E-10	1.18	1.12	1.25	2.02E-09	1.1758	2.02E-09	1.1758	0.4401	0
20	2119702	rs6112914	A	0.9967	0.9993	0.7308	1.367	2.07E-08	1.17	1.1	1.23	1.44E-08	1.1648	1.44E-08	1.1648	0.3304	0
20	2125680	rs6106261	T	0.8925	0.9798	0.7285	1.318	6.43E-09	1.16	1.11	1.23	2.72E-08	1.1543	0.01448	1.1385	0.2713	17.38
20	2127773	rs6112937	T	0.8462	1.03	0.7652	1.386	1.28E-10	1.18	1.12	1.25	5.30E-10	1.1753	5.30E-10	1.1753	0.377	0

20	2131744	rs35354551	T	0.1961	0.8118	0.5917	1.114	1.76E-07	0.85	0.8	0.91	3.59E-08	0.8487	3.59E-08	0.8487	0.7793	0
20	2134924	rs6137086	A	0.6978	1.059	0.7939	1.412	5.38E-10	1.18	1.12	1.24	2.35E-10	1.1761	2.35E-10	1.1761	0.4683	0
20	2135814	rs6106275	T	0.6638	1.072	0.7842	1.465	1.15E-09	1.18	1.12	1.25	1.67E-09	1.1767	1.67E-09	1.1767	0.5526	0
20	2136719	rs4814982	A	0.6978	1.059	0.7939	1.412	7.75E-10	1.17	1.12	1.23	1.81E-09	1.1665	1.81E-09	1.1665	0.504	0
20	2137705	rs6112969	T	0.75	0.9542	0.7152	1.273	7.85E-10	0.85	0.81	0.9	5.27E-10	0.853	5.27E-10	0.853	0.4389	0
20	2140501	rs6137099	T	0.9744	1.005	0.7472	1.351	1.93E-09	1.17	1.11	1.23	3.93E-09	1.1648	3.93E-09	1.1648	0.3219	0
20	2140546	rs6132337	A	0.9744	0.9952	0.74	1.338	8.72E-10	0.86	0.81	0.9	3.98E-09	0.8634	3.98E-09	0.8634	0.3409	0
20	2140582	rs6137102	T	0.7489	1.053	0.7691	1.441	9.94E-10	1.18	1.12	1.24	5.98E-10	1.1764	5.98E-10	1.1764	0.4829	0
20	2142070	rs6046998	T	0.5927	1.084	0.8059	1.459	6.73E-11	1.19	1.13	1.25	5.46E-11	1.1867	5.46E-11	1.1867	0.5439	0
20	2142388	rs6112980	T	0.9744	0.9952	0.74	1.338	4.28E-09	0.86	0.81	0.9	1.56E-08	0.8637	1.56E-08	0.8637	0.3414	0
20	2143374	rs6137114	A	0.9744	0.9952	0.74	1.338	5.34E-09	0.86	0.81	0.9	1.56E-08	0.8637	1.56E-08	0.8637	0.3414	0
20	2143478	rs6106280	T	0.9543	1.009	0.743	1.37	1.54E-09	1.18	1.12	1.24	1.67E-09	1.1746	1.67E-09	1.1746	0.3231	0
20	2144458	rs6047011	A	0.6964	0.9443	0.7081	1.259	4.74E-09	0.86	0.82	0.9	7.47E-09	0.8624	7.47E-09	0.8624	0.5308	0
20	2144907	rs3748512	T	0.9575	1.008	0.7497	1.356	3.97E-09	1.17	1.11	1.23	3.85E-09	1.1649	3.85E-09	1.1649	0.3312	0
20	2146963	rs6035685	A	0.6409	0.9341	0.7014	1.244	1.21E-09	0.86	0.82	0.9	1.69E-09	0.862	1.69E-09	0.862	0.5771	0
20	2147038	rs11087326	T	0.9575	1.008	0.7497	1.356	1.27E-09	1.17	1.11	1.23	8.83E-10	1.1653	8.83E-10	1.1653	0.3307	0
20	2147284	rs6112998	T	0.9575	1.008	0.7497	1.356	1.42E-09	1.17	1.11	1.22	8.83E-10	1.1653	8.83E-10	1.1653	0.3307	0
20	2149725	rs6113005	A	0.9674	1.006	0.7471	1.355	2.38E-09	0.86	0.82	0.9	5.66E-09	0.8637	7.66E-06	0.866	0.3087	3.51
20	2149878	rs6113006	A	0.9037	1.019	0.7567	1.371	2.23E-09	0.86	0.82	0.9	5.35E-09	0.864	0.0137	0.8765	0.2697	17.9
20	2150248	rs6106287	A	0.8317	1.034	0.7597	1.407	9.87E-10	0.85	0.81	0.9	1.18E-09	0.8545	0.09964	0.8818	0.2189	33.85
20	2150388	rs6113010	T	0.9672	1.006	0.747	1.356	1.44E-09	1.17	1.11	1.23	1.21E-09	1.1652	1.21E-09	1.1652	0.3272	0
20	2150543	rs6106288	T	0.9672	0.9938	0.7377	1.339	1.17E-09	0.86	0.81	0.9	5.22E-09	0.8634	5.22E-09	0.8634	0.3481	0
20	2150701	rs6106290	A	0.9672	1.006	0.747	1.356	1.57E-09	1.17	1.11	1.23	1.21E-09	1.1652	1.21E-09	1.1652	0.3272	0
20	2150717	rs6047049	A	0.7481	0.9539	0.7153	1.272	8.06E-09	0.87	0.82	0.91	2.93E-08	0.8723	2.93E-08	0.8723	0.5367	0
20	2151079	rs4814988	T	0.9066	0.9828	0.7353	1.314	9.28E-10	0.86	0.82	0.9	2.06E-09	0.8632	2.06E-09	0.8632	0.3741	0
20	2151115	rs4814989	T	0.9643	1.007	0.7477	1.356	2.33E-09	1.16	1.11	1.22	9.05E-09	1.1555	9.05E-09	1.1555	0.3581	0
20	2151475	rs6137153	T	0.9976	0.9995	0.7333	1.362	6.71E-10	1.18	1.12	1.24	5.39E-10	1.1747	7.58E-05	1.1684	0.3	6.91
20	2152351	rs927157	A	0.8747	0.9763	0.7249	1.315	1.74E-09	0.86	0.82	0.9	4.65E-09	0.863	4.65E-09	0.863	0.4102	0
20	2152978	rs6113016	A	0.9508	1.009	0.7494	1.36	3.39E-09	1.16	1.11	1.22	1.02E-08	1.1555	1.02E-08	1.1555	0.3656	0
20	2153022	rs6113017	A	0.9508	0.9907	0.7355	1.334	2.85E-09	0.86	0.82	0.9	5.89E-09	0.8634	5.89E-09	0.8634	0.3587	0
20	2153124	rs6047060	A	0.7513	0.9541	0.7136	1.276	3.31E-10	0.85	0.81	0.9	1.03E-10	0.8527	1.03E-10	0.8527	0.4421	0
20	2153197	rs6047061	T	0.7331	1.051	0.7886	1.401	4.62E-09	1.16	1.1	1.22	2.97E-09	1.1568	2.97E-09	1.1568	0.5072	0
20	2155259	rs6113024	A	0.7419	0.9527	0.7143	1.271	1.56E-10	0.85	0.81	0.9	8.57E-11	0.8527	8.57E-11	0.8527	0.4442	0
20	2156032	rs4813398	A	0.8309	1.033	0.7676	1.39	7.35E-11	1.18	1.12	1.24	1.23E-10	1.1757	1.23E-10	1.1757	0.3862	0
20	2157622	rs6113028	T	0.812	0.964	0.7126	1.304	1.36E-09	1.17	1.11	1.23	3.83E-09	1.1637	0.1164	1.1273	0.2156	34.79
20	2157651	rs6047102	A	0.7976	0.9612	0.7105	1.3	1.09E-09	1.18	1.12	1.24	1.14E-09	1.173	0.1644	1.1266	0.19	41.79

20	2157676	rs6113029	A	0.8892	0.9787	0.7228	1.325	7.76E-10	1.17	1.12	1.24	3.98E-09	1.1642	0.02999	1.142	0.2549	22.86
20	2157783	rs6047103	A	0.6321	1.07	0.8104	1.414	8.66E-09	1.15	1.1	1.21	1.55E-08	1.1476	1.55E-08	1.1476	0.6169	0
20	2158296	rs6113033	T	0.825	0.9664	0.714	1.308	5.24E-10	1.18	1.12	1.24	5.10E-10	1.1735	0.1277	1.1317	0.2025	38.43
20	2158319	rs6113034	A	0.825	0.9664	0.714	1.308	7.35E-10	1.17	1.12	1.24	3.76E-09	1.1638	0.09855	1.1298	0.2224	32.83
20	2158360	rs6106301	T	0.9318	1.013	0.7497	1.37	1.36E-09	0.85	0.81	0.9	9.08E-10	0.8542	0.0165	0.8692	0.2605	21.03
20	2158434	rs6113035	T	0.825	0.9664	0.714	1.308	6.36E-10	1.18	1.12	1.24	5.10E-10	1.1735	0.1277	1.1317	0.2025	38.43
20	2160408	rs6047134	A	0.6644	1.064	0.8047	1.406	2.36E-09	1.16	1.1	1.22	2.36E-09	1.1571	2.36E-09	1.1571	0.5501	0
20	2160585	rs6106303	A	0.9521	0.9907	0.7311	1.343	5.94E-10	1.18	1.12	1.24	5.85E-10	1.1742	0.01038	1.1558	0.266	19.16
20	2160874	rs6106304	C	0.8513	1.029	0.7608	1.393	6.47E-09	0.85	0.81	0.9	4.24E-09	0.8549	0.08606	0.8805	0.2222	32.88
20	2161006	rs6106305	A	0.9461	0.9896	0.731	1.34	1.32E-09	1.17	1.11	1.23	3.22E-09	1.1646	0.00247	1.1532	0.2852	12.45
20	2161559	rs3859635	A	0.7769	1.043	0.7779	1.399	6.42E-09	1.16	1.1	1.22	6.32E-09	1.1566	6.32E-09	1.1566	0.4841	0
20	2162109	rs6106307	T	0.7959	0.9628	0.7223	1.283	4.88E-09	0.86	0.82	0.91	2.81E-09	0.8628	2.81E-09	0.8628	0.4478	0
20	2162144	rs6106308	T	0.8975	0.9802	0.7229	1.329	1.02E-09	1.17	1.11	1.24	3.92E-09	1.1643	0.02186	1.1442	0.2611	20.83
20	2162324	rs6113046	T	0.8823	1.023	0.756	1.385	7.14E-10	0.85	0.81	0.9	6.06E-10	0.8543	0.05259	0.876	0.2366	28.6
20	2162616	rs6137178	A	0.9938	0.9988	0.7378	1.352	2.37E-09	0.86	0.81	0.9	9.22E-09	0.8635	9.22E-09	0.8635	0.3398	0
20	2162733	rs60871077	T	0.9583	1.008	0.7444	1.365	2.47E-09	0.85	0.81	0.9	3.20E-09	0.8543	0.004524	0.8647	0.2779	15.05
21	28160721	rs7278583	T	0.7221	0.9491	0.7118	1.266	2.38E-10	1.18	1.12	1.25	1.18E-09	1.1719	0.2852	1.1102	0.1444	53.07
21	28207549	rs2831639	A	0.9275	0.9855	0.719	1.351	1.24E-08	0.82	0.77	0.88	1.24E-08	0.8265	0.005577	0.8405	0.2637	19.94
21	28207731	rs2831640	T	0.9275	0.9855	0.719	1.351	1.20E-08	0.82	0.77	0.88	1.24E-08	0.8265	0.005577	0.8405	0.2637	19.94
21	28209424	rs2831644	A	0.9163	1.017	0.7419	1.394	1.03E-08	1.22	1.14	1.3	1.14E-08	1.2105	0.003635	1.1923	0.2688	18.21
21	28209480	rs2831645	T	0.9163	1.017	0.7419	1.394	1.02E-08	1.22	1.14	1.3	1.14E-08	1.2105	0.003635	1.1923	0.2688	18.21
21	28209704	rs2831646	T	0.9163	1.017	0.7419	1.394	1.05E-08	1.22	1.14	1.3	1.14E-08	1.2105	0.003635	1.1923	0.2688	18.21
21	28209902	rs2831647	C	0.9163	1.017	0.7419	1.394	9.46E-09	1.22	1.14	1.3	1.14E-08	1.2105	0.003635	1.1923	0.2688	18.21
21	28210002	rs2831648	T	0.7728	1.044	0.7781	1.402	4.50E-10	0.83	0.78	0.88	1.40E-09	0.8373	0.2555	0.8879	0.1342	55.43
21	28210028	rs2831649	A	0.7728	1.044	0.7781	1.402	4.23E-10	0.83	0.78	0.88	1.40E-09	0.8373	0.2555	0.8879	0.1342	55.43
21	28210407	rs2831651	T	0.7728	0.9576	0.7134	1.285	4.12E-10	1.21	1.14	1.28	5.78E-10	1.1993	0.2641	1.1274	0.1266	57.15
21	28211095	rs2831653	T	0.9707	1.006	0.7319	1.383	1.99E-08	1.21	1.13	1.3	4.72E-08	1.2005	0.007502	1.1811	0.2656	19.3
21	28211394	rs9305363	A	0.8068	1.041	0.7562	1.432	2.59E-08	1.21	1.13	1.3	4.43E-08	1.2023	4.43E-08	1.2023	0.3662	0
21	28211915	rs2831654	A	0.9968	1.001	0.73	1.372	1.66E-08	1.21	1.13	1.3	4.95E-08	1.2001	0.01938	1.1747	0.249	24.75
21	28212617	rs741874	C	0.9968	1.001	0.73	1.372	4.65E-09	1.22	1.14	1.31	1.42E-08	1.2096	0.03431	1.1763	0.2291	30.86
21	28212833	rs2092559	A	0.9919	1.002	0.7309	1.373	3.85E-09	0.82	0.76	0.87	1.55E-08	0.8272	0.04335	0.8523	0.2228	32.72
21	28213192	rs741873	A	0.9919	1.002	0.7309	1.373	2.63E-09	0.82	0.76	0.87	1.55E-08	0.8272	0.04335	0.8523	0.2228	32.72
21	28216949	rs2831659	A	0.9968	1.001	0.73	1.372	1.71E-09	1.23	1.15	1.32	4.40E-09	1.2189	0.05033	1.1782	0.2106	36.2
21	28218436	rs2409294	A	0.9968	1.001	0.73	1.372	1.95E-09	1.23	1.15	1.32	4.40E-09	1.2189	0.05033	1.1782	0.2106	36.2
21	28218535	rs2898150	A	0.9968	0.9994	0.7291	1.37	1.55E-09	0.81	0.76	0.87	2.33E-09	0.8175	0.059	0.8484	0.2016	38.67
21	28220301	rs112393037	T	0.8674	0.9729	0.7049	1.343	4.52E-09	0.82	0.76	0.87	1.98E-08	0.826	2.62E-06	0.8282	0.3089	3.4

21	28220331	rs112217758	T	0.8787	0.9752	0.7067	1.346	3.82E-09	0.81	0.76	0.87	2.59E-09	0.8165	0.002333	0.8291	0.2691	18.13
21	28220685	rs2205243	T	0.9919	1.002	0.7309	1.373	4.28E-09	0.82	0.76	0.87	2.30E-08	0.8273	0.04358	0.8524	0.223	32.65
21	28221033	rs2831661	A	0.9919	0.9984	0.7285	1.368	3.14E-09	1.23	1.15	1.32	6.17E-09	1.2186	0.05987	1.1756	0.2048	37.8
21	28221374	rs1997825	A	0.9968	0.9994	0.7291	1.37	4.62E-09	0.82	0.76	0.87	2.25E-08	0.8272	0.03496	0.8505	0.2294	30.79
21	28224218	rs13048808	T	0.7889	1.045	0.7564	1.444	5.25E-08	1.2	1.12	1.28	4.95E-08	1.1936	4.95E-08	1.1936	0.4112	0
21	28224375	rs2831666	A	0.7889	0.9568	0.6924	1.322	5.01E-08	0.83	0.78	0.89	2.52E-08	0.8346	2.52E-08	0.8346	0.3982	0
21	28229634	rs452763	C	0.5357	0.9073	0.6669	1.234	4.10E-09	0.82	0.77	0.88	1.46E-09	0.8235	1.46E-09	0.8235	0.5282	0
21	28231304	rs429864	A	0.5283	1.104	0.8115	1.502	6.25E-09	1.21	1.13	1.29	5.97E-09	1.2054	5.97E-09	1.2054	0.5678	0
21	28243015	rs118735	A	0.5633	1.095	0.8054	1.488	1.18E-08	1.2	1.13	1.28	2.02E-08	1.1955	2.02E-08	1.1955	0.567	0
21	28246674	rs448639	C	0.556	0.9119	0.6707	1.24	3.05E-10	0.82	0.77	0.87	1.85E-10	0.8233	1.85E-10	0.8233	0.5061	0
21	28247396	rs118737	T	0.556	1.097	0.8066	1.491	1.11E-10	1.22	1.15	1.3	1.70E-10	1.2151	1.70E-10	1.2151	0.5059	0
21	28248050	rs118736	A	0.5633	0.9135	0.672	1.242	2.46E-10	0.82	0.77	0.87	1.87E-10	0.8234	1.87E-10	0.8234	0.4988	0
21	28249473	rs391427	T	0.556	1.097	0.8066	1.491	1.87E-10	1.22	1.15	1.3	1.70E-10	1.2151	1.70E-10	1.2151	0.5059	0
21	28250940	rs439987	A	0.6959	0.9411	0.6942	1.276	7.31E-10	0.83	0.78	0.88	6.73E-10	0.8338	6.73E-10	0.8338	0.4271	0
22	20969069	rs756878	T	0.04764	1.313	1.003	1.72	4.01E-09	1.17	1.11	1.24	1.15E-09	1.175	1.15E-09	1.175	0.4109	0

Table S2. Gene prioritisation based on the effect of coding variants. For each gene, its coordinates, gene type, probability of being loss-of-function intolerant (pLI) score, and Independent Significant SNPs tagging it are shown, along with the variants (SNP ID) by which it is tagged. Additionally, the effect of variants on genes (exonic function), the deleteriousness score of each variant (CADD), and the Total Score are presented. The Total Score is calculated as the sum of the following: 1 point is added if the pLI is ≥ 0.9; 1 point is added if any of the SNPs affecting it has a CADD score ≥ 10, or 2 points if it is ≥ 20. Depending on the type of SNP effect, 1 point is added if it is a synonymous or a non-reading frame-changing mutation, and 2 points if it is a non-synonymous or a reading frame-changing mutation.

Gene	Coordinates	Gene type	pLI score	SNP ID	Exonic function	CADD score	Total score	Independent Significant SNPs
FNDC3B	3:171757418:172119455	protein_coding	1.00E+00	3:171969077:C:G	nonsynonymous_SNV	17.76	4	rs28641087;rs12629746;rs4243401;rs73025487;rs7653242;rs4894414
KIF6	6:39297766:39693181	protein_coding	1.04E-16	6:39563863:C:T	synonymous_SNV	11.05	2	rs10947821
PKHD1	6:51480098:51952423	protein_coding	2.89E-23	6:51613177:C:T	synonymous_SNV	8.251	1	rs9382005
INTS8	8:95825539:95893974	protein_coding	9.90E-01	8:95888307:C:T	synonymous_SNV	16.55	3	rs4734285;rs7820818;rs7819800
TP53INP1	8:95938200:95961639	protein_coding	6.86E-05	8:95951916:G:GTGAC	frameshift_substitution	0.762	2	rs7820818;rs7819800;rs4734285
C8G	9:139839698:139841426	protein_coding	1.63E-09	9:139839904:G:T	synonymous_SNV	13.54	2	rs2271872;rs2090627;rs7019538
C9orf141	9:139863695:139866442	protein_coding	NA	9:139866247:G:T	nonsynonymous_SNV	2.355	2	rs2090627;rs7019538
				9:139864341:A:G	nonsynonymous_SNV	0.813	2	
INVS	9:102861538:103063282	protein_coding	1.45E-06	9:103054951:C:T	synonymous_SNV	12.11	2	rs2806689
LCN12	9:139844003:139849949	protein_coding	1.53E-03	9:139848688:C:T	nonsynonymous_SNV	0.484	2	rs2271872;rs2090627;rs7019538
LCNL1	9:139876356:139880862	protein_coding	3.02E-03	9:139879170:A:G	nonsynonymous_SNV	17.02	3	rs2090627
KAZALD1	10:102821598:102827888	protein_coding	3.36E-04	10:102824349:C:G	nonsynonymous_SNV	22.1	4	rs807037
EPS8L2	11:694438:727727	protein_coding	1.83E-03	11:721570:C:T	nonsynonymous_SNV	22	4	rs4963153;rs4078520;rs72844798
PDDC1	11:767220:777488	protein_coding	6.17E-01	11:771034:A:G	synonymous_SNV	3.267	1	rs10902219;rs4963153;rs4078520;rs72844798;rs6597984;rs12277141
				11:772490:A:G	synonymous_SNV	0.459	1	

PIDD	11:799179:809753	protein_coding	1.60E-09	11:802379:C:T	nonsynonymous_SNV	9.021	2	rs4963153;rs12277141;rs7942159;rs72844798;rs4078520;rs10902219;rs6597984
				11:804212:A:C	synonymous_SNV	1.042	1	
PNPLA2	11:818902:825573	protein_coding	1.14E-03	11:823809:C:G	synonymous_SNV	0.768	1	rs72844798;rs12277141;rs4963153;rs7942159
SLC39A13	11:47428683:47438047	protein_coding	1.19E-02	11:47434986:A:G	synonymous_SNV	8.988	1	rs7120548
SMAD3	15:67356101:67487533	protein_coding	8.68E-01	15:67457335:A:G	nonsynonymous_SNV	13.17	3	rs2289263;rs62006055;rs12912045;rs1065080;rs744910;rs7183244;rs35251008;rs55994097;rs1037039
ALDH3A1	17:19641297:19652256	protein_coding	1.33E-12	17:19642952:C:G	nonsynonymous_SNV	16.38	3	rs4646785
CAMTA2	17:4871287:4890960	protein_coding	1.00E+00	17:4883818:C:G	nonsynonymous_SNV	0.001	3	rs12603055;rs2586527;rs427703;rs4609912;rs12600367;rs2243090;rs7208035
ENO3	17:4851387:4860426	protein_coding	6.91E-11	17:4856580:C:T	nonsynonymous_SNV	22.6	4	rs12603055;rs2586527;rs2243090;rs7208035;rs427703;rs12600367
				17:4856376:A:G	nonsynonymous_SNV	22.2	4	
HOXB1	17:46605888:46608359	protein_coding	1.15E-01	17:46608184:G:GGGGCGCTGT	nonframeshift_substitution	17.92	2	rs7225995
				17:46607958:A:T	nonsynonymous_SNV	8.903	2	rs7225995
				17:46608030:A:G	synonymous_SNV	5.379	1	rs7225995
				17:46607817:C:T	synonymous_SNV	1.066	1	rs7225995
MINK1	17:4736683:4801356	protein_coding	1.00E+00	17:4797305:A:G	nonsynonymous_SNV	19.07	4	rs4609912;rs2243090;rs12603055;rs2586527;rs427703;rs12600367
SLC52A1	17:4935895:4955304	protein_coding	2.74E-05	17:4936898:C:T	nonsynonymous_SNV	18.45	3	rs12603055;rs12600367;rs7208035;rs2243090;rs2586527;rs427703
				17:4936972:A:G	nonsynonymous_SNV	12.87	3	

Table S3. Gene prioritisation based on the effect of non-coding variants. For each gene, its coordinates, gene type, probability of being loss-of-function intolerant (pLI) score, non-coding residual variation intolerance score (ncRVIS) score, and Independent Significant SNPs tagging it are shown. Additionally, it is indicated whether the gene is tagged by any SNP with expression quantitative trait locus (eQTL) effect, any SNP with available chromatin interaction (CI) data, SNPs with deleteriousness (CADD) score higher than 10 (Yes) or 20 (Yes§), or any SNP with a regulomeDB (RDB) score of 4 or 3 (Yes) or 2 or 1 (Yes*). The total score is calculated as the sum of the following: 1 point is added if the pLI is ≥ 0.9; 1 point if any of the SNPs affecting it has a CADD score ≥ 10, or 2 points if it is ≥ 20; 1 point if the ncRVIS score of the gene is < 0; 1 point if the gene is protein coding; 1 point if it is affected by an eQTL SNP; 1 point if affected by an SNP with an RDB score of 4 or 3, or 2 points if it is 1 or 2; and finally, 1 point if there is available chromatin interaction data for any of the SNPs tagging it.

Gene	Coordinates	pLI score	ncRVIS score	Gene type	eQTL effect	CI effect	CADD score	RDB score	Total score	Independent Significant SNPs
ATP1B1	1:169074935:169101960	9.96E-01	6.38E-01	protein_coding	No	No	No	Yes	3	rs1200108
C1orf132	1:207974863:208042495	NA	NA	processed_transcript	No	No	Yes	Yes*	3	rs761276
CACYBP	1:174968300:174980851	9.43E-01	3.05E-01	protein_coding	No	No	Yes	Yes*	5	rs6669560
CD46	1:207925402:207968858	1.92E-04	-6.02E-01	protein_coding	Yes	No	Yes	Yes*	6	rs761276
CR1L	1:207818458:207911761	3.56E-15	1.16E-01	protein_coding	No	No	Yes	Yes	3	rs761276
LINC00970	1:168873143:169056243	NA	NA	antisense	No	No	Yes	Yes*	3	rs1200108
MRPS14	1:174979925:174992561	1.66E-03	-3.53E-02	protein_coding	No	No	No	Yes	3	rs6669560
RABGAP1L	1:174128548:174964445	8.92E-02	NA	protein_coding	Yes	No	Yes	No	3	rs6669560;rs997985
RC3H1	1:173900352:173991435	1.00E+00	5.24E-01	protein_coding	Yes	No	No	No	3	rs6669560
RNA5SP66	1:169036502:169036602	NA	NA	rRNA	No	No	No	No	0	rs1200108
RNU6-307P	1:174965661:174965766	NA	NA	snRNA	No	No	No	Yes	1	rs6669560
RP1-102G20.5	1:174904084:174923398	NA	NA	antisense	No	No	Yes	No	1	rs6669560
RP11-454L1.2	1:207935929:207936608	NA	NA	pseudogene	No	No	No	No	0	rs761276

RP5-1018K9.1	1:169073362:169074145	NA	NA	antisense	No	No	Yes	Yes	2	rs1200108
SDCCAG3P2	1:175013762:175014784	NA	NA	pseudogene	No	No	No	Yes	1	rs997985
LRP1B	2:140988992:142889270	1.00E+00	-1.65E-01	protein_coding	No	No	Yes	Yes	5	rs116792882
FNDC3B	3:171757418:172119455	1.00E+00	6.15E-01	protein_coding	No	No	Yes	Yes*	5	rs28641087;rs12629746;rs4243401;rs73025487;rs7653242;rs4894414
PLD1	3:171318195:171528740	7.21E-16	-6.46E-01	protein_coding	Yes	No	No	No	3	rs17480104
RBMS3	3:29322473:30051886	7.41E-01	1.27E+00	protein_coding	No	No	No	Yes	2	rs11129361
RN7SL141P	3:171955116:171955384	NA	NA	misc_RNA	No	No	No	No	0	rs4243401;rs7653242;rs28641087;rs12629746
RP11-423E7.1	3:171943218:171943662	NA	NA	pseudogene	No	No	No	No	0	rs7653242;rs4243401;rs28641087;rs4894414;rs12629746
TMEM212	3:171561139:171656505	NA	-5.26E-01	protein_coding	No	No	Yes	Yes*	5	rs17480104;rs9870071
TMEM212-AS1	3:171594142:171618530	NA	NA	lincRNA	No	No	Yes	Yes*	3	rs17480104;rs9870071
TMEM212-IT1	3:171612226:171613030	NA	NA	sense_intronic	No	No	No	No	0	rs9870071
CTD-2366F13.1	5:52405672:52410956	NA	NA	antisense	Yes	No	Yes	Yes*	4	rs10471373;rs6867917;rs56909170;rs7737693;rs252035;rs254617
FST	5:52776239:52782964	9.64E-01	NA	protein_coding	No	Yes	No	No	3	rs252035
LOX	5:121398890:121413980	9.79E-01	-1.44E+00	protein_coding	Yes	No	Yes	Yes	6	rs41490044;rs2731657;rs2914598
MOCS2	5:52391509:52405893	4.05E-02	-2.96E-01	protein_coding	Yes	No	No	No	3	rs56909170;rs252035
RP11-94D20.1	5:52502391:52503006	NA	NA	pseudogene	No	No	Yes	Yes*	3	rs7737693;rs6867917;rs252035;rs56909170;rs10471373;rs254617
SRFBP1	5:121297656:121411265	8.19E-05	4.50E-01	protein_coding	No	No	Yes	Yes	3	rs2914598;rs41490044;rs2731657
AL079342.1	6:89674246:89676685	NA	NA	protein_coding	No	No	No	No	1	rs1321085
FTH1P5	6:50880425:50880969	NA	NA	pseudogene	No	No	Yes	Yes	2	rs72891726;rs6904450
KIF6	6:39297766:39693181	1.04E-16	5.67E+00	protein_coding	No	No	Yes	No	2	rs10947821

PKHD1	6:51480098:51952423	2.89E-23	1.41E-02	protein_coding	Yes	No	Yes[§]	Yes	5	rs9382005
RNGTT	6:89319985:89673348	7.63E-01	7.69E-02	protein_coding	Yes	Yes	Yes	Yes*	6	rs1321085
RNU1-54P	6:39588121:39588240	NA	NA	snRNA	No	No	No	No	0	rs10947821
RP4-753D5.3	6:50865328:50865938	NA	NA	pseudogene	No	No	Yes	Yes	2	rs6904450;rs72891726
RPS17P5	6:50824968:50825375	NA	NA	pseudogene	No	No	Yes	Yes	2	rs6904450
SRSF12	6:89805678:89827800	1.62E-02	1.06E+00	protein_coding	No	Yes	No	No	2	rs1321085
TFAP2B	6:50786436:50815326	9.91E-01	1.92E-01	protein_coding	No	No	Yes	Yes*	5	rs72891726;rs6904450
C8orf37	8:96257147:96281429	2.18E-03	1.13E+00	protein_coding	No	Yes	No	No	2	rs7819800
CCNE2	8:95891998:95908906	3.38E-01	8.80E-02	protein_coding	No	No	Yes	Yes	3	rs4734285;rs7820818;rs7819800
CTC-756D1.2	8:23316114:23342484	NA	NA	lincRNA	Yes	No	No	No	1	rs73228208
DPY19L4	8:95731931:95806064	1.51E-15	-2.91E-01	protein_coding	No	No	No	No	2	rs4734285
ENTPD4	8:23243296:23315208	2.07E-11	1.39E+00	protein_coding	No	Yes	No	No	2	rs73228208
INTS8	8:95825539:95893974	9.90E-01	-2.40E-01	protein_coding	No	No	No	Yes*	5	rs4734285;rs7820818;rs7819800
MIR3150A	8:96079036:96085410	NA	NA	antisense	Yes	No	No	No	1	rs7820818
NDUFAF6	8:95907995:96128683	2.91E-05	1.27E-01	protein_coding	Yes	No	Yes	Yes*	5	rs7820818;rs7819800;rs4734285
PLEKHF2	8:96146032:96168912	7.50E-01	-7.20E-02	protein_coding	No	Yes	No	No	3	rs7819800
RP11-347C18.1	8:95903467:95904335	NA	NA	pseudogene	No	No	No	No	0	rs7820818;rs7819800
RP11-347C18.3	8:95962265:95963624	NA	NA	sense_intronic	No	No	No	No	0	rs7820818;rs7819800
RP11-347C18.4	8:95986801:95987081	NA	NA	pseudogene	No	No	Yes	No	1	rs7820818;rs7819800;rs4734285
RP11-347C18.5	8:95896837:95897298	NA	NA	antisense	No	No	No	No	0	rs4734285;rs7820818
SLC25A37	8:23386318:23432976	5.12E-01	9.64E-01	protein_coding	Yes	Yes	Yes	Yes	5	rs73228208
TP53INP1	8:95938200:95961639	6.86E-05	-9.65E-01	protein_coding	Yes	Yes	Yes	Yes*	7	rs7820818;rs7819800;rs4734285

ABCA2	9:139901686:139923367	1.00E+00	NA	protein_coding	Yes	Yes	Yes	Yes*	7	rs2090627
C8G	9:139839698:139841426	1.63E-09	-1.74E-01	protein_coding	No	No	No	Yes*	4	rs2271872;rs2090627;rs7019538
C9orf141	9:139863695:139866442	NA	NA	protein_coding	No	No	Yes	Yes*	4	rs2090627;rs7019538
C9orf142	9:139886870:139888436	5.70E-01	NA	protein_coding	No	Yes	No	Yes	3	rs2090627
C9orf172	9:139738867:139741797	NA	NA	protein_coding	Yes	No	No	No	2	rs2271872;rs2090627;rs7019538
CLIC3	9:139889087:139891255	3.88E-04	-5.02E-01	protein_coding	Yes	Yes	No	Yes*	6	rs2090627
COL5A1	9:137533620:137736686	1.00E+00	8.09E-01	protein_coding	No	No	No	Yes*	4	rs56361301
COL5A1-AS1	9:137541231:137544689	NA	NA	antisense	No	No	No	Yes	1	rs56361301
DPP7	9:140004994:140009629	2.02E-15	NA	protein_coding	Yes	No	No	No	2	rs2090627;rs7019538;rs2271872
EDF1	9:139756571:139760738	9.09E-01	NA	protein_coding	Yes	No	No	No	3	rs2271872;rs2090627
ERP44	9:102741461:102861322	9.57E-01	-1.15E+00	protein_coding	No	No	Yes	Yes*	6	rs2806689
FAM206A	9:111696461:111713024	3.36E-03	-4.67E-01	protein_coding	Yes	No	No	No	3	rs7859737
FBXW5	9:139834887:139839148	1.16E-08	NA	protein_coding	Yes	No	No	Yes	3	rs2271872;rs2090627;rs7019538
GRIN1	9:140032842:140063207	9.73E-01	NA	protein_coding	Yes	No	No	No	3	rs2090627
INVS	9:102861538:103063282	1.45E-06	-1.04E-01	protein_coding	Yes	No	Yes	Yes*	6	rs2806689
LCN12	9:139844003:139849949	1.53E-03	-1.28E-01	protein_coding	No	No	Yes	Yes*	5	rs2271872;rs2090627;rs7019538
LCNL1	9:139876356:139880862	3.02E-03	-8.58E-01	protein_coding	Yes	No	No	Yes	4	rs2090627
MAN1B1	9:139981379:140003635	2.63E-06	-4.93E-01	protein_coding	Yes	No	No	No	3	rs2090627
MSANTD3	9:103189438:103213511	6.30E-02	NA	protein_coding	Yes	No	No	No	2	rs2806689
NANOGP5	9:102937460:102938336	NA	NA	pseudogene	No	No	No	No	0	rs2806689
NCLP1	9:139707240:139709988	NA	NA	pseudogene	Yes	No	No	No	1	rs2090627
NELFB	9:140149625:140167998	7.49E-01	NA	protein_coding	Yes	No	No	No	2	rs2090627

PHPT1	9:139743176:139745488	NA	NA	protein_coding	Yes	No	No	No	2	rs2271872;rs2090627;rs7019538
PTGDS	9:139871956:139879887	8.87E-03	-3.38E-01	protein_coding	No	No	No	No	2	rs2090627;rs7019538
RN7SL75P	9:102864841:102865119	NA	NA	misc_RNA	No	No	No	No	0	rs2806689
RP11-220D10.1	9:111389113:111389430	NA	NA	pseudogene	No	No	Yes	Yes	2	rs7038482;rs10816690
RP11-229P13.19	9:139869546:139871433	NA	NA	processed_transcript	No	Yes	No	Yes	2	rs7019538;rs2090627
RP11-229P13.2	9:139830292:139831613	NA	NA	pseudogene	No	No	No	No	0	rs2271872;rs2090627
RP11-229P13.25	9:139831621:139832440	NA	NA	sense_overlapping	No	No	No	Yes	1	rs2271872;rs2090627
RP11-29B11.5	9:102882717:102883182	NA	NA	pseudogene	No	No	No	No	0	rs2806689
RP11-339N8.1	9:111463650:111465372	NA	NA	lincRNA	No	No	Yes	Yes	2	rs7859737;rs7860325
RP11-473E2.2	9:137397318:137413288	NA	NA	lincRNA	No	No	No	No	0	rs3132307;rs9409902;rs4391503
RP11-473E2.3	9:137444584:137445743	NA	NA	lincRNA	No	No	Yes	Yes	2	rs4391503;rs3132307;rs3118518;rs3132304
RP11-473E2.4	9:137419028:137437090	NA	NA	lincRNA	No	No	Yes	Yes*	3	rs3132307;rs9409902;rs4391503;rs3118518;rs3132304
RPS2P35	9:102879037:102879933	NA	NA	pseudogene	No	No	No	No	0	rs2806689
STX17	9:102668915:102732618	8.50E-02	-9.90E-01	protein_coding	Yes	No	No	Yes	4	rs2806689
TEX10	9:103064359:103115221	1.00E+00	-6.24E-01	protein_coding	Yes	No	No	No	4	rs2806689
TMEFF1	9:103204560:103339918	5.52E-01	NA	protein_coding	Yes	No	No	No	2	rs2806689
TMEM245	9:111777432:111882225	3.62E-04	2.30E+00	protein_coding	Yes	No	No	No	2	rs7859737;rs7860325
TRAF2	9:139776364:139821059	9.96E-01	-1.69E-01	protein_coding	No	No	No	No	3	rs2271872
UPF3AP3	9:102760583:102761351	NA	NA	pseudogene	No	No	No	No	0	rs2806689
AL157788.1	10:120765302:120765411	NA	NA	miRNA	No	No	Yes	Yes*	3	rs11198725;rs10787897
EIF3A	10:120794356:120840316	1.00E+00	-9.06E-01	protein_coding	No	Yes	Yes	Yes*	7	rs10886377;rs10787897
FAM45A	10:120863598:120897496	2.43E-04	NA	protein_coding	Yes	No	Yes	Yes*	5	rs10886377;rs11198803

NANOS1	10:120789228:120793854	1.81E-01	-1.24E+00	protein_coding	Yes	No	Yes	Yes*	6	rs10787897;rs10886377
RP11-108L7.15	10:102819786:102820762	NA	NA	lincRNA	No	No	No	No	0	rs807037
RP11-435O11.5	10:120852652:120852853	NA	NA	pseudogene	No	No	Yes	Yes	2	rs10886377;rs11198803
RP11-498J9.2	10:120763048:120763396	NA	NA	antisense	No	No	No	No	0	rs11198725;rs10787897
SFXN4	10:120900279:120925179	1.48E-04	NA	protein_coding	Yes	No	No	No	2	rs10886377;rs11198803
SNORA19	10:120819523:120819650	NA	NA	snoRNA	No	No	No	No	0	rs10886377
AC090559.1	11:47507374:47507465	NA	NA	miRNA	No	No	No	No	0	rs7120548
AC090559.2	11:47407633:47407733	NA	NA	miRNA	No	No	No	No	0	rs7120548
AGBL2	11:47681143:47736941	6.81E-12	-7.66E-01	protein_coding	No	No	No	No	2	rs7120548
AP006621.1	11:780117:780755	NA	NA	pseudogene	No	No	No	Yes	1	rs4963153;rs4078520;rs10902219;rs72844798;rs6597984;rs12277141
AP006621.5	11:777578:784297	NA	NA	protein_coding	Yes	No	No	No	2	rs10902219;rs4963153;rs4078520;rs72844798;rs6597984;rs12277141;rs7942159
AP006621.6	11:781645:782105	NA	NA	antisense	Yes	No	No	No	1	rs4963153;rs4078520;rs10902219;rs72844798;rs6597984;rs12277141;rs7942159
AP006621.8	11:823634:832883	NA	NA	antisense	No	No	Yes	No	1	rs4963153;rs7942159;rs12277141;rs72844798
AP006621.9	11:708564:727047	NA	NA	antisense	No	No	Yes	Yes*	3	rs4963153;rs4078520;rs72844798
ARHGAP1	11:46698630:46722165	1.86E-01	-3.89E-01	protein_coding	Yes	No	No	No	3	rs7120548
B4GALNT4	11:369796:382116	8.17E-01	NA	protein_coding	Yes	No	No	No	2	rs7942159
C1QTNF4	11:47611216:47616211	6.79E-01	NA	protein_coding	Yes	No	Yes	No	3	rs7120548
CD151	11:832843:839831	1.64E-03	NA	protein_coding	Yes	No	No	No	2	rs72844798;rs12277141;rs7942159;rs10902219;rs4963153;rs4078520;rs6597984

CELF1	11:47487496:47587121	9.99E-01	-3.76E-01	protein_coding	Yes	No	Yes	Yes*	7	rs7120548
CEND1	11:787104:790123	5.84E-01	-6.78E-02	protein_coding	Yes	No	No	Yes	4	rs10902219;rs72844798;rs4963153;rs4078520;rs6597984;rs12277141;rs7942159
CHID1	11:867357:915058	7.26E-04	-1.19E+00	protein_coding	Yes	No	No	No	3	rs4963153;rs4078520;rs6597984;rs12277141;rs7942159;rs72844798
DDB2	11:47236493:47260767	5.12E-03	6.77E-01	protein_coding	Yes	No	No	No	2	rs7120548
EFCAB4A	11:826144:831991	1.86E-03	6.21E-01	protein_coding	Yes	No	No	No	2	rs7942159;rs12277141;rs72844798;rs10902219;rs4963153;rs4078520;rs6597984
EPS8L2	11:694438:727727	1.83E-03	NA	protein_coding	Yes	No	Yes	Yes*	5	rs4963153;rs4078520;rs72844798
FAM180B	11:47608198:47610746	3.16E-01	NA	protein_coding	No	No	No	No	1	rs7120548
FNBP4	11:47738072:47788995	1.00E+00	-7.14E-01	protein_coding	Yes	No	Yes	Yes	6	rs7120548
KBTBD4	11:47593749:47600567	1.81E-02	-4.93E-01	protein_coding	No	No	No	Yes*	4	rs7120548
KRTAP5-3	11:1628795:1629693	2.63E-01	8.42E-01	protein_coding	Yes	No	No	No	2	rs4078520
MADD	11:47290712:47351582	9.02E-04	-3.75E-01	protein_coding	Yes	No	No	No	3	rs7120548
MIR4487	11:47422521:47422593	NA	NA	miRNA	No	No	No	No	0	rs7120548
MTCH2	11:47638867:47664175	1.67E-01	3.69E-02	protein_coding	No	No	No	Yes*	3	rs7120548
MYBPC3	11:47352957:47374253	1.35E-06	-4.32E-01	protein_coding	No	Yes	No	No	3	rs7120548
NDUFS3	11:47586888:47606114	4.71E-02	-1.23E-02	protein_coding	No	No	No	Yes	3	rs7120548
NR1H3	11:47269851:47290396	8.56E-01	6.58E-01	protein_coding	Yes	No	No	No	2	rs7120548
NUP160	11:47799639:47870107	2.40E-02	-3.46E-01	protein_coding	Yes	No	No	Yes*	5	rs7120548
PACSIN3	11:47199076:47207994	1.68E-02	-5.15E-01	protein_coding	Yes	No	No	No	3	rs7120548
PDDC1	11:767220:777488	6.17E-01	-1.50E+00	protein_coding	Yes	No	No	Yes*	5	rs10902219;rs4963153;rs4078520;rs72844798;rs6597984;rs12277141

PIDD	11:799179:809753	1.60E-09	NA	protein_coding	Yes	No	Yes	Yes*	5	rs4963153;rs12277141;rs7942159;rs72844798;rs4078520;rs10902219;rs6597984
PNPLA2	11:818902:825573	1.14E-03	-5.86E-01	protein_coding	No	No	Yes	Yes	4	rs72844798;rs12277141;rs4963153;rs7942159
POLR2L	11:837356:842545	5.33E-01	NA	protein_coding	Yes	No	No	No	2	rs10902219;rs72844798;rs7942159
PSMC3	11:47440320:47447993	9.79E-01	-4.05E-01	protein_coding	No	No	No	Yes*	5	rs7120548
PTPMT1	11:47586982:47595013	4.59E-01	NA	protein_coding	No	No	No	No	1	rs7120548
RAPSN	11:47459308:47470730	3.26E-02	4.88E-02	protein_coding	Yes	No	No	Yes*	4	rs7120548
RN7SL652P	11:47579088:47579385	NA	NA	misc_RNA	No	No	No	No	0	rs7120548
RNA5SP340	11:47825848:47825954	NA	NA	rRNA	No	No	No	No	0	rs7120548
RNU5E-10P	11:47598023:47598140	NA	NA	snRNA	No	No	No	No	0	rs7120548
RNU6-1302P	11:47461557:47461617	NA	NA	snRNA	No	No	No	No	0	rs7120548
RP11-1391J7.1	11:856880:859795	NA	NA	antisense	Yes	No	No	No	1	rs4963153;rs4078520;rs10902219;rs72844798;rs7942159
RP11-692M12.5	11:47817721:47817931	NA	NA	pseudogene	No	No	No	No	0	rs7120548
RP11-750H9.5	11:47404699:47430741	NA	NA	antisense	No	No	Yes	Yes*	3	rs7120548
RP11-750H9.7	11:47535157:47536441	NA	NA	sense_intronic	No	No	No	No	0	rs7120548
RP11-793I11.1	11:47926875:47927159	NA	NA	pseudogene	No	No	Yes	Yes*	3	rs7120548
RPLP2	11:809647:812880	7.09E-01	-7.54E-01	protein_coding	Yes	No	Yes	Yes*	6	rs12277141;rs4963153;rs7942159;rs72844798;rs4078520;rs10902219;rs6597984
SLC25A22	11:790475:798316	5.68E-01	-3.27E-01	protein_coding	Yes	No	Yes	Yes*	6	rs4963153;rs4078520;rs12277141;rs10902219;rs6597984;rs72844798;rs7942159
SLC39A13	11:47428683:47438047	1.19E-02	-3.32E-01	protein_coding	Yes	Yes	No	No	4	rs7120548

SNORA52	11:811681:811814	NA	NA	snoRNA	No	No	No	No	0	rs4963153;rs12277141;rs7942159;rs72844798
snoU13	11:47749098:47749189	NA	NA	snoRNA	No	No	No	No	0	rs7120548
SPI1	11:47376411:47400127	8.97E-01	NA	protein_coding	No	Yes	No	No	2	rs7120548
TALDO1	11:747329:765024	1.76E-04	NA	protein_coding	No	No	No	Yes*	3	rs10902219;rs4078520;rs4963153;rs72844798
TMEM80	11:695428:705028	4.91E-05	NA	protein_coding	Yes	No	No	No	2	rs10902219;rs72844798;rs12277141
CELA1	12:51722227:51740463	1.30E-01	2.17E-01	protein_coding	No	No	No	No	1	rs3782473
GALNT6	12:51745031:51786651	1.66E-06	-5.88E-01	protein_coding	Yes	No	Yes	Yes*	6	rs3782473
POU6F1	12:51580719:51611477	9.08E-01	5.18E-01	protein_coding	Yes	No	No	No	3	rs3782473
AL133318.1	13:41111138:41111323	NA	NA	protein_coding	No	No	Yes	Yes	3	rs2721052;rs2755238;rs2755233;rs7331857;rs2755237;rs74948688
AL391384.1	13:73425226:73425307	NA	NA	miRNA	No	No	No	No	0	rs55761542
BORA	13:73302061:73330336	6.93E-04	-5.19E-01	protein_coding	Yes	No	No	No	3	rs55761542
DIS3	13:73329540:73356234	2.05E-11	5.70E-01	protein_coding	No	No	No	Yes	2	rs55761542
ELF1	13:41506056:41635576	5.48E-01	-4.76E-01	protein_coding	No	No	No	No	2	rs113204272
FOXO1	13:41129804:41240734	9.66E-01	-1.02E+00	protein_coding	No	No	Yes	Yes	5	rs7331857;rs2701895;rs2755238;rs113204272;rs74948688
KLF5	13:73629114:73651676	8.72E-01	-9.59E-01	protein_coding	No	No	Yes	Yes	4	rs55761542;rs17285550
LINC00598	13:41025131:41055143	NA	NA	lincRNA	Yes	No	Yes	Yes	3	rs9603762;rs2755238;rs2755233
MTRF1	13:41790505:41837742	1.54E-07	-2.64E-01	protein_coding	Yes	No	Yes	No	4	rs7331857
NAA16	13:41885341:41951166	1.71E-10	4.30E-01	protein_coding	No	No	No	Yes	2	rs9566743
OR7E155P	13:42013952:42015035	NA	NA	pseudogene	No	No	No	No	0	rs9566743
OR7E36P	13:42005425:42006446	NA	NA	pseudogene	No	No	No	No	0	rs9566743
OR7E37P	13:42016906:42017786	NA	NA	pseudogene	No	No	No	No	0	rs9566743

PIBF1	13:73356197:73590591	4.98E-08	-3.13E-01	protein_coding	Yes	No	Yes	No	4	rs55761542
PSMD10P3	13:73610539:73610986	NA	NA	pseudogene	No	No	Yes	No	1	rs55761542
RAC1P3	13:41861877:41862456	NA	NA	pseudogene	No	No	Yes	Yes	2	rs9566743
RLIMP1	13:41192875:41195485	NA	NA	pseudogene	No	No	No	No	0	rs74948688
RNU6-57P	13:41928693:41928795	NA	NA	snRNA	No	No	No	No	0	rs9566743
RNU6-79P	13:73455246:73455352	NA	NA	snRNA	No	No	No	No	0	rs55761542
SUGT1P3	13:41482295:41495910	NA	NA	pseudogene	Yes	No	No	No	1	rs113204272
TPTE2P5	13:41396432:41495890	NA	NA	pseudogene	Yes	No	No	No	1	rs2755238;rs2755237;rs74948688;rs113204272
TUBBP2	13:41958909:41959319	NA	NA	pseudogene	Yes	No	No	No	1	rs9566743
WBP4	13:41635410:41658137	4.82E-04	-6.69E-01	protein_coding	No	No	No	No	2	rs113204272
AAGAB	15:67493371:67547533	8.10E-03	1.97E-01	protein_coding	No	No	Yes	Yes*	4	rs1037039;rs55994097
AP4E1	15:51200869:51298097	9.80E-01	-1.61E-01	protein_coding	Yes	No	Yes	Yes*	7	rs35439349;rs11634895
DCAF13P3	15:51236860:51238193	NA	NA	pseudogene	Yes	No	Yes	No	2	rs35439349;rs11634895
IQCH	15:67547138:67794598	2.69E-12	-1.05E+00	protein_coding	Yes	No	Yes[§]	Yes*	7	rs1037039;rs55994097;rs6494654;rs35251008;rs12912045
IQCH-AS1	15:67695957:67814182	NA	NA	lincRNA	Yes	No	No	Yes*	3	rs6494654
MAP2K5	15:67835047:68099461	4.76E-01	NA	protein_coding	Yes	No	No	No	2	rs12912045;rs35251008;rs55994097;rs1037039;rs6494654
RP11-108K3.1	15:51329685:51586109	NA	NA	lincRNA	No	No	Yes	Yes*	3	rs11634895;rs35439349
RP11-342M21.2	15:67435072:67439277	NA	NA	protein_coding	No	Yes	No	No	2	rs2289263;rs62006055;rs12912045;rs1065080;rs744910;rs1065080:rs12912045
RP11-394B5.2	15:51356806:51361783	NA	NA	sense_intronic	No	No	No	No	0	rs35439349;rs11634895
RP11-502I4.3	15:67833410:67834942	NA	NA	lincRNA	Yes	No	No	No	1	rs6494654;rs55994097;rs1037039

RP11-507J18.1	15:51090867:51091148	NA	NA	pseudogene	No	No	Yes	Yes*	3	rs35439349
RPS24P16	15:67524784:67525175	NA	NA	pseudogene	No	No	Yes	No	1	rs1037039
SKOR1	15:68112042:68126899	NA	-1.28E-01	protein_coding	Yes	No	No	No	3	rs1037039;rs55994097;rs6494654
SMAD3	15:67356101:67487533	8.68E-01	-9.16E-02	protein_coding	Yes	No	Yes	Yes*	6	rs2289263;rs62006055;rs12912045;rs1065080;rs744910;rs7183244;rs35251008;rs55994097;rs1037039
SPPL2A	15:50999506:51058005	4.71E-02	-6.22E-01	protein_coding	Yes	No	Yes	Yes	5	rs35439349;rs11634895
TNFAIP8L3	15:51348795:51397473	2.33E-06	-7.55E-01	protein_coding	No	No	No	No	2	rs35439349;rs11634895
LA16c-444G7.1	16:88268391:88336117	NA	NA	lincRNA	No	No	No	Yes*	2	rs11860207;rs56124600;rs35542380;rs9938961
RP11-863P13.3	16:88210904:88219696	NA	NA	lincRNA	Yes	No	No	No	1	rs35542380;rs9938961
ZNF469	16:88493879:88507165	NA	4.59E-01	protein_coding	Yes	No	No	Yes*	4	rs35542380;rs9938961
AC003101.1	17:29898161:29902678	1.13E-01	NA	protein_coding	No	No	No	No	1	rs56161228
AC004771.1	17:4890302:4890972	NA	NA	pseudogene	No	No	Yes§	Yes	3	rs12603055;rs2586527;rs427703
AC005722.4	17:19658557:19659520	NA	NA	pseudogene	No	No	No	No	0	rs4646785
AC012146.7	17:5014763:5017674	NA	NA	processed_transcript	Yes	No	No	No	1	rs2243090;rs7208035
AC090627.1	17:46371709:46386124	NA	NA	antisense	No	No	Yes	Yes	2	rs35533148
AC109333.10	17:4922509:4923388	NA	NA	antisense	No	No	No	No	0	rs12603055
ALDH3A1	17:19641297:19652256	1.33E-12	-1.14E-01	protein_coding	Yes	No	Yes	Yes*	6	rs4646785
ATP6V0CP1	17:4760052:4760669	NA	NA	pseudogene	No	No	No	No	0	rs4609912;rs2243090
B4GALNT2	17:47209822:47247351	9.64E-07	-5.18E-01	protein_coding	Yes	No	No	No	3	rs7225995
C17orf107	17:4802713:4806227	NA	NA	protein_coding	Yes	Yes	No	No	3	rs4609912;rs2243090;rs7208035;rs12603055;rs2586527
CAMTA2	17:4871287:4890960	1.00E+00	-1.19E+00	protein_coding	Yes	No	Yes	Yes*	7	rs12603055;rs2586527;rs427703;rs4609912;r

										s12600367;rs2243090;rs7208035
CDK5RAP3	17:46045176:46059140	2.87E-12	4.41E-01	protein_coding	Yes	No	No	No	2	rs35533148;rs7225995;rs4793938
CHRNE	17:4801069:4806369	3.48E-04	2.19E+00	protein_coding	Yes	No	Yes	Yes*	5	rs4609912;rs2243090;rs12603055;rs2586527;rs7208035;rs427703
COL1A1	17:48260650:48278993	1.00E+00	2.41E+00	protein_coding	Yes	No	No	Yes*	5	rs12939159
COPZ2	17:46103533:46115392	1.71E-03	NA	protein_coding	Yes	No	No	No	2	rs35533148;rs4793938
CTC-524C5.2	17:5328459:5336196	NA	NA	antisense	Yes	No	No	No	1	rs4609912;rs2243090;rs7208035
CTD-2377D24.2	17:46767843:46767986	NA	NA	pseudogene	No	No	No	No	0	rs4793938
CTD-2377D24.8	17:46760729:46781844	NA	NA	lincRNA	No	No	No	No	0	rs4793938
CXCL16	17:4636821:4643217	4.32E-02	1.53E+00	protein_coding	No	Yes	No	No	2	rs12603055;rs2586527
ENO3	17:4851387:4860426	6.91E-11	1.47E-01	protein_coding	Yes	No	Yes	Yes	4	rs12603055;rs2586527;rs2243090;rs7208035;rs427703;rs12600367
GGT6	17:4460222:4464113	2.46E-06	1.23E-01	protein_coding	No	Yes	No	No	2	rs2243090
GP1BA	17:4835592:4838325	1.32E-02	-3.55E-01	protein_coding	Yes	No	Yes	Yes*	6	rs2243090;rs12603055;rs2586527;rs7208035;rs4609912;rs427703;rs12600367
HILS1	17:48248789:48258539	NA	NA	pseudogene	Yes	No	No	Yes	2	rs12939159
HOXB1	17:46605888:46608359	1.15E-01	-7.96E-01	protein_coding	No	No	Yes	Yes*	5	rs7225995
HOXB2	17:46618256:46623441	1.84E-03	9.74E-01	protein_coding	Yes	No	Yes§	Yes*	6	rs7225995;rs35533148;rs4793938
HOXB3	17:46626232:46682274	6.11E-01	-1.02E+00	protein_coding	Yes	No	No	No	3	rs7225995;rs4793938;rs35533148
HOXB4	17:46652875:46657473	1.20E-01	-1.09E+00	protein_coding	No	No	No	No	2	rs7225995;rs4793938
HOXB5	17:46668619:46671323	4.81E-01	-8.72E-02	protein_coding	No	No	No	No	2	rs7225995;rs4793938
HOXB6	17:46671639:46682354	9.58E-03	-1.66E-02	protein_coding	No	No	No	No	2	rs4793938
HOXB7	17:46684594:46710934	7.60E-03	-3.76E-01	protein_coding	No	No	Yes	Yes*	5	rs4793938

HOXB8	17:46688739:46692478	4.45E-01	NA	protein_coding	Yes	No	No	Yes	3	rs4793938
HOXB9	17:46698518:46703839	1.02E-01	7.25E-01	protein_coding	No	No	No	Yes	2	rs4793938
HOXB-AS1	17:46620913:46628610	NA	NA	antisense	Yes	Yes	Yes[§]	Yes*	6	rs7225995;rs4793938;rs35533148
HOXB-AS2	17:46634624:46637695	NA	NA	antisense	Yes	No	No	Yes*	3	rs7225995;rs4793938
HOXB-AS3	17:46626992:46683776	NA	NA	antisense	No	No	Yes	Yes*	3	rs7225995;rs4793938
HOXB-AS4	17:46706037:46712294	NA	NA	antisense	No	No	Yes	Yes	2	rs4793938
INCA1	17:4891425:4900905	8.26E-09	NA	protein_coding	Yes	No	Yes	Yes*	5	rs12603055;rs2586527;rs427703;rs2243090;rs7208035;rs4609912
KIF1C	17:4901243:4931696	4.71E-01	3.70E+00	protein_coding	Yes	No	Yes	Yes*	5	rs427703;rs12603055;rs2586527;rs12600367;rs7208035;rs2243090;rs4609912
MINK1	17:4736683:4801356	1.00E+00	NA	protein_coding	Yes	Yes	Yes	Yes*	7	rs4609912;rs2243090;rs12603055;rs2586527;rs427703;rs12600367
MIR10A	17:46656992:46659621	NA	NA	sense_intronic	No	No	Yes	Yes*	3	rs7225995;rs4793938
MIR193A	17:29887015:29887102	NA	NA	miRNA	No	No	Yes	Yes	2	rs56161228
MIR196A1	17:46709852:46709921	NA	NA	miRNA	No	No	No	No	0	rs4793938
MIR4724	17:29861901:29861989	NA	NA	miRNA	No	No	No	No	0	rs56161228
MIS12	17:5389605:5394134	2.06E-02	2.15E+00	protein_coding	Yes	No	No	No	2	rs12603055;rs2586527;rs12600367
NF1	17:29421945:29709134	1.00E+00	-1.15E+00	protein_coding	Yes	No	No	No	4	rs56161228
NUP88	17:5264258:5323480	4.70E-06	2.13E-01	protein_coding	Yes	No	No	No	2	rs7208035;rs2243090;rs4609912;rs12603055;rs12600367
PFN1	17:4848947:4852356	6.89E-01	NA	protein_coding	No	No	No	Yes	2	rs2243090;rs12603055;rs2586527;rs7208035
PLD2	17:4710391:4726729	8.13E-14	NA	protein_coding	Yes	No	No	Yes*	4	rs4609912;rs2243090
PNPO	17:46018872:46025654	3.72E-05	1.79E-01	protein_coding	Yes	No	No	No	2	rs35533148;rs7225995;rs4793938
RAB11FIP4	17:29718642:29865236	9.94E-01	4.27E+00	protein_coding	No	No	No	Yes	3	rs56161228

RABEP1	17:5185558:5289129	9.83E-01	1.27E+00	protein_coding	Yes	No	No	No	3	rs7208035
RN7SL171P	17:4778921:4779205	NA	NA	misc_RNA	No	No	No	No	0	rs2243090;rs4609912
RN7SL45P	17:29845450:29845745	NA	NA	misc_RNA	No	No	No	No	0	rs56161228
RN7SL784P	17:4786270:4786591	NA	NA	misc_RNA	No	No	No	No	0	rs2243090;rs4609912
RNF167	17:4843303:4848517	1.29E-01	-1.69E-01	protein_coding	Yes	No	Yes	Yes*	6	rs2243090;rs12603055;rs2586527;rs7208035;rs4609912;rs427703;rs12600367
RNU6ATAC7P	17:29890787:29890913	NA	NA	snRNA	No	No	Yes	Yes	2	rs56161228
RP11-311F12.1	17:19622372:19625741	NA	NA	lincRNA	Yes	No	No	Yes	2	rs4646785
RP11-311F12.2	17:19640995:19641855	NA	NA	antisense	No	No	No	Yes	1	rs4646785
RP11-333E1.2	17:5143803:5144838	NA	NA	lincRNA	Yes	No	No	No	1	rs7208035
RP11-357H14.17	17:46713285:46724385	NA	NA	lincRNA	No	No	No	Yes	1	rs4793938
RP11-433M22.1	17:46454624:46457732	NA	NA	sense_intronic	No	No	No	No	0	rs35533148
RP11-433M22.2	17:46537732:46543402	NA	NA	lincRNA	No	No	Yes	Yes	2	rs35533148
RP11-46I8.1	17:4963651:4965418	NA	NA	pseudogene	No	No	No	No	0	rs12603055;rs12600367;rs7208035
RP11-46I8.3	17:4978973:4981408	NA	NA	antisense	No	No	No	Yes*	2	rs12603055;rs12600367;rs7208035
RP11-6N17.4	17:45968621:45973178	NA	NA	processed_transcript	Yes	No	No	No	1	rs35533148;rs4793938;rs7225995
RP11-81A22.5	17:4704454:4710308	NA	NA	lincRNA	Yes	No	No	No	1	rs427703;rs2243090
RP11-893F2.13	17:48235694:48238637	NA	NA	antisense	No	No	No	No	0	rs12939159
RP11-893F2.14	17:48240884:48242677	NA	NA	antisense	No	No	No	No	0	rs12939159
RP5-1050D4.2	17:4876146:4877976	NA	NA	antisense	No	No	No	Yes*	2	rs12603055;rs2586527;rs427703
RP5-1050D4.3	17:4871290:4872117	NA	NA	antisense	No	No	No	No	0	rs12603055;rs2586527;rs427703
RP5-1050D4.4	17:4889761:4890619	NA	NA	antisense	No	Yes	No	No	1	rs12603055;rs2586527;rs427703

RP5-1050D4.5	17:4891001:4891741	NA	NA	antisense	Yes	No	No	No	1	rs12603055;rs2586527;rs427703;rs7208035;rs2243090
RPAIN	17:5322961:5336196	6.36E-06	-3.03E-01	protein_coding	Yes	No	No	No	3	rs2243090
RPL9P28	17:46768478:46769056	NA	NA	pseudogene	No	No	No	No	0	rs4793938
SCIMP	17:5112256:5138155	1.26E-02	4.32E-01	protein_coding	Yes	No	No	No	2	rs12603055;rs12600367;rs7208035
SCRN2	17:45915058:45918699	6.56E-07	-3.00E-01	protein_coding	Yes	No	No	No	3	rs35533148;rs7225995;rs4793938
SGCA	17:48241575:48253292	1.94E-01	2.85E-01	protein_coding	Yes	No	No	No	2	rs12939159
SKAP1	17:46210802:46507637	3.02E-04	NA	protein_coding	No	Yes	Yes§	Yes*	6	rs35533148;rs7225995
SLC25A11	17:4840425:4843546	6.66E-01	6.64E-02	protein_coding	Yes	No	No	Yes*	4	rs2243090;rs12603055;rs2586527;rs7208035;rs4609912;rs427703;rs12600367
SLC47A1	17:19398698:19482347	1.22E-09	2.42E-01	protein_coding	Yes	No	No	No	2	rs4646785
SLC47A2	17:19581601:19622292	9.02E-14	-2.56E-01	protein_coding	Yes	No	Yes	Yes*	6	rs4646785
SLC52A1	17:4935895:4955304	2.74E-05	2.34E-02	protein_coding	Yes	No	No	Yes	3	rs12603055;rs12600367;rs7208035;rs2243090;rs2586527;rs427703
SNX11	17:46180719:46200436	3.86E-05	1.40E-01	protein_coding	Yes	No	No	No	2	rs35533148
SP6	17:45922279:45933240	2.29E-02	-1.13E+00	protein_coding	Yes	No	No	No	3	rs35533148;rs4793938
SPAG7	17:4862521:4871167	1.15E-03	1.96E-02	protein_coding	Yes	No	No	Yes*	4	rs2243090;rs7208035;rs12603055;rs2586527;rs427703;rs4609912;rs12600367
TBKBP1	17:45771447:45789416	3.84E-02	-1.55E+00	protein_coding	Yes	No	No	No	3	rs35533148;rs4793938
U3	17:46459734:46459948	NA	NA	snoRNA	No	No	No	No	0	rs35533148
ULK2	17:19674142:19771249	1.30E-05	-7.85E-01	protein_coding	Yes	No	No	No	3	rs4646785
USP6	17:5019733:5078329	1.76E-18	-1.13E+00	protein_coding	Yes	No	No	No	3	rs2243090;rs7208035
XYLT2	17:48423453:48440499	3.37E-01	1.14E+00	protein_coding	Yes	No	No	No	2	rs12939159
Y_RNA	17:46556459:46556560	NA	NA	misc_RNA	No	No	Yes	Yes*	3	rs35533148;rs7225995

ZFP3	17:4981543:4999669	5.84E-04	8.18E-01	protein_coding	No	No	No	Yes*	3	rs12603055;rs12600367;rs7208035
ZMYND15	17:4643319:4649411	1.44E-05	1.93E-01	protein_coding	No	Yes	No	No	2	rs12603055;rs2586527
ZNF232	17:5008836:5026411	4.35E-04	NA	protein_coding	Yes	Yes	No	No	3	rs12600367;rs7208035;rs2243090;rs12603055;rs2586527
AL121899.1	20:2137858:2137927	NA	NA	miRNA	No	No	No	No	0	rs6106210;rs6113033
RPL7P2	20:2056201:2056468	NA	NA	pseudogene	No	No	No	No	0	rs6106210
STK35	20:2082257:2157684	9.18E-01	3.88E-01	protein_coding	Yes	No	Yes	Yes*	6	rs6106210;rs6113033
AL035610.2	21:29488413:29600986	NA	NA	lincRNA	No	No	Yes	Yes	2	rs7278583;rs118737
AIFM3	22:21319396:21335649	1.96E-10	NA	protein_coding	No	No	No	Yes	2	rs756878
LZTR1	22:21333751:21353327	3.38E-52	-4.18E-01	protein_coding	Yes	No	No	No	3	rs756878
XXbac-B135H6.15	22:21311380:21318967	NA	NA	lincRNA	No	No	No	No	0	rs756878
XXbac-B135H6.18	22:21335650:21336044	NA	NA	lincRNA	No	No	No	No	0	rs756878

Table S4. Gene tiers for coding and non-coding variant prioritisation. The tier is determined by the total score of each gene in each prioritisation. Additionally, the number of genes and the gene ID are displayed for each tier, both for prioritisation based on coding variants and prioritisation based on non-coding variants. Tiers for non-coding variants prioritization: tier 1 (6 - 9), tier 2 (3 - 5), tier 3 (0 - 2). Tiers for coding variants prioritization: tier 1 (4 - 5), tier 2 (2 - 3), tier 3 (0 - 1).

	**Coding variants prioritization**		**Non-coding variants prioritization**	
**Tier (total score)**	**# Genes**	**Gene ID**	**# Genes**	**Gene ID**
Tier 1	5	ENO3, EPS8L2, FNDC3B, KAZALD1, **MINK1**	28	ABCA2, AP4E1, CAMTA2, CELF1, EIF3A, IQCH, **MINK1**, TP53INP1, ALDH3A1, CD46, CLIC3, ERP44, FNBP4, GALNT6, GP1BA, HOXB2, HOXB-AS1, INVS, LOX, NANOS1, RNF167, RNGTT, RPLP2, SKAP1, SLC25A22, SLC47A2, SMAD3, STK35
Tier 2	14	ALDH3A1, CAMTA2, INTS8, LCNL1, SLC52A1, SMAD3, C8G, C9orf141, HOXB1, INVS, KIF6, LCN12, PIDD, P53INP1	111	CACYBP, CHRNE, COL1A1, EPS8L2, FAM45A, FNDC3B, FOXO1, HOXB1, HOXB7, INCA1, INTS8, KIF1C, LCN12, LRP1B, NDUFAF6, NUP160, PDDC1, PIDD, PKHD1, PSMC3, SLC25A37, SPPL2A, TFAP2B, TMEM212, AAGAB, C8G, C9orf141, CEND1, CTD-2366F13.1, ENO3, KBTBD4, KLF5, LCNL1, MTRF1, NF1, PIBF1, PLD2, PNPLA2, RAPSN, SLC25A11, SLC39A13, SPAG7, STX17, TALDO1, TEX10, ZNF469, COL5A1, AC004771.1, AL133318.1, AL157788.1, AP006621.9, ARHGAP1, ATP1B1, B4GALNT2, BORA, C17orf107, C1orf132, C1QTNF4, C9orf142, CCNE2, CHID1, CR1L, EDF1, FAM206A, FBXW5, FST, GRIN1, HOXB3, HOXB8, HOXB-AS2, HOXB-AS3, IQCH-AS1, LINC00598, LINC00970, LZTR1, MADD, MAN1B1, MIR10A, MOCS2, MRPS14, MTCH2, MYBPC3, NDUFS3, PACSIN3, PLD1, PLEKHF2, POU6F1, RAB11FIP4, RABEP1, RABGAP1L, RC3H1, RP11-108K3.1, RP11-473E2.4, RP11-507J18.1, RP11-750H9.5, RP11-793I11.1, RP11-94D20.1, RPAIN, SCRN2, SKOR1, SLC52A1, SP6, SRFBP1, TBKBP1, TMEM212-AS1, TRAF2, ULK2, USP6, Y_RNA, ZFP3, ZNF232
Tier 3	4	PDDC1, PKHD1, PNPLA2, SLC39A13	167	AC090627.1, AGBL2, AIFM3, AL035610.2, AP006621.5, B4GALNT4, C8orf37, C9orf172, CD151, CDK5RAP3, COPZ2, CXCL16, DCAF13P3, DDB2, DIS3, DPP7, DPY19L4, EFCAB4A, ELF1, ENTPD4, FTH1P5, GGT6, HILS1, HOXB4, HOXB5, HOXB6, HOXB9, HOXB-AS4, KIF6, KRTAP5-3, LA16c-444G7.1, MAP2K5, MIR193A, MIS12, MSANTD3, NAA16, NELFB, NR1H3, NUP88, PFN1, PHPT1, PNPO, POLR2L, PTGDS, RAC1P3, RBMS3, RNU6ATAC7P, RP11-220D10.1, RP11-229P13.19, RP11-311F12.1, RP11-339N8.1, RP11-342M21.2, RP11-433M22.2, RP11-435O11.5, RP11-46I8.3, RP11-473E2.3, RP4-753D5.3, RP5-1018K9.1, RP5-1050D4.2, RPS17P5, SCIMP, SFXN4, SGCA, SLC47A1, SNX11, SPI1, SRSF12, TMEFF1, TMEM245, TMEM80, TNFAIP8L3, WBP4, XYLT2, ZMYND15, AC003101.1, AC012146.7, AL079342.1, AP006621.1, AP006621.6, AP006621.8, CELA1, COL5A1-AS1, CTC-524C5.2, CTC-756D1.2, FAM180B, MIR3150A, NCLP1, PSMD10P3, PTPMT1, RNU6-307P, RP1-102G20.5, RP11-1391J7.1, RP11-229P13.25, RP11-311F12.2, RP11-333E1.2, RP11-347C18.4, RP11-357H14.17, RP11-502I4.3, RP11-6N17.4, RP11-81A22.5, RP11-863P13.3, RP5-1050D4.4, RP5-1050D4.5, RPS24P16, SDCCAG3P2, SUGT1P3, TPTE2P5, TUBBP2, AC005722.4, AC090559.1, AC090559.2, AC109333.10, AL121899.1, AL391384.1, ATP6V0CP1, CTD-2377D24.2, CTD-2377D24.8, MIR196A1, MIR4487, MIR4724, NANOGP5, OR7E155P, OR7E36P, OR7E37P, RLIMP1, RN7SL141P, RN7SL171P, RN7SL45P, RN7SL652P, RN7SL75P, RN7SL784P, RNA5SP340, RNA5SP66, RNU1-54P, RNU5E-10P, RNU6-1302P, RNU6-57P, RNU6-79P, RP11-108L7.15, RP11-229P13.2, RP11-29B11.5, RP11-347C18.1, RP11-347C18.3, RP11-347C18.5, RP11-394B5.2, RP11-423E7.1, RP11-433M22.1, RP11-454L1.2, RP11-46I8.1, RP11-473E2.2, RP11-498J9.2, RP11-692M12.5, RP11-750H9.7, RP11-893F2.13, RP11-893F2.14, RP5-1050D4.3, RPL7P2, RPL9P28, RPS2P35, SNORA19, SNORA52, snoU13, TMEM212-IT1, U3, UPF3AP3, XXbac-B135H6.15, Xbac-B135H6.18

Table S5. Potential roles of tier 1 genes in the pathogenesis of keratoconus. PubMed identifiers (PMID) of studies supporting the evidences are provided.

Gene	Gene Prioritisation	Position (GRCh38)	Relevant evidence related to keratoconus
ABCA2	Non-coding	9:139901686:139923367	None reported
ALDH3A1	Non-coding	17:19641297:19652256	Protects corneal cells from both apoptosis and DNA damage [PMID: 36982917]. It is thought to promote resistance to UV and 4-hydroxy-2-nonenal-induced oxidative damage in the cornea [PMID: 12706498]. Genetic polymorphisms associated with keratoconus [PMID: 35011749, PMID: 33596621, PMID: 37990318]
AP4E1	Non-coding	15:51200869:51298097	Genetic polymorphisms associated with keratoconus [PMID: 33649486]
CAMTA2	Non-coding	17:4871287:4890960	None reported
CD46	Non-coding	1:207925402:207968858	Plays an important role in the pathogenesis of adenoviral keratoconjunctivitis [PMID: 30046247]
CELF1	Non-coding	11:47487496:47587121	The inactivation of this gene in mice leads to misregulation of post-transcriptional gene expression control in the lens and cataract [PMID: 37503005]
CLIC3	Non-coding	9:139889087:139891255	None reported
EIF3A	Non-coding	10:120794356:120840316	Genetic polymorphisms associated with keratoconus [PMID: 33649486]
ENO3	Coding	17:4851387:4860426	Genetic polymorphisms associated with keratoconus [PMID: 33649486]
EPS8L2	Coding	11:694438-727727	None reported
ERP44	Non-coding	9:102741461-102861322	Related with a deficit in lacrimal secretion [PMID: 18813072]
FNBP4	Non-coding	11:47738072-47788995	None reported
FNDC3B	Coding	3:171757418-172119455	Genetic polymorphisms associated with keratoconus [PMID: 33649486, PMID: 23291589]
GALNT6	Non-coding	12:51745031-51786651	Genetic polymorphisms associated with keratoconus [PMID: 33649486]
GP1BA	Non-coding	17:4835592-4838325	Genetic polymorphisms associated with keratoconus [PMID: 33649486]
HOXB2	Non-coding	17:4661825646623441	Genetic polymorphisms associated with keratoconus [PMID: 33649486]
HOXB-AS1	Non-coding	17:46620913-46628610	None reported
INVS	Non-coding	9:102861538-103063282	None reported

IQCH	Non-coding	15:67547138-67794598	Associated with corneal resistance factor [PMID: 33311554]
KAZALD1	Coding	10:102821598-102827888	Involved in extracellular matrix formation [PMID: 32193507]. Genetic polymorphism associated with corneal curvature [PMID: 32193507], corneal resistance factor [PMID:33311554], intraocular pressure [PMID:29617998], refractive error [PMID:32231278], and spherical equivalent [PMID:32352494].
LOX	Non-coding	5:121398890-121413980	Involved in the cross-linking of collagen and elastin [PMID: 27350955]. Its expression is downregulated in keratoconus [PMID: 38483957], and genetic polymorphisms have been associated with keratoconus [PMID: 36748061, PMID: 22661479, PMID: 25735481, PMID: 25675348, PMID: 24502826, PMID: 26713757]
MINK1	Coding/Non-coding	17:4833340-4898061	Involved in WNT-mediated planar cell polarity establishment and maintenance [PMID: 22037766].
NANOS1	Non-coding	10:120789228-120793854	None reported
RNF167	Non-coding	17:4843303-4848517	Gene expression associated with intraocular pressure [PMID: 37239387]
RNGTT	Non-coding	6:89319985-89673348	None reported
RPLP2	Non-coding	11:809647-812880	None reported
SKAP1	Non-coding	17:46210802-46507637	Genetic polymorphisms associated with keratoconus [PMID: 33649486]
SLC25A22	Non-coding	11:790475-798316	Genetic polymorphisms associated with central corneal thickness [PMID:35446358], corneal resistance factor [PMID:35446358], and intraocular pressure [PMID:29617998]
SLC47A2	Non-coding	17:19581601-19622292	None reported
SMAD3	Non-coding	15:67356101-67487533	Required for maintenance of corneal epithelial identity and homeostasis [PMID: 33462242]
STK35	Non-coding	20:2082257-2157684	Genetic polymorphisms associated with KC and overexpressed in keratoconus corneas [PMID: 33649486]
TP53INP1	Non-coding	8:95938200-95961639	Upregulated expression promotes cataractogenesis in age-related cataract [PMID: 32841649]

Table S6. Approved drugs whose target is any of the genes in tier 1 or 2, indicating whether this gene has been prioritised based on coding variants, non-coding variants, or both.

Drug ID	Drug Name	Drug Status	Target Gene	Gene priorisation
DB00048	Collagenase clostridium histolyticum	approved, investigational	COL1A1	Non-Coding
DB00122	Choline	approved, nutraceutical	PLD1, PLD2	Non-Coding
DB00142	Glutamic acid	approved, nutraceutical	GRIN1	Non-Coding
DB00157	NADH	approved, nutraceutical	ALDH3A1, NDUFS3	Both
DB00193	Tramadol	approved, investigational	GRIN1	Non-Coding
DB00228	Enflurane	approved, investigational, vet_approved	GRIN1	Non-Coding
DB00289	Atomoxetine	approved	GRIN1	Non-Coding
DB00312	Pentobarbital	approved, investigational, vet_approved	GRIN1	Non-Coding
DB00333	Methadone	approved	GRIN1	Non-Coding
DB00418	Secobarbital	approved, vet_approved	GRIN1	Non-Coding
DB00454	Meperidine	approved	GRIN1	Non-Coding
DB00659	Acamprosate	approved, investigational	GRIN1	Non-Coding
DB00843	Donepezil	approved	GRIN1	Non-Coding
DB00874	Guaifenesin	approved, investigational, vet_approved	GRIN1	Non-Coding
DB01043	Memantine	approved, investigational	GRIN1	Non-Coding
DB01050	Ibuprofen	approved	GP1BA	Both
DB01173	Orphenadrine	approved	GRIN1	Non-Coding
DB01174	Phenobarbital	approved, investigational	GRIN1	Non-Coding
DB01238	Aripiprazole	approved, investigational	GRIN1	Non-Coding
DB01593	Zinc	approved, investigational	C8G	Non-Coding
DB01708	Prasterone	approved, investigational, nutraceutical	GRIN1	Non-Coding
DB03017	Lauric acid	approved, experimental	C8G	Non-Coding
DB03929	D-Serine	approved, experimental	GRIN1	Non-Coding
DB04272	Citric acid	approved, nutraceutical, vet_approved	C8G	Non-Coding
DB04896	Milnacipran	approved, investigational	GRIN1	Non-Coding
DB06151	Acetylcysteine	approved, investigational	GRIN1	Non-Coding
DB09213	Dexibuprofen	approved, investigational	GP1BA	Both
DB09409	Magnesium acetate tetrahydrate	approved	GRIN1	Non-Coding
DB09481	Magnesium carbonate	approved, investigational	GRIN1	Non-Coding

DB11338	Clove oil	approved, nutraceutical	COL1A1	Non-Coding
DB11823	Esketamine	approved, investigational	GRIN1	Non-Coding
DB12010	Fostamatinib	approved, investigational	MINK1, STK35, ULK2	Both
DB12872	Vonicog alfa	approved, investigational	COL1A1	Non-Coding
DB13133	Von Willebrand factor human	approved, investigational	COL1A1	Non-Coding
DB13146	Fluciclovine (18F)	approved	GRIN1	Non-Coding
DB14006	Choline salicylate	approved, nutraceutical	PLD1, PLD2	Non-Coding
DB14487	Zinc acetate	approved, investigational	C8G	Non-Coding
DB16628	Fosdenopterin	approved	MOCS2	Non-Coding

Table S7. Estimated statistical power for both the meta-analysis and the replication analysis in the Spanish cohort accordingly with different minor allele frequencies (MAF) and odds ratios (OR).

	**MAF**	**OR**					
		1.20	1.50	2.00	2.50	3.00	
**Meta-analysis**	0,050	0,048	0,998	1,000	1,000	1,000	**Statistical power**
	0,100	0,388	1,000	1,000	1,000	1,000	
	0,200	0,905	1,000	1,000	1,000	1,000	
	0,300	0,984	1,000	1,000	1,000	1,000	
	0,400	0,994	1,000	1,000	1,000	1,000	
	0,500	0,995	1,000	1,000	1,000	1,000	
**Spanish cohort as replication cohort**	0,050	0,093	0,270	0,638	0,867	0,960	
	0,100	0,130	0,440	0,864	0,980	0,998	
	0,200	0,189	0,637	0,966	0,998	1,000	
	0,300	0,226	0,718	0,982	0,999	1,000	
	0,400	0,244	0,741	0,982	0,999	1,000	
	0,500	0,245	0,725	0,974	0,997	1,000	

Table S8. Parameters applied to perform the SNP2GENE analysis using FUMA.

	Parameter	Value
**Versions**	FUMA	v1.5.3
	MAGMA	v1.08
	GWAScatalog	e0_r2022-11-29
	ANNOVAR	2017-07-17
**General parameters**	N	122,332
	exMHC	NONE
	ensembl	v92
	gene type	all
	leadP	5.00E-08
	gwasP	0.05
	r2	0.6
	r2 (2)*	0.1
	refpanel	1KG/Phase3
	population	EUR
	MAF	0.01
	refSNPs	1
	mergeDist	250
**Magma parameters**	MAGMA	YES
	Window	NONE
	Exp	GTEx (v8): gtex_v8_ts_avg_log2TPM, gtex_v8_ts_general_avg_log2TPM
**Positional mapping**	posMap	YES
	posMapWindowSize	10
	posMapAnnot	NA
	posMapCADDth	NONE
	posMapRDBth	NA
	posMapChr15	NA
	posMapChr15Max	NA
	posMapChr15Meth	NA
	posMapAnnoDs	NA
	posMapAnnoMeth	NA
**eQTL mapping**	eqtlMap	YES
	eqtlMaptss	GTEx (v8): Cells_Cultured_fibroblasts.txt.gz, Skin_Not_Sun_Exposed_Suprapubic.txt.gz, Skin_Sun_Exposed_Lower_leg.txt.gz
	eqtlMapSig	YES
	eqtlMapP	YES
	eqtlMapCADDth	NONE
	eqtlMapRDBth	NA
	eqtlMapChr15	NA
	eqtlMapChr15Max	NA
	eqtlMapChr15Meth	NA
	eqtlMapAnnoDs	NA
	eqtlMapAnnoMeth	NA

**Chromatin interaction mapping**	ciMap	YES
	ciMapBuiltin	EP/FANTOM5/EP_correlation_cell_type_oneway.txt.gz
	ciMapFileN	NONE
	ciMapFiles	NA
	ciMapFDR	0.000001
	ciMapPromWindow	250-500
	ciMapRoadmap	E055:E056:E057:E058:E059:E061
	ciMapEnhFilt	NONE
	ciMapPromFilt	NONE
	ciMapCADDth	NONE
	ciMapRDBth	NA
	ciMapChr15	NA
	ciMapChr15Max	NA
	ciMapChr15Meth	NA
	ciMapAnnoDs	NA
	ciMapAnnoMeth	NA

Table S9. Parameters considered for the in-depth gene prioritisation, including context (gene if referred to the gene, variant if referred to the single nucleotide polymorphism, SNP), described effect, range, and meaning.

Parameter	Context	Predicted effect	Score range	Note
CADD	Variant	benign to pathogenic	[1, 99]	Score above 10 is considered for potentially pathogenic variants
RDB	Variant	Functionality	1a	eQTL + TF binding + matched TF motif + matched DNase Foprint + DNase peak.
			1b	eQTL + TF binding + any motif + DNase Footprint + Dnase peak.
			1c	eQTL + TF binding + matched TF motif + DNase peak.
			1d	eQTL + TF binding + any motif + DNase peak.
			1e	eQTL + TF binding + matched TF motif.
			1f	eQTL + TF binding / DNase peak.
			2a	TF binding + matched TF motif + matched DNase Footprint + DNase peak.
			2b	TF binding + any motif + DNase Footprint + DNase peak.
			2c	TF binding + matched TF motif + DNase peak.
			3a	TF binding + any motif + DNase peak.
			3b	TF binding + matched TF motif.
			4	TF binding + DNase peak.
			5	TF binding or DNase peak.
			6	Other.
pLI	Gene	loss-of-function intolerance	[0, 1]	Score > 0.9 is considered intolerance to loss-of-function
ncRVIS	Gene	Non-coding residual variation intolerance	[- / +]	Negative (-) values for the score imply less functional variation than expected, positive (+) values for the score imply more functional variation than expected
