# Supplementary material for: Comprehensive Evaluation of the Genetic Basis of Keratoconus: New Perspectives for Clinical Translation
Source: Invest Ophthalmol Vis Sci. 2024 Oct 22;65(12):32. doi: 10.1167/iovs.65.12.32 (PMC11500050; doi:10.1167/iovs.65.12.32)
Supplement: Supplement 3 [file iovs-65-12-32_s003.pdf]

SUPPLEMENTARY METHODS

Comprehensive evaluation of the genetic basis of keratoconus: new perspectives for clinical translation

Miriam Cerván-Martín, Inmaculada Higueras-Serrano, Sara González-Muñoz, Andrea Guzmán-Jiménez, Blas Chaves-Urbano, Rogelio J. Palomino-Morales, Arancha Poo-López, Luis Fernández-Vega Cueto, Jesús Merayo-Lloves, Ignacio Alcalde, Lara Bossini-Castillo, F. David Carmona

Cohort selection criteria

All KC participants were recruited by the Fernandez-Vega Ophthalmological Institute (Oviedo, Spain). A comprehensive ophthalmological examination was conducted in all cases, which included analyses of best-corrected visual acuity, slit-lamp biomicroscopy (Topcon, Tokyo, Japan), and anterior segment optical coherence tomography (Casia2, OCT, Tomey, Nagoya, Japan). Additionally, the family medical history of each individual was also reviewed. KC patients without systemic associations were recruited and categorised according to the Amsler-Krumeich classification.[1, 2] All patients exhibited one or more signs associated with KC, including central corneal protrusion (<1.25 mm from the centre) or paracentral protrusion (a ring of over 1.25 mm around the central corneal area), thinning of the cornea, positive Munson's sign, Vogt's striae, and/or Fleischer's ring during slit-lamp examination. Similarly, signs of KC were also identified in corneal tomography (displacement of the corneal apex, thickness at the thinnest point, asymmetric topographic pattern) in at least one eye. Corneal tomography was performed using swept-source optical coherence tomography (Casia2 OCT). Curvature keratometry (K2s), flat keratometry (K1), average keratometry (Avg K), and best-fit sphere diameter (BFS) data were collected from the anterior and posterior corneal surfaces, apex pachymetry, thinnest pachymetry, and manifest refraction of spherical equivalent (SEQ). Ks, Kf, and Avg K represent keratometry in the curved axis, flat axis, and the average of curved and flat keratometry values, respectively. The best-fit-sphere (BFS) diameter was 8 mm, a constant value in all measurements. Individuals suspected of KC (but without a definitive diagnosis) or with subclinical KC were excluded, as their clinical diagnostic criteria are confusing and controversial. Patients were advised not to wear rigid contact lenses for the 4 weeks preceding the ocular examination or soft contact lenses for 2 weeks prior. Stable and progressive types of KC patients were included. Progression criteria included an increase of at least 1 dioptre (D) in the steepest keratometry measurement obtained through corneal topography, an increase of 1 D or more in refractive astigmatism, or the loss of at least 2 lines of corrected distance visual acuity on two consecutive visits. Conversely, samples from healthy controls were provided by the National DNA Bank (University of Salamanca, Spain). The age, gender, and geographic origin of these individuals matched those of the KC cases.

Generation of genotype data and quality controls

Genomic DNA samples were extracted from peripheral blood mononuclear cells and genotyped at the genome-wide level using the Infinium™ Global Screening Array-24 v3.0 (GSA) in an iScan System (Illumina, Inc). This sophisticated high-throughput genotyping platform allows the assessment of over 700,000 carefully selected genetic variants, including tag polymorphisms, relevant clinical markers and quality control variants (including markers for ancestry). Chromosome positions were assigned according to the Genome Reference Consortium Human Build 38 (GRCh38), and genotype calling was performed using the Genotyping Module (v.2.0) implemented in the GenomeStudio software (Illumina, Inc).

The software PLINK v.1.9[3] and R were used for the quality controls (QC), which comprised the following: 1) Genetic variants with a cluster separation < 0.4 were removed, along with INDELs and rare variants with minor allele frequencies (MAF) < 0.01; 2) SNPs with call rates < 0.98 and genotype distributions deviating from Hardy–Weinberg equilibrium (HWE) in controls (P < 0.001) were filtered out; 3) samples with < 95% successfully called SNPs and one subject per pair of first-degree relatives (identity by descent > 0.4) were removed; 4) population outliers (>4 standard deviations from the cluster centroids of each population) were identified through principal component (PC) analyses, which incorporated 2,921 ancestry-informative markers contained in the GSA chip. A plot of the first two PCs against each other for samples that successfully met all QCs criteria is displayed in **Supplementary Fig. S1**.

Imputation methods

To increase the genetic coverage of the GWAS dataset, a genotype imputation for chromosomes 1-22 was performed using the haplotype data of the 'NHLBI Trans-OMICs for Precision Medicine' (TOPMed) programme (freeze 5) as reference panel in the TOPMed Imputation Server.[4, 5]

Singletons, rare variants (MAF < 0.01), and polymorphisms with call rates lower than 98% were excluded from the dataset. Similarly, SNPs whose genotype frequencies deviated significantly from HWE (P < 0.001) were also

removed. Furthermore, only SNPs with a very reliable imputation quality metric (Rsq > 0.9) were included in the analysis.

Statistics and reproducibility

Genetic Association Study (GAS) Power Calculator was used in order to determine the statistical power of the study. This online tool applies the methods described in Skol *et al.*[6] assuming additive genetic effects (**Supplementary Table S7**).

PLINK and R were also used for the case-control analyses. First, we analysed the Spanish cohort by logistic regression on the best-guess genotypes (Rsq > 0.9), adding the 10 first PCs and sex (male or female) as covariates and assuming additive effects. Subsequently, we conducted a meta-analysis between our Spanish results and those reported by Hardcastle *et al*.[7] (available in the NHGRI-EBI GWAS Catalogue) using the inverse variance method under a fixed effects model. I^2 and Cochran's Q tests were applied to evaluate the heterogeneity in effect sizes between the two analysed data sets.

Odds ratios (OR) and 95% confidence intervals (CI) were calculated for the statistical analyses, and the significance level for the meta-analysis was set at the genome-wide level (P < 5E−08). An in-house R script was used to generate the Manhattan plots and, for a more detailed exploration of associated regions, zoomed-in plots of those regions were created with LocusZoom.js.[8]

Polygenic risk score analysis

PRSs were computed using the software PRSice-2[9], considering the additive model for the effect allele (EA). The PRSice-2 tool allowed the calculation of the product of the number of EAs (0, 1, or 2) for each individual and the corresponding weight of each SNP. Linkage disequilibrium (LD) clumping was conducted using the following parameters: 500Kb window with the index SNP at the centre (--clump-kb 250), R2 > 0.1 (--clump-r2 0.1), and no P-value threshold (--clump-p 1). The empirical P-value was calculated using 1,000 permutations (--perm 1000). Additionally, different PRS models were fitted using PRSice-2 in order to select variants surpassing different p-

value thresholds in the base GWAS summary statistics (argument --bar-levels 5e-11, 5e-10, 5e-09, 5e-08, 5e-07, 5e-06, 5e-05, 0.0001, 0.001, 0.05, 0.1, 0.2, 0.3, 0.4, 0.5, 1). Ten PCs and sex were used as covariates. Thus, the model fit was defined as the R^2 of the full model (KC case or control ~ PRS + 10 PCs + sex) minus the R^2 of the null model (KC case or control ~ 10 PCs + sex). Following the PRS analysis, we used the pROC R package[10] to calculate the predictive capability of the model, generating the area under the receiver operating characteristic curve (AUC). Finally, by employing various risk percentile thresholds and analysing the statistical significance of each division through a χ^2 test, the model successfully identified individuals at high-risk.

We defined PRSs to predict the genetic predisposition of an individual to develop KC. Genomic data used for the training set were obtained from two distinct previously mentioned studies: KC summary statistics from Hardcastle *et al.*[7] and CCT summary statistics from Choquet *et al.*[11] The genomic information from these studies was used both individually (by calculating two separate PRS) and collectively (through an additional PRS, for which a meta-analysis between the genomic data of KC and CCT was conducted). The dataset of the Spanish cohort was used to evaluate the predictive capability of the model.

Functional annotation and gene prioritisation

The Functional Mapping and Annotation (FUMA) of GWAS online tool[12] was used to carry out the functional prioritisation of the significant associated variants reported in our meta-analysis. Specifically, the SNP2GENE function as implemented in FUMA was utilised to perform gene mapping using diverse transcriptomic and epigenomic databases. This approach provided valuable information about the genes affected by these variants. FUMA parameters are detailed in **Supplementary Table S8**.

To prioritise potential genes affected by the associated SNPs and proxies, we categorised all genes provided by FUMA into two groups: those affected by coding variants and those affected by non-coding variants. Subsequently, we assessed different parameters provided by FUMA for each gene group. For genes affected by coding variants, parameters such as gene probability of being loss-of-function intolerant (pLI), exonic function,

and the deleteriousness score (CADD) of the variants were evaluated. For genes affected by non-coding variants, we evaluated gene-related parameters, including pLI, gene non-coding residual variation intolerance (ncRVIS) score, gene type or chromatin interaction (CI) data, as well as variant-related parameters, such as expression quantitative trait *locus* (eQTL) effects, CADD score, and RegulomeDB (RDB) score. This prioritisation resulted in the establishment of 3 gene tiers based on the aforementioned parameters. **Supplementary Table S9** provides details on the meaning and range of the considered parameters. Finally, tissue-expression analysis for KC was performed using the Multi-Marker Analysis of GenoMic Annotation (MAGMA) integrated within FUMA, and the GENE2FUNCTION tool of FUMA was used to annotate the genes in a biological context.

Additionally, the Retrieval of Interacting Genes/Proteins (STRING) portal[13] was used to provide an illustrative picture of the putative functional role of the prioritised genes. We conducted enrichment analyses of protein-protein interactions (PPI), considering all genes included in tiers 1 and 2. Information about human phenotypes and diseases associated with those genes was also consulted.

Drug repositioning evaluation

Drug repositioning was considered to propose potential novel treatments for the therapeutic intervention of KC. For this assessment, drugs included in the DrugBank database[14] were screened, considering genes included in tier 1 and 2 as potential targets. Only approved treatments were regarded as potential candidate drugs for the management of KC disease. Additionally, action mechanisms and available literature were explored for those drugs whose indication or associated conditions were potentially relevant for KC.

REFERENCES OF SUPPLEMENTARY METHODS

1. Krumeich JH, Daniel J. [Live epikeratophakia and deep lamellar keratoplasty for I-III stage-specific surgical treatment of keratoconus]. *Klinische Monatsblatter fur Augenheilkunde* 1997;211:94-100.
2. Alio JL, Shabayek MH. Corneal higher order aberrations: a method to grade keratoconus. *J Refract Surg* 2006;22:539-545.
3. Chang CC, Chow CC, Tellier LC, Vattikuti S, Purcell SM, Lee JJ. Second-generation PLINK: rising to the challenge of larger and richer datasets. *GigaScience* 2015;4:7.
4. Das S, Forer L, Schonherr S, et al. Next-generation genotype imputation service and methods. *Nature genetics* 2016;48:1284-1287.
5. Taliun D, Harris DN, Kessler MD, et al. Sequencing of 53,831 diverse genomes from the NHLBI TOPMed Program. *Nature* 2021;590:290-299.
6. Skol AD, Scott LJ, Abecasis GR, Boehnke M. Joint analysis is more efficient than replication-based analysis for two-stage genome-wide association studies. *Nature genetics* 2006;38:209-213.
7. Hardcastle AJ, Liskova P, Bykhovskaya Y, et al. A multi-ethnic genome-wide association study implicates collagen matrix integrity and cell differentiation pathways in keratoconus. *Communications biology* 2021;4:266.
8. Boughton AP, Welch RP, Flickinger M, et al. LocusZoom.js: interactive and embeddable visualization of genetic association study results. *Bioinformatics* 2021;37:3017-3018.
9. Choi SW, O'Reilly PF. PRSice-2: Polygenic Risk Score software for biobank-scale data. *GigaScience* 2019;8.
10. Robin X, Turck N, Hainard A, et al. pROC: an open-source package for R and S+ to analyze and compare ROC curves. *BMC bioinformatics* 2011;12:77.
11. Choquet H, Melles RB, Yin J, et al. A multiethnic genome-wide analysis of 44,039 individuals identifies 41 new loci associated with central corneal thickness. *Communications biology* 2020;3:301.
12. Watanabe K, Taskesen E, van Bochoven A, Posthuma D. Functional mapping and annotation of genetic associations with FUMA. *Nature communications* 2017;8:1826.
13. Szklarczyk D, Franceschini A, Wyder S, et al. STRING v10: protein-protein interaction networks, integrated over the tree of life. *Nucleic acids research* 2015;43:D447-452.
14. Wishart DS, Feunang YD, Guo AC, et al. DrugBank 5.0: a major update to the DrugBank database for 2018. *Nucleic acids research* 2018;46:D1074-D1082.
